# Pseudo‐heterolepticity in Low‐Symmetry Metal‐Organic Cages

**DOI:** 10.1002/anie.202212392

**Published:** 2022-09-29

**Authors:** James E. M. Lewis

**Affiliations:** ^1^ School of Chemistry University of Birmingham Edgbaston Birmingham B15 2TT UK; ^2^ Previous address: Department of Chemistry Molecular Sciences Research Hub Imperial College London 82 Wood Lane London W12 0BZ UK

**Keywords:** Cage, Heteroleptic, Low-Symmetry, Metallosupramolecular, Self-Assembly

## Abstract

Heteroleptic metal‐organic cages, formed through integrative self‐assembly of ligand mixtures, are highly attractive as reduced symmetry supramolecular hosts. Ensuring high‐fidelity, non‐statistical self‐assembly, however, presents a significant challenge in molecular engineering due to the inherent difficulty in predicting thermodynamic energy landscapes. In this work, two conceptual strategies are described that circumvent this issue, using ligand design strategies to access structurally sophisticated metal‐organic hosts. Using these approaches, it was possible to realise cavity environments described by two inequivalent, unsymmetrical ligand frameworks, representing a significant step forward in the construction of highly anisotropic confined spaces.

## Graphical abstract

Heteroleptic metal‐organic cages are prized for their structural complexity and multi‐functionality. Controlling integrative self‐sorting processes necessary to achieve defined self‐assembly of these supramolecular hosts is a significant design challenge. In this work, ligand‐tethering strategies are described that allow access to metal‐organic cages with highly anisotropic cavities described by two inequivalent, unsymmetrical ligand scaffolds.

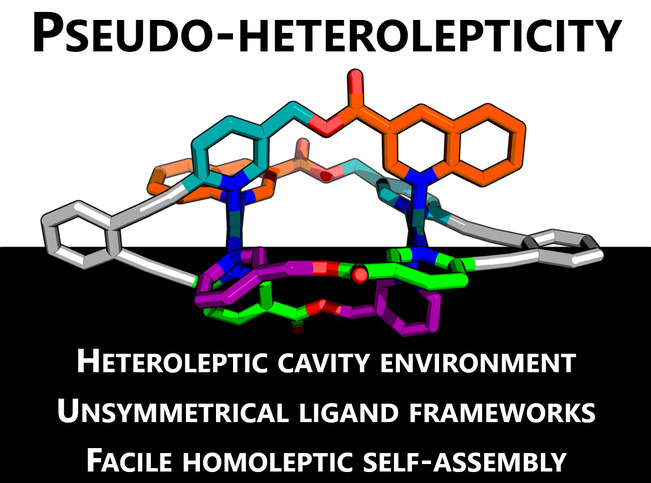

## Introduction

Metal‐organic self‐assembly has proven to be a powerful tool to construct functional supramolecular architectures from relatively simple components.^[1]^ Of particular interest are systems possessing internal cavities^[2]^ capable of binding ions or small molecules—typically referred to as metal‐organic cages (MOCs) or polyhedra (MOPs).^[3]^ The ability to bind guest species,^[4]^ such as drugs,^[5]^ pollutants^[6]^ and anions,^[7]^ within these cavities has been exploited for catalysis,^[8]^ stabilising reactive species^[9]^ and modulation of photophysical properties,^[10]^ amongst other applications.

**Figure 1:**
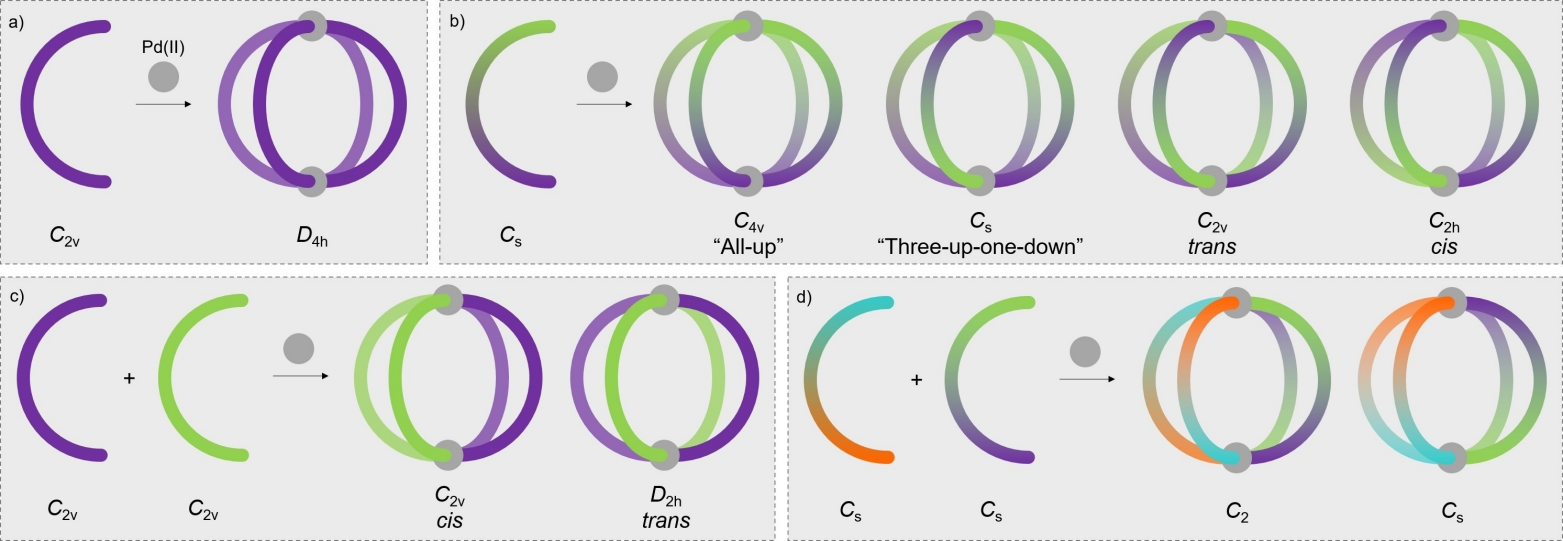
a) Self‐assembly of *C*_2v_‐symmetric ditopic ligands with square‐planar Pd^II^ ions leads to formation of Pd_2_L_4_ cages with (pseudo‐)*D*_4h_ symmetry. b) Ditopic ligands lacking bilateral symmetry (*C*_2_ rotation axis) can assemble into four possible Pd_2_L_4_ cage diastereoisomers. c) Integrative self‐assembly of ligand mixtures leading to selective formation of heteroleptic structures. d) Integrative self‐assembly of unsymmetrical ligands into heteroleptic cages has not been previously reported.

To advance the functionality of these systems, and move towards more structurally sophisticated assemblies, researchers have recently begun to develop strategies to access MOCs of reduced symmetry in order to tailor the shape and functionality of the cavity environment.^[11]^ Two main approaches have shown success in this endeavour. Employing unsymmetrical ligands will inherently generate MOCs of reduced symmetry (Figure 1b).^[12]^ Alternatively, the incorporation of more than one ligand type to generate heteroleptic,^[13]^ or mixed‐ligand, cages is also effective (Figure 1c).^[14]^ The latter approach has the added benefit of being able to introduce different endo‐/exohedral functional moieties into a single structure.^[15]^ For both of these strategies, ensuring high‐fidelity self‐sorting in the assembly process is essential to avoid formation of mixtures of cage structures, which makes their design challenging. It can further be imagined that combining heteroleptic and low‐symmetry strategies through the incorporation of multiple, unsymmetrical ligands into a single MOC in a defined manner (Figure 1d) would be especially difficult, which likely explains why no examples have been reported to date.

Herein are detailed two strategies to access cavity environments within MOCs that are described by two, unsymmetrical ligand structures. The first employs a covalent‐tethering strategy to specifically constrain ligand fragments in a *trans* orientation within a polytopic framework. The second exploits the geometrically‐enforced arrangement of ligands with *C*_s_ symmetry in the selective assembly of *C*_2h_—symmetry palladium nanocages (Figure 1b) within a previously unreported class of double‐cavity Pd_3_L_4_ cages. With both of these approaches, the cavities generated are heteroleptic environments—i.e. they are defined by two inequivalent, unsymmetrical ligand frameworks. With the cages as a whole assembled from one type of ligand, the term *pseudo‐heteroleptic* is proposed to describe these structures.

The significant advantage of these pseudo‐heterolepticity approaches lies in the ability to construct sophisticated, multi‐functional internal spaces without the requirement for integrative self‐sorting of multiple components, a prerequisite of traditional heteroleptic assemblies. The delineation of these strategies opens the door to develop more sophisticated and functional confined spaces in combination with facile self‐assembly.

## Results and Discussion

**Figure 2:**
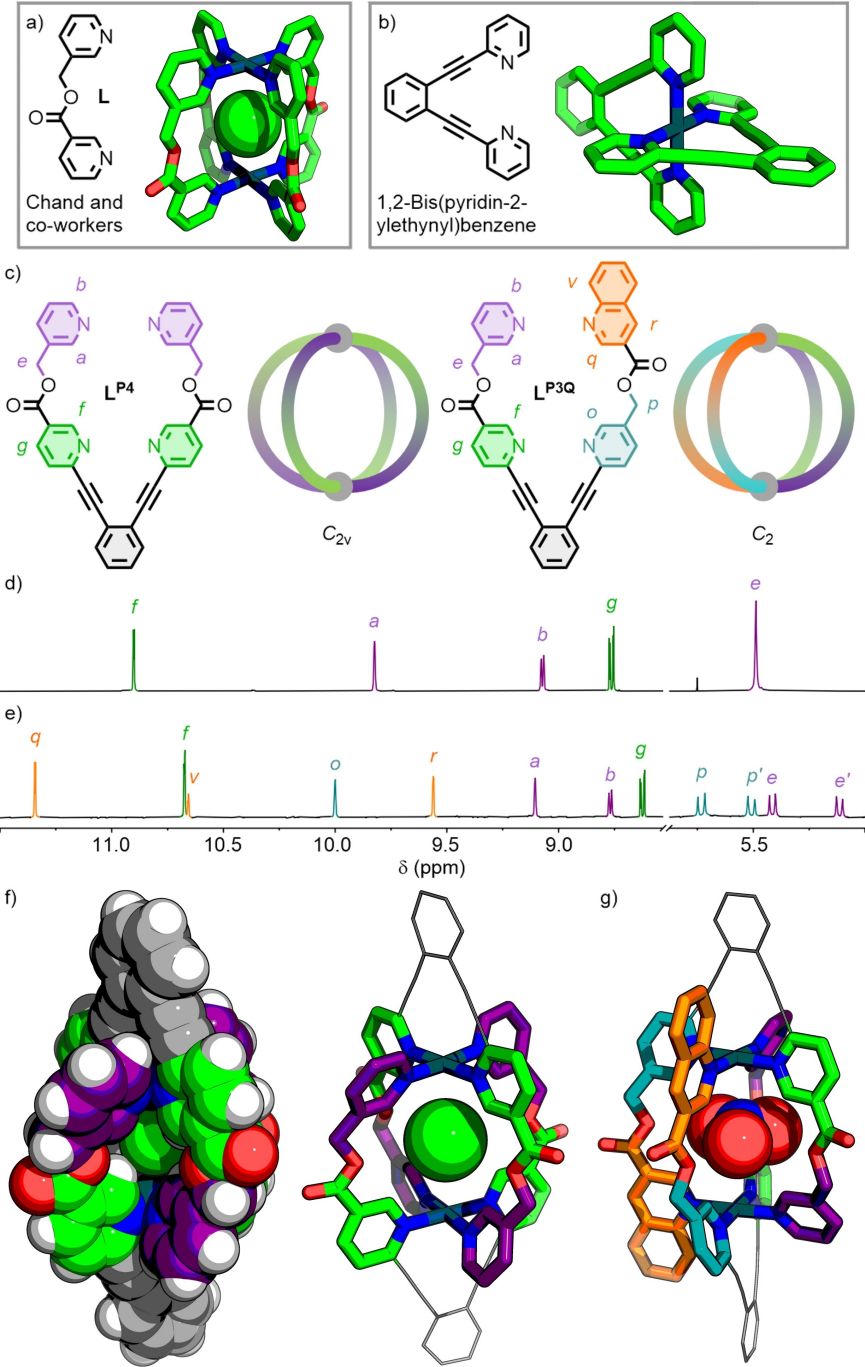
a) Chand's low‐symmetry ditopic ligand, **L**, and SCXRD structure of the dinuclear Pd^II^ cage structure [Pd_2_ **L**_4_⊃Cl]^3+^ (CCDC# 1941617; only *C*_4v_ isomer shown for clarity); b) 1,2‐bis(pyridin‐2‐ylethynyl)benzene and the SCXRD structure of its mononuclear Pd^II^ complex (CCDC# 185471); c) tetratopic ligands **L^P4^** and **L^P3Q^** with cartoon diagrams representing the relative orientations of the ligand fragments within **C^P4^** and **C^P3Q^**; partial ^1^H NMR spectra (500 MHz, *d*_6_‐DMSO, 298 K) of d) **C^P4^** and e) **C^P3Q^**; SCXRD structures of f) [Pd_2_(**L^P4^**)_2_⊃Cl]^3+^ and g) [Pd_2_(**L^P3Q^**)_2_⊃NO_3_]^3+^.

First demonstrated by McMorran and Steel,^[16]^ ditopic ligands are able to assemble with Pd^II^ ions to form quadruply‐stranded, Pd_2_L_4_‐type cage structures.^[17]^ For ligands with *C*_2v_ symmetry there is only one possible configurational isomer of these cages, with (pseudo‐)*D*_4h_ symmetry (Figure 1a). Ligands with reduced *C*_s_ symmetry, however, can assemble into four possible Pd_2_L_4_ diastereoisomers (Figure 1b). Unless sufficient directing effects are employed in the design of the ligand, a statistical mixture of these isomers will be generated.^[18]^ For example, Chand and co‐workers have previously reported the self‐assembly of ditopic ligand **L** (Figure 2a), which is unsymmetrical by virtue of the methylene ester backbone that separates the two coordinating pyridine units. Upon self‐assembly with Pd^II^, a statistical mixture of the four possible Pd_2_L_4_ cage isomers was observed to form both in solution and the solid state.^[19]^

This group,^[20]^ and others,[^21^, ^22^] have previously used geometric design principles within reduced symmetry, ditopic ligands to successfully bias the self‐assembly process with Pd^II^ ions towards *cis*‐Pd_2_L_4_ cages with *C*_2h_ symmetry, due to the favourable antiparallel arrangement of *trans*‐oriented ligands (Figure 1b). Accessing Pd_2_L_4_ cage isomers of alternative symmetries represents a significant design challenge. The use of geometric parameters to prepare *C*_2v_ *trans*‐Pd_2_L_4_ cages, for example, would require enforcement of a parallel relationship between *trans*‐oriented ligands, and antiparallel configuration of *cis*‐oriented ligands. Recently, an M_2_L_4_ system with *C*_4v_ symmetry was able to be realised by Crowley and co‐workers, using a sub‐component self‐assembly strategy in the synthesis of a heteronuclear PdPtL_4_ cage.^[23]^

The 1,2‐bis(pyridin‐2‐ylethynyl)benzene motif has previously been used as a *trans*‐chelating unit for square‐planar Pd^II^ ions (Figure 2b),^[24]^ and has been investigated for its potential as a *trans* analogue of Fujita's *cis*‐protection strategy^[25]^ in metal‐organic assembly.^[26]^ It was envisaged that covalently tethering ligand fragments^[27]^ via this unit could be used to constrain the relative orientations of ligands held *trans* to each other. In this manner it might be possible to obtain cages of *C*_2v_ symmetry that have previously proved difficult to access.

### trans‐Pd_2_L_2_ C_2v_ Cage

**L^P^** ^4^ was readily synthesised from 1,2‐diethynylbenzene and commercially available reagents in 2 steps in 73 % overall yield. It was anticipated that two **L^P4^** ligands would assemble in an anti‐parallel arrangement around two Pd^II^ ions. Combining **L^P4^** with Pd(NO_3_)_2_ ⋅ 2H_2_O in *d*_6_‐DMSO at room temperature gradually led to quantitative formation of a single species (**C^P4^**), as observed by ^1^H NMR (Figure 2d). Formation of an assembly with the anticipated [Pd_2_(**L^P4^**)_2_]^4+^ formula was confirmed by electrospray ionisation mass spectrometry (ESI‐MS; Fig S19 and S20). Ultimately, despite the poor quality of the crystals, unambiguous confirmation of the structure was achieved via single crystal X‐ray diffraction (SCXRD) studies of the host–guest complex with an encapsulated chloride anion, [Pd_2_(**L^P4^**)_2_⊃Cl]^3+^ (Figure 2f).^[28]^

Due to the ligand structure and the steric bulk of the diethynylbenzene linker, **C^P4^** exclusively assembles as a Pd_2_L_2_ cage with *C*_2v_ symmetry. Consequently, the cavity of the cage can be described as being surrounded by four iterations of the ligand **L**, reported by Chand, in a (pseudo‐)*trans*‐Pd_2_L_4_ configuration (Figure 1b). As with **L**, efficient formation of **C^P4^** required the presence of a suitable anion template such as NO_3_^−^ or Cl^−^, with BF_4_^−^ alone resulting in a mixture of products (Figure S22). Cl^−^ appeared to be a better guest than NO_3_^−^, evidenced by an increased downfield shift of the endohedral protons H_*a*_ and H_*f*_ (Δδ=0.43 and 0.41 ppm, respectively).

### trans‐Pd_2_L_2_ Pseudo‐heteroleptic C_2_ Cage

Having successfully demonstrated the viability of this tethering strategy to constrain two dipyridyl ligand moieties in a *trans* arrangement, the thought occurred that the two ditopic units need not be identical. Consequently, **L^P3Q^** (Figure 2c) was prepared incorporating one of the previously described dipyridyl fragments, and a second ditopic ligand with one pyridine and one quinoline coordinating unit. Self‐assembly of a stoichiometric mixture of **L^P3Q^** with Pd(NO_3_)_2_ ⋅ 2H_2_O in *d*_6_‐DMSO at 50 °C for 2 h again yielded a single species, determined to be [Pd_2_(**L^P3Q^**)_2_](NO_3_)_4_ (**C^P3Q^**) by NMR and ESI‐MS. Diastereotopic splitting of the signals assigned to the methylene units was observed (Figure 2e), giving two pairs of doublets (5.74/5.51 ppm and 5.42/5.12 ppm), indicative of inequivalent chemical environments on either face of the ditopic ligand fragments. Vapour diffusion of Et_2_O into a solution of the assembly in 1 : 1 DMSO/CH_3_CN yielded X‐ray quality crystals, with SCXRD revealing the anticipated [Pd_2_(**L^P4^**)_2_⊃NO_3_]^3+^ structure (Figure 2g).^[28]^ By virtue of the inequivalence of the two ditopic ligand fragments within **L^P3Q^**, the structural framework of **C^P3Q^** is analogous to that of a heteroleptic Pd_2_L_2_L′_2_ cage^[29]^ assembled from two unsymmetrical ligands (L and L′). Within the assembly, pairs of identical ligands are held *cis* to each other in an anti‐parallel arrangement, giving a structure with *C*_2_ symmetry.

Of note, due to the (bis‐)*trans*‐heterobidentate nature of **L^P3Q^**, each of the Pd^II^ centres in **C^P3Q^** possesses axial chirality.^[30]^ From the SCXRD structure it could be shown that within **C^P3Q^** both ions are of the same stereochemistry, and the system crystallises as a racemic mixture of both (*S*,*S*)‐ and (*R*,*R)‐* **C^P3Q^** enantiomers (see Supporting Information section 6).

This covalent tethering strategy was clearly highly effective at constraining the relative orientations of ligand fragments, and even for the incorporation of more than one framework into an assembly. The *bent*, or *V‐shaped*, nature of **L^P3Q^** gave rise to the *C*_2_ symmetry of **C^P3Q^**. To widen the scope of accessible Pd_2_L_2_L′_2_ cavity symmetries, there was motivation to investigate alternative modes of tethering that might lead to *C*_s_ symmetry structures (Figure 1d). These would be formed from pairs of unsymmetrical ligands with identical ligands arranged *cis* to each other, but now in a parallel orientation. Tethering of ligand fragments in a *linear* fashion appeared to present a solution to accessing these structures.

### cis‐Pd_2_L_4_ Cages from Unsymmetrical Ditopic Ligands

**Figure 3:**
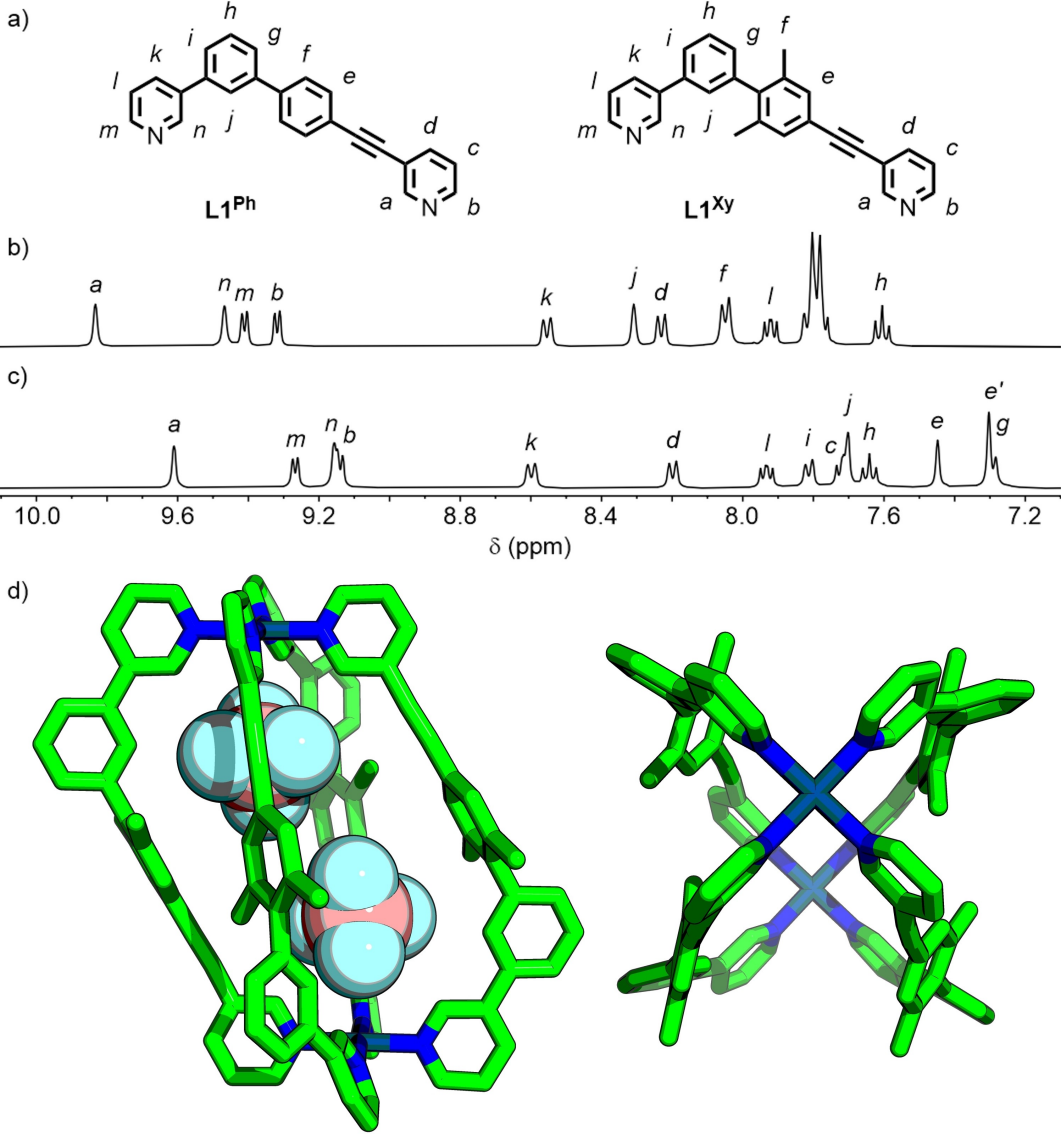
a) Ligands **L1^Ph^** and **L1^Xy^**; partial ^1^H NMR spectra (400 MHz, *d*_6_‐DMSO, 298 K) of b) **C1^Ph^** and c) **C1^Xy^**; d) SCXRD structure of [Pd_2_(**L1^Xy^**)_4_⊃2BF_4_]^2+^.

Based on principles of geometric complementarity delineated in previous work,^[20]^ two low‐symmetry, ditopic ligands were synthesised with isostructural core frameworks (Figure 3a). The ligands differed only in the identity of the flanking aromatic units—1,4‐phenyl (**L1^Ph^**) and 2,5‐*m*‐xylyl (**L1^Xy^**). In each case, self‐assembly with Pd^II^ as the tetrafluoroborate salt in a 2 : 1 ligand/metal ratio resulted in formation of the anticipated *cis*‐[Pd_2_L_4_](BF_4_)_4_ architectures (**C1^Ph^** and **C1^Xy^**), as determined by NMR (Figure 3b and c), ESI‐MS (Figure S75–77 and S100–102) and, for **C1^Xy^**, SCXRD (see below).

The impact of exchanging the flanking phenyl group in **C1^Ph^** for a xylyl unit in **C1^Xy^** resulted in some spectroscopic differences, noticeably a shift in the resonance of the signal assigned to endohedral proton H_*j*_ from 8.31 ppm to 7.71 ppm. The symmetry of the ^1^H NMR spectra of **C1^Ph^** and **C1^Xy^**, through‐space interactions between *ortho*‐pyridyl protons observed by NOESY (H_*a*_⋅⋅⋅H_*n*_, H_*b*_⋅⋅⋅H_*m*_; Figure S73 and S98) and splitting of the H_*e*_ (7.46 and 7.31 ppm, Figure 3c) and H_*f*_ (2.13 and 1.99 ppm) resonances within **C1^Xy^** into two distinct sets of signals, were all congruent with the *C*_2h_ symmetry associated with selective formation of the *cis* assembly (Figure 1b). This was ultimately further confirmed by the solid‐state SCXRD structure of **C1^Xy^** (Figure 3d).^[28]^

### cis‐Pd_3_L_4_ Double‐Cavity Cages

There has been some interest in recent years in the synthesis of linearly‐elongated, polytopic ligand scaffolds, and their self‐assembly to form multi‐cavity cage architectures.^[31]^ In this manner, larger assemblies can be formed that possess several small cavities^[32]^ instead of a single large one.^[33]^ The formation of such a species from low‐symmetry ligands has, to the best of my knowledge, never been reported.^[34]^

**Figure 4:**
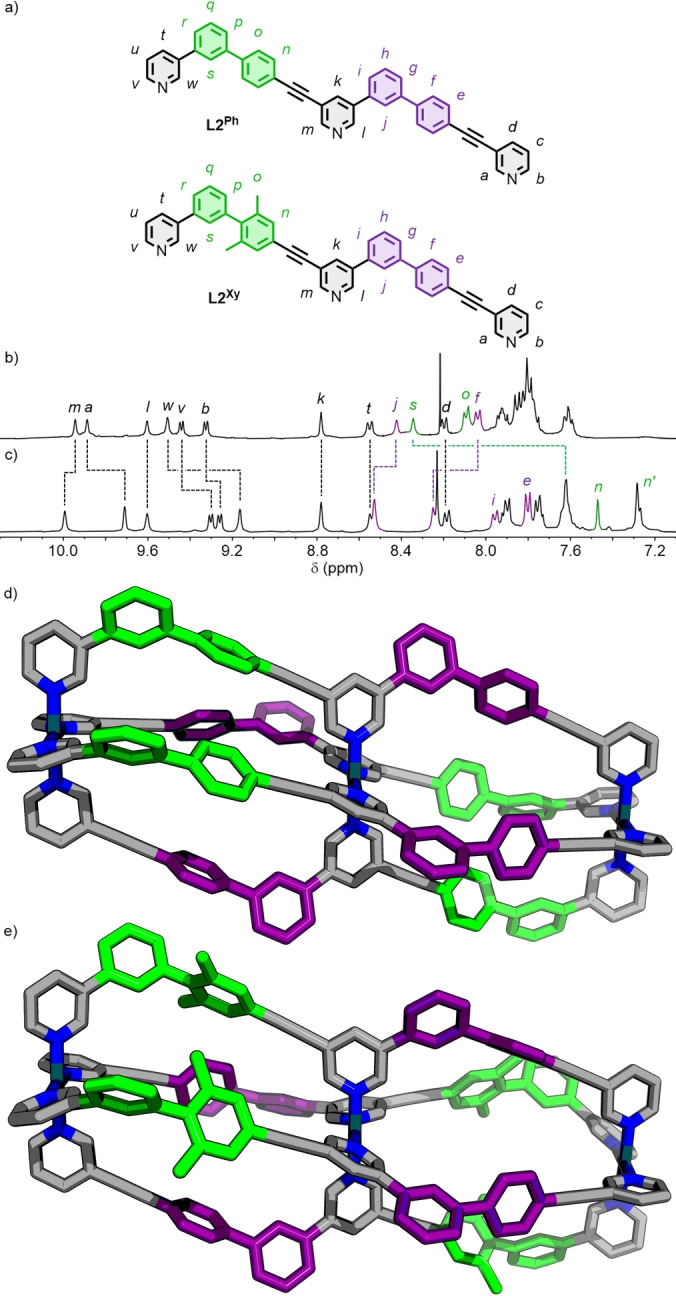
a) Tritopic ligands **L2^Ph^** and **L2^Xy^**; partial ^1^H NMR spectra (400 MHz, 3 : 1 *d*_6_‐DMSO/CDCl_3_, 298 K) of b) **C2^Ph^** and c) **C2^Xy^**; molecular models (PM6) of d) **C2^Ph^** and e) **C2^Xy^** with some chemically inequivalent core ligand fragments shown in green and purple, highlighting the pseudo‐heteroleptic nature of the cavities.

To probe whether the geometric complementarity approach would remain effective for the assembly of low‐symmetry, multi‐cavity systems, tritopic ligand **L2^Ph^** (Figure 4a) was synthesised, incorporating two linearly‐fused iterations of the **L1^Ph^** scaffold. Pleasingly, self‐assembly with [Pd(CH_3_CN)_4_](BF_4_)_2_ in a 3 : 4 metal/ligand ratio in 3 : 1 *d*_6_‐DMSO/CDCl_3_ successfully yielded the desired *cis*‐Pd_3_L_4_ double‐cavity cage (**C2^Ph^**), determined by NMR (Figure 4b; see below) and ESI‐MS (Figure S151–153).

As with previous work on the self‐assembly of unsymmetrical ditopic ligands,^[20]^ the symmetry of the ^1^H NMR spectrum and through‐space interactions observed by NOESY between opposite ends of the ligand framework (H_*a*_⋅⋅⋅H_*w*_, H_*l*_⋅⋅⋅H_*m*_; Figure S149) within the trinuclear architecture were consistent with cages of either *C*_2v_ (*trans*) or *C*_2h_ (*cis*) symmetry. Disappointingly, the growth of X‐ray quality crystals proved elusive. Based, however, on previous work, the dinuclear cages **C1^Ph^** and **C1^Xy^**, and molecular modelling of the *cis*‐Pd_3_L_4_ structure (Figure 4d), it was concluded that **C2^Ph^** must be the *cis* assembly. As such, the efficacy of using geometric design parameters to enforce ligands *trans* to each other to arrange in an anti‐parallel fashion is retained by the elongated, tritopic ligand.

Although the two cavities of the double‐decker assembly **C2^Ph^** are chemically equivalent, separate sets of signals in the ^1^H NMR spectrum were observed for the protons associated with each half of the ligand framework (Figure 4b), confirming the difference in their chemical environments. This can readily be seen by the different chemical shifts in the ^1^H NMR spectrum of the homologous *ortho*‐pyridyl protons H_*a*_ and H_*m*_ (δ=9.89 and 9.94 ppm, respectively) and H_*l*_ and H_*w*_ (δ=9.60 and 9.51 ppm, respectively), and endohedral protons H_*j*_ and H_*s*_ (δ=8.42 and 8.34 ppm, respectively).

The consequence of the combination of chemically inequivalent fragments of **L2^Ph^** and the symmetry of **C2^Ph^** is that each of the identical cage cavities are encompassed by two chemically distinct, unsymmetrical ligand fragments (Figure 4d). As a result, the cavities can be visualised as (pseudo‐)heteroleptic Pd_2_L_2_L′_2_ environments of *C_s_* symmetry.

This concept was further demonstrated through the self‐assembly of ligand **L2^Xy^** (Figure 4a) with [Pd(CH_3_CN)_4_](BF_4_)_2_, under the same conditions as **L2^Ph^**, to give **C2^Xy^**, in which one half of the ligand scaffold possesses a phenyl ring as the flanking aromatic unit, the other a xylyl moiety. As with the dinuclear **C1** cages and **C2^Ph^**, confirmation of successfully forming the targeted *cis*‐Pd_3_L_4_ isomer came from ESI‐MS (Figure S168–170), the high symmetry of the NMR spectrum (Figure 4c), NOE interactions only feasible between ligands in an anti‐parallel arrangement (H_*a*_⋅⋅⋅H_*w*_, H_*l*_⋅⋅⋅H_*m*_; Figure S165) and, consistent with **C1^Xy^**, splitting of the aromatic (H_*n*_; δ=7.47 and 7.28 ppm) and methyl (H_*o*_; δ=2.03 and 1.92 ppm) protons of the xylyl unit in **C2^Xy^** into two distinct signals.

Within the trinuclear **C2^Xy^**, two pairs of ligands surround each individual cage cavity (Figure 4e)—one with phenyl units as the flanking aromatic moieties (as per **C1^Ph^**), the other with *m*‐xylyl (as per **C1^Xy^**), the close proximity of which was demonstrated by NOE interactions observed between the two (H_*e*_⋅⋅⋅H_*o*_; Figure S165 and S166). Again, the difference in the ligand environments was readily shown by ^1^H NMR (Figure 4c) in the chemical shifts of homologous endohedral protons (e.g. H_*j*_ and H_*s*_—8.53 and 7.62 ppm, respectively).

The geometric parameters that drive selective formation of the *cis*‐[Pd_3_(**L2**)_4_]^6+^ cage structures mean that ligands positioned *trans* from each other across the Pd^II^ ions are in an anti‐parallel relative orientation. The result is that each cavity is described by two chemically distinct, unsymmetrical ligand environments (Figure 4d and e). Within a single, dinuclear cage structure, such an assembly would be termed heteroleptic. As the *cis*‐[Pd_3_(**L2**)_4_]^6+^ assemblies are homoleptic as a whole, and it is the individual cavities that possess an *induced* heteroleptic environment, the term *pseudo‐heteroleptic* is suggested to describe the cavity environments that result from the self‐assembly of the low‐symmetry ligands. This term would also seem appropriate for the **C^P3Q^** architecture described above, in which two distinct, unsymmetrical ligand fragments were incorporated into a single cage structure in a configurationally defined manner. As such, these pseudo‐heterolepticity approaches offer the potential for incorporating multiple functionalities into the cavity environment of the cage that could not otherwise be achieved via integrative self‐sorting of multiple ligands, and under facile self‐assembly conditions afforded by the formally homoleptic architectures.

## Conclusion

With general principles of metal‐organic self‐assembly well evolved, and growing interest in the various applications of metal‐organic cages, the development of strategies to access more structurally sophisticated systems would greatly advance their utility. Constructing MOCs with more than one ligand framework offers the potential to precision engineer both the shape and functionality of the cavity environment. Previous approaches to the assembly of heteroleptic metal‐organic architectures rely on integrative self‐assembly of multiple ligands, either resulting from geometric complementarity, or coordination sphere engineering strategies. Whilst these have proven effective to a certain extent, there are inherent limitations to these approaches.

In this work, strategies have been outlined towards the development of *pseudo*‐heteroleptic MOCs—cages that, although they are assembled from single ligands, possess cavity spaces described by multiple ligand environments. These rely on covalent tethering of ligand fragments to constrain their relative orientations. By controlling the geometry of the tethering—either *bent* or *linearly fused*—MOCs have been realised that incorporate two inequivalent, unsymmetrical ligand scaffolds in a defined manner, generating cavities of *C*_2_ and *C*_s_ symmetry, respectively—a feat that has so far not been achieved via integrative self‐assembly of ligand mixtures.

The delicate balance of entropic and enthalpic factors can be easily disturbed to shift thermodynamic minima away from desired products. Removing the reliance on integrative self‐sorting greatly simplifies the self‐assembly process, whilst enhancing the structural and functional complexity of the cavity space. As such, pseudo‐heteroleptic design strategies will allow fine‐tuning of the cavity shape and functionality of discrete, porous MOCs, under facile conditions, to an extent not previously possible.

## Conflict of interest

The authors declare no conflict of interest.

1

## Supporting information

As a service to our authors and readers, this journal provides supporting information supplied by the authors. Such materials are peer reviewed and may be re‐organized for online delivery, but are not copy‐edited or typeset. Technical support issues arising from supporting information (other than missing files) should be addressed to the authors.

Supporting Information

Supporting Information

## Data Availability

The data that support the findings of this study are available from the corresponding author upon reasonable request.
