# Supplementary material for: Pseudo‐heterolepticity in Low‐Symmetry Metal‐Organic Cages
Source: Angew Chem Int Ed Engl. 2022 Sep 29;61(44):e202212392. doi: 10.1002/anie.202212392 (PMC9828238; doi:10.1002/anie.202212392)
Supplement: Supplementary file 2 — Supporting Information [file ANIE-61-0-s001.pdf]

Angewandte Chemie
Eine Zeitschrift der Gesellschaft Deutscher Chemiker

Supporting Information

Pseudo-heterolepticity in Low-Symmetry Metal-Organic Cages

*J. E. M. Lewis**

Contents

1. General Experimental 4

2. Synthetic Procedures 5

Synthesis of **S1** 6

Synthesis of **S2** 9

Synthesis of $\mathbf{L^{P4}}$ 10

Synthesis of $[Pd_2(\mathbf{L^{P4}})_2](NO_3)_4$ ($\mathbf{C^{P4}}$) 13

Synthesis of $[Pd_2(\mathbf{L^{P4}})_2\supset Cl](BF_4)_3$ 17

Synthesis of **S3** 19

Synthesis of **S4** 22

Synthesis of **S5** 25

Synthesis of **S6** 28

Synthesis of $\mathbf{L^{P3Q}}$ 31

Synthesis of $[Pd_2(\mathbf{L^{P3Q}})_2\supset NO_3](NO_3)_3$ ($\mathbf{C^{P3Q}}$) 34

Synthesis of **S7** 40

Synthesis of $\mathbf{L1^{Ph}}$ 43

Synthesis of *cis*-$[Pd_2(\mathbf{L1^{Ph}})_4](BF_4)_4$ ($\mathbf{C1^{Ph}}$) 46

Synthesis of **S8** 51

Synthesis of **S9** 54

Synthesis of $\mathbf{L1^{Xy}}$ 57

Synthesis of *cis*-$[Pd_2(\mathbf{L1^{Xy}})_4](BF_4)_4$ ($\mathbf{C1^{Xy}}$) 60

Synthesis of **S10** 65

Synthesis of **S11** 68

Synthesis of **S12** 71

Synthesis of **S13** 74

Synthesis of **S14** 77

Synthesis of $\mathbf{L1^{TIPS}}$ 80

Synthesis of $\mathbf{L1^{CCH}}$ 83

Synthesis of $\mathbf{L2^{Ph}}$ 86

Synthesis of *cis*-$[Pd_3(\mathbf{L2^{Ph}})_4](BF_4)_6$ ($\mathbf{C2^{Ph}}$) 90

Synthesis of $\mathbf{L2^{Xy}}$ 96

Synthesis of *cis*-$[Pd_3(\mathbf{L2^{Xy}})_4](BF_4)_6$ ($\mathbf{C2^{Xy}}$) 100

3. Geometry Optimised Structures 106

$C2^{Ph}$ 106

$C2^{Xy}$ 113

4. Hydrodynamic Radii Calculations 121

5. X-ray Crystallography 122
cis-[Pd_2(**L1**^{Xy})$_4$](BF_4)$_4$ 122
[Pd_2(**L**^{P4})$_2$⊃Cl](BF_4)$_3$ 124
[Pd_2(**L**^{P3Q})$_2$⊃NO_3](NO_3)$_3$ 126
6. Chirality Assignment in **C**^{P3Q} 127
7. References 128

1. General Experimental

Synthesis: Unless otherwise stated, all reagents, including anhydrous solvents, were purchased from commercial sources and used without further purification. All reactions were carried out under an atmosphere of N_2 using degassed, anhydrous solvents unless otherwise stated. Petrol refers to the fraction of petroleum ether boiling in the range 40-60 °C. Analytical TLC was performed on pre-coated silica gel plates (0.25 mm thick, 60F254, Merck, Germany) and observed under UV light. EDTA solution refers to a 0.1 M solution of EDTA-Na_2 in 3% $NH_{3(aq)}$.

Analysis: NMR spectra were recorded on Bruker AV400 or AV500 instrument, at a constant temperature of 298 K. Chemical shifts are reported in parts per million from low to high field and referenced to residual solvent. Standard abbreviations indicating multiplicity were used as follows: m = multiplet, quint = quintet, q = quartet, t = triplet, d = doublet, s = singlet, app. = apparent, br. = broad. Chemical shifts are reported in parts per million (ppm) and referenced to residual solvent peaks ($CDCl_3$: ^{1}H δ = 7.26 ppm, ^{13}C δ = 77.16 ppm; d_6-DMSO: ^{1}H δ = 2.50 ppm. ^{13}C δ = 39.52 ppm; CD_3OD: ^{1H} δ = 3.31 ppm, ^{13}C δ = 49.00 ppm). For **C2^{Ph}** and **C2^{Xy}** in d_6-DMSO/$CDCl_3$ mixtures, spectra were referenced to d_6-DMSO residual solvent peaks. Signal assignment was carried out using 2D NMR methods (HSQC, HMBC, COSY, NOESY) where necessary. In the case of some signals absolute assignment was not possible. Here indicative either/or assignments (e.g. H_A/H_B for H_A or H_B) are provided. Mass spectrometry was carried out by the Imperial College London, Department of Chemistry Mass Spectroscopy Service using Waters LCT Premier for HR-ESI-MS and Thermo Scientific Q-Exactive.

2. Synthetic Procedures

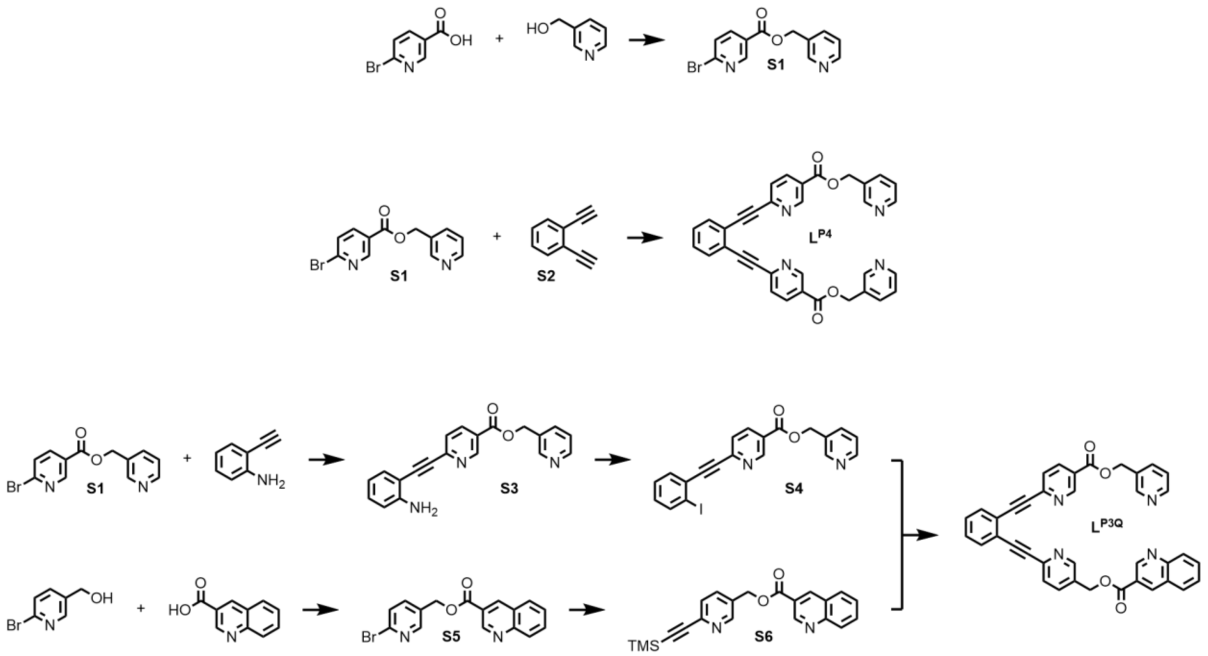

Scheme S1 Synthetic route to ligands $\mathbf{L^{P4}}$ and $\mathbf{L^{P3Q}}$.

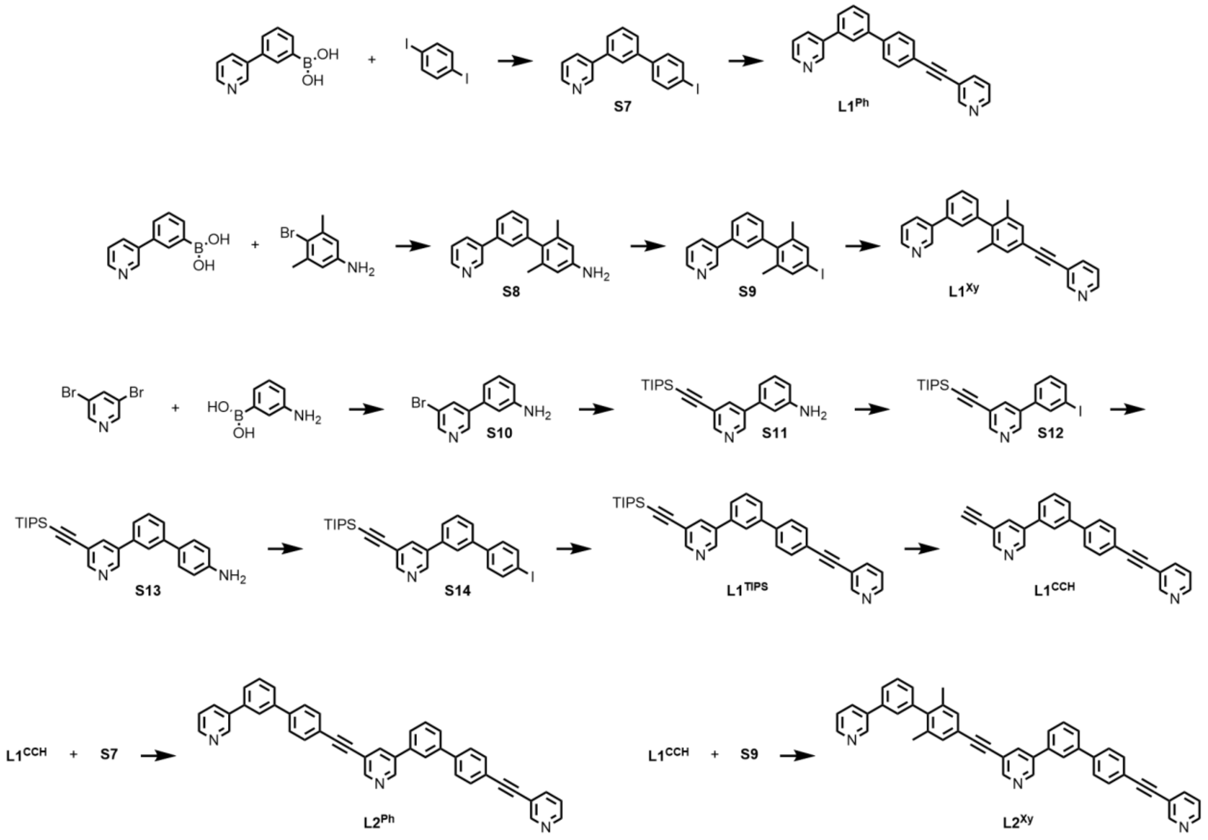

Scheme S2 Synthetic route to ligands $\mathbf{L1^{Ph}}$, $\mathbf{L1^{Xy}}$, $\mathbf{L2^{Ph}}$, and $\mathbf{L2^{Xy}}$.

Synthesis of S1

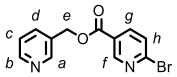

6-Bromonicotinic acid (1.01 g, 5 mmol, 1 eq.) was added portionwise to a stirring solution of EDCI·HCl (1.15 g, 6 mmol, 1.2 eq.) and DMAP (0.061 g, 0.5 mmol, 0.1 eq.) in $CHCl_3$ (25 mL) at 0 °C. After 30 minutes 3-pyridinemethanol (0.53 mL, 5.5 mmol, 1.1 eq.) was added dropwise via syringe and the reaction allowed to warm to rt. After 3 d the reaction mixture was washed with sat. aq. $NaHCO_3$ (25 mL), brine (25 mL), dried ($MgSO_4$) and the solvent removed *in vacuo*. After purification by column chromatography on silica gel (1:4 acetone/CH_2Cl_2) the product was obtained as a white solid (1.46 g, 99%).

^{1}H NMR (400 MHz, $CDCl_3$) δ: 8.97 (d, *J* = 2.4 Hz, 1H, H_f), 8.71 (s, 1H, H_a), 8.62 (dd, *J* = 4.9, 1.6 Hz, 1H, H_b), 8.12 (dd, *J* = 8.4, 2.4 Hz, 1H H_g), 7.79 (app. dt, *J* = 7.9, 1.9 Hz, 1H, H_d), 7.58 (d, *J* = 8.3 Hz, 1H, H_h), 7.35 (dd, *J* = 7.9, 4.9 Hz, 1H, H_c), 5.40 (s, 2H, H_e).

^{13}C NMR (101 MHz, $CDCl_3$) δ: 164.4, 151.6 (C_f), 150.0 (C_b), 149.8 (C_a), 147.4, 139.3 (C_g), 136.6 (C_d), 131.1, 128.3 (C_h), 125.0, 123.8 (C_c), 64.9 (C_e).

HR-ESI-MS m/z = 292.9936 $[M+H]^+$ calc. 292.9926.

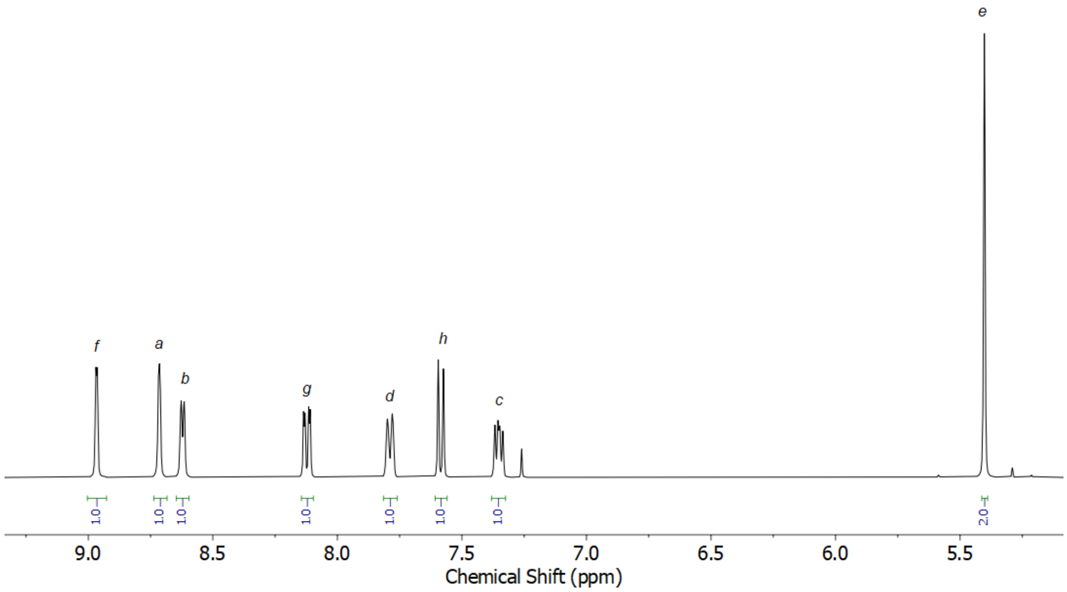

Figure S1 ^{1}H NMR (400 MHz, $CDCl_3$) of **S1**.

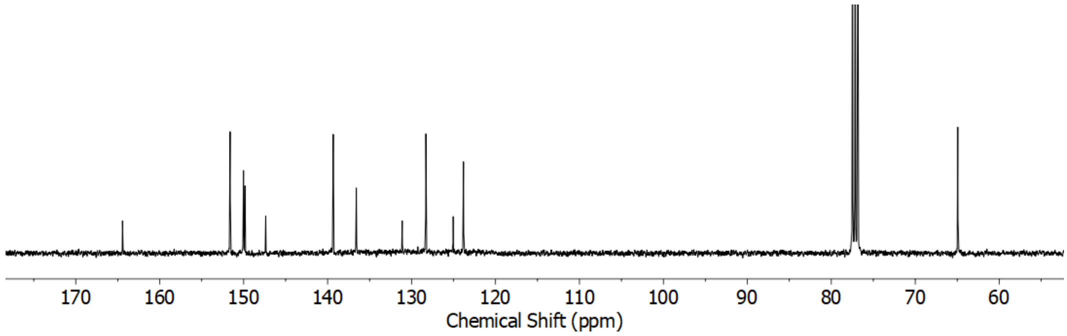

Figure S2 ^{13}C NMR (101 MHz, $CDCl_3$) of **S1**.

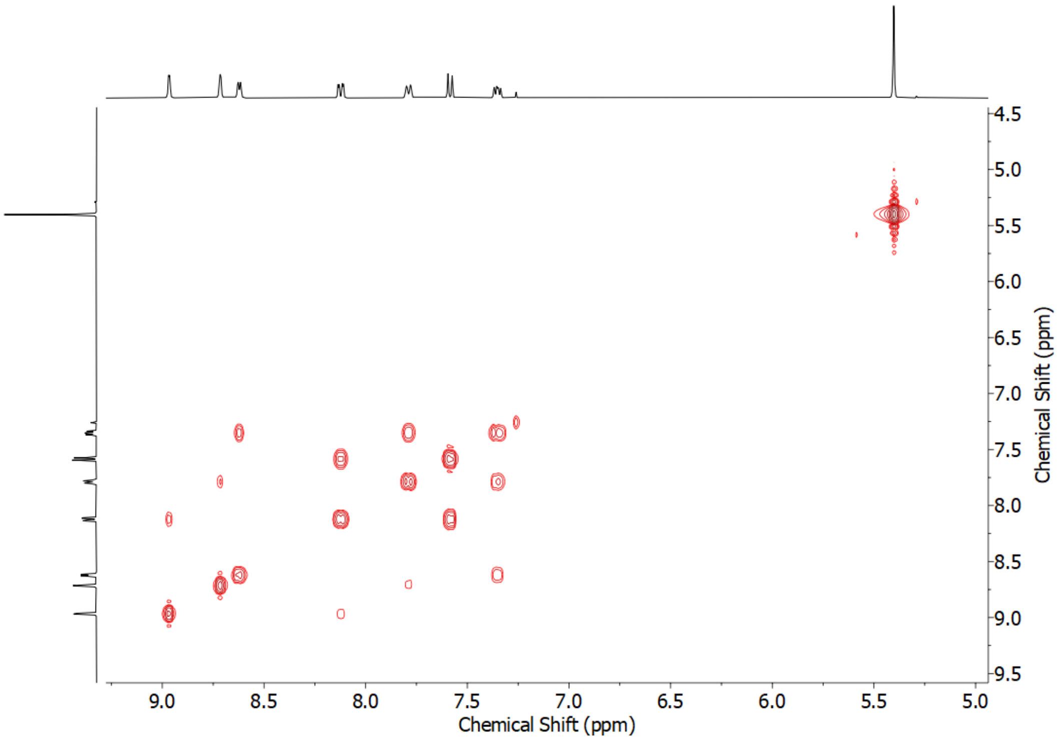

Figure S3 COSY NMR ($CDCl_3$) of **S1**.

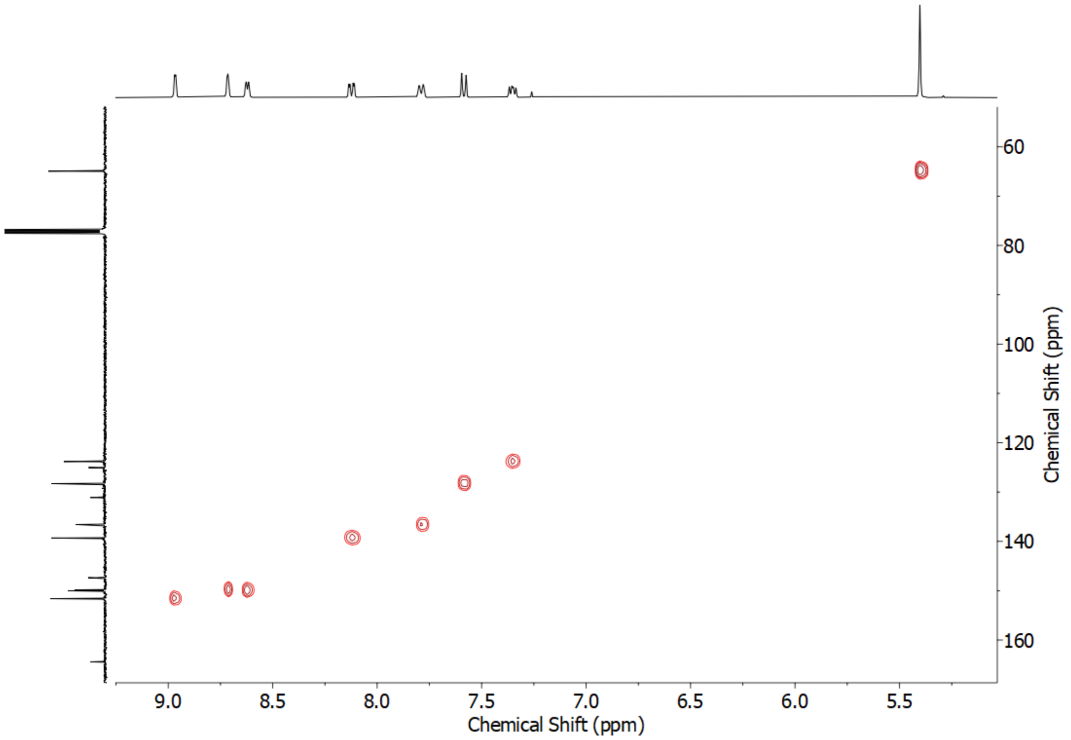

Figure S4 HSQC NMR ($CDCl_3$) of **S1**.

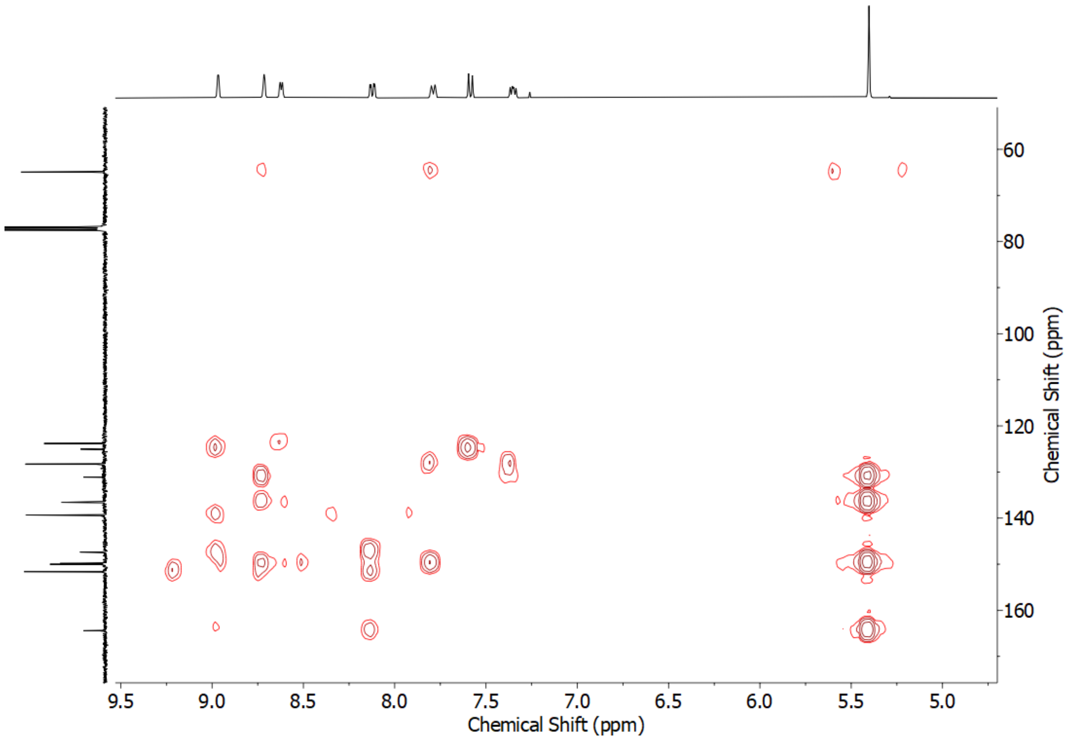

Figure S5 HMBC NMR ($CDCl_3$) of **S1**.

Synthesis of S2

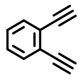

To a stirring solution of 1,2-diiodobenzene (0.990 g, 3 mmol, 1 eq.), $Pd(PPh_3)_2Cl_2$ (0.042 g, 0.06 mmol, 2 mol%) and CuI (0.011 g, 0.06 mmol, 2 mol%) in $^iPr_2NH$ (30 mL) was added trimethylsilylacetylene (1.25 mL, 9 mmol, 3 eq.) via syringe and the reaction mixture stirred at rt for 24 h. H_2O (25 mL) was added and the aqueous phase extracted with CH_2Cl_2 (3 × 25 mL). The combined organic phases were dried ($MgSO_4$) and the solvent removed *in vacuo*. The residue was filtered through a plug of silica gel (pentane) to give 1,2-bis(trimethylsilylethynyl)benzene as a yellow oil. This was dissolved in 1:1 CH_2Cl_2/MeOH (50 mL) under air and K_2CO_3 (2.07 g, 15 mmol, 5 eq.) added as a solid. After stirring at rt for 2 h the mixture was filtered through celite and the solvent removed *in vacuo*. After filtration through a plug of silica gel (pentane) the product was obtained as a yellow oil (0.291 g, 77% over 2 steps). Spectroscopic data matched literature values.[1]

^{1}H NMR (400 MHz, $CDCl_3$) δ: 7.52 (m, 2H), 7.32 (m, 2H), 3.34 (s, 2H).

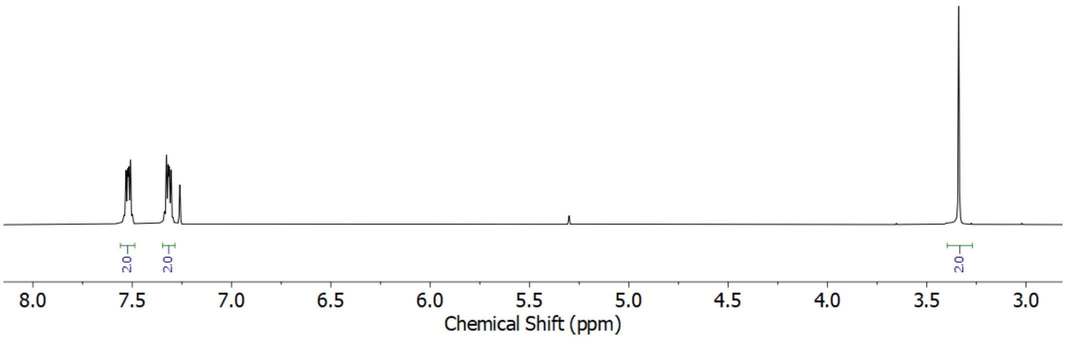

Figure S6 ^{1}H NMR (400 MHz, $CDCl_3$) of **S2**.

Synthesis of L^{P4}

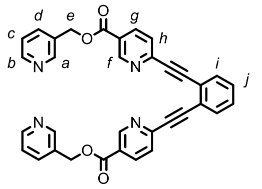

$^{i}Pr_2NH$ (1 mL) was added to a solution of **S2** (0.063 g, 0.5 mmol, 1 eq.), **S1** (0.308 g, 1.05 mmol, 2.1 eq.), $Pd(PPh_3)_4$ (0.014 g, 0.013 mmol, 2.5 mol%) and CuI (0.0024 g, 0.013 mmol, 2.5 mol%) in MeCN (4 mL) at rt and the reaction mixture stirred for 18 h. EDTA solution (10 mL) was added and the aqueous phase extracted with CH_2Cl_2 (3 × 10 mL). The combined organic phases were dried ($MgSO_4$) and the solvent removed *in vacuo*. After purification by column chromatography on silica gel (step gradient 0:10 to 5:5 acetone/CH_2Cl_2 in 10% increments) the product was obtained as a beige solid (0.203 g, 74%).

^{1}H NMR (400 MHz, $CDCl_3$) δ: 9.24 (m, 2H, H_f), 8.75 (s, 2H, H_a), 8.63 (m, 2H, H_b), 8.29 (dd, *J* = 8.2, 2.2 Hz, 2H, H_g), 7.83-7.81 (m, 4H, H_d, H_h), 7.67 (dd, *J* = 5.8, 3.3 Hz, 2H, H_i), 7.42 (dd, *J* = 5.9, 3.3 Hz, 2H, H_j), 7.37 (dd, *J* = 7.9, 4.9 Hz, 2H, H_c), 5.43 (s, 4H, H_e).

^{13}C NMR (101 MHz, $CDCl_3$) δ: 164.7, 151.4 (C_f), 150.0 (C_b), 149.9 (C_a), 147.3, 137.4 (C_g), 136.6 (C_d/C_h), 132.6 (C_i), 131.3, 129.6 (C_j), 127.3 (C_d/C_h), 125.2, 124.4, 123.8 (C_c), 92.9, 90.8, 64.8 (C_e).

HR-ESI-MS m/z = 551.1733 $[M+H]^+$ calc. 551.1719.

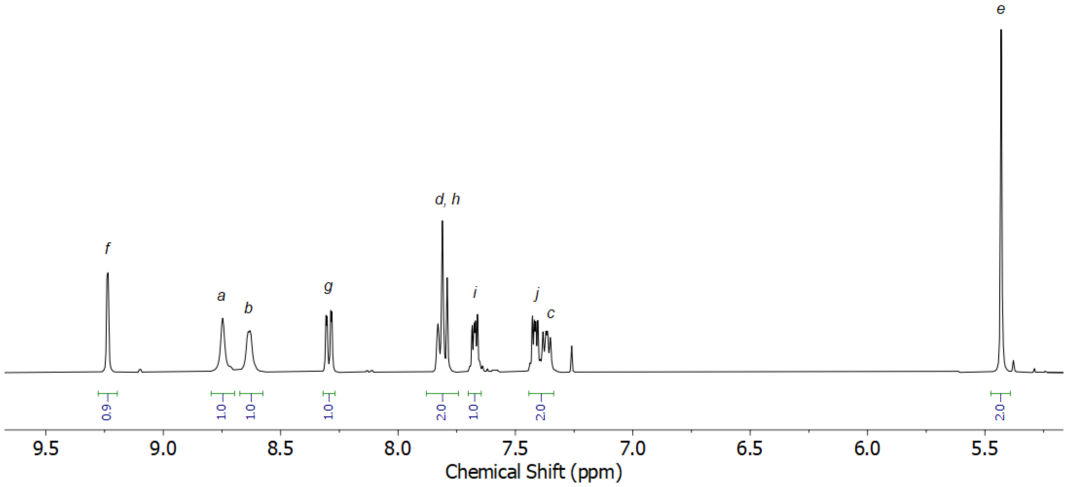

Figure S7 ^{1}H NMR (400 MHz, $CDCl_3$) of $\mathbf{L^{P4}}$.

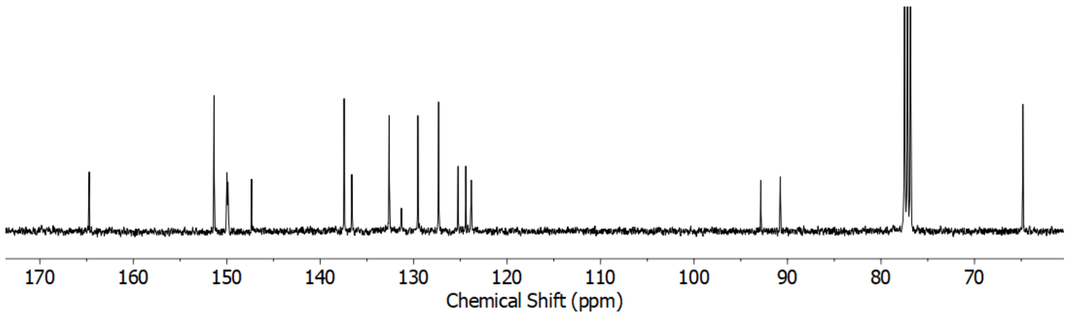

Figure S8 ^{13}C NMR (101 MHz, $CDCl_3$) of $\mathbf{L^{P4}}$.

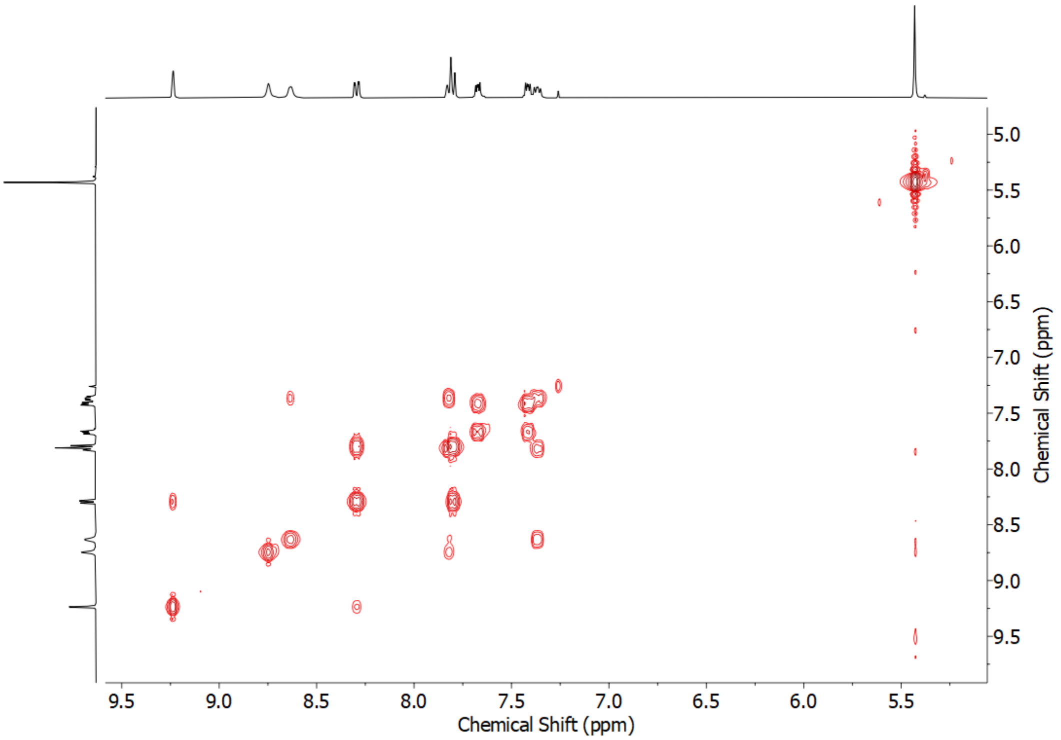

Figure S9 COSY NMR ($CDCl_3$) of **L**$^{\mathbf{P4}}$.

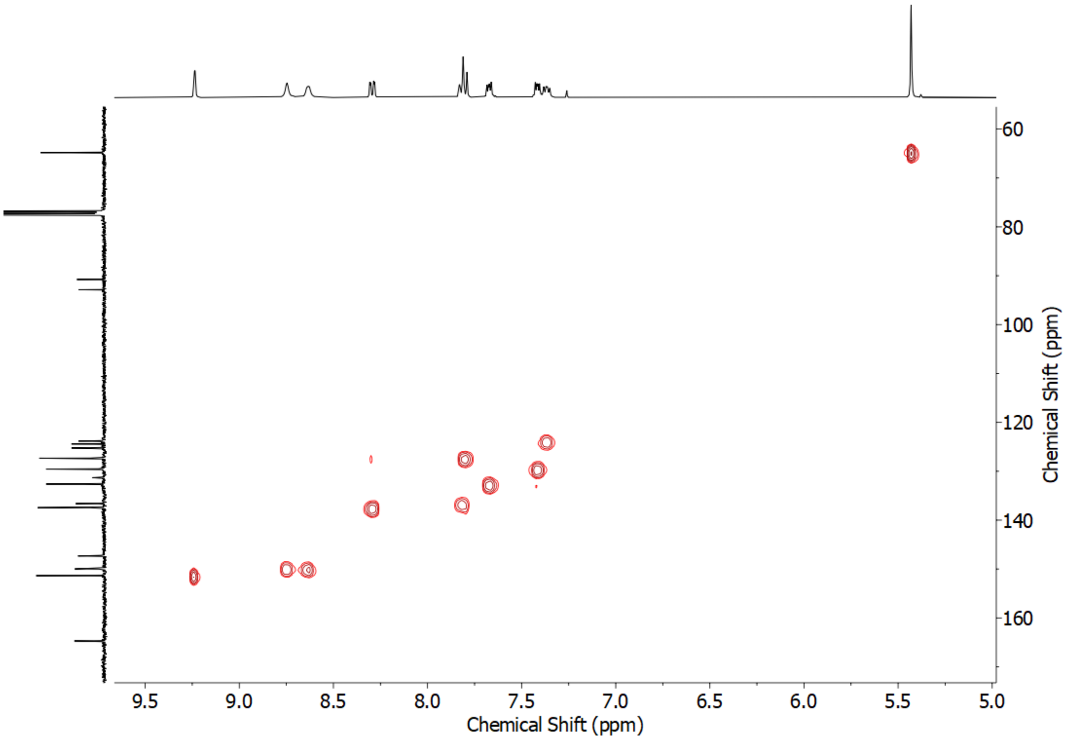

Figure S10 HSQC NMR ($CDCl_3$) of **L**$^{\mathbf{P4}}$.

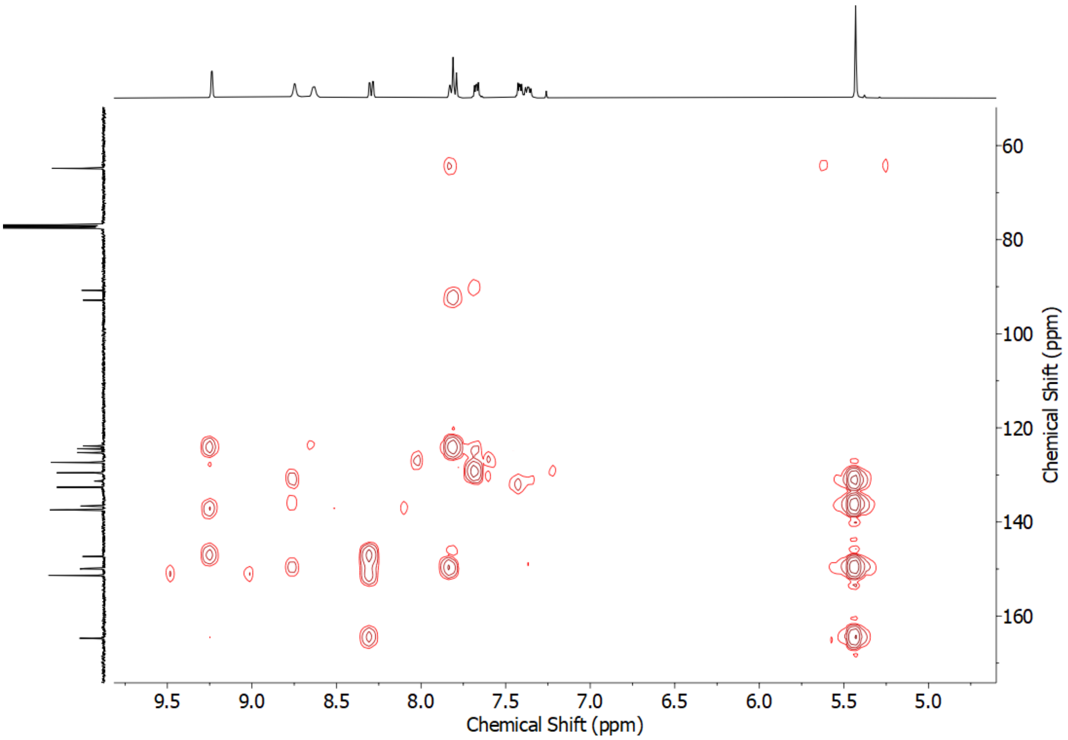

Figure S11 HMBC NMR ($CDCl_3$) of **L**[**P4**].

Synthesis of $[Pd_2(L^{P4})_2](NO_3)_4$ (C^{P4})

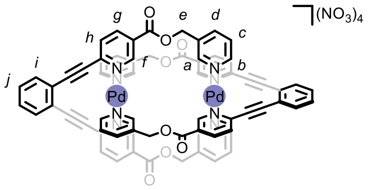

L^P4 (11.0 mg, 20 µmol, 1 eq.) and $Pd(NO_3)_2{\cdot}2H_2O$ (5.3 mg, 20 µmol, 1 eq.) were sonicated in d_6-DMSO (0.75 mL) until a homogenous solution was obtained. After standing at rt for less than a day, quantitative conversion to $[Pd_2($**L**$^{P4})_2](NO_3)_4$ was observed by ^{1}H NMR.

^{1}H NMR (500 MHz, d_6-DMSO) δ: 10.90 (dd, J = 1.9, 0.7 Hz, 4H, H_f), 9.82 (app. dt, J = 1.7, 0.8 Hz, 4H, H_a), 9.07 (ddd, J = 5.8, 1.4, 0.7 Hz, 4H, H_b), 8.76 (dd, J = 8.3, 1.9 Hz, 4H, H_g), 8.35 (dd, J = 8.2, 0.6 Hz, 4H, H_h), 8.26 (dd, J = 5.7, 3.3 Hz, 4H, H_i/H_j), 8.11 (ddd, J = 7.9, 2.1, 1.2 Hz, 4H, H_d), 7.98 (dd, J = 5.7, 3.4 Hz, 4H, H_i/H_j), 7.70 (m, 4H, H_c), 5.49 (s, 8H, H_e).

Diffusion coefficient (500 MHz, d_6-DMSO) D: 1.16 × 10^{-10} m^2s^{-1}; R_H: 8.64 Å.

^{13}C NMR (126 MHz, d_6-DMSO) δ: 162.2, 154.4 (C_f), 150.9 (C_b), 149.3 (C_a), 145.7, 142.7 (C_g), 139.9 (C_d), 135.4, 133.9 (C_i/C_j), 132.5 (C_i/C_j), 132.0 (C_h), 128.2, 127.1 (C_c), 123.3, 99.1, 89.5, 65.2 (C_e).

ESI-MS m/z = 458.75 $\{[Pd_2($**L**$^{P4})_2](NO_3)\}^{3+}$ calc. 458.71.

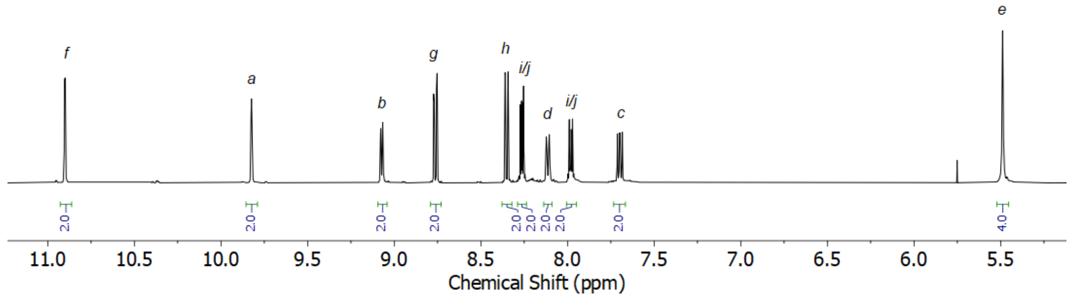

Figure S12 ^{1}H NMR (500 MHz, d_6-DMSO) of $[Pd_2($**L**$^{P4})_2](NO_3)_4$.

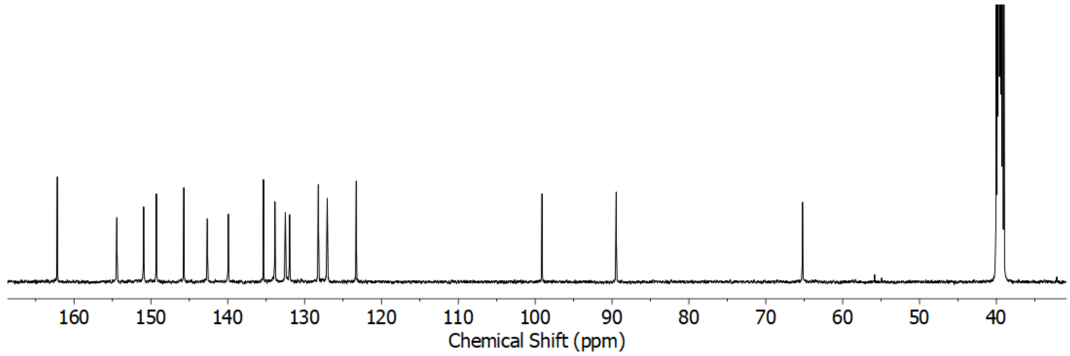

Figure S13 ^{13}C NMR (126 MHz, d_6-DMSO) of $[Pd_2($**L**$^{P4})_2](NO_3)_4$.

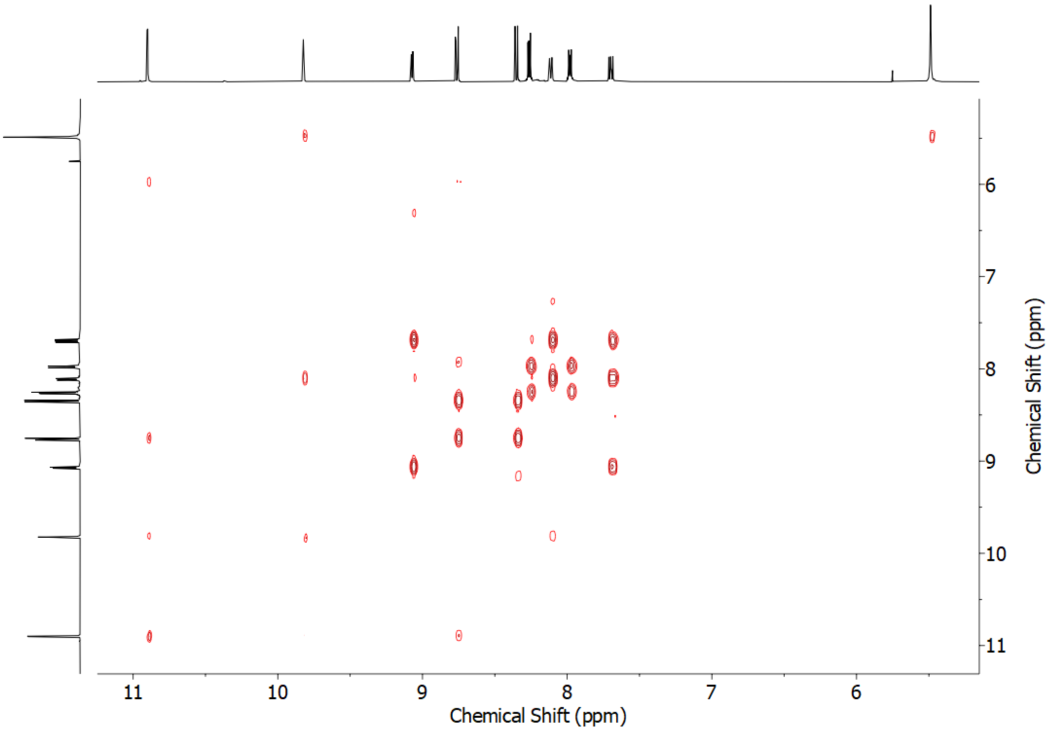

Figure S14 COSY NMR (d_6-DMSO) of $[Pd_2(\mathbf{L^{P4}})_2](NO_3)_4$.

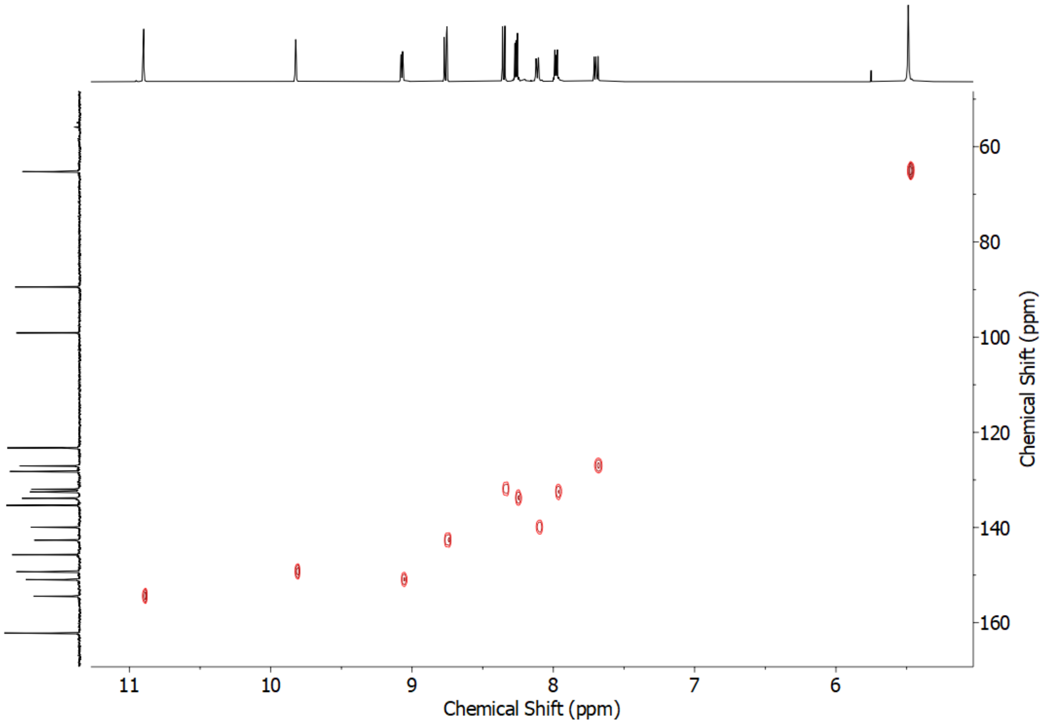

Figure S15 HSQC NMR (d_6-DMSO) of $[Pd_2(\mathbf{L^{P4}})_2](NO_3)_4$.

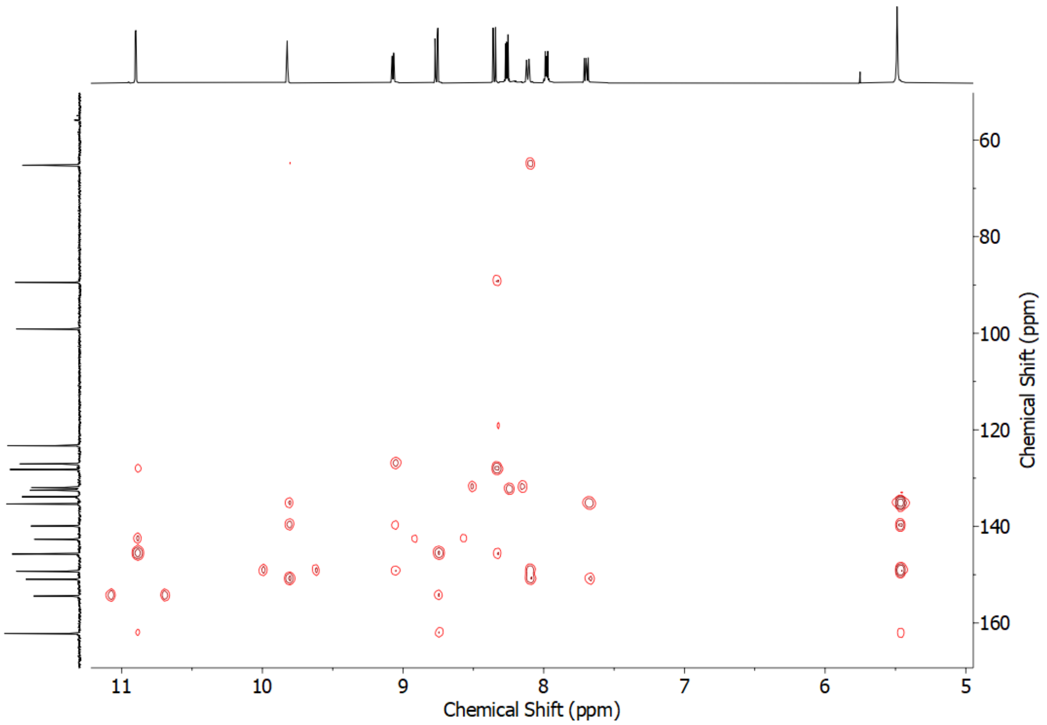

Figure S16 HMBC NMR (d_6-DMSO) of $[Pd_2(\mathbf{L^{P4}})_2](NO_3)_4$.

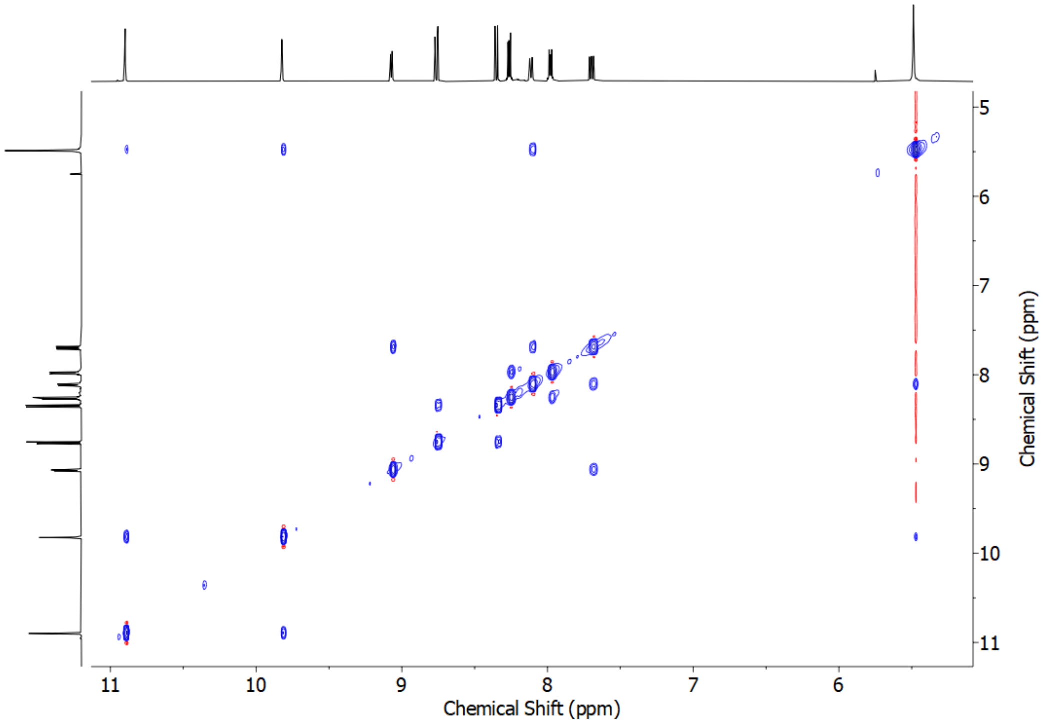

Figure S17 NOESY (d_6-DMSO) of $[Pd_2(\mathbf{L^{P4}})_2](NO_3)_4$.

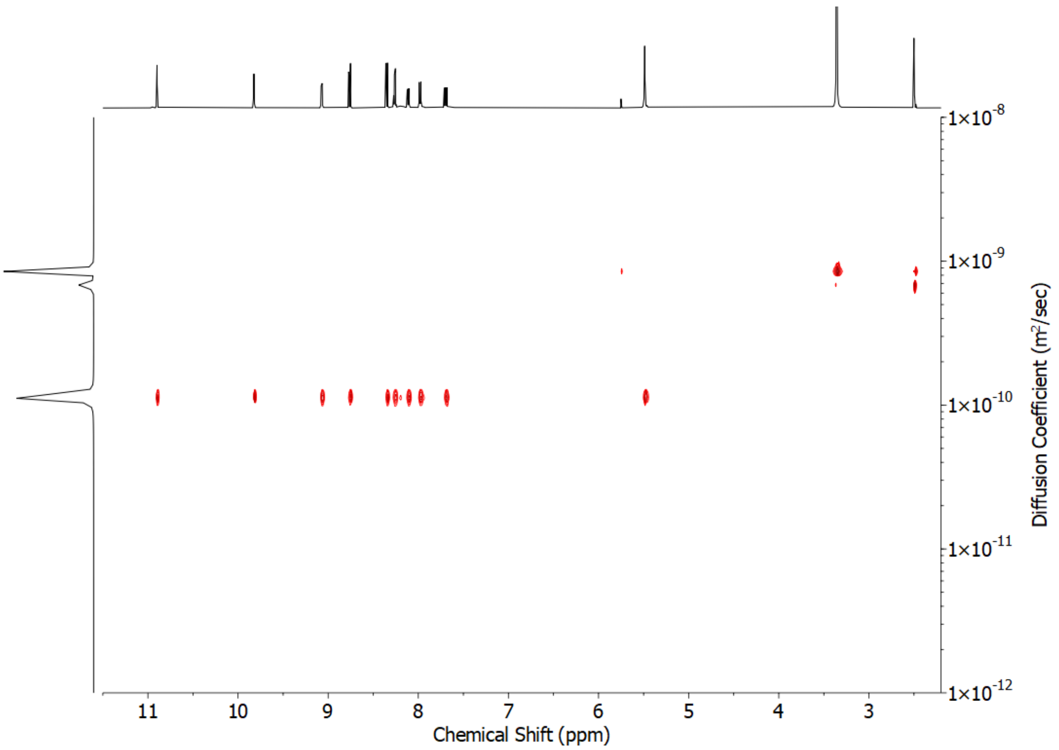

Figure S18 DOSY (d_6-DMSO) of $[Pd_2(\mathbf{L^{P4}})_2](NO_3)_4$.

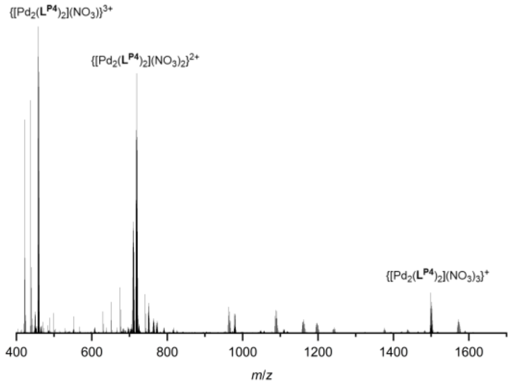

Figure S19 ESI-MS of $[Pd_2(\mathbf{L^{P4}})_2](NO_3)_4$.

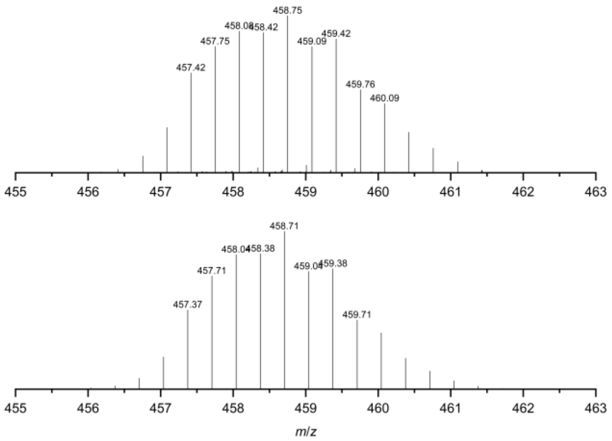

Figure S20 Observed (top) and calculated (bottom) isotopic patterns for $\{[Pd_2(\mathbf{L^{P4}})_2](NO_3)\}^{3+}$.

Synthesis of $[Pd_2(L^{P4})_2⊃Cl](BF_4)_3$

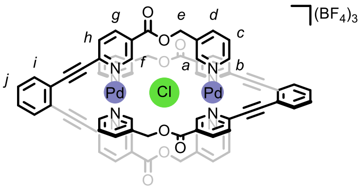

L^{P4} (11.0 mg, 20 µmol), $[Pd(CH_3CN)_4](BF_4)_2$ (8.9 mg, 20 µmol) and Bu_4NCl (2.8 mg, 10 µmol) were sonicated in d_6-DMSO (0.75 mL) until a homogenous solution was obtained. After standing at rt for 2 d the solution was diluted with DMF (0.75 mL), filtered through celite and left for vapour diffusion of Et_2O. After 5 d the mother liquor was decanted off and the yellow precipitate washed with Et_2O before drying in air. This was dissolved in DMF, filtered through celite and left for vapour diffusion of Et_2O, resulting in light yellow, X-ray quality crystals. After 11 d, the mother liquor was decanted off and the precipitate washed with Et_2O before drying in air to give the product as a yellow solid (9.5 mg, 59%).

^{1}H NMR (400 MHz, d_6-DMSO) δ: 11.30 (d, J = 1.9 Hz, 2H, H_f), 10.23 (s, 2H, H_a), 9.25 (d, J = 5.7 Hz, 2H, H_b), 8.75 (dd, J = 8.3, 1.9 Hz, 2H, H_g), 8.34 (d, J = 8.3 Hz, 2H, H_h), 8.27 (dd, J = 5.7, 3.3 Hz, 2H, H_i/H_j), 8.07 (dt, J = 8.0, 1.6 Hz, 2H, H_d), 7.99 (dd, J = 5.7, 3.4 Hz, 2H, H_i/H_j), 7.75 (dd, J = 8.0, 5.8 Hz, 2H, H_c), 5.46 (s, 4H, H_e).

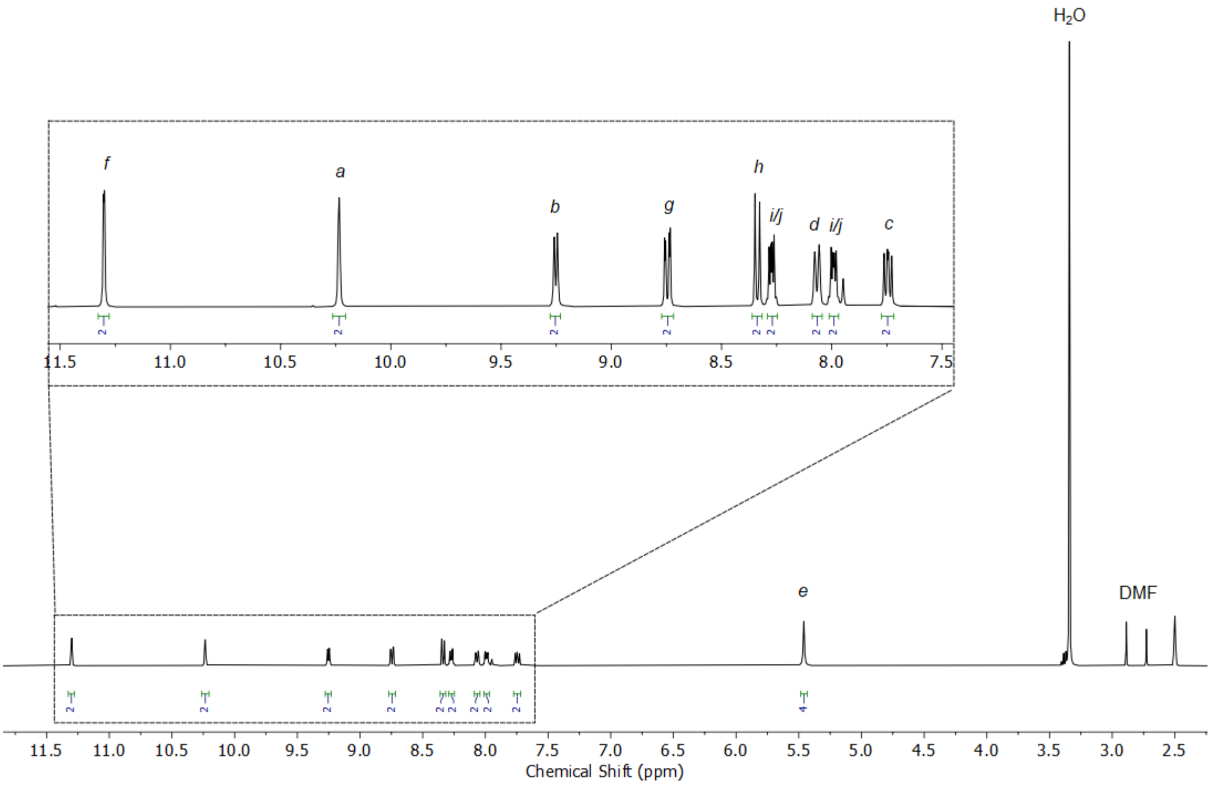

Figure S21 ^{1}H NMR (400 MHz, d_6-DMSO) of $[Pd_2(\mathbf{L^{P4}})_2⊃Cl](BF_4)_3$.

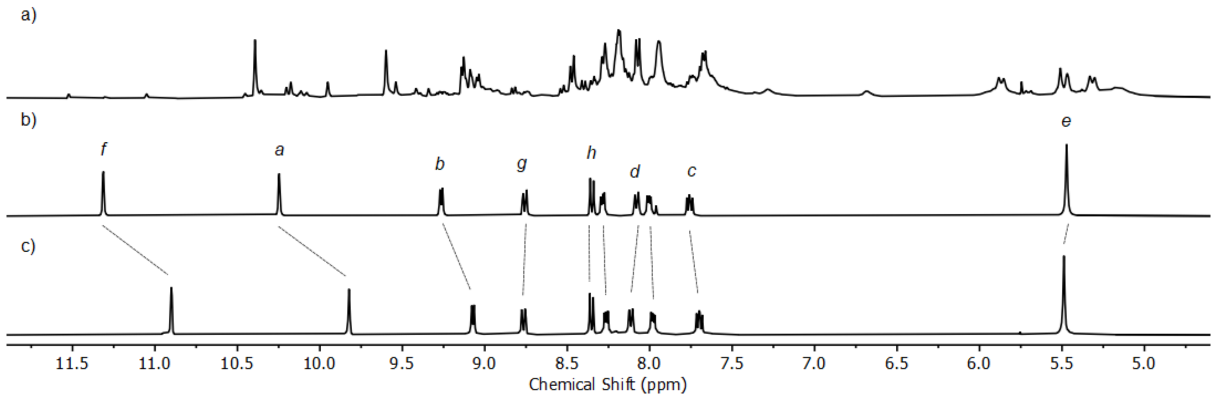

Figure S22 Partial ^{1}H NMR (400 MHz, d_6-DMSO, 298 K) of a) 1:1 mixture of $\mathbf{L^{P4}}$ and $[Pd(CH_3CN)_4](BF_4)_2$ after standing at rt for 2 d; b) $[Pd_2(\mathbf{L^{P4}})_2{\supset}Cl](BF_4)_3$, and c) $[Pd_2(\mathbf{L^{P4}})_2{\supset}NO_3](NO_3)_3$.

Synthesis of S3

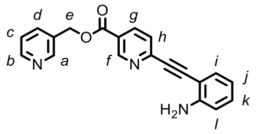

S1 (0.586 g, 2.0 mmol, 1 eq.), 2-ethynylaniline (0.281 g, 2.4 mmol, 1.2 eq.), [Pd(PPh$_3$)$_2$Cl$_2$] (0.035 g, 0.050 mmol, 2.5 mol%) and CuI (0.010 g, 0.050 mmol, 2.5 mol%) were stirred at rt in 2:1 dioxane/^{i}Pr$_2$NH (15 mL) for 48 h. EDTA solution (25 mL) was added and the reaction mixture extracted with CH_2Cl_2 (3 × 20 mL). The combined organic layers were dried ($MgSO_4$) and the solvent removed *in vacuo*. Following purification by column chromatography on silica gel (step gradient 0:10 to 5:5 acetone/CH_2Cl_2 in 10% increments) the product was obtained as a fluffy yellow solid (0.560 g, 85%).

^{1}H NMR (400 MHz, $CDCl_3$) δ: 9.21 (dd, *J* = 2.2, 0.9 Hz, 1H, H_f), 8.74 (br. s, 1H, H_a), 8.63 (br. s, 1H, H_b), 8.28 (dd, *J* = 8.2, 2.2 Hz, 1H, H_g), 7.80 (app. dt, *J* = 7.8, 1.9 Hz, 1H, H_d), 7.57 (dd, *J* = 8.2, 0.9 Hz, 1H, H_h), 7.42 (dd, *J* = 8.0, 1.6 Hz, 1H, H_i), 7.35 (dd, *J* = 7.8, 4.8 Hz, 1H, H_c) 7.19 (app. td, *J* = 7.6, 1.5 Hz, 1H, H_k), 6.74-6.70 (m, 2H, H_j, H_l), 5.42 (s, 2H, H_e), 4.42 (br. s, 2H, H_{NH}).

^{13}C NMR (101 MHz, $CDCl_3$) δ: 164.7, 151.4 (C_f), 150.2 (C_b), 150.0 (C_a), 149.0, 147.7, 137.4 (C_g), 136.4 (C_d), 133.0 (C_i), 131.3 (C_k), 126.5 (C_h), 124.0, 123.8 (C_c), 118.1 (C_j/C_l), 114.7 (C_j/C_l), 106.0, 94.0, 90.1, 64.8 (C_e).

HR-ESI-MS m/z = 330.1241 [M+H]$^+$ calc. 330.1243.

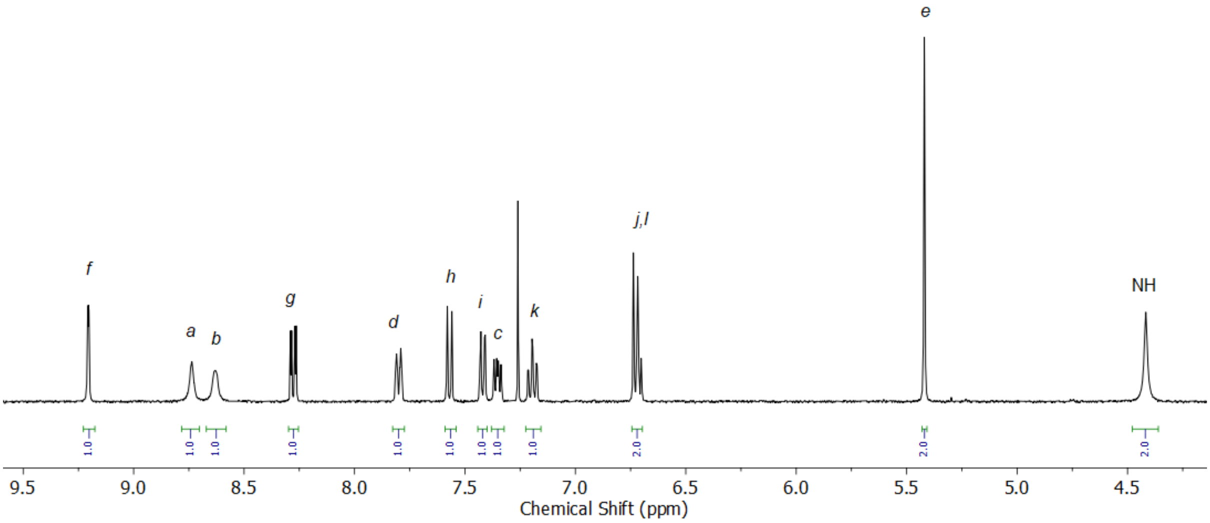

Figure S23 ^{1}H NMR (400 MHz, $CDCl_3$) of **S3**.

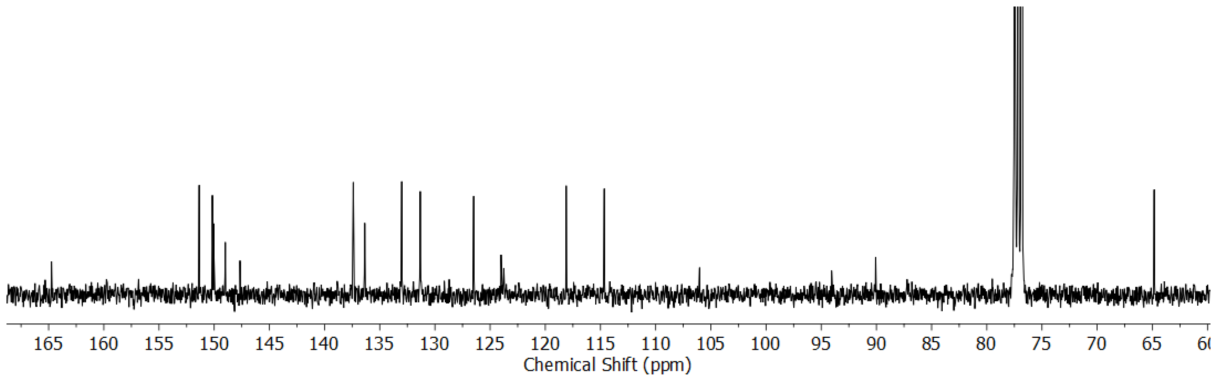

Figure S24 ^{13}C NMR (101 MHz, $CDCl_3$) of **S3**.

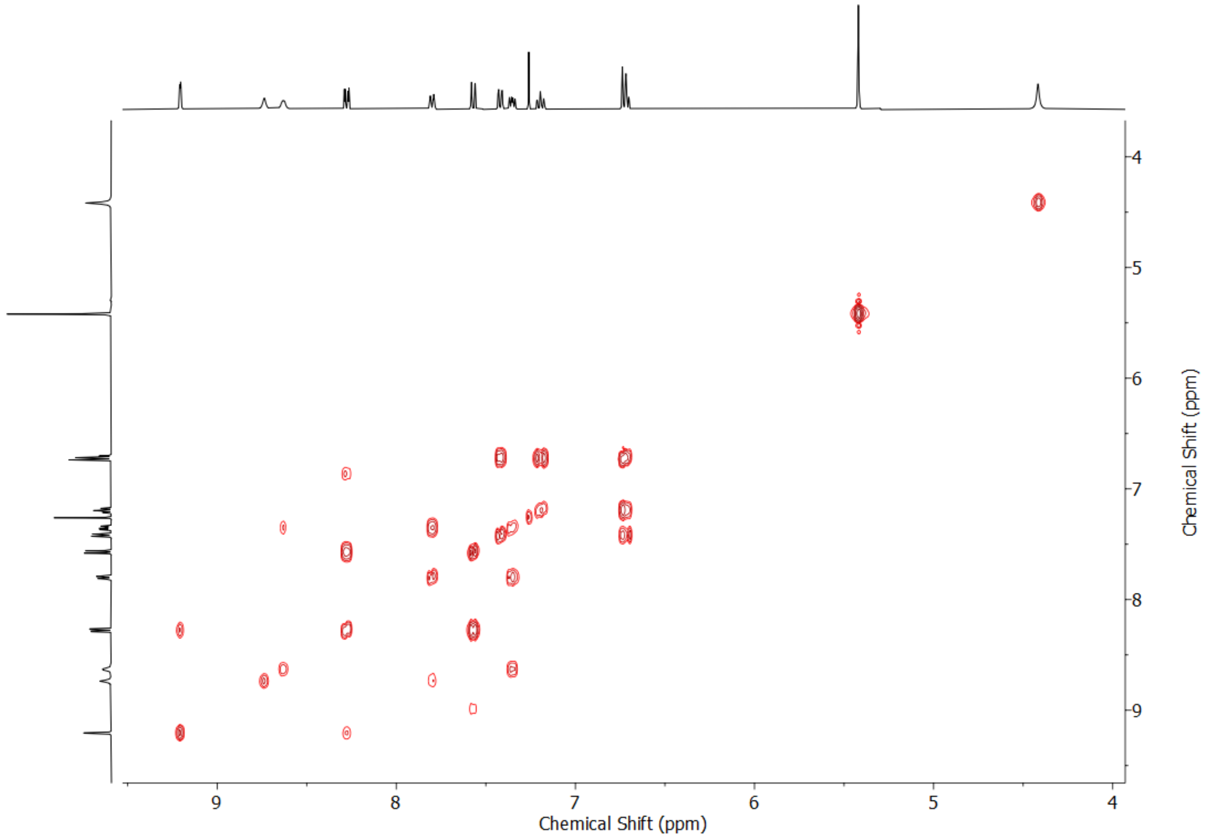

Figure S25 COSY NMR ($CDCl_3$) of **S3**.

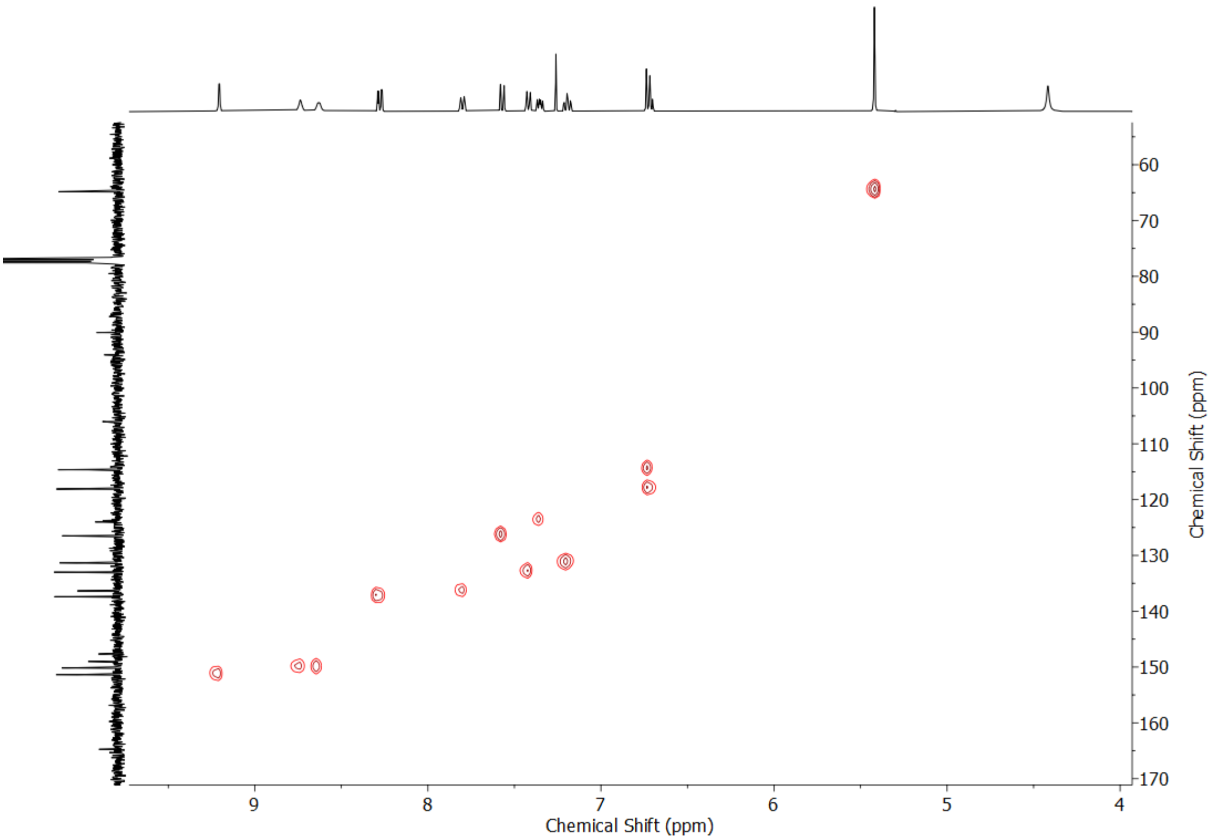

Figure S26 HSQC NMR ($CDCl_3$) of **S3**.

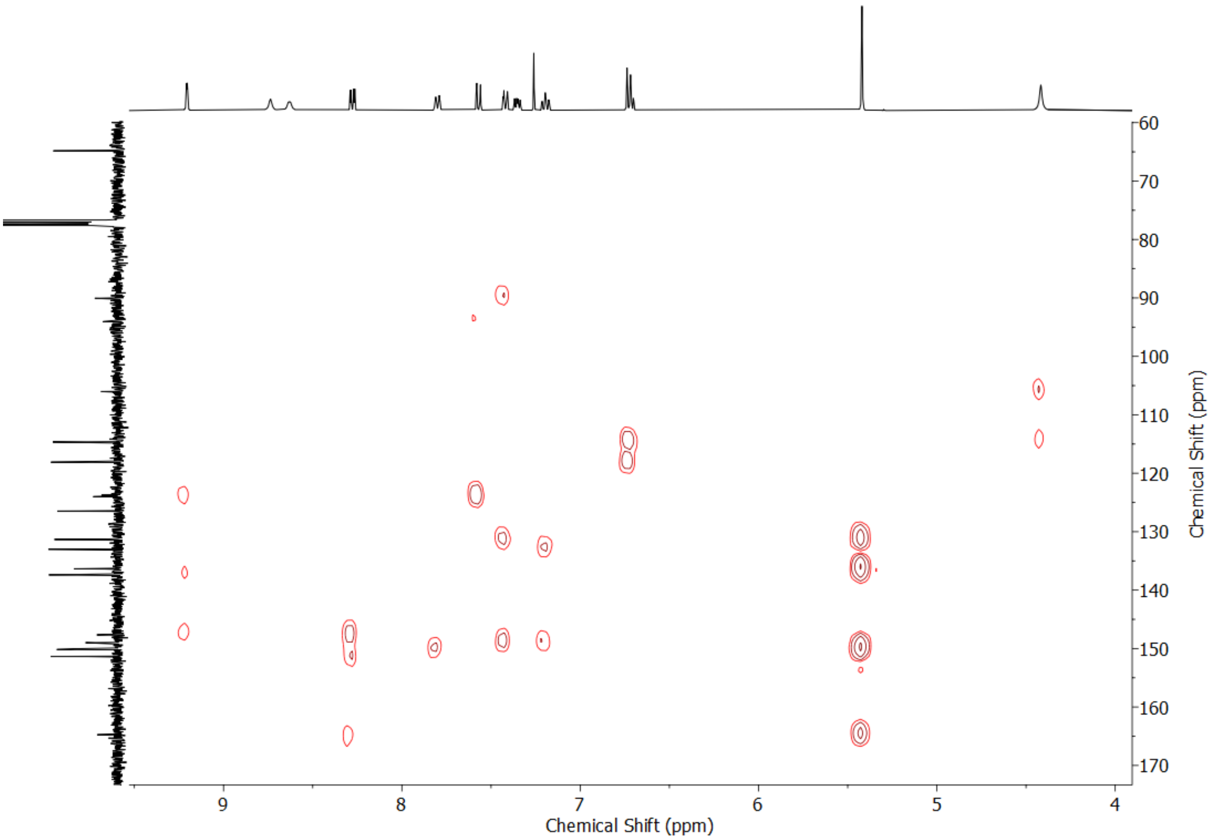

Figure S27 HMBC NMR ($CDCl_3$) of **S3**.

Synthesis of S4

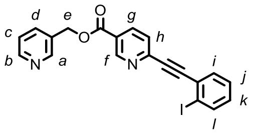

To a vigorously stirring mixture of **S3** (0.494 g, 1.5 mmol, 1 eq.) and TsOH·H_2O (0.856 g, 4.5 mmol, 3 eq.) in CH_3CN (9 mL) at 0 °C under air was added dropwise a solution of $NaNO_2$ (0.207 g, 3.0 mmol, 2 eq.) and KI (0.623 g, 3.75 mmol, 2.5 eq.) in H_2O (1 mL). The reaction was stirred, allowing to warm to rt, for 21 h. H_2O (20 mL), sat. aq. $NaHCO_3$ (20 mL) and 0.5 M $Na_2S_2O_{3(aq)}$ (20 mL) were added sequentially. The reaction mixtures was extracted with EtOAc (3 × 25 mL) and the combined organic phases dried ($MgSO_4$) and the solvent removed *in vacuo*. After purification by column chromatography on silica gel (1:9 acetone/CH_2Cl_2) the product was obtained as a yellow oil (0.476 g, 72%) that solidified on standing.

^{1}H NMR (400 MHz, $CDCl_3$) δ: 9.24 (dd, *J* = 2.2, 0.9 Hz, 1H, H_f), 8.73 (d, *J* = 1.9 Hz, 1H, H_a), 8.63 (dd, *J* = 4.9, 1.7 Hz, 1H, H_b), 8.30 (dd, *J* = 8.2, 2.2 Hz, 1H, H_g), 7.90 (dd, *J* = 8.0, 1.1 Hz, 1H, H_l), 7.80 (ddd, *J* = 7.9, 2.3, 1.7 Hz, 1H, H_d), 7.69 (dd, *J* = 8.2, 0.9 Hz, 1H, H_h), 7.62 (dd, *J* = 7.8, 1.7 Hz, 2H, H_i), 7.38-7.33 (m, 2H, H_c, H_j), 7.08 (td, *J* = 7.7, 1.7 Hz, 1H, H_k), 5.42 (s, 2H, H_e).

^{13}C NMR (101 MHz, $CDCl_3$) δ: 164.7, 151.4 (C_f), 150.2 (C_b), 150.0 (C_a), 147.3, 139.1 (C_l), 137.4 (C_g), 136.4 (C_d), 133.5 (C_i), 131.2, 130.8 (C_k), 128.4, 128.1 (C_c/C_j), 127.2 (C_h), 124.5, 123.7 (C_c/C_j), 101.4, 94.4, 91.5, 64.9 (C_e).

HR-ESI-MS m/z = 441.0087 $[M+H]^+$ calc. 441.0100.

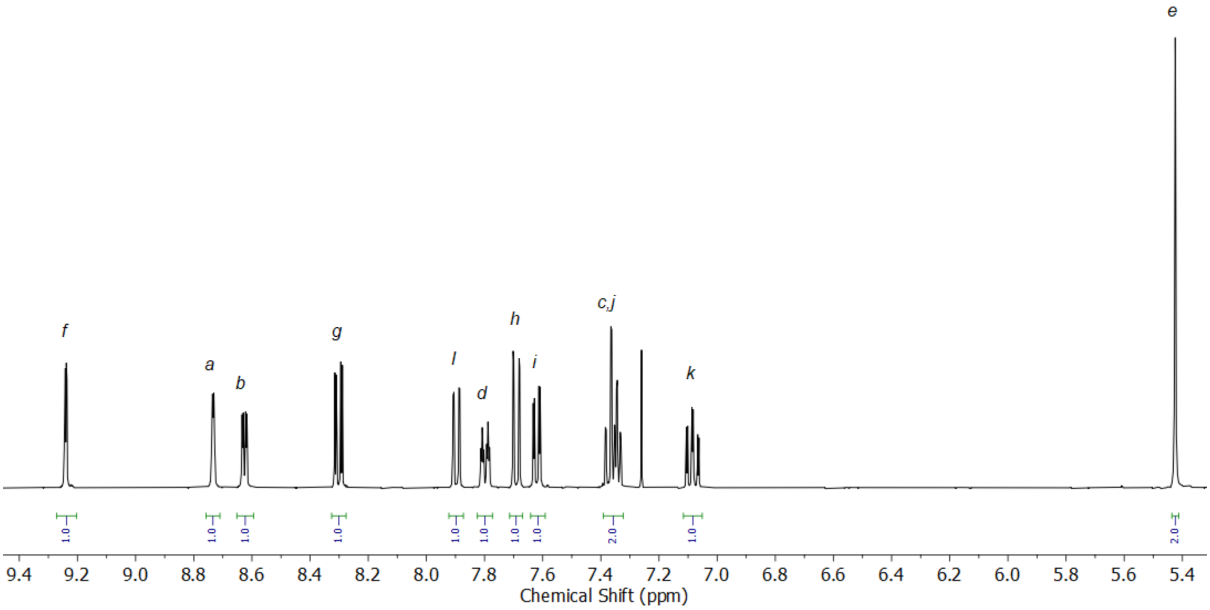

Figure S28 ^{1}H NMR (400 MHz, $CDCl_3$) of **S4**.

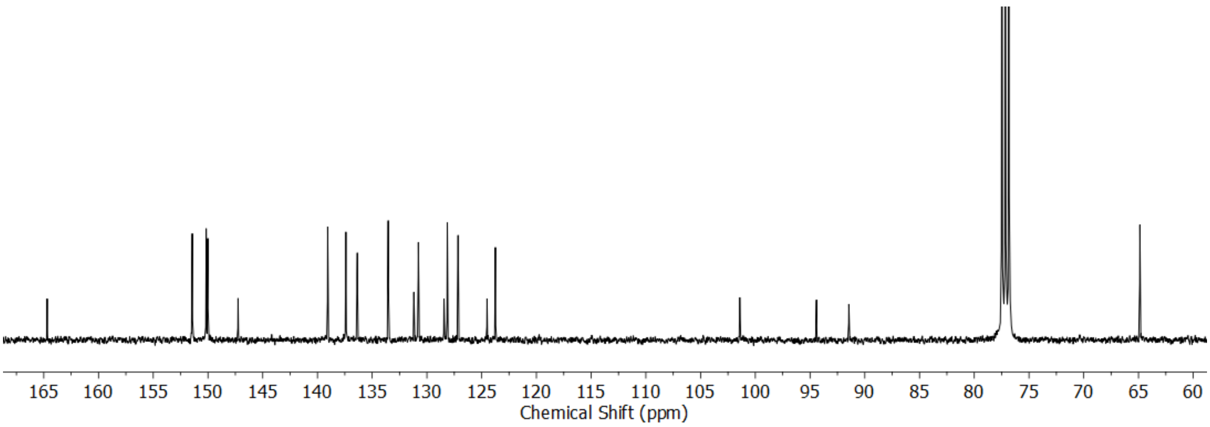

Figure S29 ^{13}C NMR (101 MHz, $CDCl_3$) of **S4**.

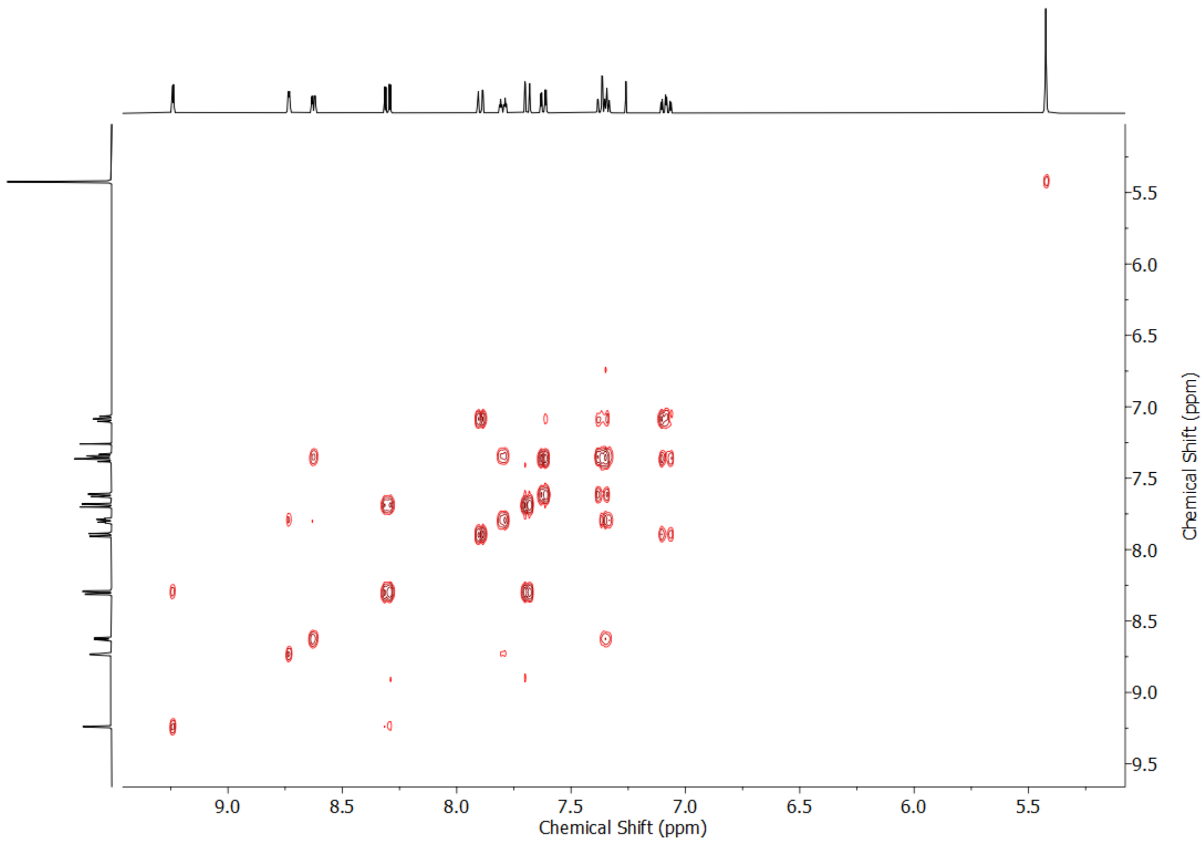

Figure S30 COSY NMR ($CDCl_3$) of **S4**.

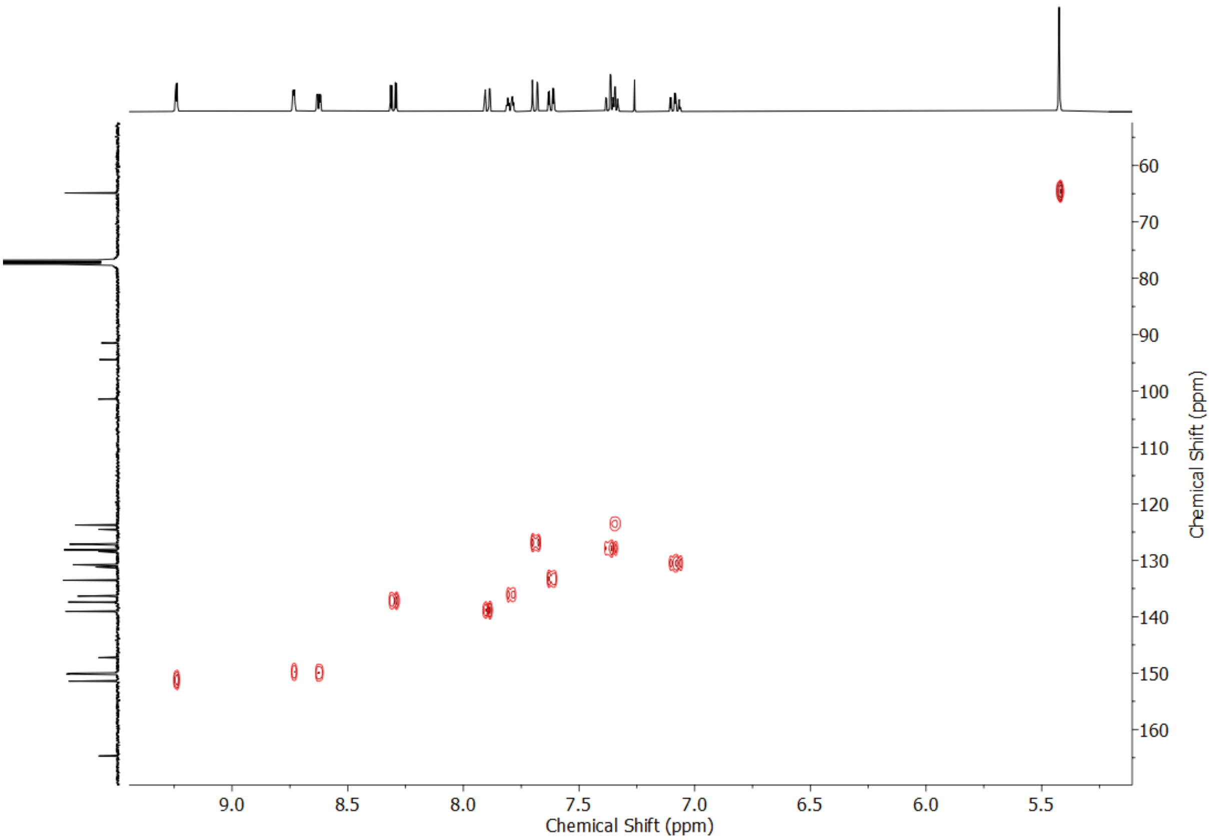

Figure S31 HSQC NMR ($CDCl_3$) of **S4**.

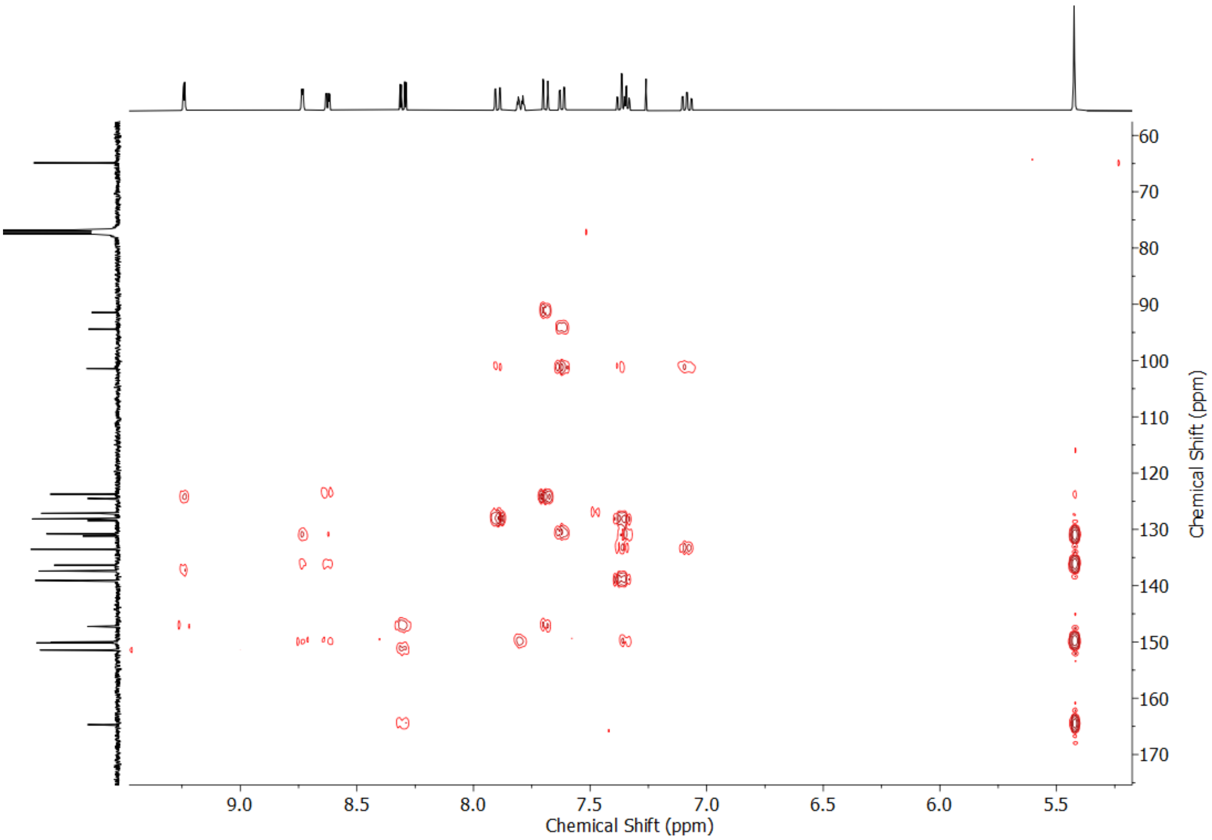

Figure S32 HMBC NMR ($CDCl_3$) of **S4**.

Synthesis of S5

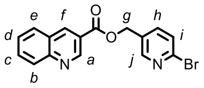

To a stirring solution of EDCI·HCl (0.690 g, 3.6 mmol, 1.2 eq.) and DMAP (0.047 g, 0.3 mmol, 0.1 eq.) in $CHCl_3$ (15 mL) at 0 °C was added 3-quinolinecarboxylic acid (0.520 g, 3.0 mmol, 1 eq.) as a solid. After 30 minutes, 6-bromopyridine-3-methanol (0.620 g, 3.3 mmol, 1.1 eq.) was added as a solid. The reaction mixture was allowed to warm to rt and stirred for 18 h before washing with sat. aq. $NaHCO_3$ (25 mL) and brine (25 mL), drying ($MgSO_4$) and the solvent removed *in vacuo*. After purification by column chromatography on silica gel (1:9 acetone/CH_2Cl_2) the product was obtained as a white solid (0.929 g, 90%).

^{1}H NMR (400 MHz, $CDCl_3$) δ: 9.44 (d, *J* = 2.1 Hz, 1H, H_a), 8.84 (d, *J* = 2.0 Hz, 1H, H_f), 8.53 (dd, *J* = 2.5, 0.7 Hz, 1H, H_j), 8.17 (dd, *J* = 8.5, 1.0 Hz, 1H, H_e), 7.94 (m, 1H, H_b), 7.86 (ddd, *J* = 8.5, 6.9, 1.4 Hz, 1H, H_d), 7.71 (dd, *J* = 8.2, 2.5 Hz, 1H, H_h), 7.64 (ddd, *J* = 8.1, 6.9, 1.2 Hz, 1H, H_c), 7.55 (dd, *J* = 8.2, 0.7 Hz, 1H, H_i), 5.43 (s, 2H, H_g).

^{13}C NMR (101 MHz, $CDCl_3$) δ: 165.2, 150.4 (C_j), 150.2, 150.0 (C_a), 142.6, 139.2 (C_f), 139.0 (C_h), 132.4 (C_d), 130.8, 129.7 (C_e), 129.3 (C_b), 128.4 (C_i), 127.8 (C_c), 126.9, 122.5, 63.9 (C_g).

HR-ESI-MS m/z = 343.0086 $[M+H]^+$ calc. 343.0082.

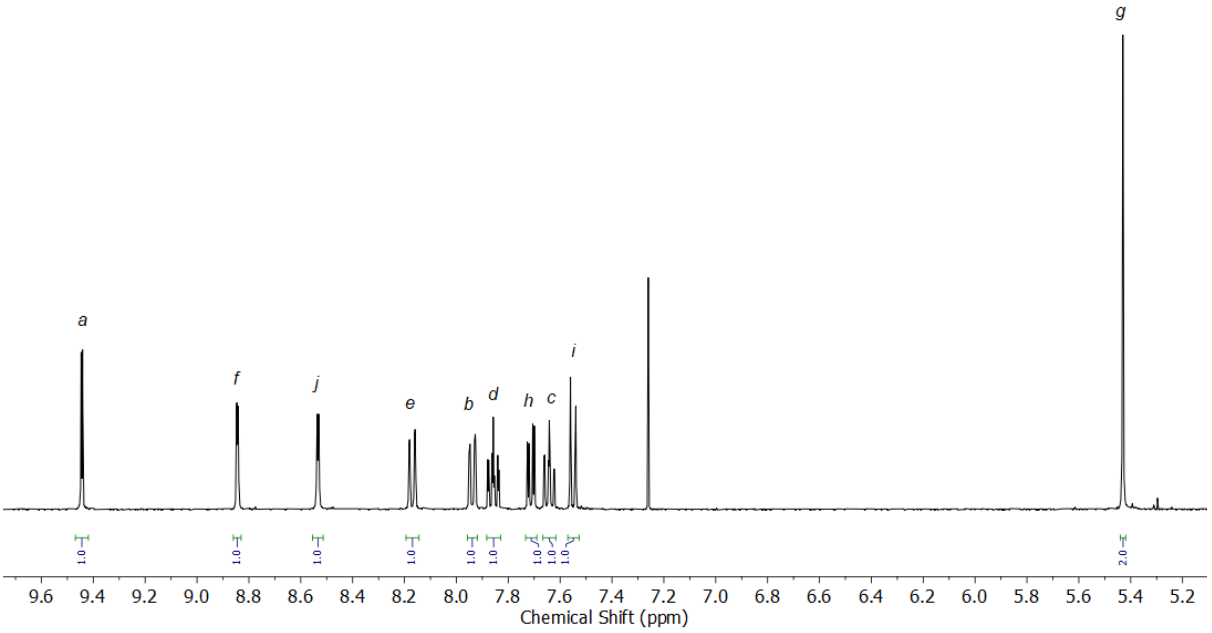

Figure S33 ^{1}H NMR (400 MHz, $CDCl_3$) of **S5**.

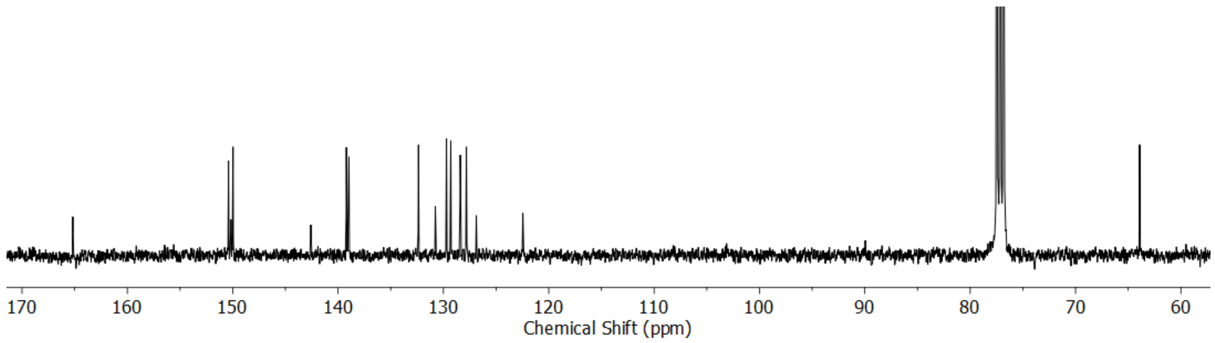

Figure S34 ^{13}C NMR (101 MHz, $CDCl_3$) of **S5**.

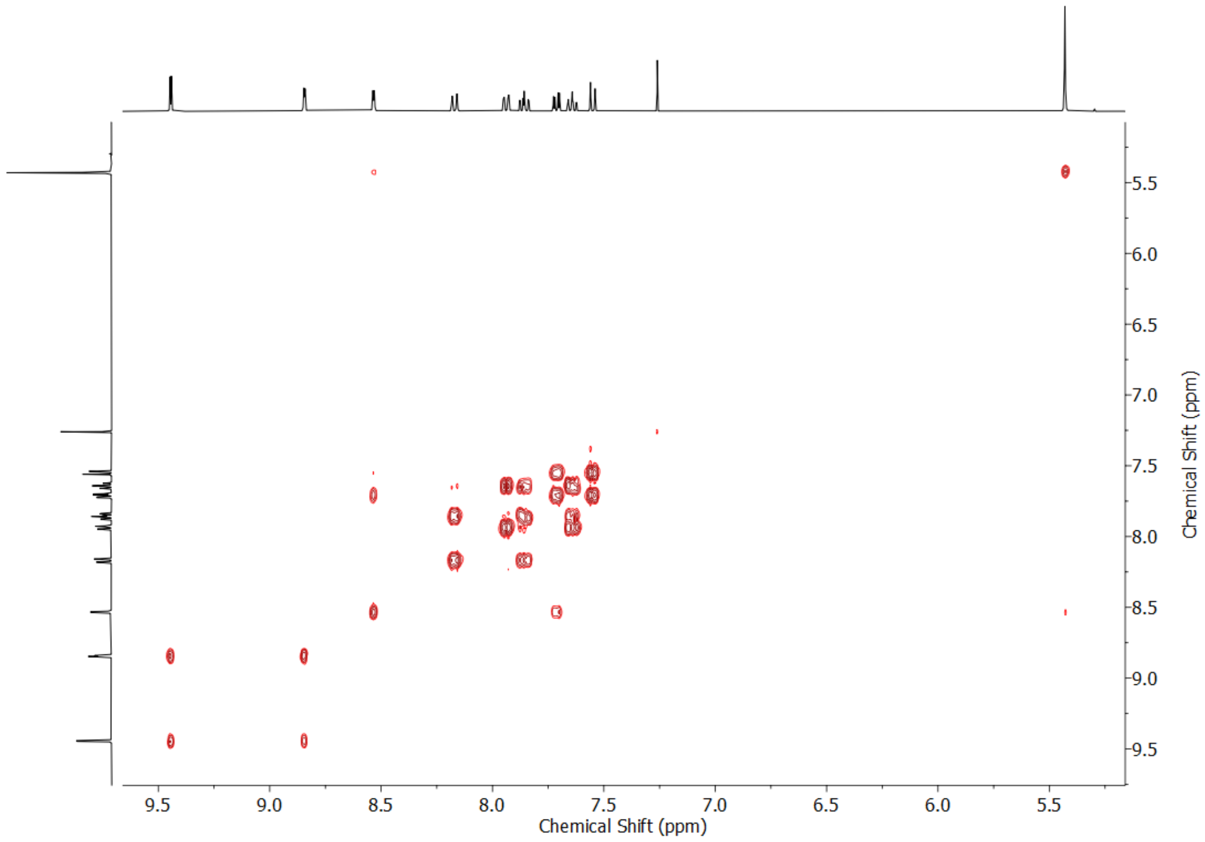

Figure S35 COSY NMR ($CDCl_3$) of **S5**.

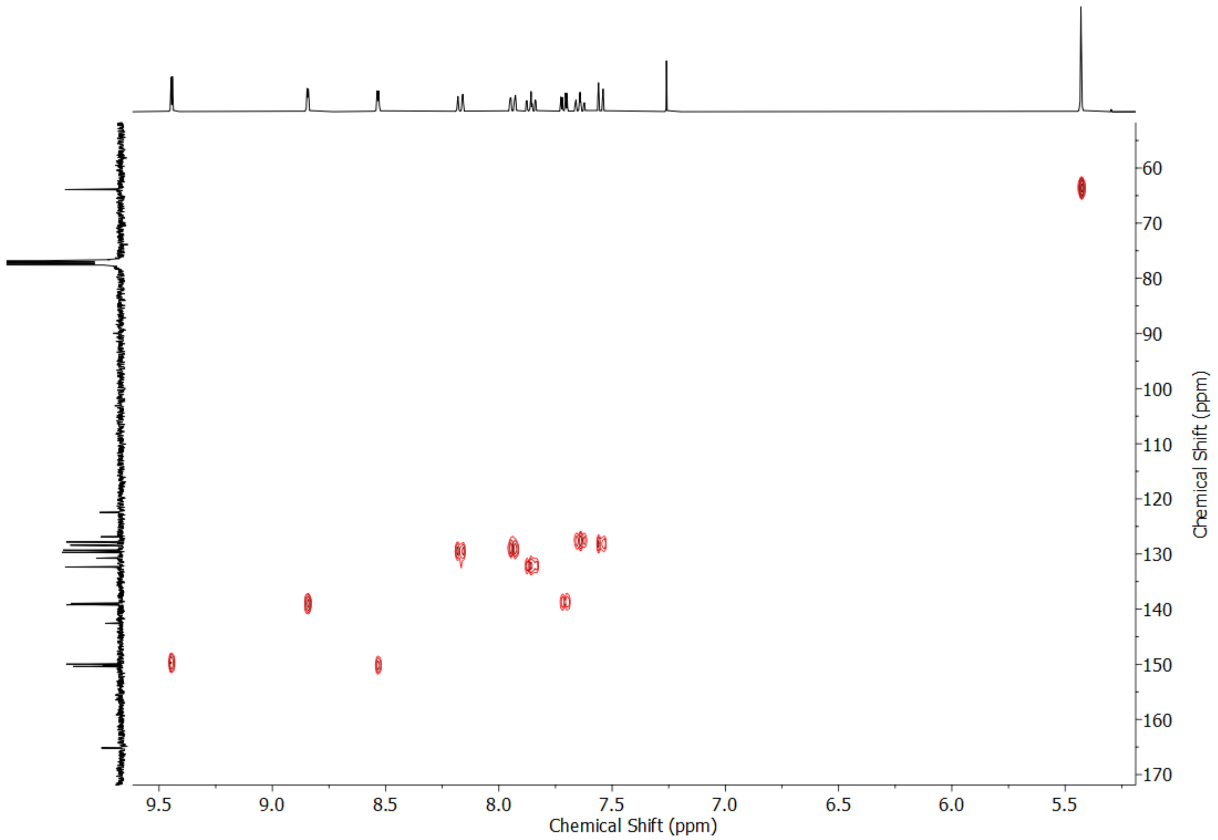

Figure S36 HSQC NMR ($CDCl_3$) of **S5**.

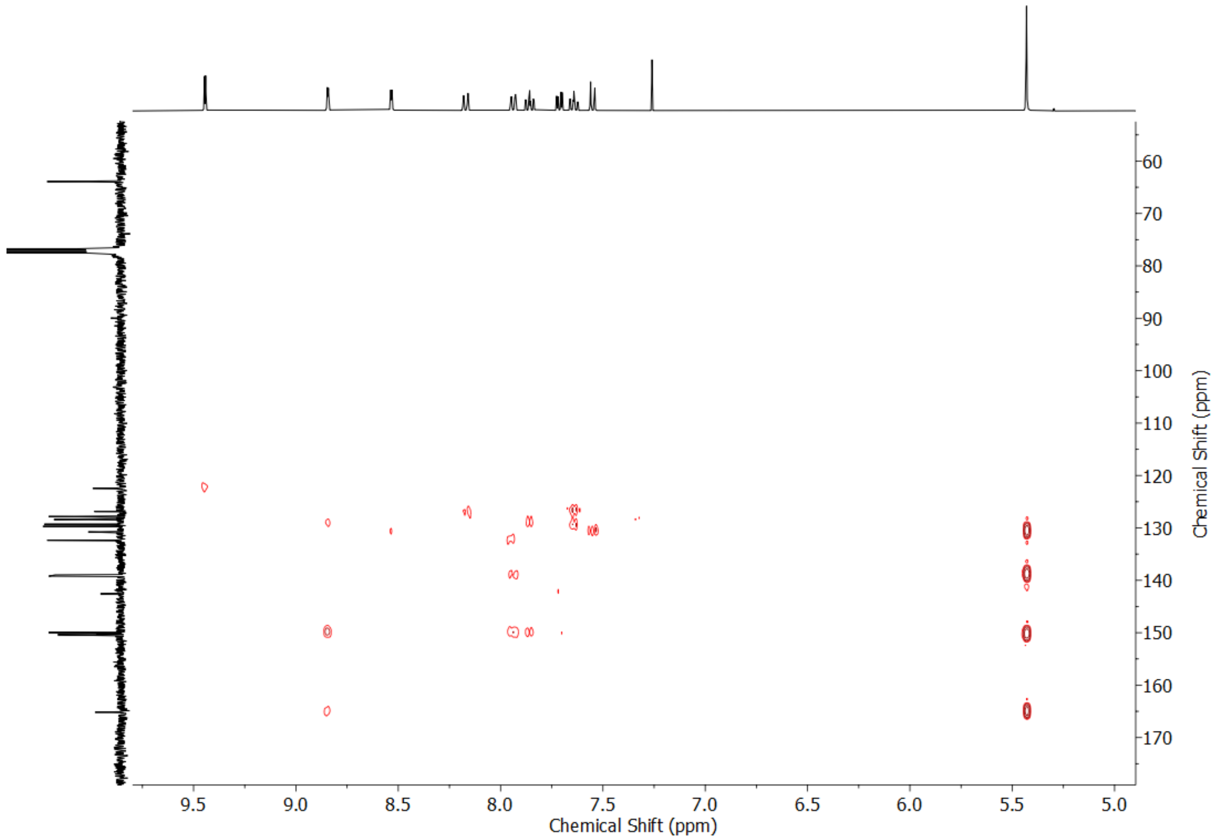

Figure S37 HMBC NMR ($CDCl_3$) of **S5**.

Synthesis of S6

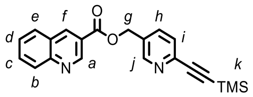

To **S5** (0.343 g, 1.0 mmol, 1 eq.), [Pd(PPh_3)$_2$$Cl_2$] (0.018 g, 0.025 mmol, 2.5 mol%) and CuI (0.010 g, 0.05 mmol, 5 mol%) in $^{i}Pr_2NH$ (5 mL) was added trimethylsilylacetylene (0.21 mL, 1.5 mmol, 1.5 eq.) via syringe. After stirring at rt for 28 h, EDTA solution (20 mL) was added and the aqueous phase was extracted with CH_2Cl_2 (3 × 15 mL). The combined organic phases were dried ($MgSO_4$) and the solvent removed *in vacuo*. After purification by column chromatography (1st column 1:19 acetone/CH_2Cl_2; 2nd column 0→30% EtOAc in pentane in 10% increments) the product was obtained as an off-white solid (0.266 g, 74%).

^{1}H NMR (400 MHz, $CDCl_3$) δ: 9.45 (d, *J* = 2.1 Hz, 1H, H_a), 8.85 (dd, *J* = 2.2, 0.9 Hz, 1H, H_f), 8.71 (dd, *J* = 2.3, 0.9 Hz, 1H, H_j), 8.17 (dd, *J* = 8.5, 1.0 Hz, 1H, H_e), 7.94 (dd, *J* = 8.1, 1.3 Hz, 1H, H_b), 7.86 (ddd, *J* = 8.5, 6.9, 1.5 Hz, 1H, H_d), 7.79 (dd, *J* = 8.1, 2.3 Hz, 1H, H_h), 7.64 (ddd, *J* = 8.2, 6.9, 1.2 Hz, 1H, H_c), 7.51 (dd, *J* = 8.0, 0.9 Hz, 1H, H_i), 5.46 (s, 2H, H_g), 0.27 (s, 9H, H_k).

^{13}C NMR (101 MHz, $CDCl_3$) δ: 165.2, 150.1, 150.1 (C_j), 150.0 (C_a), 143.4, 139.2 (C_f), 136.5 (C_h), 132.3 (C_d), 130.8, 129.7 (C_e), 129.3 (C_b), 127.8 (C_c), 127.3 (C_i), 126.9, 122.6, 103.4, 95.9, 64.4 (C_g), -0.2 (C_k).

HR-ESI-MS m/z = 361.1454 [M+H]$^+$ calc. 361.1367.

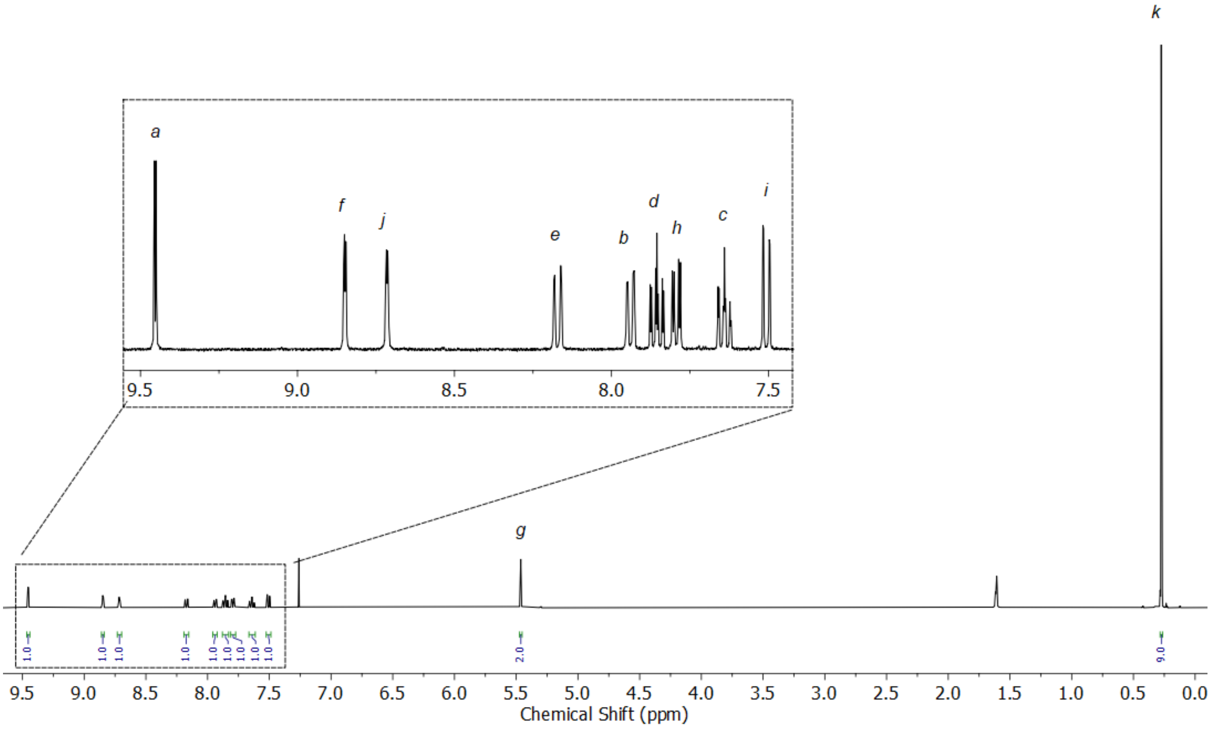

Figure S38 ^{1}H NMR (400 MHz, $CDCl_3$) of **S6**.

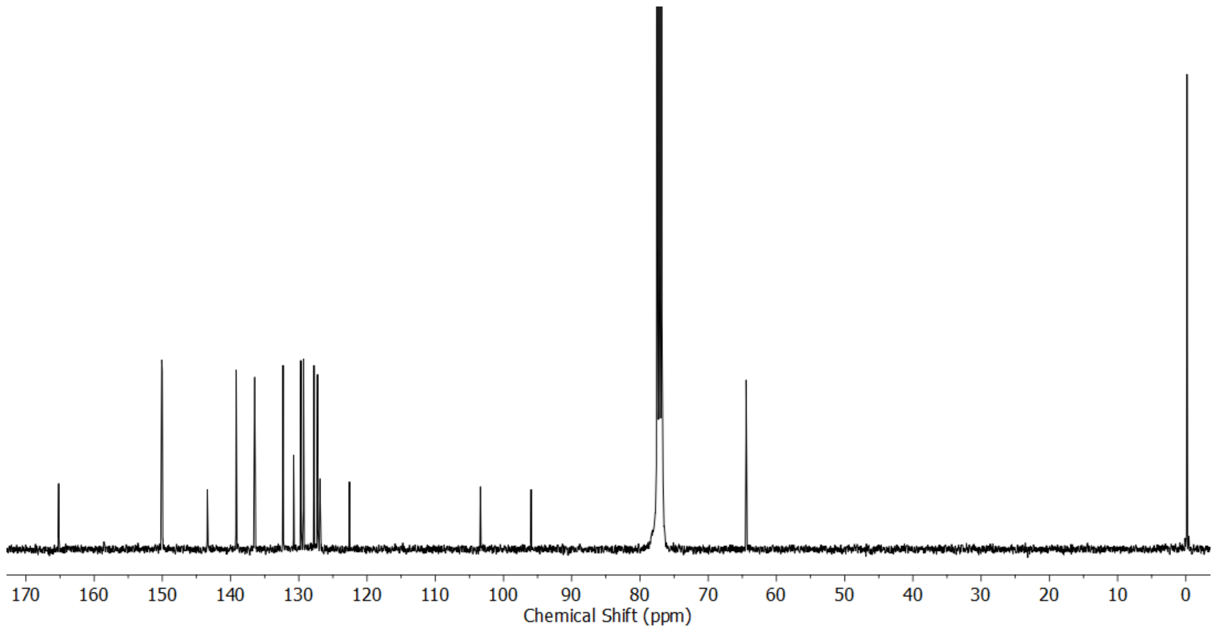

Figure S39 ^{13}C NMR (101 MHz, $CDCl_3$) of **S6**.

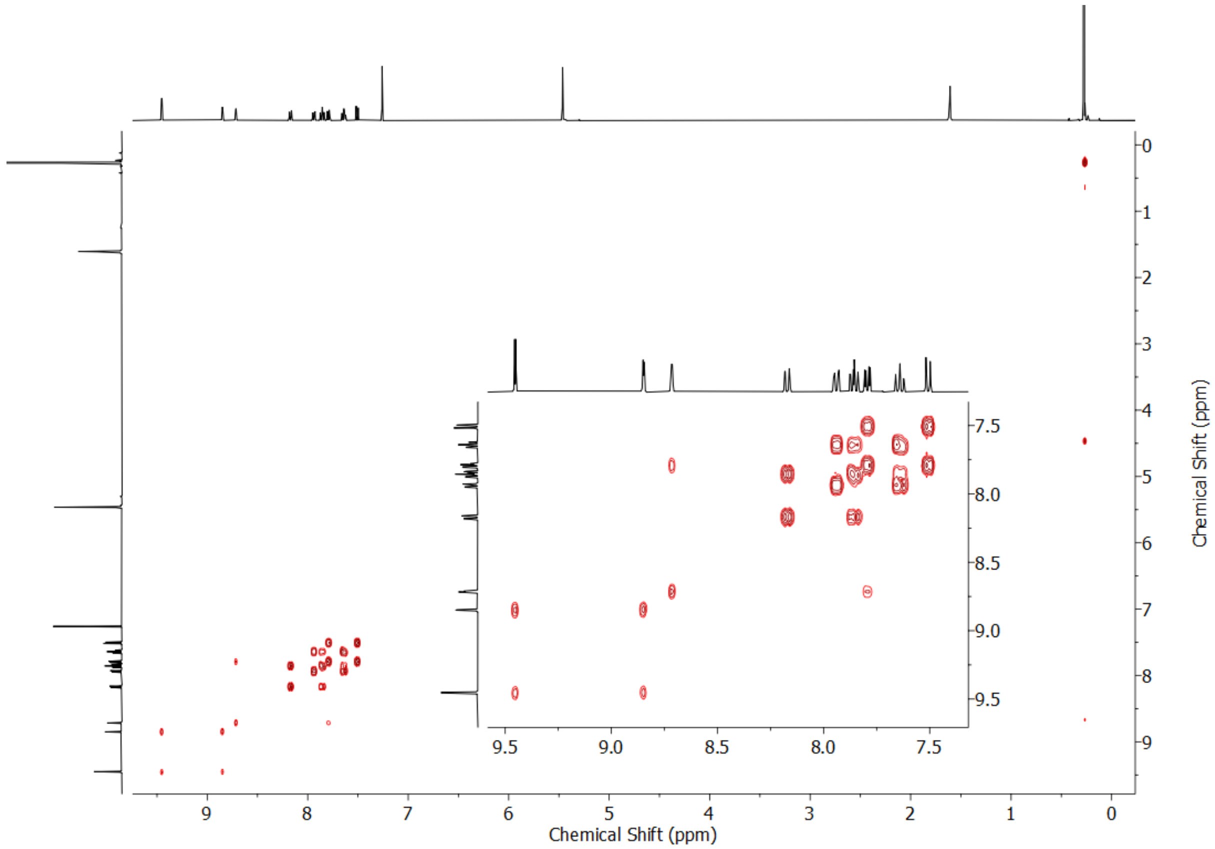

Figure S40 COSY NMR ($CDCl_3$) of **S6**.

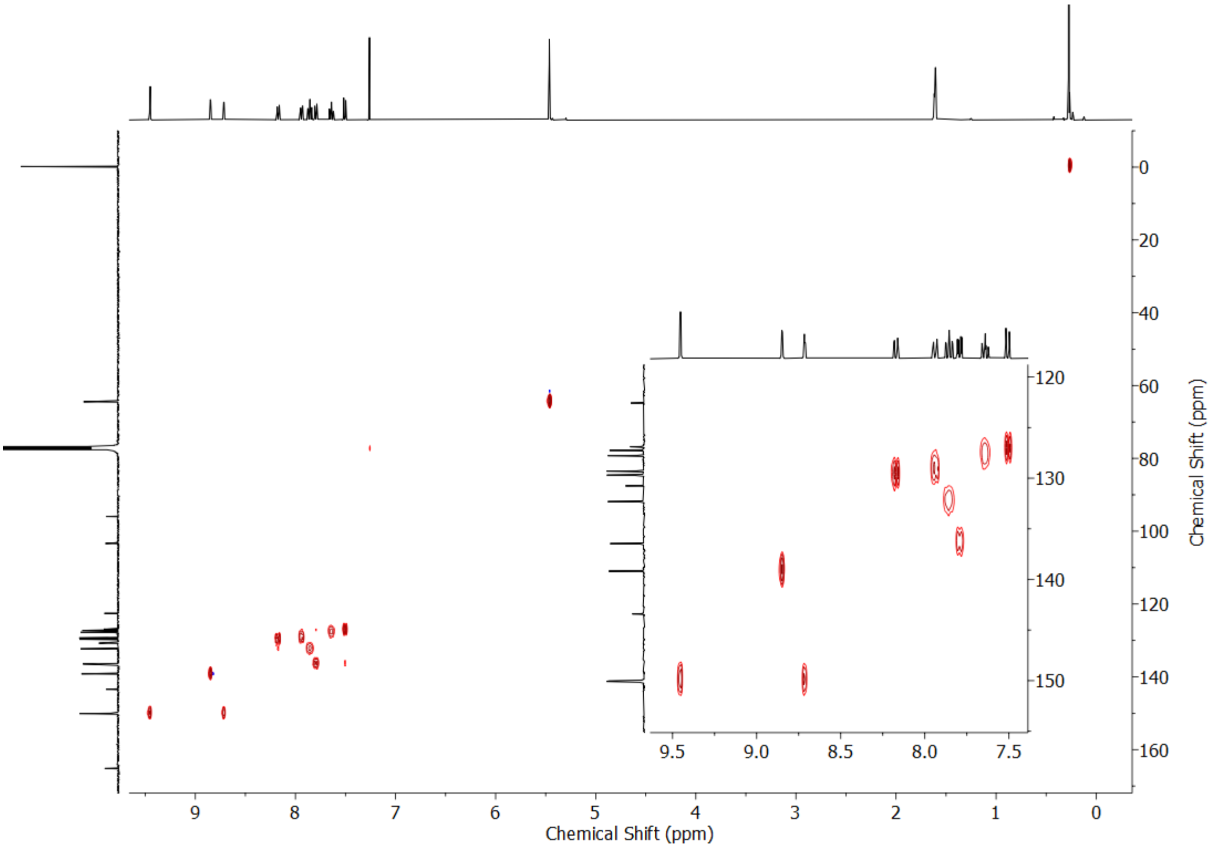

Figure S41 HSQC NMR ($CDCl_3$) of **S6**.

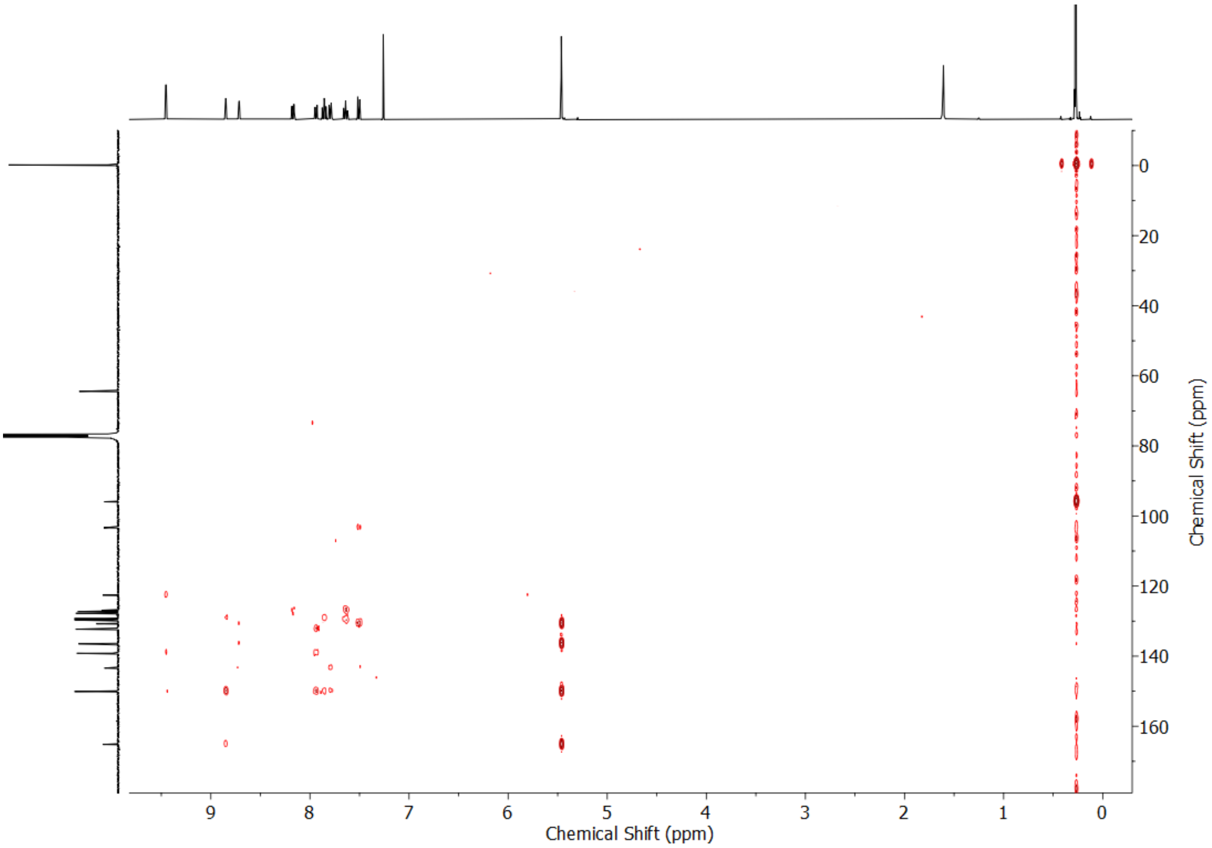

Figure S42 HMBC NMR ($CDCl_3$) of **S6**.

Synthesis of L[P3Q]

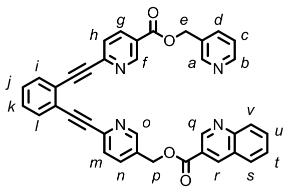

To a solution of **S4** (0.0817 g, 0.186 mmol, 1 eq.), **S6** (0.0669 g, 0.186 mmol, 1 eq.), $Pd(PPh_3)_4$ (0.0107 g, 0.0093 mmol, 5 mol%) and CuI (0.0018 g, 0.0093 mmol, 5 mol%) in MeCN (1.9 mL) was added DBU (0.17 mL, 1.1 mmol, 6 eq.). After stirring at rt for 21 h, EDTA solution (20 mL) was added. The aqueous phase was extracted with CH_2Cl_2 (3 × 10 mL), the combined organic phases dried ($MgSO_4$) and the solvent removed *in vacuo*. After purification by column chromatography on silica gel (0→40% acetone in CH_2Cl_2 in 10% increments) the product was obtained as a light yellow solid (0.0592 g, 53%).

^{1}H NMR (400 MHz, $CDCl_3$) δ: 9.46 (d, *J* = 1.6 Hz, 1H, H_q), 9.23 (d, *J* = 1.7 Hz, 1H, H_f), 8.86 (d, *J* = 1.9 Hz, 1H, H_r), 8.79 (d, *J* = 1.5 Hz, 1H, H_o), 8.75 (br. m, 1H, H_a), 8.63 (br. m, 1H, H_b), 8.29 (dd, *J* = 8.2, 2.2 Hz, 1H, H_g), 8.17 (d, *J* = 8.5 Hz, 1H, H_v), 7.94 (d, *J* = 8.2 Hz, 1H, H_s), 7.87-7.78 (m, 4H, H_d, H_h, H_n, H_u), 7.75 (d, *J* = 8.0 Hz, 1H, H_m), 7.67-7.61 (m, 3H, H_i, H_j, H_t), 7.43-7.37 (m, 2H, H_k, H_l), 7.34 (m, 1H, H_c), 5.49 (s, 2H, H_p), 5.41 (s, 2H, H_e).

^{13}C NMR (101 MHz, $CDCl_3$) δ: 165.2, 164.7, 151.3 (C_f), 150.3 (C_o), 150.1, 150.0, 150.0 (C_q), 149.9, 147.4, 143.6, 139.2 (C_r), 137.4 (C_g), 136.6 (C_d/C_h/C_n/C_u), 136.4 (C_d/C_h/C_n/C_u), 132.6 (C_i/C_j/C_t), 132.5 (C_i/C_j/C_t), 132.3 (C_d/C_h/C_n/C_u), 130.8, 129.6 (C_v), 129.5 (C_k/C_l), 129.3 (C_s), 129.1 (C_k/C_l), 127.8 (C_i/C_j/C_t), 127.6 (C_m), 127.4 (C_d/C_h/C_n/C_u), 126.9, 125.7, 125.0, 124.3, 123.8 (C_c) 122.6, 93.0, 92.6, 91.0, 88.3, 64.8 (C_e), 64.5 (C_p) (1 signal missing due to peak overlap).

HR-ESI-MS *m/z* = 601.1871 $[M+H]^+$ calc. 601.1876.

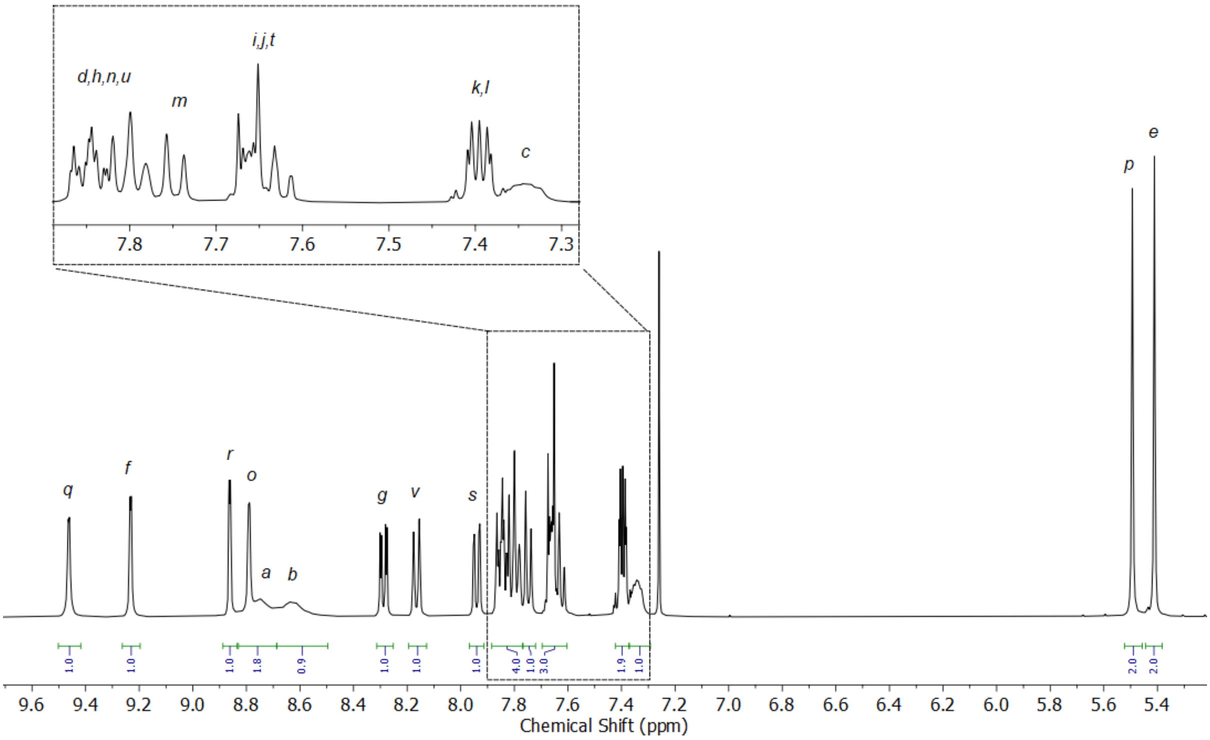

Figure S43 ^{1}H NMR (400 MHz, $CDCl_3$) of **L^{P3Q}**.

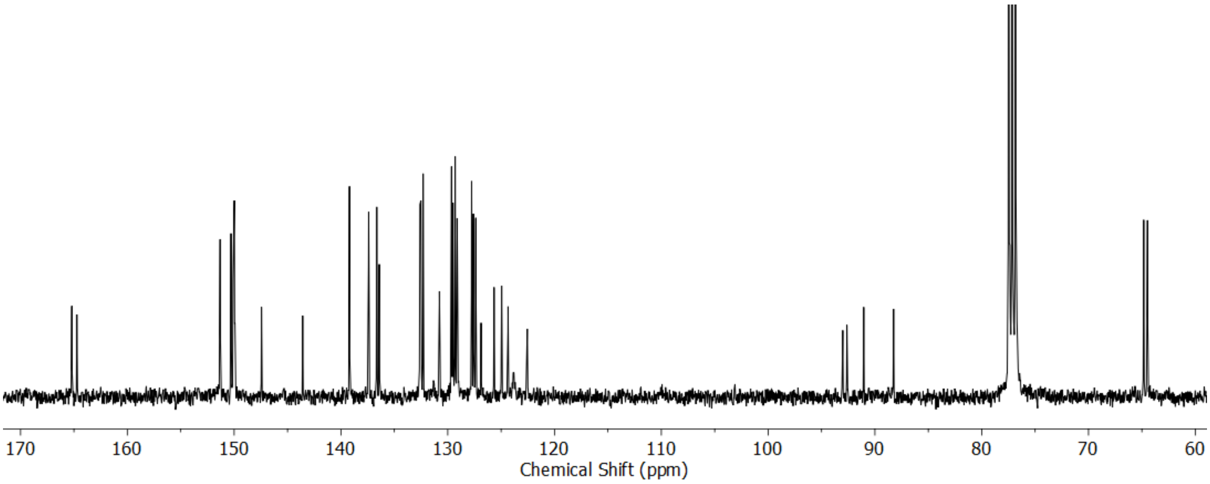

Figure S44 ^{13}C NMR (101 MHz, $CDCl_3$) of **L^{P3Q}**.

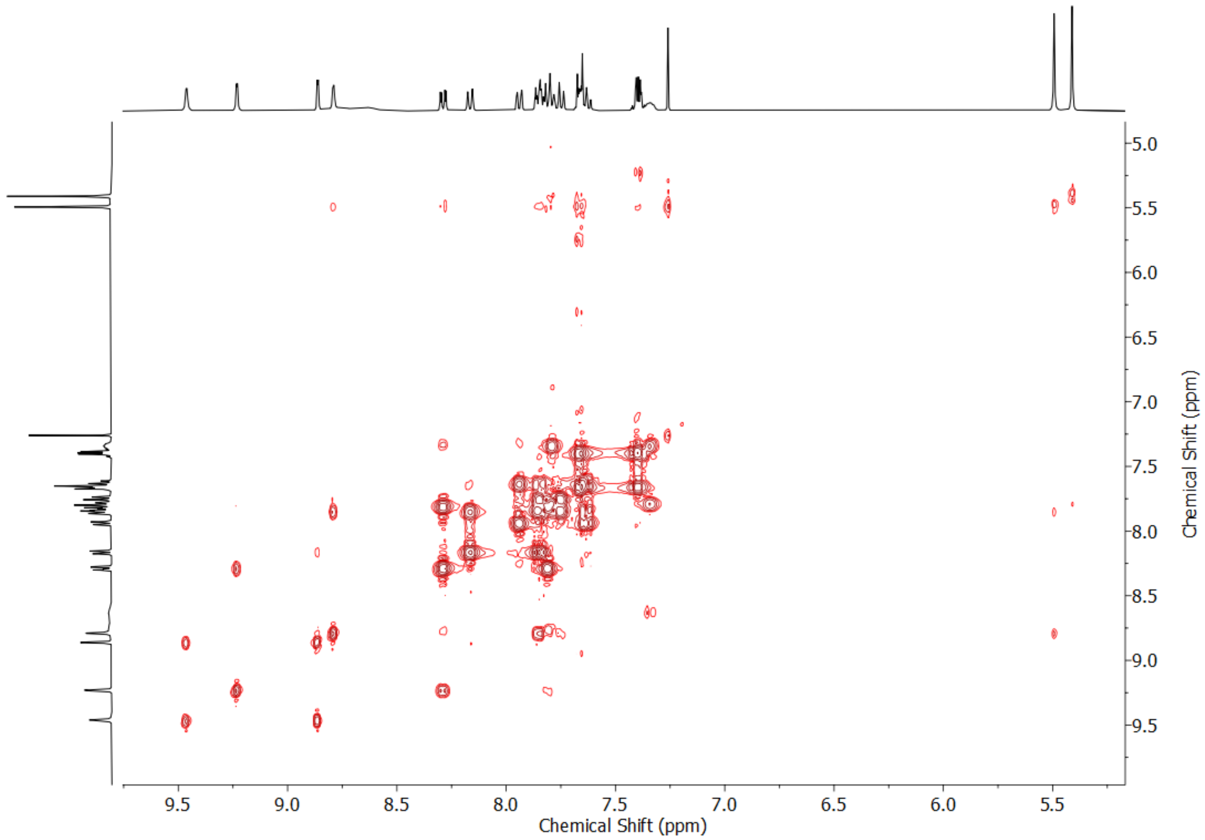

Figure S45 COSY NMR ($CDCl_3$) of **L^{P3Q}**.

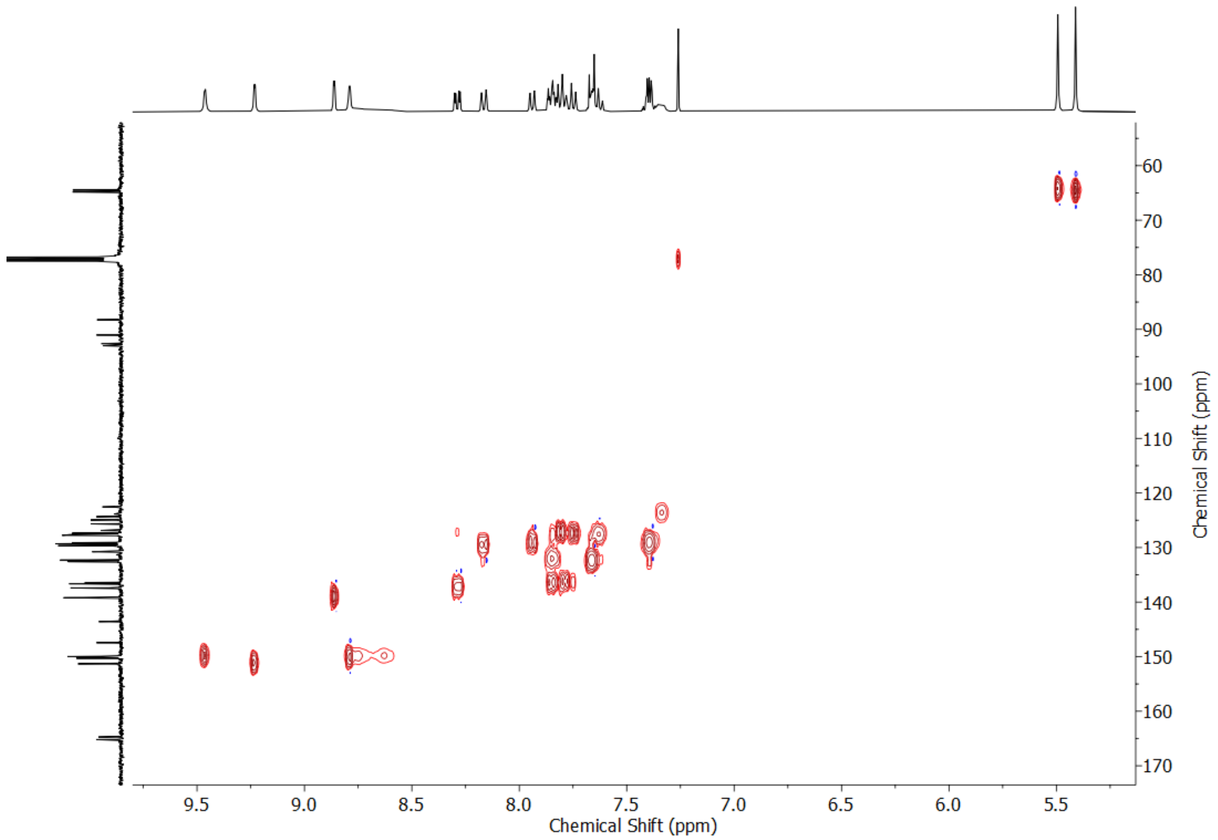

Figure S46 HSQC NMR ($CDCl_3$) of **L**$^{\mathbf{P3Q}}$.

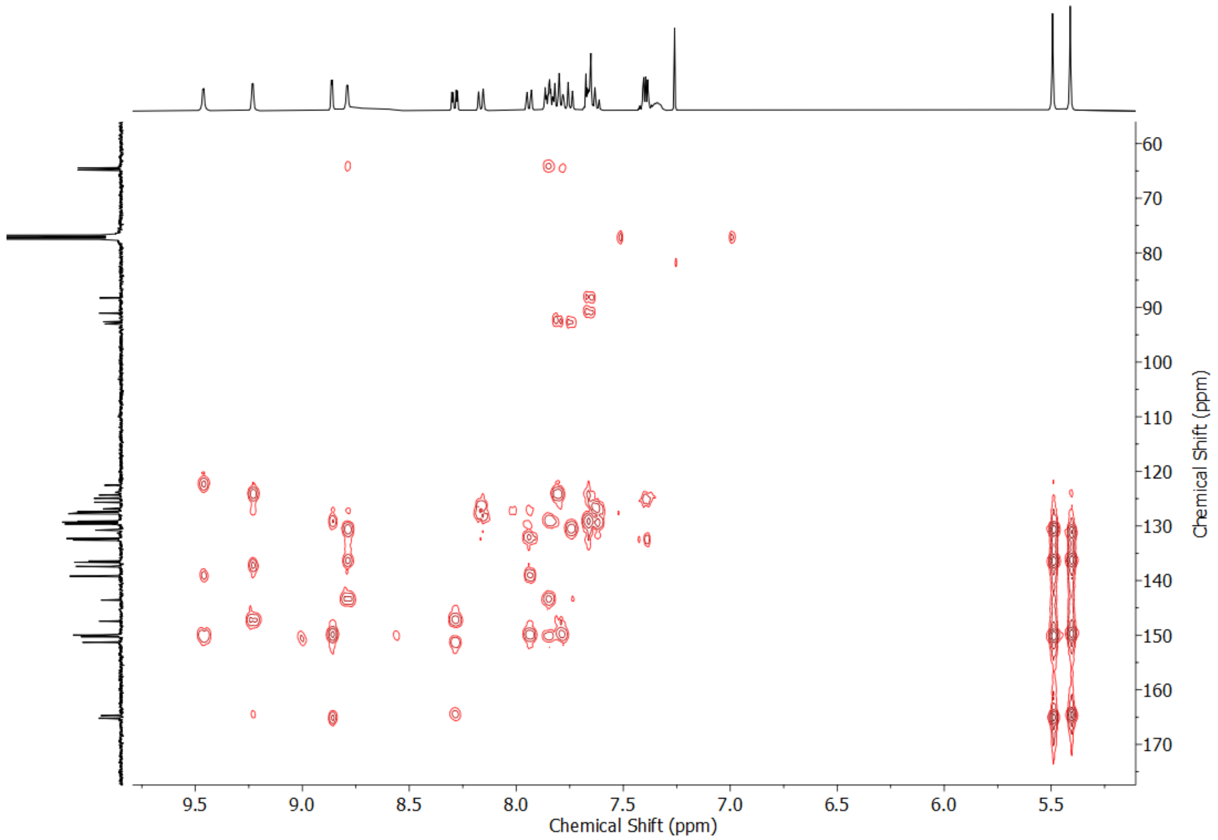

Figure S47 HMBC NMR ($CDCl_3$) of **L**$^{\mathbf{P3Q}}$.

Synthesis of $[Pd_2(L^{P3Q})_2 \supset NO_3](NO_3)_3$ (C^{P3Q})

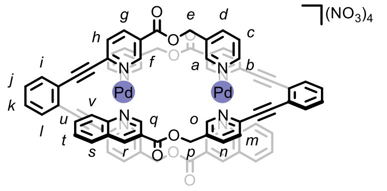

L^{P3Q} (12.0 mg, 20 µmol, 1 eq.) and $Pd(NO_3)_2{\cdot}2H_2O$ (5.3 mg, 20 µmol, 1 eq.) were sonicated in d_6-DMSO (0.75 mL) until a solution was obtained. After standing at 50 °C for 2 h, ^{1}H NMR demonstrated quantitative conversion to **C^{P4Q}**. The solution was diluted with MeCN (0.75 mL), filtered through celite and left for vapour diffusion of Et_2O. After 1 week the mother liquor was carefully decanted and the precipitate washed with Et_2O and dried *in vacuo* to give the product as a light yellow solid (12.6 mg, 76%).

^{1}H NMR (500 MHz, d_6-DMSO) δ: 11.34 (d, J = 1.7 Hz, 2H, H_q), 10.68-10.66 (m, 4H, H_f, H_v), 10.00 (s, 2H, H_o), 9.56 (m, 2H, H_r), 9.10 (s, 2H, H_a), 8.77 (d, J = 6.0 Hz, 2H, H_b), 8.62 (dd, J = 8.2, 1.8 Hz, 2H, H_g), 8.41 (m, 2H, H_s), 8.34 (m, 2H, H_n), 8.27-8.24 (m, 4H, H_h, 1 of H_i, H_j, H_k, H_l), 8.13-8.11 (m, 4H, H_d, 1 of H_i, H_j, H_k, H_l), 8.08 (d, J = 8.2 Hz, 3H, H_m), 8.00-7.95 (m, 4H, 2 of H_i, H_j, H_k, H_l), 7.87-7.81 (m, 4H, H_t, H_u), 7.59 (dd, J = 7.7, 6.1 Hz, 2H, H_c), 5.73 (d, J = 15.8 Hz, 2H, H_p), 5.51 (d, J = 15.6 Hz, 2H, $H_{p'}$), 5.41 (d, J = 13.4 Hz, 2H, H_e), 5.11 (d, J = 13.5 Hz, 2H, $H_{e'}$).

Diffusion coefficient (500 MHz, d_6-DMSO) D: 1.13 × 10^{-10} m^2s^{-1}; R_H: 8.86 Å.

^{13}C NMR (126 MHz, d_6-DMSO) δ: 163.2, 162.2, 155.1 (C_f/C_q), 155.1 (C_f/C_q), 152.4 (C_a), 151.2 (C_b), 150.9 (C_o), 146.4, 145.4 (C_r), 145.2, 141.9, 141.7 (C_g), 141.4 (C_d/C_i/C_j/C_k/C_l), 140.6 (C_n), 135.9, 134.3 (C_i/C_j/C_k/C_l), 134.1 (C_t/C_u), 133.8 (C_d/C_i/C_j/C_k/C_l), 132.8 (C_i/C_j/C_k/C_l), 132.3 (C_i/C_j/C_k/C_l), 131.9 (C_h), 131.4 (C_s), 130.9 (C_m), 129.8 (C_t/C_u), 128.4, 128.1, 127.2 (C_v), 126.5 (C_c), 125.2, 122.8, 122.1, 100.3, 97.0, 91.4, 90.8, 65.4 (C_e/C_p), 65.3 (C_e/C_p) (1 peak missing due to overlap).

ESI-MS m/z = 491.98 $\{[Pd_2(\mathbf{L^{P3Q}})_2](NO_3)\}^{3+}$ calc. 492.05; 768.97 $\{[Pd_2(\mathbf{L^{P3Q}})_2](NO_3)_2\}^{2+}$ calc. 769.07.

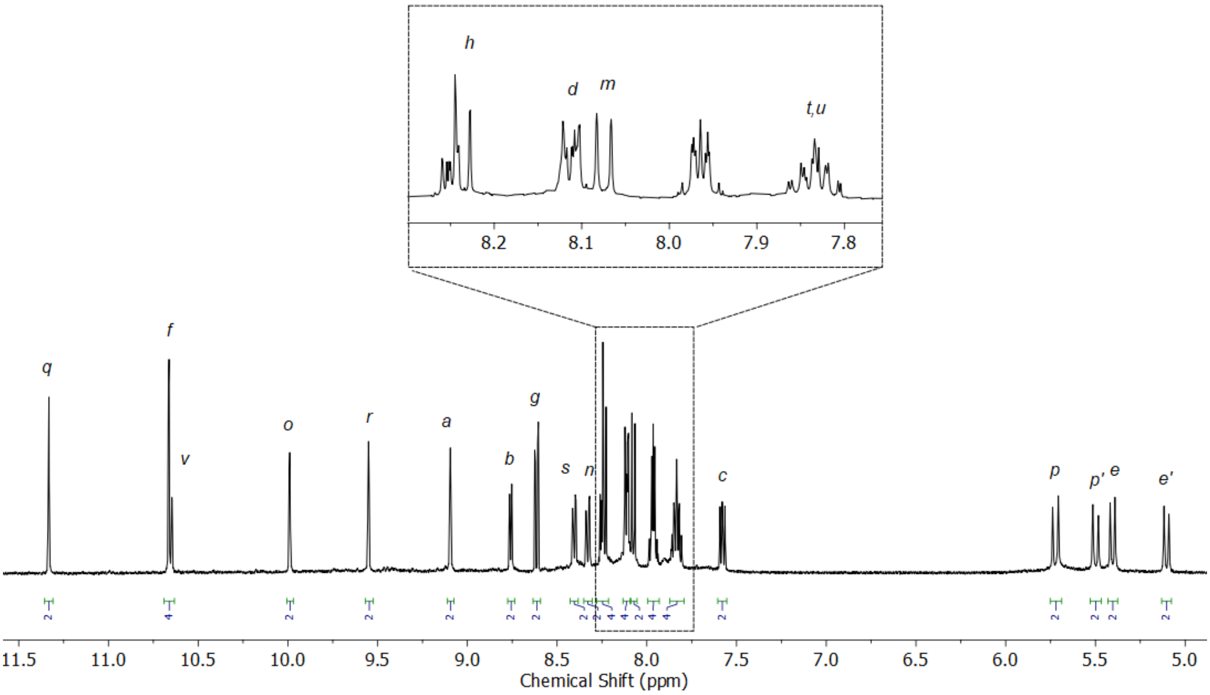

Figure S48 ^{1}H NMR (500 MHz, d_6-DMSO) of $[Pd_2(\mathbf{L}^{\mathbf{P3Q}})_2](NO_3)_4$.

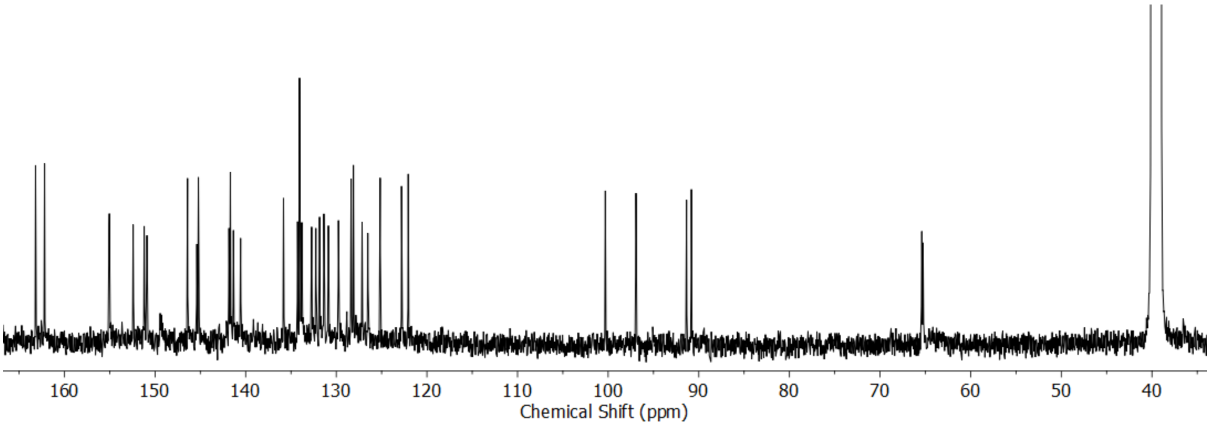

Figure S49 ^{13}C NMR (126 MHz, d_6-DMSO) of $[Pd_2(\mathbf{L}^{\mathbf{P3Q}})_2](NO_3)_4$.

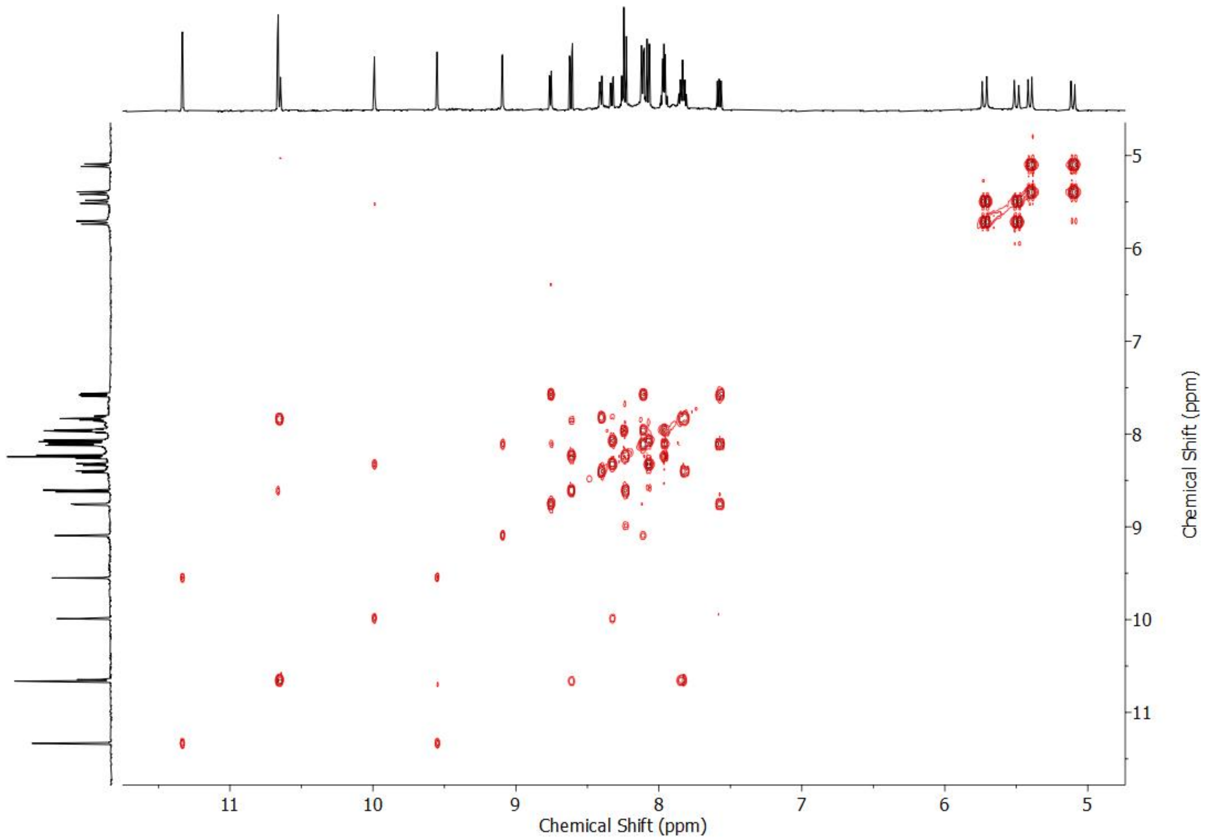

Figure S50 COSY NMR (d_6-DMSO) of $[Pd_2(\mathbf{L^{P3Q}})_2](NO_3)_4$.

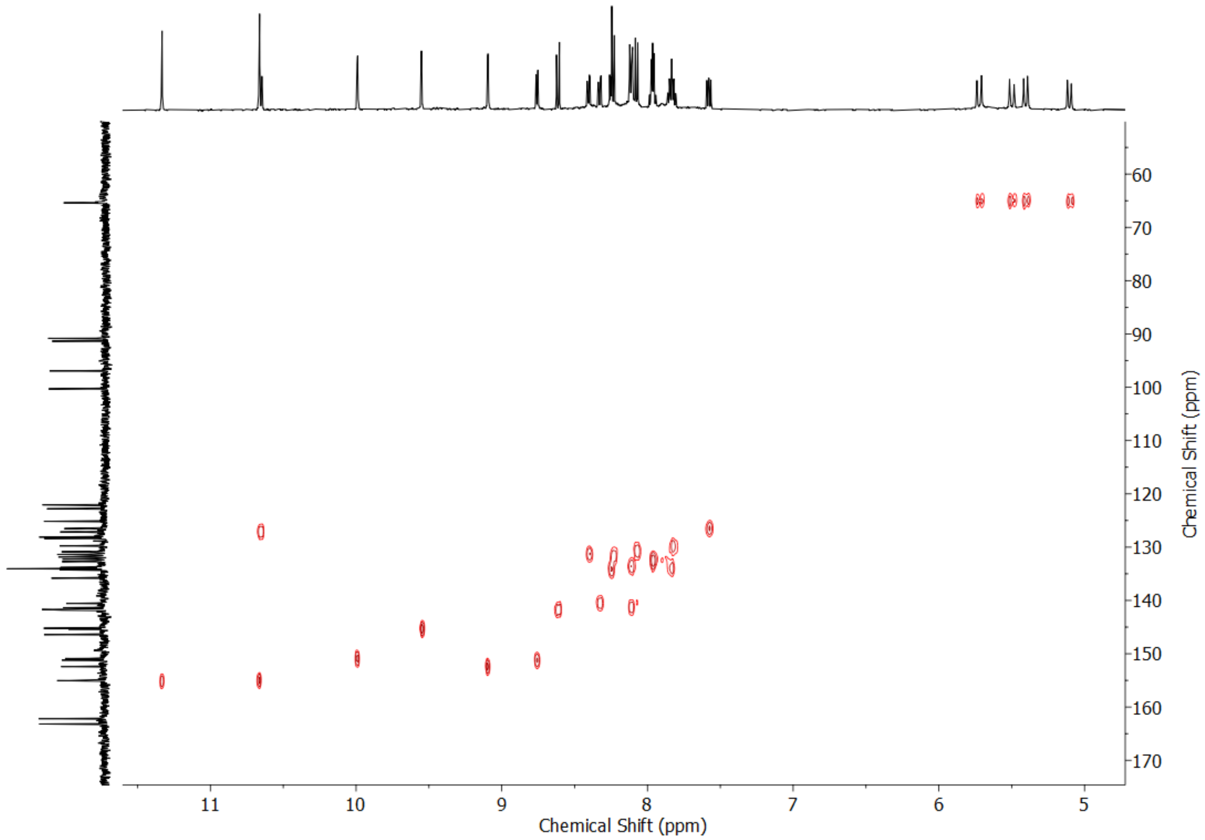

Figure S51 HSQC NMR (d_6-DMSO) of $[Pd_2(\mathbf{L^{P3Q}})_2](NO_3)_4$.

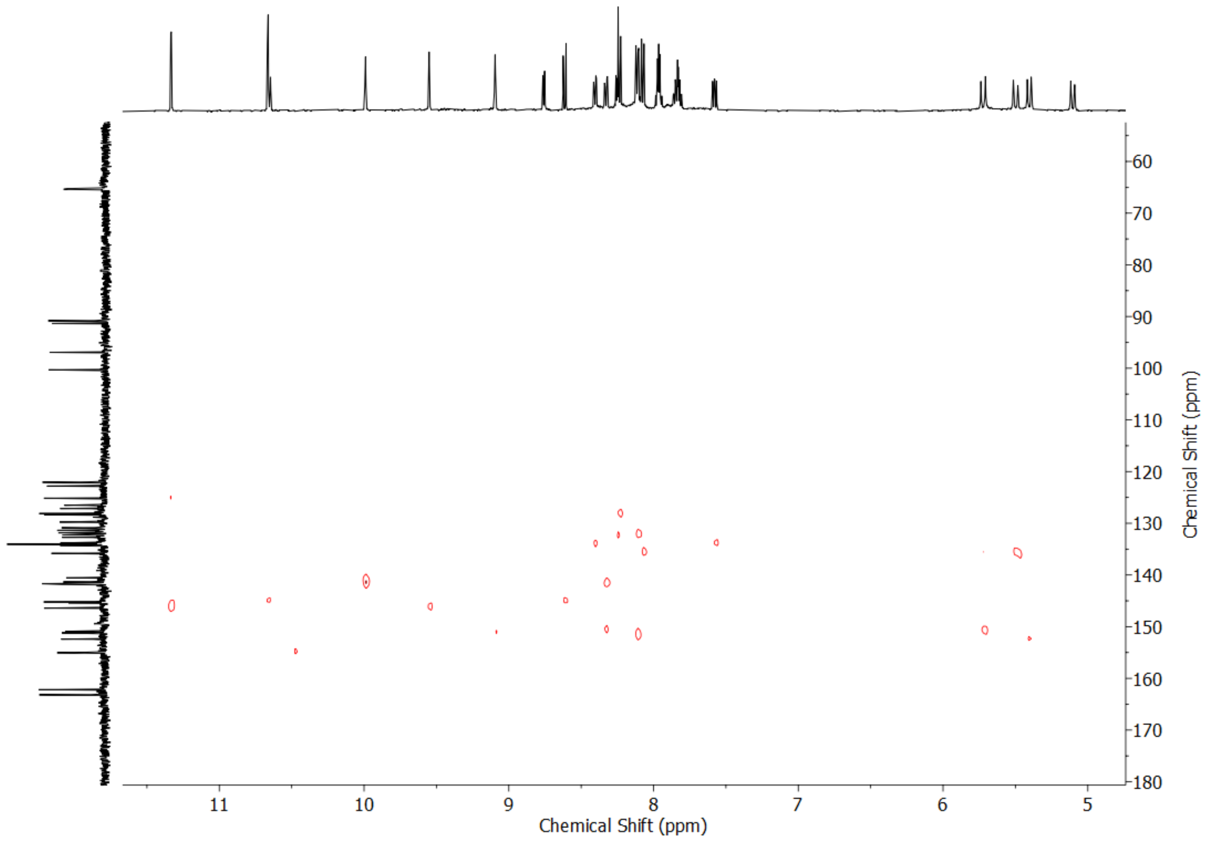

Figure S52 HMBC NMR (d_6-DMSO) of $[Pd_2(\mathbf{L^{P3Q}})_2](NO_3)_4$.

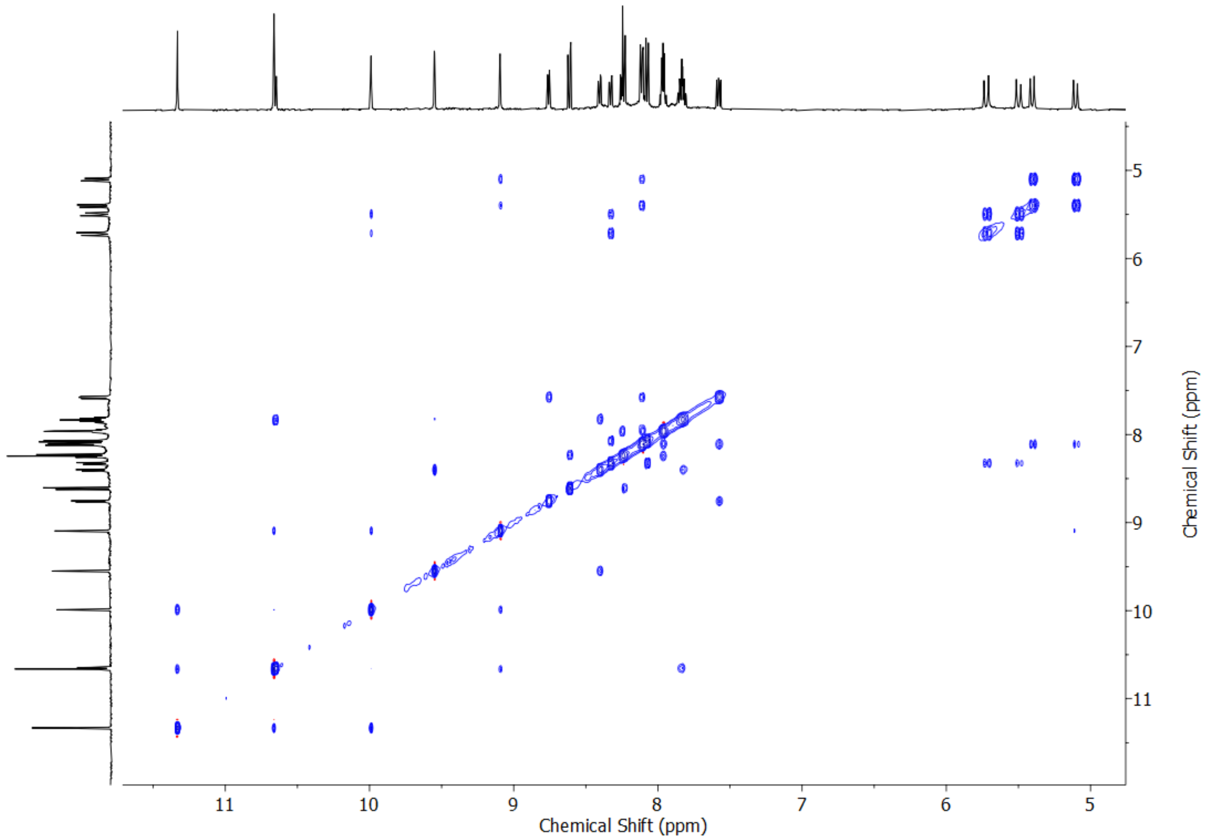

Figure S53 NOESY (d_6-DMSO) of $[Pd_2(\mathbf{L^{P3Q}})_2](NO_3)_4$.

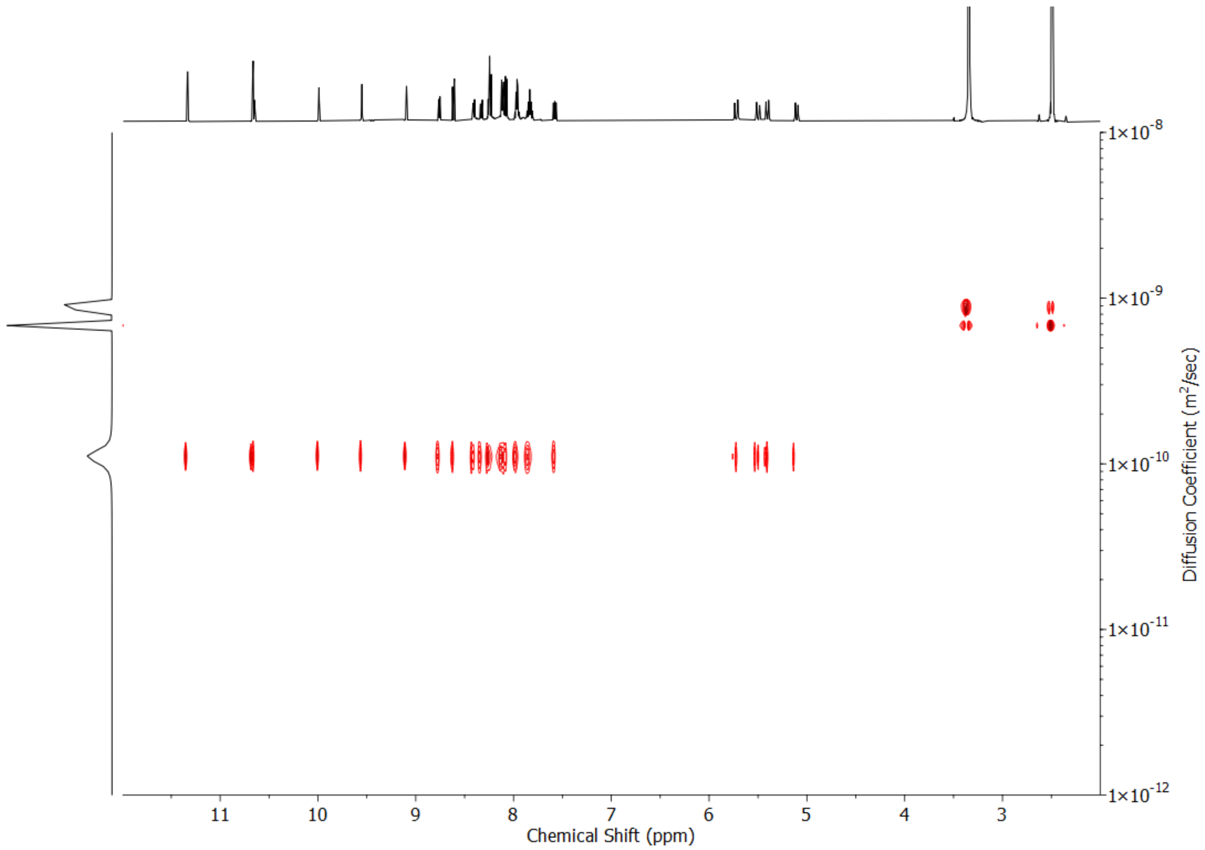

Figure S54 DOSY (d_6-DMSO) of $[Pd_2(\mathbf{L}^{P3Q})_2](NO_3)_4$.

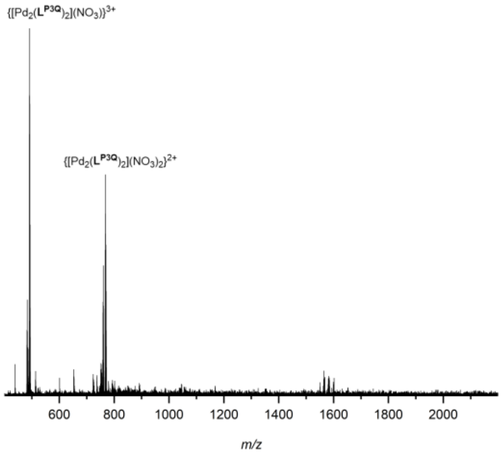

Figure S55 ESI-MS of $[Pd_2(\mathbf{L}^{P3Q})_2](NO_3)_4$.

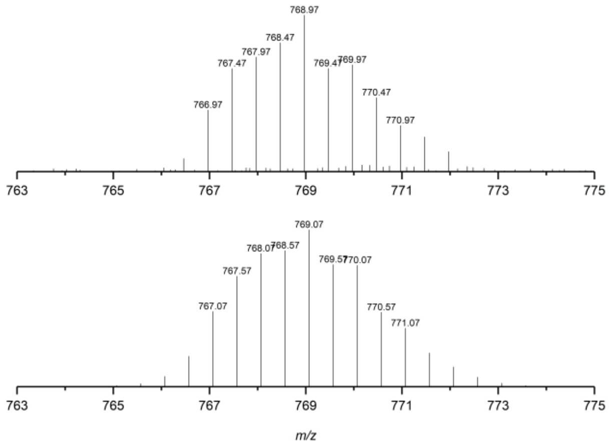

Figure S56 Observed (top) and calculated (bottom) isotopic patterns for $\{[Pd_2(\mathbf{L}^{\mathbf{P3Q}})_2](NO_3)_2\}^{2+}$.

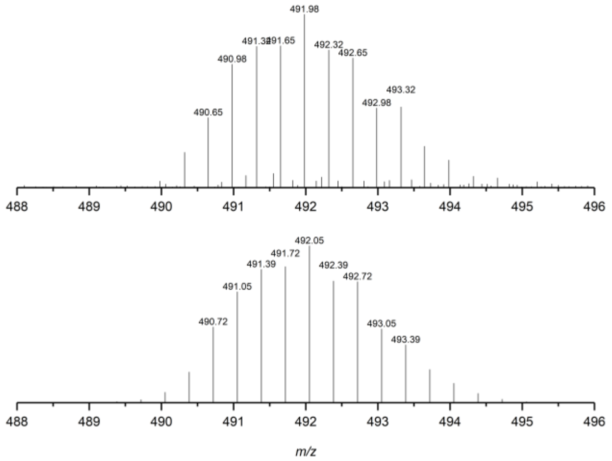

Figure S57 Observed (top) and calculated (bottom) isotopic patterns for $\{[Pd_2(\mathbf{L}^{\mathbf{P3Q}})_2](NO_3)\}^{3+}$.

Synthesis of S7

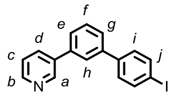

(3-(Pyridin-3-yl)phenyl)boronic acid (0.597 g, 3 mmol, 1 eq.), 1,4-diiodobenzene (1.93 g, 4.5 mmol, 1.5 eq.), $Pd(PPh_3)_2Cl_2$ (0.053 g, 0.075 mmol, 2.5 mol%) and K_2CO_3 (1.04 g, 7.5 mmol, 2.5 eq.) were stirred at 90 °C in 2:1 dioxane/H_2O (9 mL) in a sealed vial for 24 h. H_2O (20 mL) was added and the aqueous phase extracted with CH_2Cl_2 (3 × 20 mL). The combined organic phases were dried ($MgSO_4$) and the solvent removed *in vacuo*. After purification by column chromatography (5% acetone in 1:1 CH_2Cl_2/pentane) the product was obtained as an orange solid (0.265 g, 25%).

^{1}H NMR (400 MHz, $CDCl_3$) δ: 8.89 (d, J = 1.6 Hz, 1H, H_a), 8.62 (dd, J = 4.8, 1.5 Hz, 1H, H_b), 7.92 (ddd, J = 7.9, 2.2, 1.7 Hz, 1H, H_d), 7.80 (d, J = 8.5 Hz, 2H, H_i/H_j), 7.73 (s, 1H, H_h), 7.60-7.53 (m, 3H, H_e, H_f, H_g), 7.41-7.37 (m, 3H, H_c, H_i/H_j).

^{13}C NMR (101 MHz, $CDCl_3$) δ: 148.8 (C_b), 148.5 (C_a), 141.2, 140.4, 138.7, 138.1 (C_i/C_j), 136.6, 134.6 (C_d), 129.8 (C_e/C_f/C_g), 129.2 (C_i/C_j), 126.8 (C_e/C_f/C_g), 126.6 (C_e/C_f/C_g), 125.9 (C_h), 123.8 (C_c), 93.6.

HR-ESI-MS m/z = 358.0081 $[M+H]^+$ calc. 358.0093.

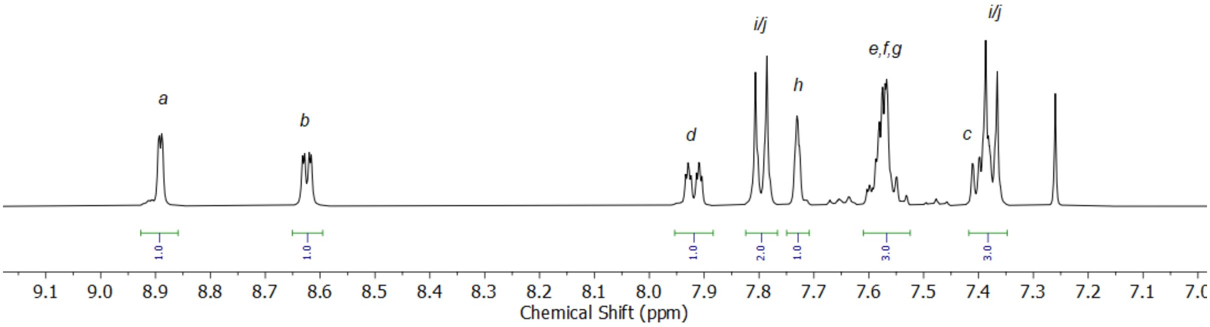

Figure S58 ^{1}H NMR (400 MHz, $CDCl_3$) of **S7**.

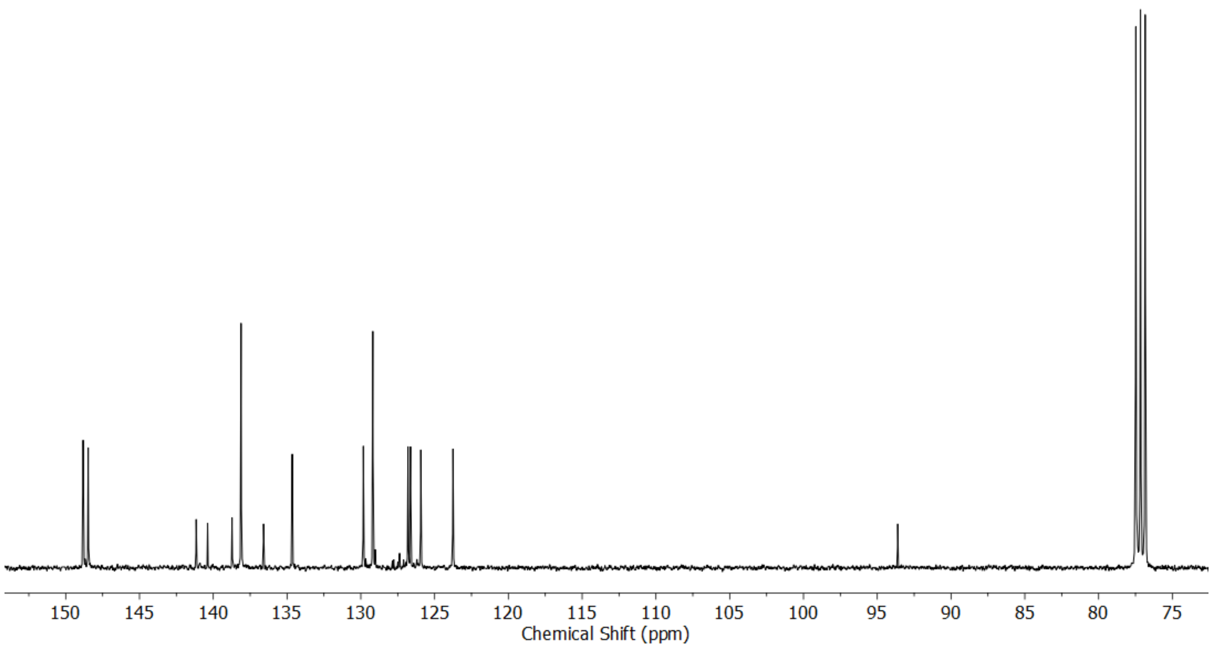

Figure S59 ^{13}C NMR (101 MHz, $CDCl_3$) of **S7**.

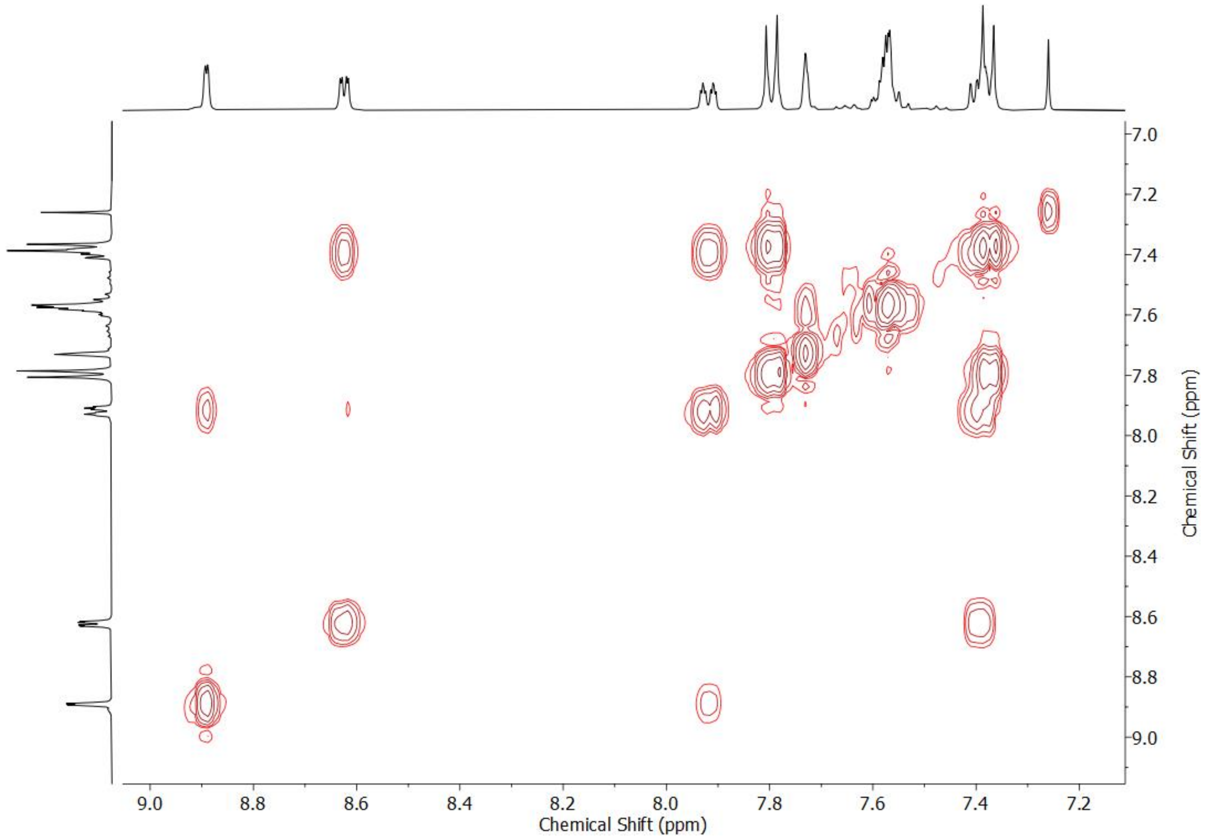

Figure S60 COSY NMR ($CDCl_3$) of **S7**.

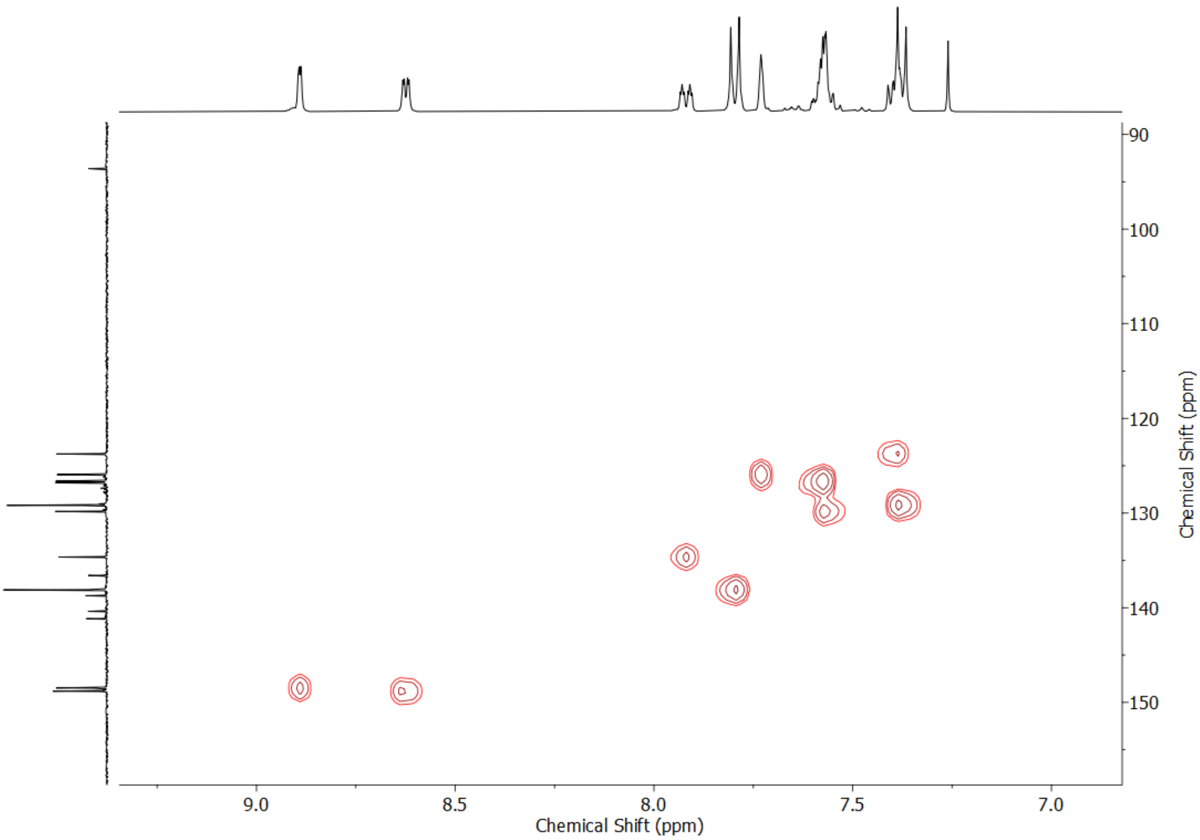

Figure S61 HSQC NMR ($CDCl_3$) of **S7**.

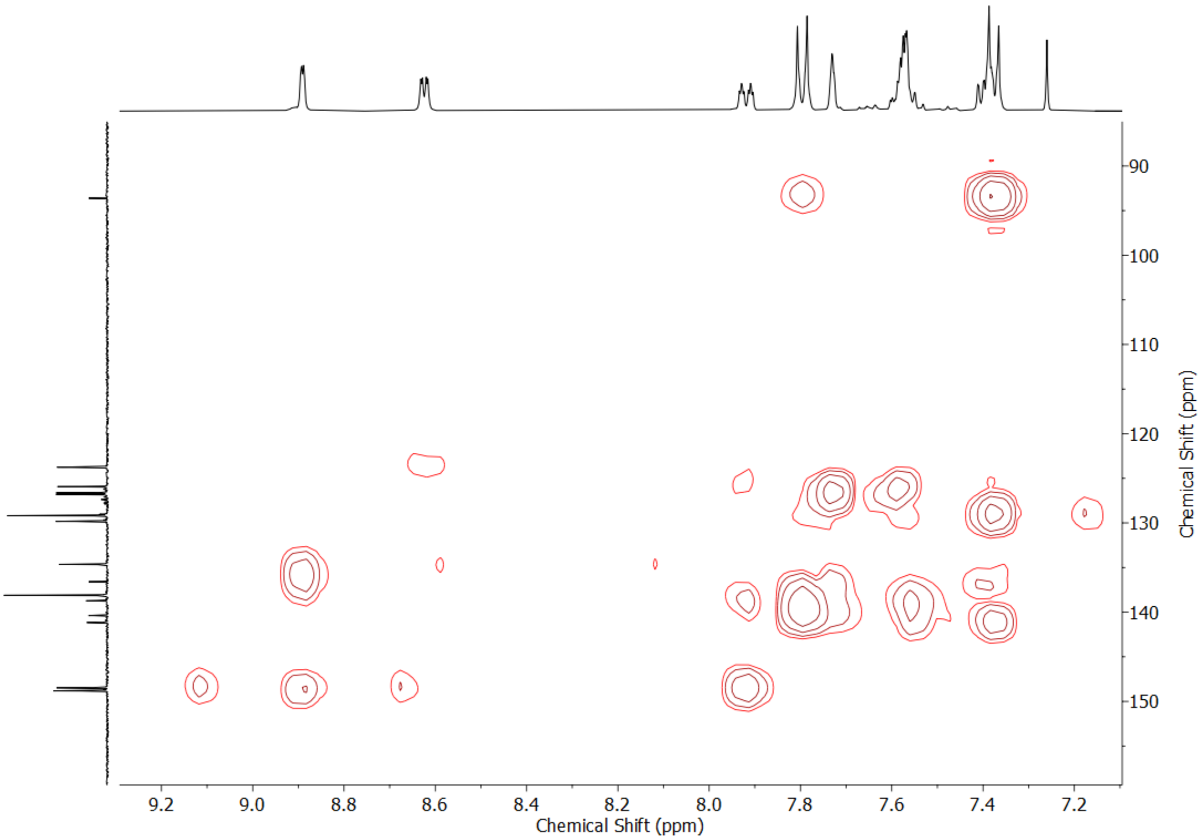

Figure S62 HMBC NMR ($CDCl_3$) of **S7**.

Synthesis of L1^{Ph}

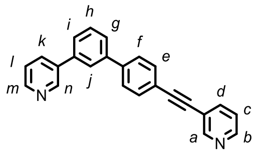

S7 (0.107 g, 0.3 mmol, 1 eq.), 3-ethynylpyridine (0.034 g, 0.33 mmol, 1.1 eq.), $Pd(PPh_3)_2Cl_2$ (0.011 g, 0.015 mmol, 5 mol%) and CuI (0.0029 g, 0.015 mmol, 5 mol%) were stirred at rt in 1:1 $^iPr_2NH$/dioxane (3 mL) for 20 h. EDTA solution (20 mL) was added and the aqueous phase extracted with CH_2Cl_2 (3 × 20 mL). The combined organic phases were dried ($MgSO_4$) and the solvent removed *in vacuo*. After purification by column chromatography on silica (pentane with step gradient 0 to 70% EtOAc in 10% increments) the product was obtained as an orange solid (0.090 g, 90%).

^{1}H NMR (400 MHz, $CDCl_3$) δ: 8.91 (d, J = 1.7 Hz, 1H, H_n), 8.79 (dd, J = 2.2, 0.9 Hz, 1H, H_a), 8.63 (dd, J = 4.8, 1.5 Hz, 1H, H_m), 8.56 (dd, J = 4.9, 1.6 Hz, 1H, H_b), 7.94 (dt, J = 7.9, 1.9 Hz, 1H, H_k), 7.83 (dt, J = 7.9, 1.9 Hz, 1H, H_d), 7.80 (s, 1H, 1 of H_j), 7.66-7.64 (m, 5H, H_e, H_f, 1 of H_g/H_h/H_i), 7.59-7.57 (m, 2H, 2 of H_g/H_h/H_i), 7.40 (dd, J = 7.9, 4.8, 0.9 Hz, 1H, H_l), 7.30 (ddd, J = 7.9, 4.9, 0.9 Hz, 1H, H_c).

^{13}C NMR (101 MHz, $CDCl_3$) δ: 152.4 (C_a), 148.9 (C_m), 148.8 (C_b), 148.5 (C_n), 141.3, 141.2, 138.7, 138.6 (C_d), 136.6, 134.6 (C_k), 132.4 (C_e/C_f), 129.8 (C_g/C_h/C_i), 127.4 (C_e/C_f), 126.9 (C_g/C_h/C_i), 126.7 (C_g/C_h/C_i), 126.1 (C_j), 123.8 (C_l), 123.2 (C_c), 121.9, 120.6, 92.6, 87.0.

HR-ESI-MS m/z = 333.1396 $[M+H]^+$ calc. 333.1392.

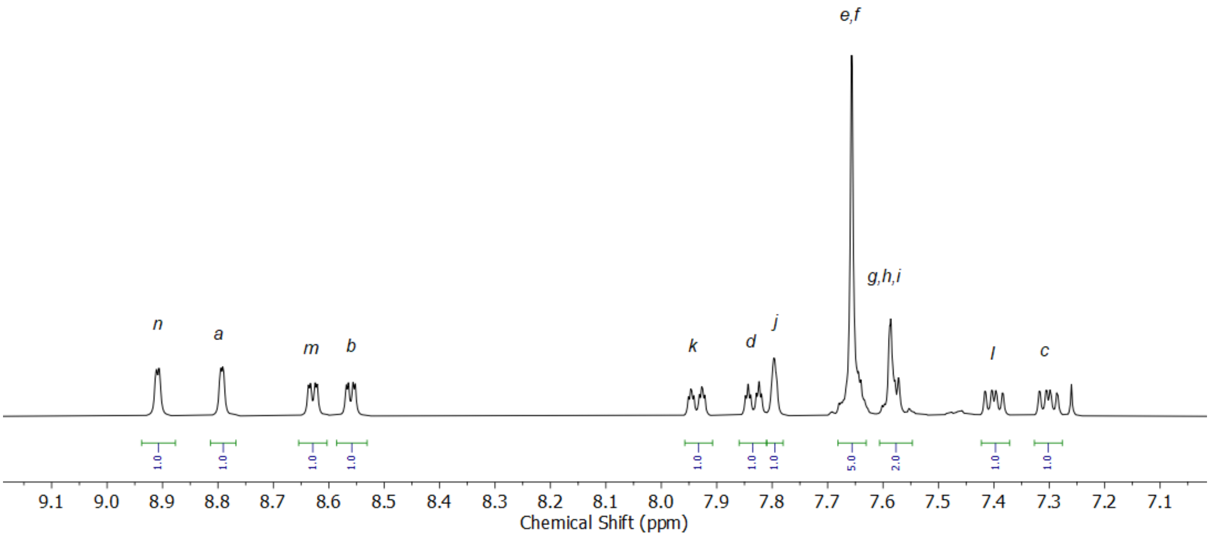

Figure S63 ^{1}H NMR (400 MHz, $CDCl_3$) of **L1^{Ph}**.

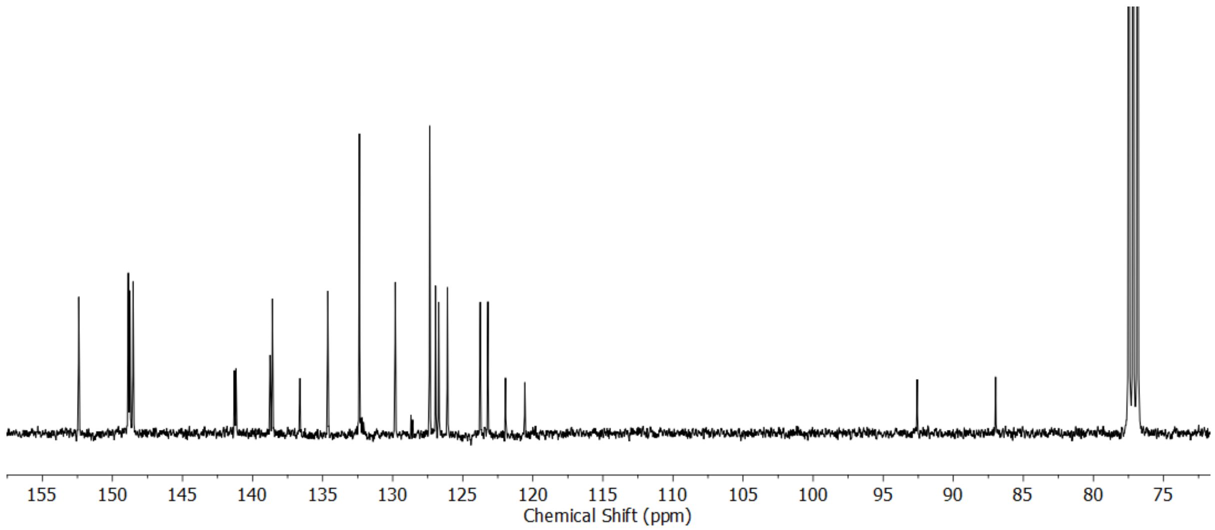

Figure S64 ^{13}C NMR (101 MHz, $CDCl_3$) of **L1^{Ph}**.

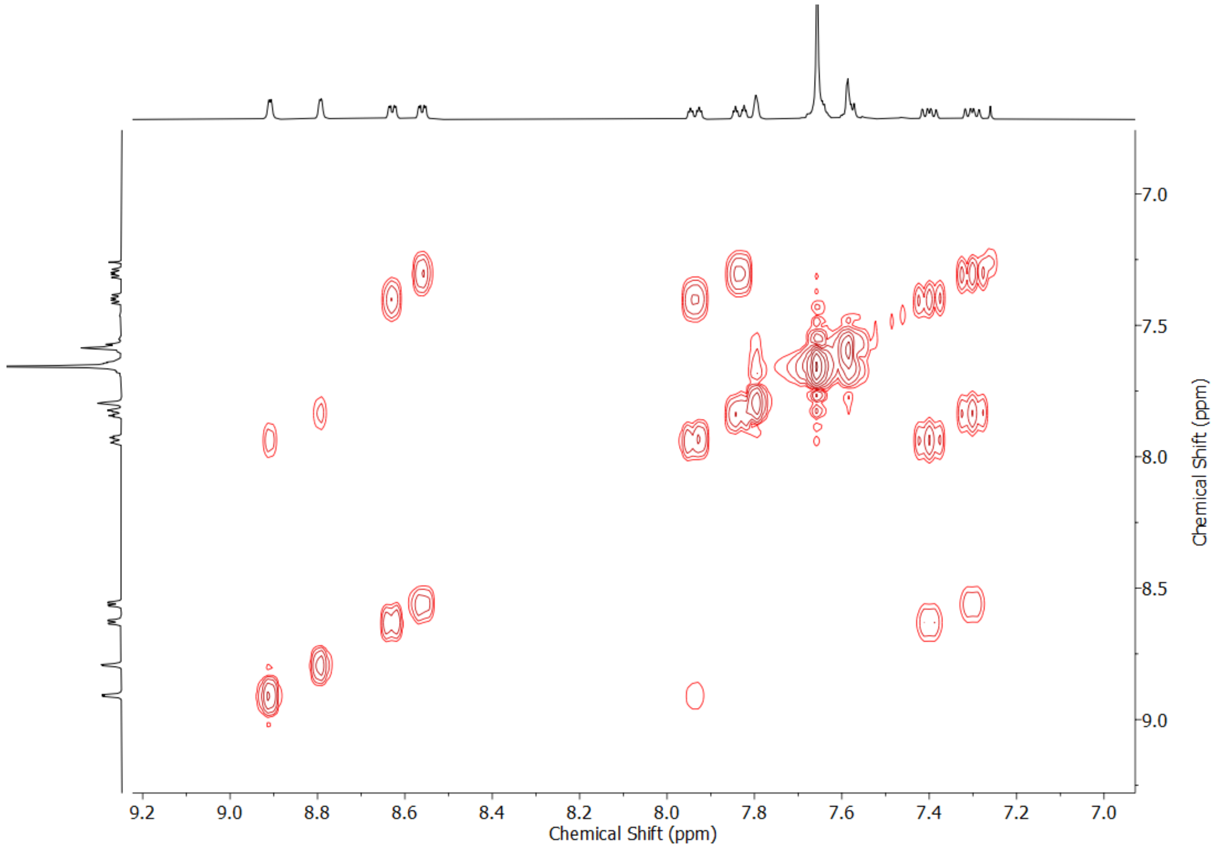

Figure S65 COSY NMR ($CDCl_3$) of **L1^{Ph}**.

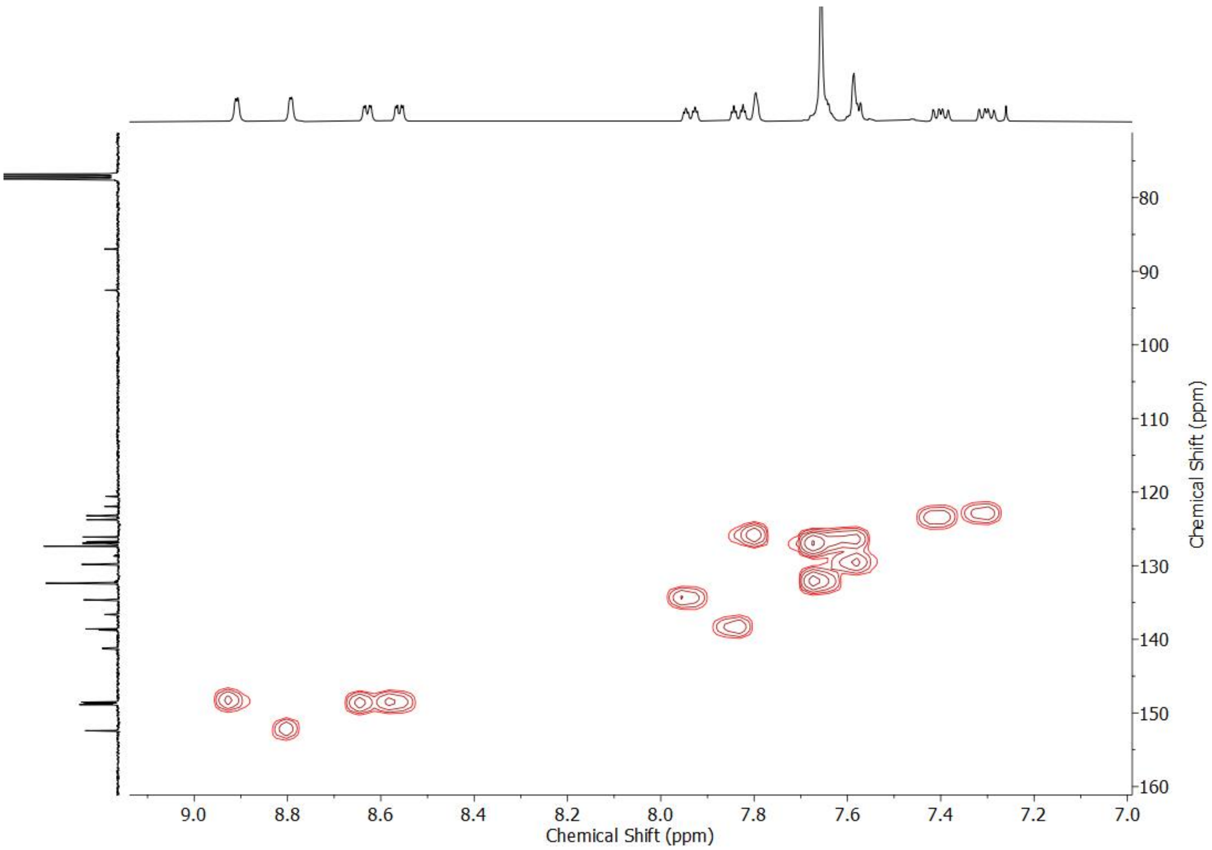

Figure S66 HSQC NMR ($CDCl_3$) of **L1^{Ph}**.

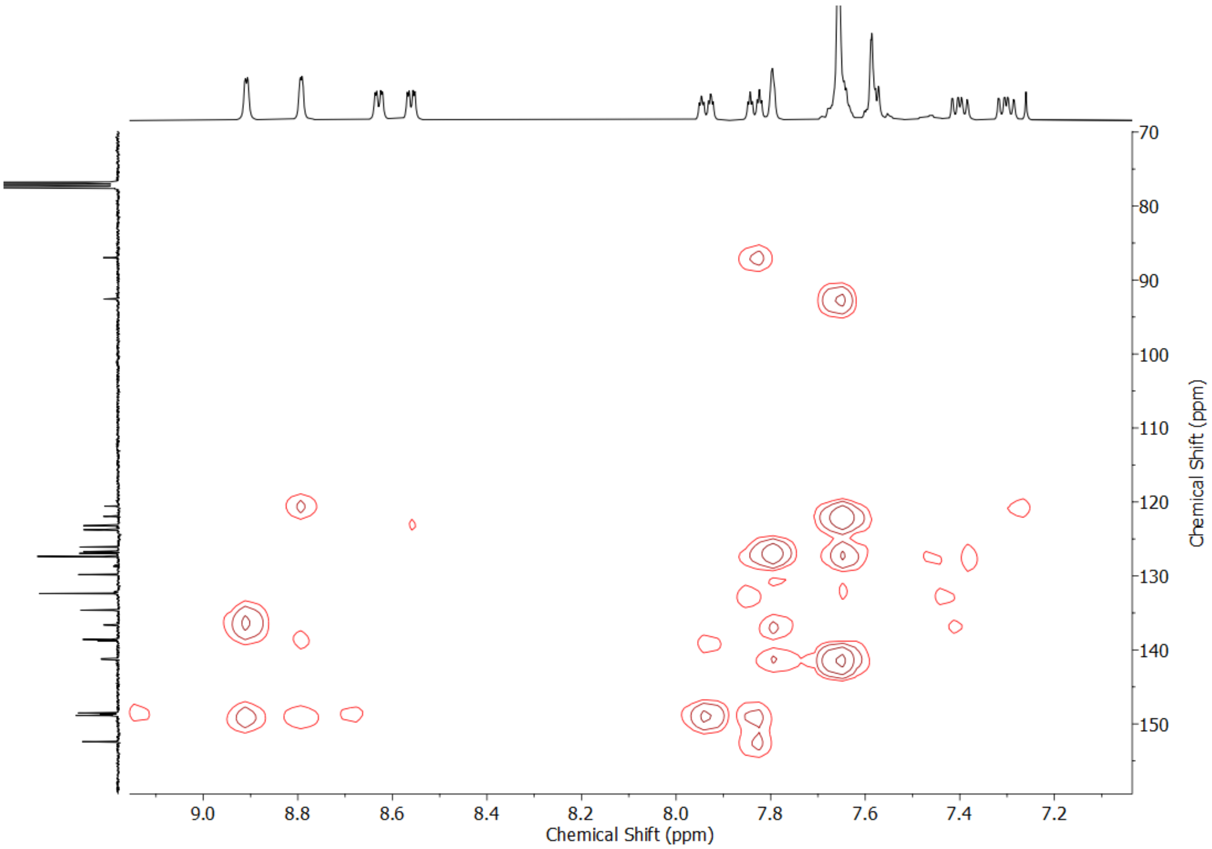

Figure S67 HMBC NMR ($CDCl_3$) of **L1^{Ph}**.

Synthesis of *cis*-$[Pd_2(L1^{Ph})_4](BF_4)_4$ ($C1^{Ph}$)

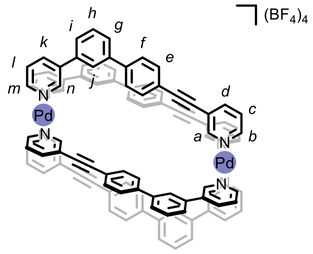

L1^{Ph} (10.0 mg, 30 µmol, 2 eq.) and $[Pd(CH_3CN)_4](BF_4)_2$ (6.7 mg, 15 µmol, 1 eq.) were sonicated in d_6-DMSO (0.75 mL) until a homogenous solution was obtained. After standing at rt for 1 d, ^{1}H NMR demonstrated quantitative conversion to **C1^{Ph}**. The solution was diluted with MeCN (0.75 mL), filtered through celite, and left for vapour diffusion of Et_2O. After 7 d the mother liquor was carefully decanted and the precipitate washed with Et_2O and dried *in vacuo* to give the product as a yellow solid (12.1 mg, 85%).

^{1}H NMR (400 MHz, d_6-DMSO) δ: 9.83 (s, 4H, H_a), 9.47 (s, 4H, H_n), 9.41 (d, J = 5.5 Hz, 4H, H_m), 9.32 (d, J = 5.4 Hz, 4H, H_b), 8.55 (d, J = 8.2 Hz, 4H, H_k), 8.31 (s, 4H, H_j), 8.23 (d, J = 8.0 Hz, 4H, H_d), 8.05 (d, J = 7.9 Hz, 8H, H_f), 7.92 (dd, J = 8.0, 5.8 Hz, 4H, H_l), 7.83-7.76 (m, 20H, H_c, H_e, H_g, H_i), 7.61 (app. t, J = 7.7 Hz, 4H, H_h).

Diffusion coefficient (400 MHz, d_6-DMSO) D: 9.99 × 10^{-11} m^2s^{-1}; R_H: 10.0 Å.

^{13}C NMR (101 MHz, d_6-DMSO) δ: 153.2 (C_a), 150.3 (C_b), 149.8 (C_m), 148.4 (C_n), 142.1 (C_d), 140.7, 139.9, 138.8 (C_k), 137.9, 134.5, 132.2 (C_e), 130.4 (C_h), 128.1 ($C_c/C_g/C_i/C_l$), 127.7 (C_f), 127.5 ($C_c/C_g/C_i/C_l$), 127.2 ($C_c/C_g/C_i/C_l$), 127.1 ($C_c/C_g/C_i/C_l$), 124.4 (C_j), 122.6, 120.3, 94.6, 84.8.

ESI-MS m/z = 857.09 $\{[Pd_2(\mathbf{L1^{Ph}})_4](BF_4)_2\}^{2+}$ calc. 857.17; 1762.16 $\{[Pd_2(\mathbf{L1^{Ph}})_4](BF_4)_2(HCO_2)\}^{+}$ calc. 1762.34.

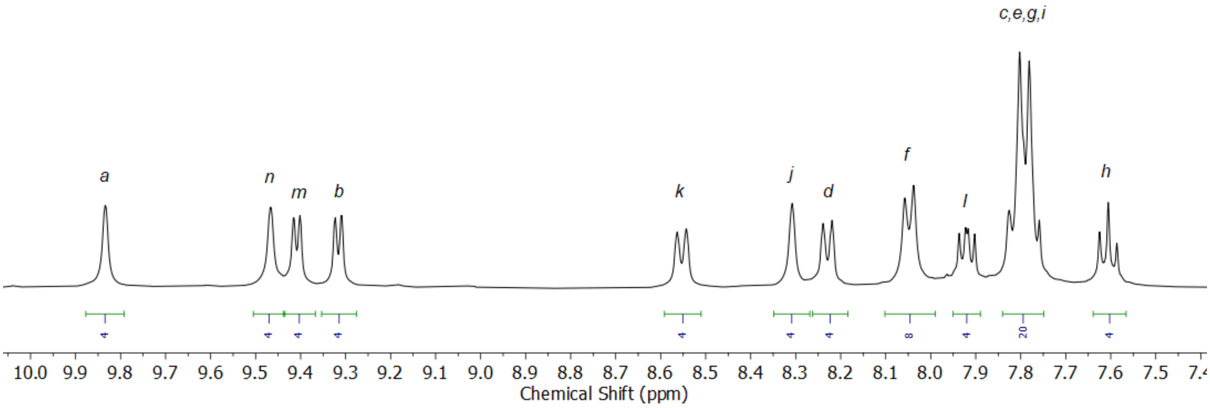

Figure S68 ^{1}H NMR (400 MHz, d_6-DMSO) of $[Pd_2(\mathbf{L1^{Ph}})_2](BF_4)_4$.

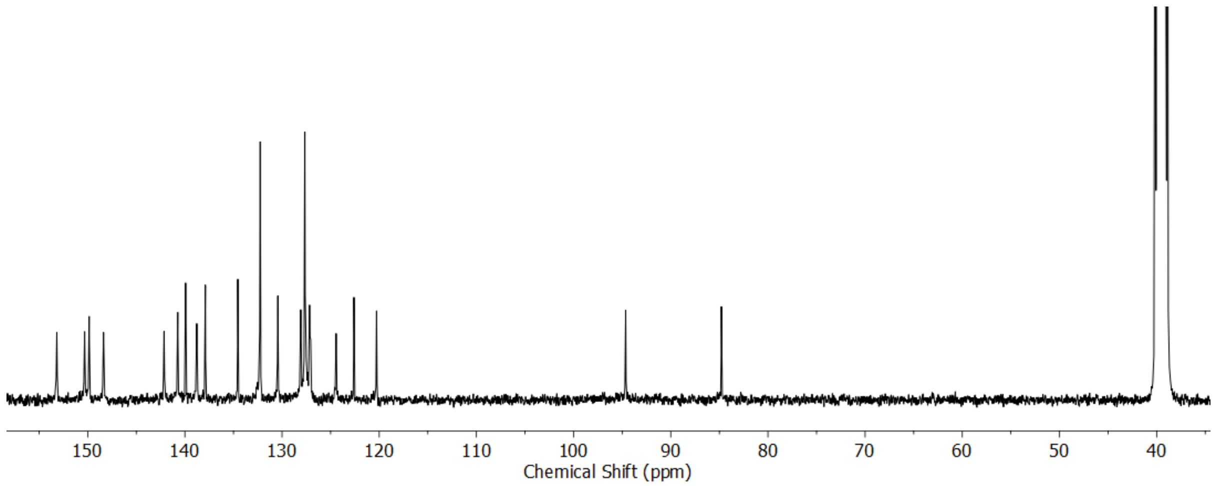

Figure S69 ^{13}C NMR (101 MHz, d_6-DMSO) of $[Pd_2(\mathbf{L1^{Ph}})_2](BF_4)_4$.

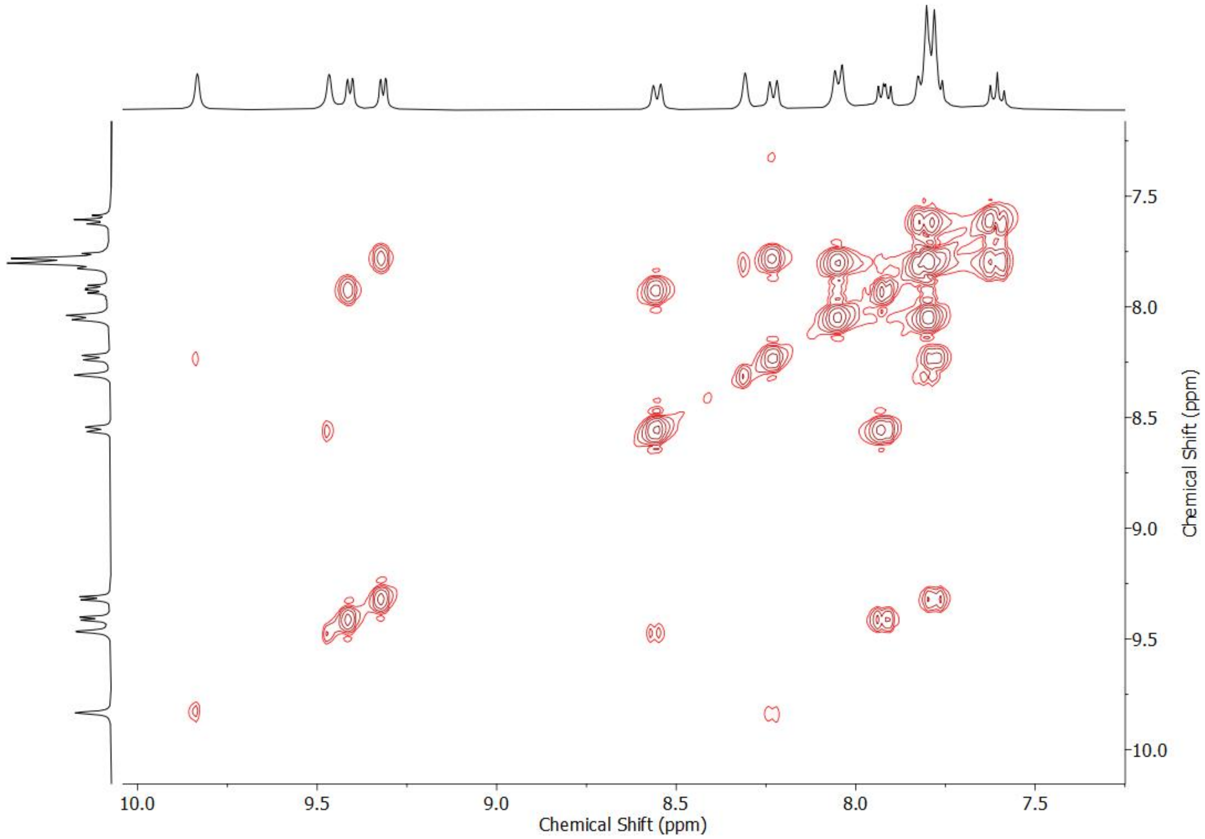

Figure S70 COSY NMR (d_6-DMSO) of $[Pd_2(\mathbf{L1^{Ph}})_2](BF_4)_4$.

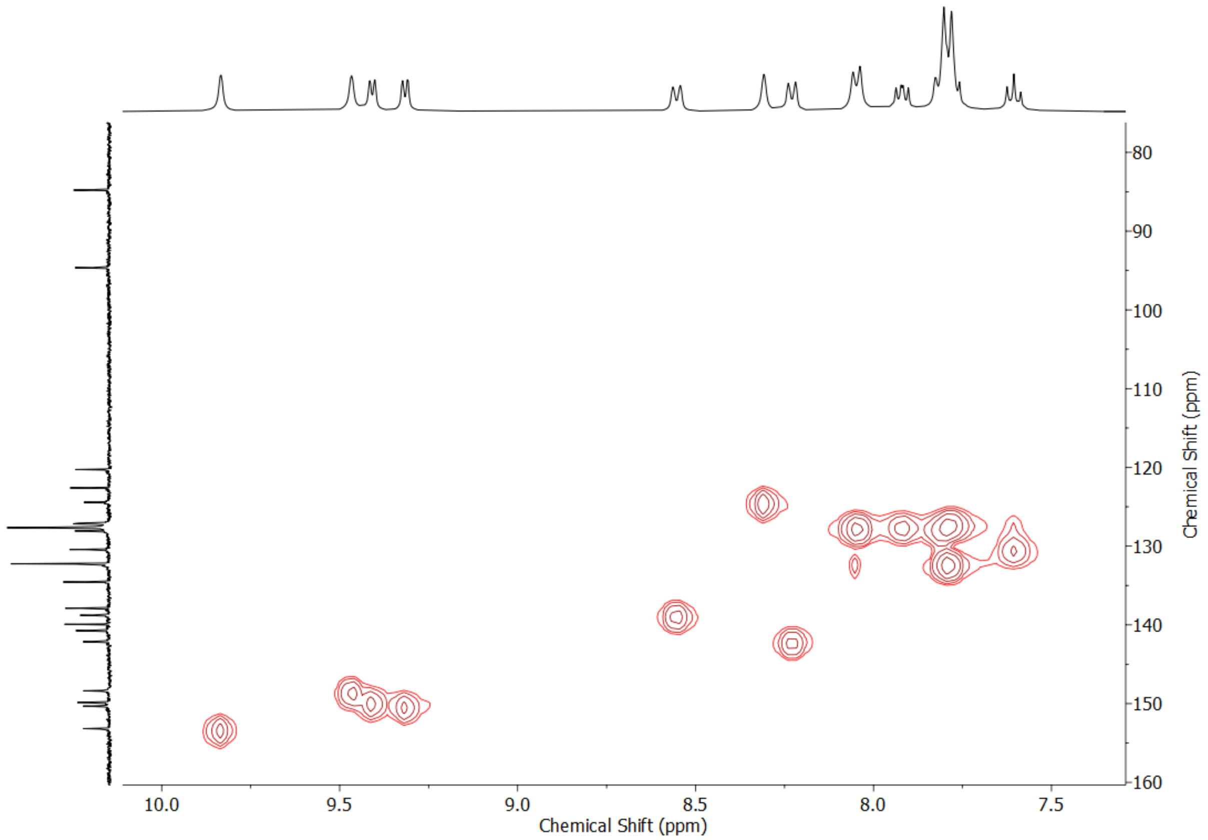

Figure S71 HSQC NMR (d_6-DMSO) of [Pd_2(**L1**$^{\mathbf{Ph}}$)$_2$](BF_4)$_4$.

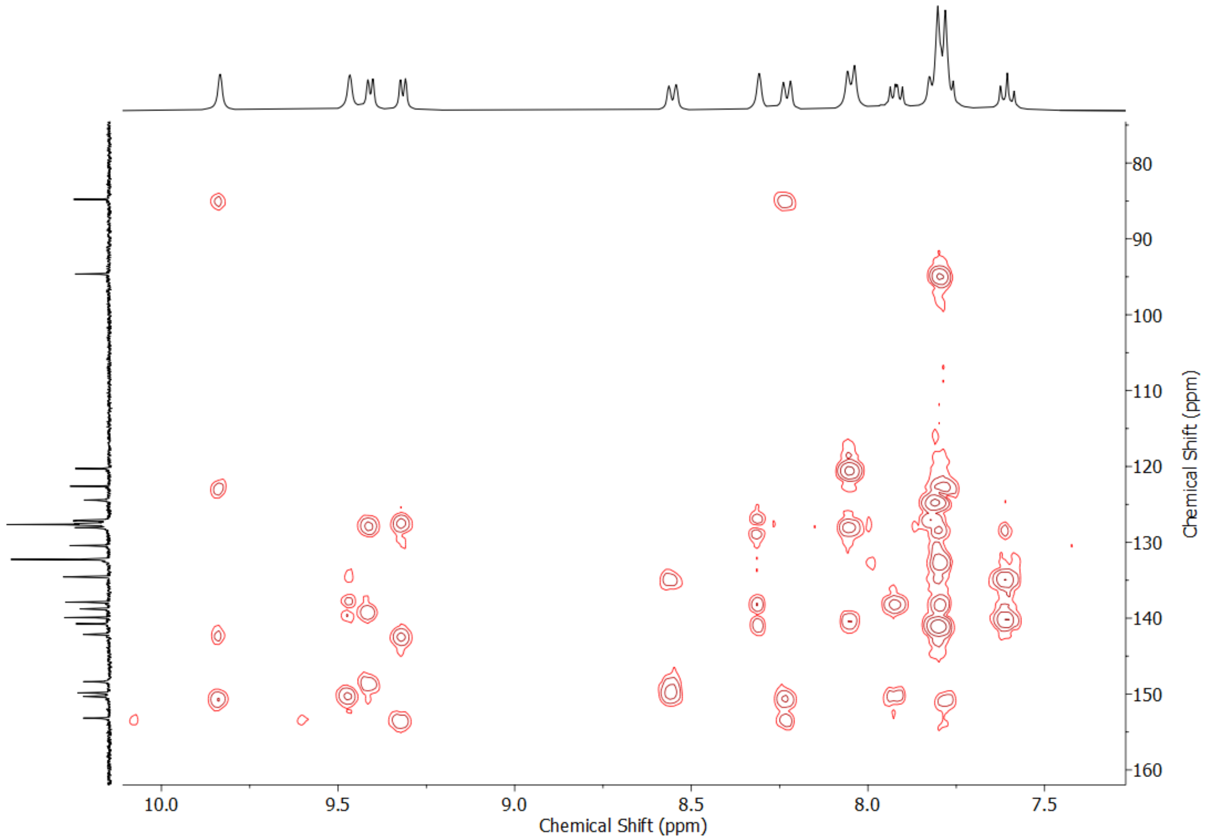

Figure S72 HMBC NMR (d_6-DMSO) of [Pd_2(**L1**$^{\mathbf{Ph}}$)$_2$](BF_4)$_4$.

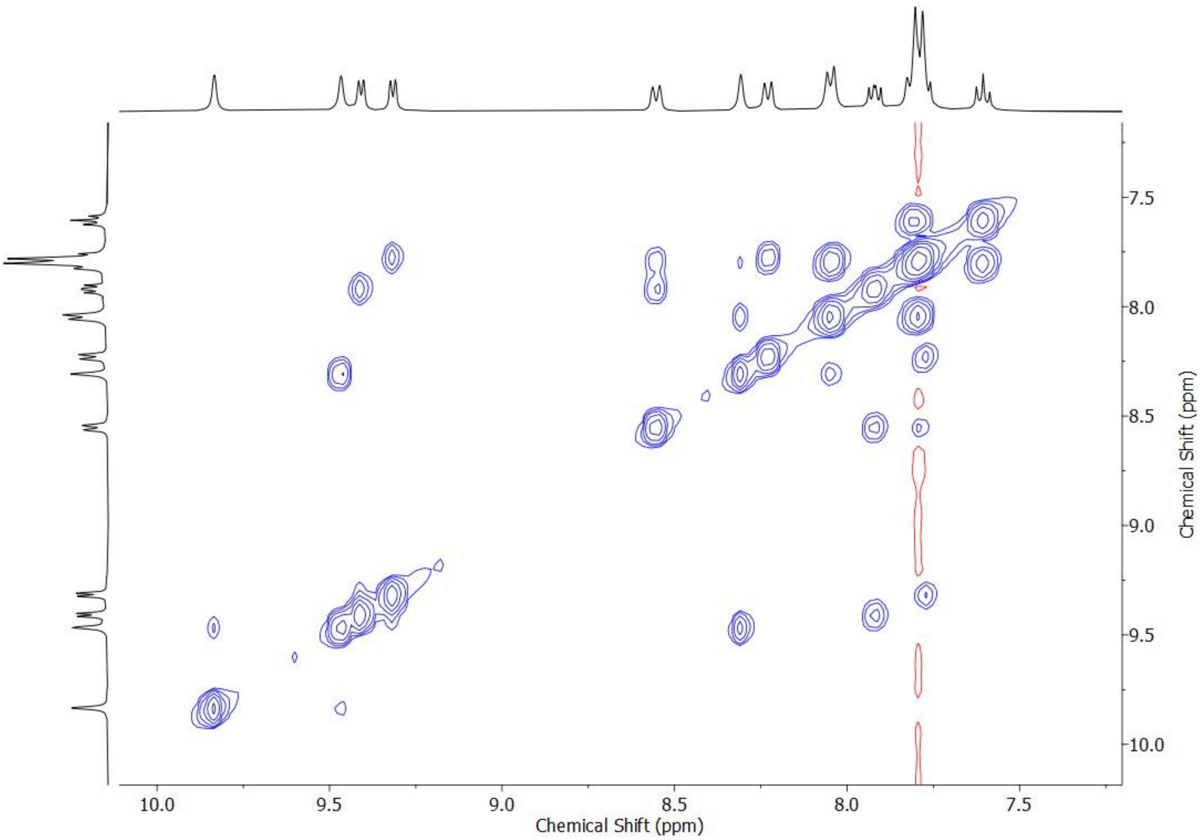

Figure S73 NOESY (d_6-DMSO) of $[Pd_2(\mathbf{L1}^{\mathbf{Ph}})_2](BF_4)_4$.

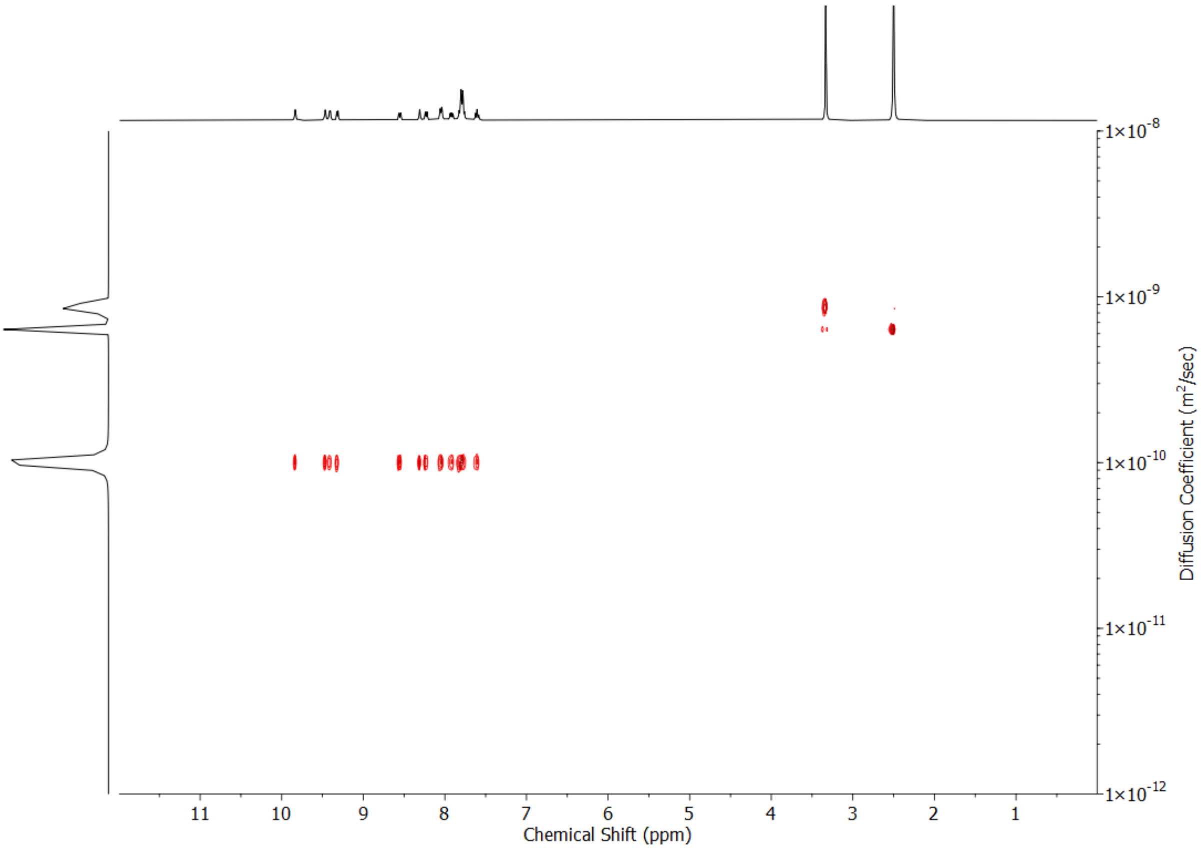

Figure S74 DOSY (d_6-DMSO) of $[Pd_2(\mathbf{L1}^{\mathbf{Ph}})_2](BF_4)_4$.

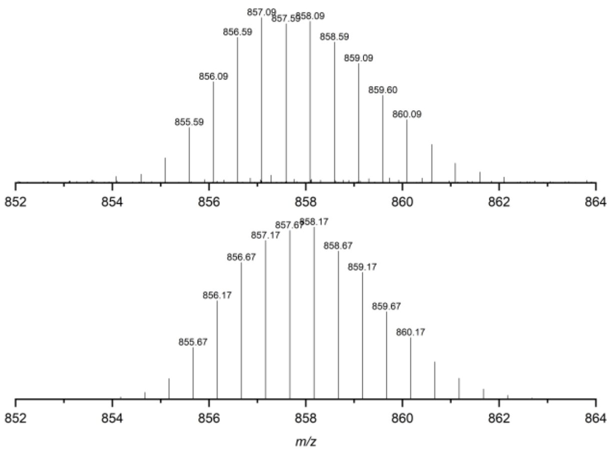

Figure S75 Observed (top) and calculated (bottom) isotopic patterns for $\{[Pd_2(\mathbf{L1}^{\mathbf{Ph}})_4](BF_4)_2\}^{2+}$.

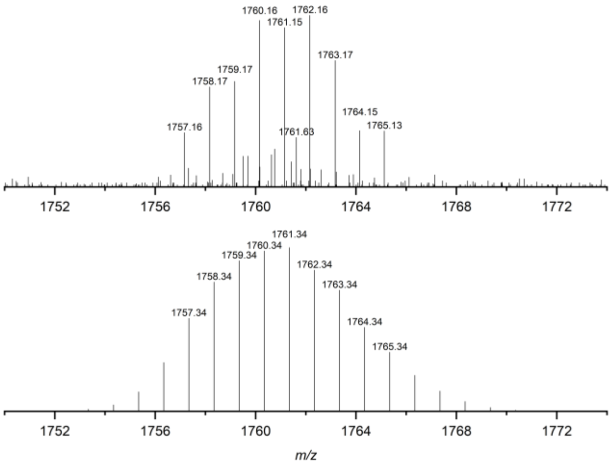

Figure S76 Observed (top) and calculated (bottom) isotopic patterns for $\{[Pd_2(\mathbf{L1}^{\mathbf{Ph}})_4](BF_4)_2(HCO_2)\}^{+}$.

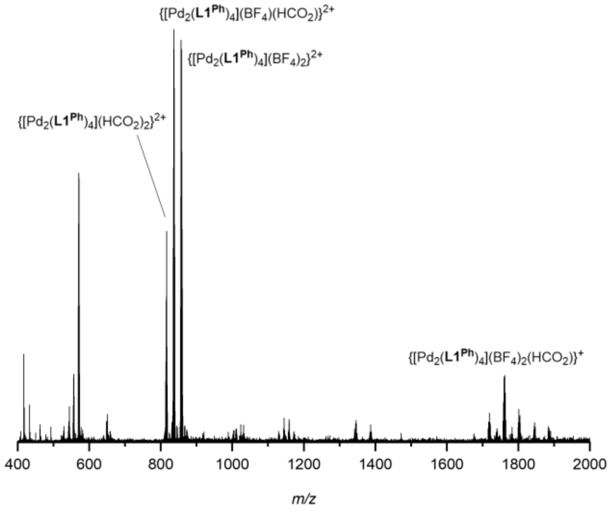

Figure S77 ESI-MS of $[Pd_2(\mathbf{L1}^{\mathbf{Ph}})_2](BF_4)_4$.

Synthesis of S8

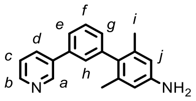

(3-(Pyridin-3-yl)phenyl)boronic acid (0.398 g, 2 mmol, 1 eq.), 4-bromo-3,5-dimethylaniline (0.440 g, 2.2 mmol, 1.1 eq.), $Pd(PPh_3)_2Cl_2$ (0.035 g, 0.05 mmol, 2.5 mol%) and K_2CO_3 (0.691 g, 5 mmol, 2.5 eq.) were stirred at 100 °C in 2:1 dioxane/H_2O (6 mL) in a sealed vial for 18 h. H_2O (20 mL) was added and the aqueous phase extracted with CH_2Cl_2 (3 × 25 mL). The combined organic phases were dried ($MgSO_4$) and the solvent removed *in vacuo*. After purification by column chromatography on silica gel (CH_2Cl_2 with step gradient 0 to 20% acetone in 5% increments) the product was obtained as an orange oil that solidified on standing (0.531 g, 97%).

^{1}H NMR (400 MHz, d_6-DMSO) δ: 8.91 (s, 1H, H_a), 8.56 (d, J = 4.6 Hz, 1H, H_b), 8.10 (d, J = 7.9 Hz, 1H, H_d), 7.65 (d, J = 7.7 Hz, 1H, H_e), 7.52 (app. t, J = 7.7 Hz, 1H, H_f), 7.46 (dd, J = 7.7, 4.9 Hz, 1H, H_c), 7.42 (s, 1H, H_h), 7.14 (d, J = 7.5 Hz, 1H, H_g), 6.34 (s, 2H, H_j), 4.93 (s, 2H, H_{NH}), 1.89 (s, 6H, H_i).

^{13}C NMR (101 MHz, d_6-DMSO) δ: 148.4 (C_b), 147.7 (C_a), 147.4, 142.1, 137.0, 135.6, 135.5, 134.2 (C_d), 129.8 (C_g), 129.1 (C_f), 128.3 (C_h), 124.7 (C_e), 123.9 (C_c), 113.0 (C_j), 20.8 (C_i) (1 signal missing).

HR-ESI-MS m/z = 275.1544 $[M+H]^+$ calc. 275.1548.

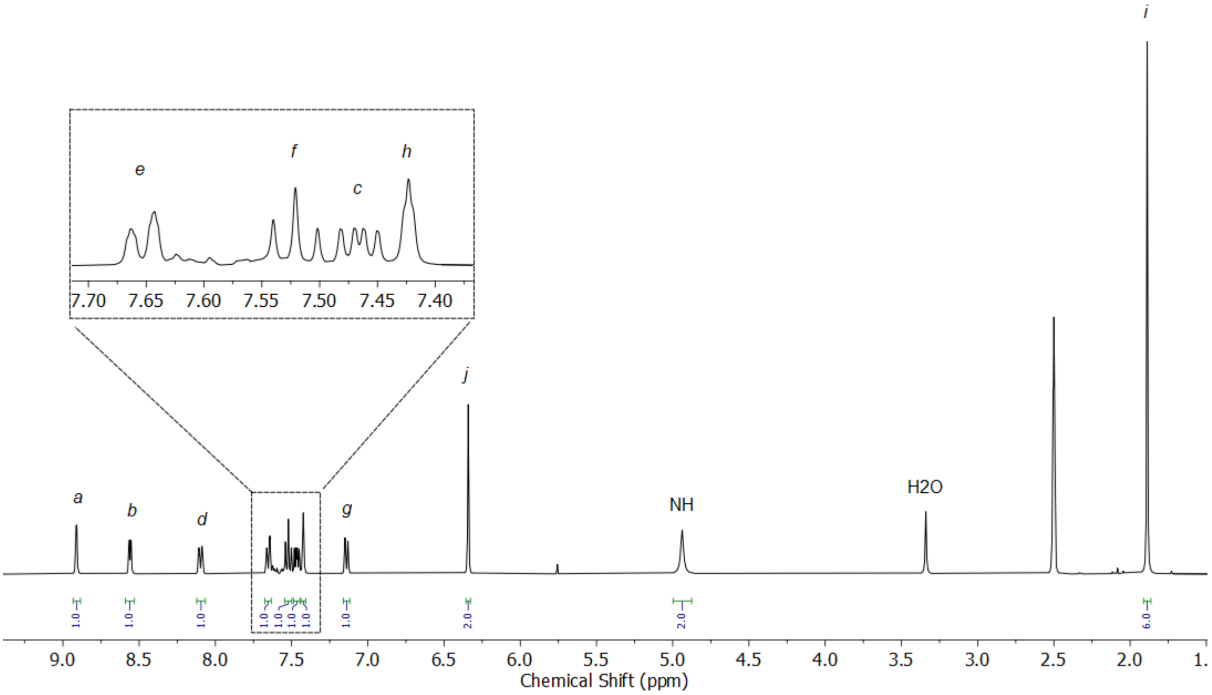

Figure S78 ^{1}H NMR (400 MHz, d_6-DMSO) of **S8**.

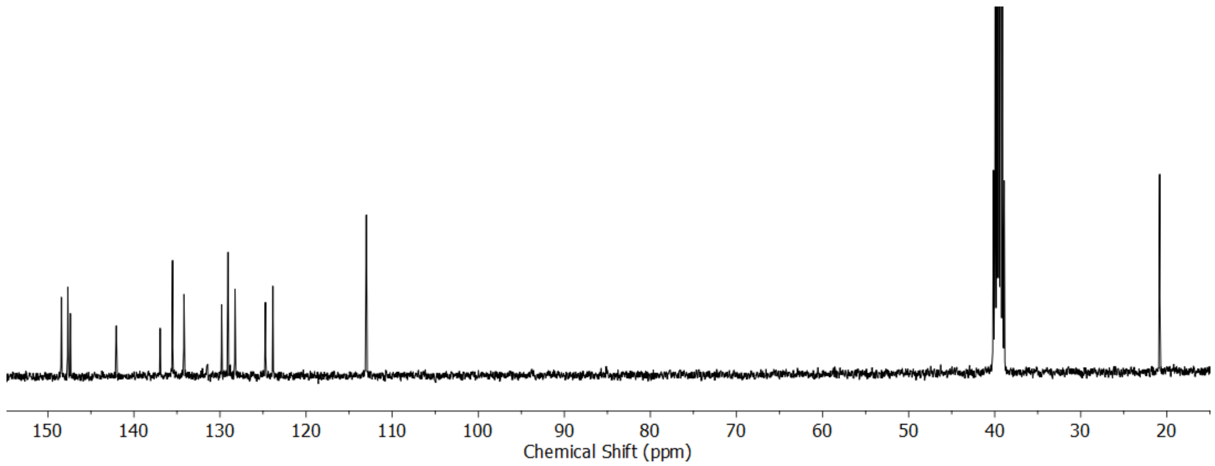

Figure S79 ^{13}C NMR (101 MHz, d_6-DMSO) of **S8**.

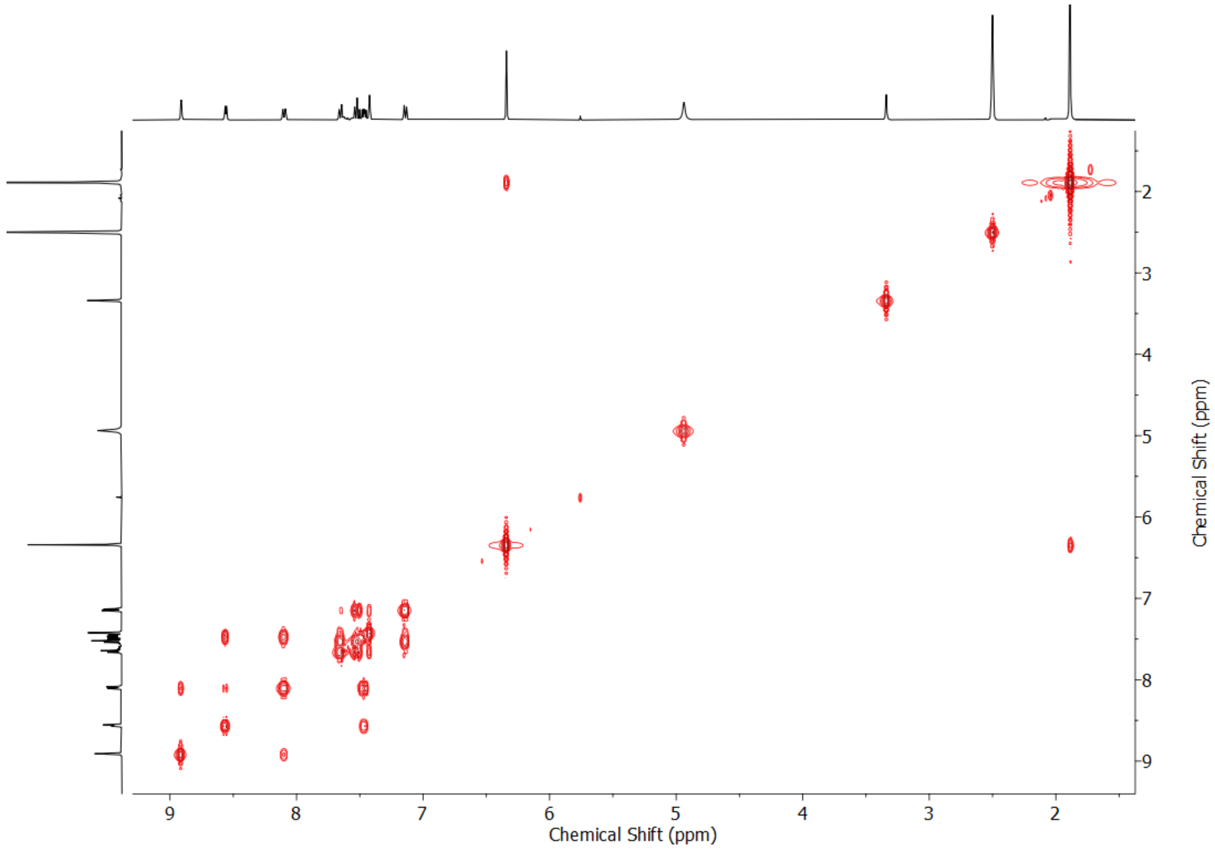

Figure S80 COSY NMR (d_6-DMSO) of **S8**.

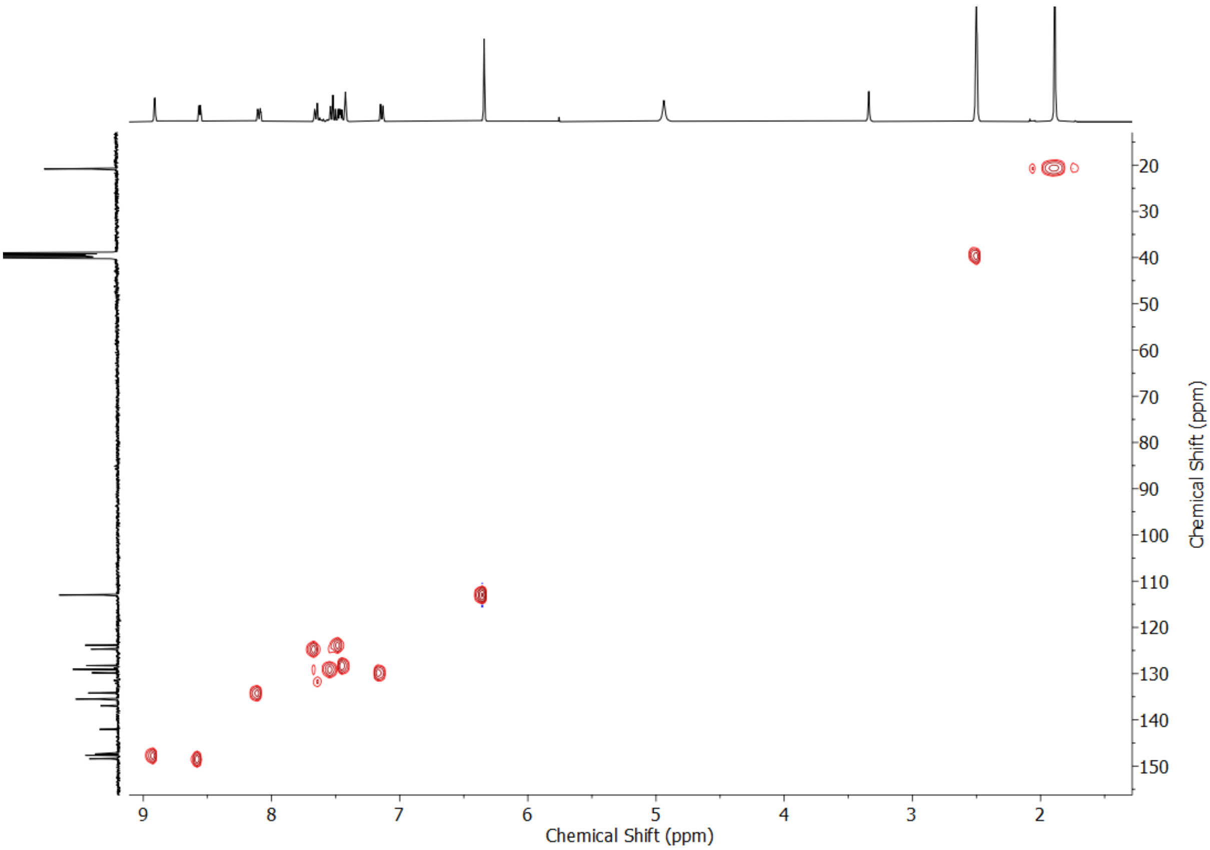

Figure S81 HSQC NMR (d_6-DMSO) of **S8**.

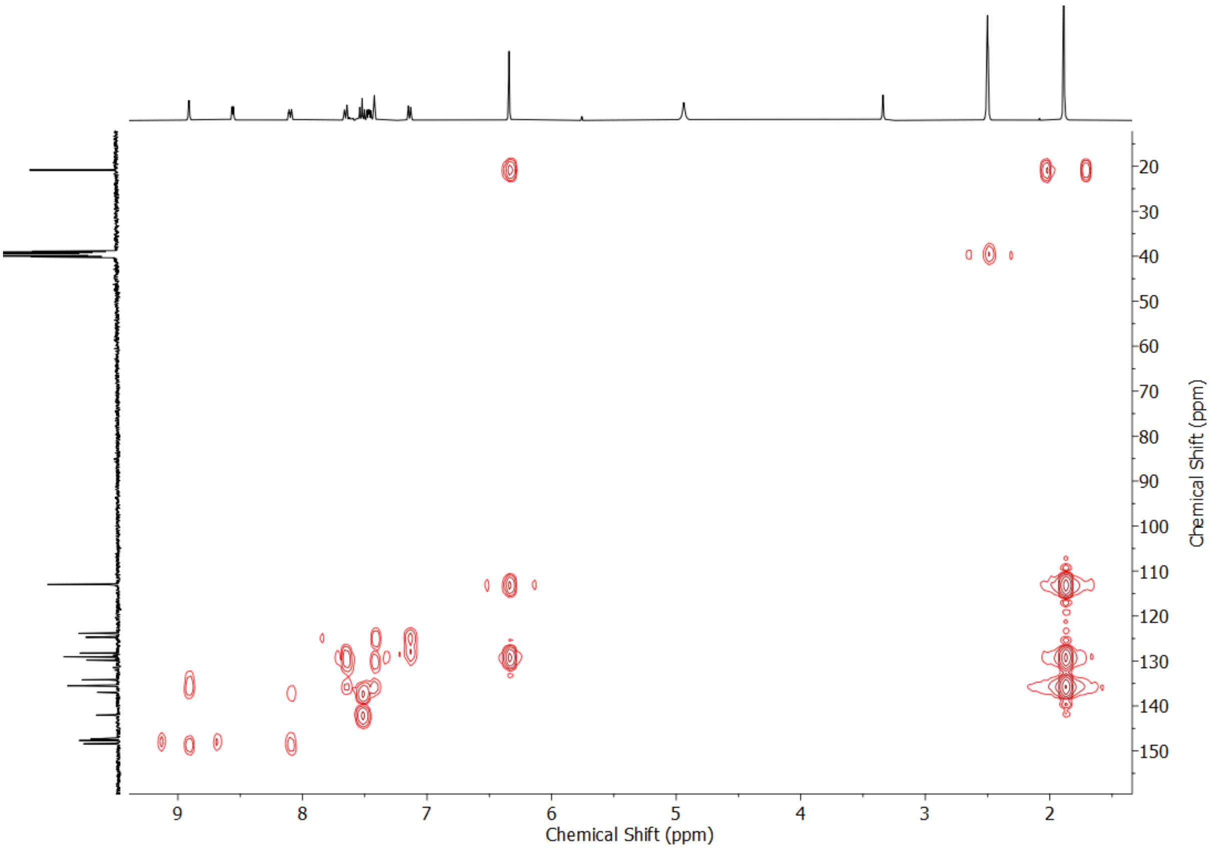

Figure S82 HMBC NMR (d_6-DMSO) of **S8**.

Synthesis of S9

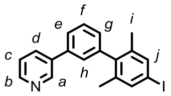

To a vigorously stirring solution of **S8** (0.412 g, 1.5 mmol, 1 eq.) in CH_3CN (9 mL) at 0 °C under air was added TsOH·H_2O (0.856 g, 4.5 mmol, 3 eq.) portionwise. A solution of $NaNO_2$ (0.207 g, 3 mmol, 2 eq.) and KI (0.623 g, 2.75 mmol, 2.5 eq.) in H_2O (1 mL) was added dropwise and the reaction mixture stirred, allowing to warm to rt, for 15 h. H_2O (20 mL), sat. aq. $NaHCO_3$ (20 mL) and 0.5 M $Na_2S_2O_{3(aq)}$ were added sequentially. The aqueous phase was extracted with EtOAc (3 × 25 mL) and the combined organic phases dried ($MgSO_4$) and the solvent removed *in vacuo*. After purification by column chromatography on silica gel (1:9 acetone/CH_2Cl_2) the product was obtained as an orange oil (0.427 g, 74%).

^{1}H NMR (400 MHz, $CDCl_3$) δ: 8.87 (dd, *J* = 2.4, 0.9 Hz, 1H, H_a), 8.60 (dd, *J* = 4.8, 1.6 Hz, 1H, H_b), 7.89 (ddd, *J* = 7.9, 2.4, 1.6 Hz, 1H, H_d), 7.60-7.52 (m, 2H, H_e/H_g, H_f), 7.50 (s, 2H, H_j), 7.38-7.34 (m, 2H, H_c, H_h) 7.16 (app. dt, *J* = 7.2, 1.6 Hz, 1H, H_e/H_g), 2.02 (s, 6H, H_i).

^{13}C NMR (101 MHz, $CDCl_3$) δ: 148.8 (C_b), 148.5 (C_a), 141.1, 141.1, 138.5, 138.3, 136.5, 136.3 (C_j), 134.5 (C_d), 129.6 (C_f), 128.8 (C_e/C_g), 127.6 (C_h), 125.9 (C_e/C_g), 123.7 (C_c), 93.1, 20.6 (C_i).

HR-ESI-MS *m/z* = 386.0394 [M+H]$^+$ calc. 386.0406.

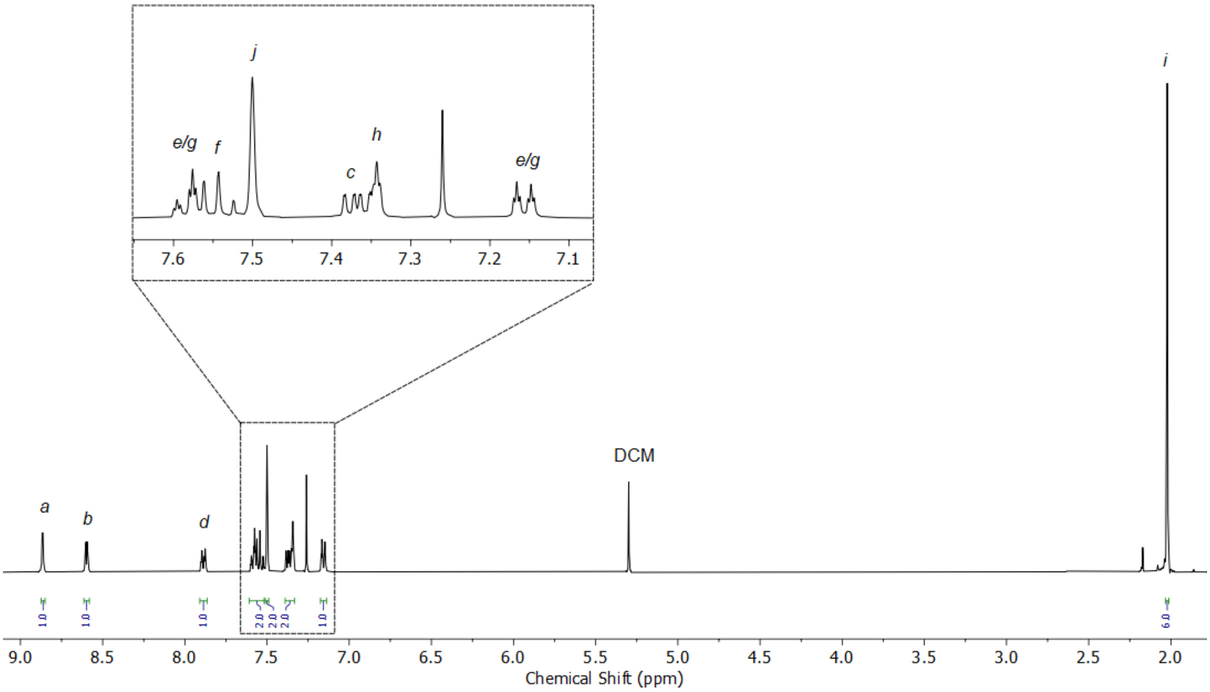

Figure S83 ^{1}H NMR (400 MHz, $CDCl_3$) of **S9**.

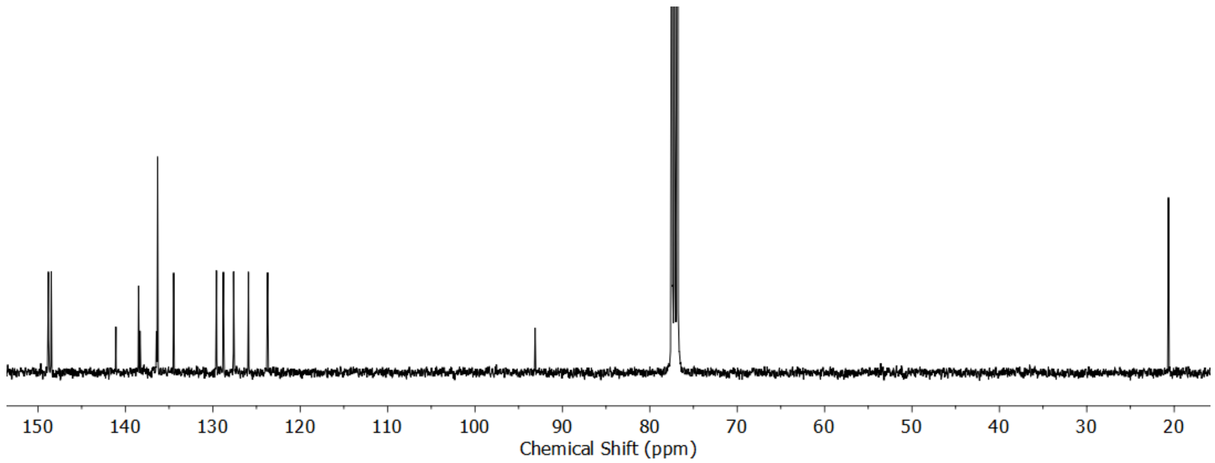

Figure S84 ^{13}C NMR (101 MHz, $CDCl_3$) of **S9**.

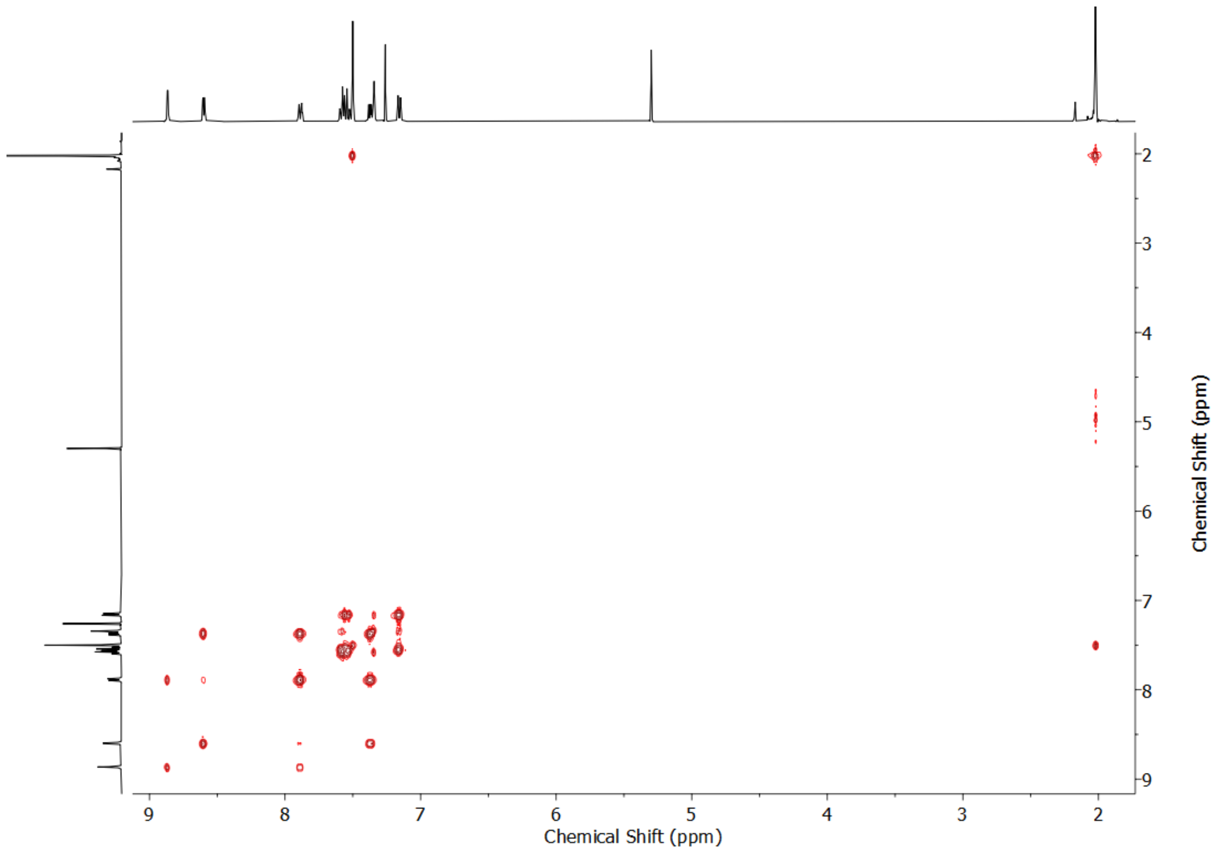

Figure S85 COSY NMR ($CDCl_3$) of **S9**.

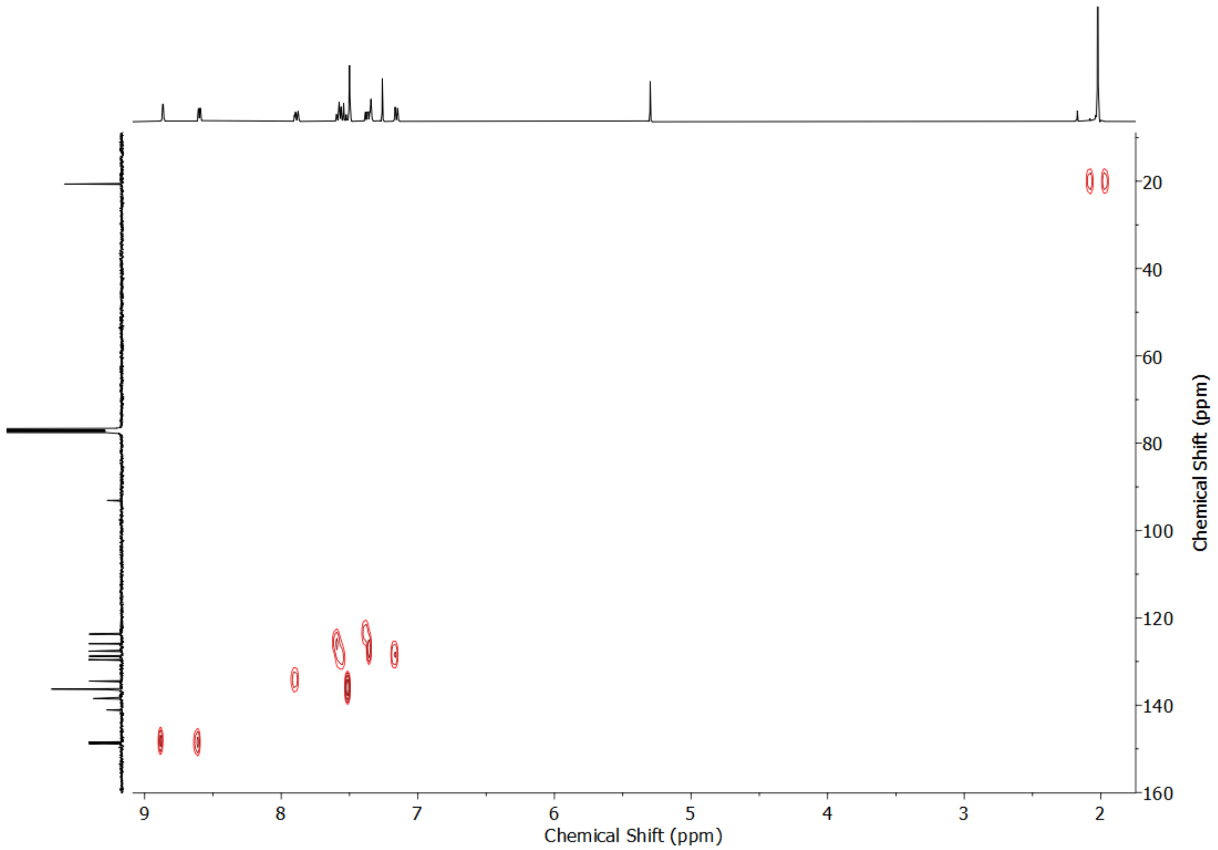

Figure S86 HSQC NMR ($CDCl_3$) of **S9**.

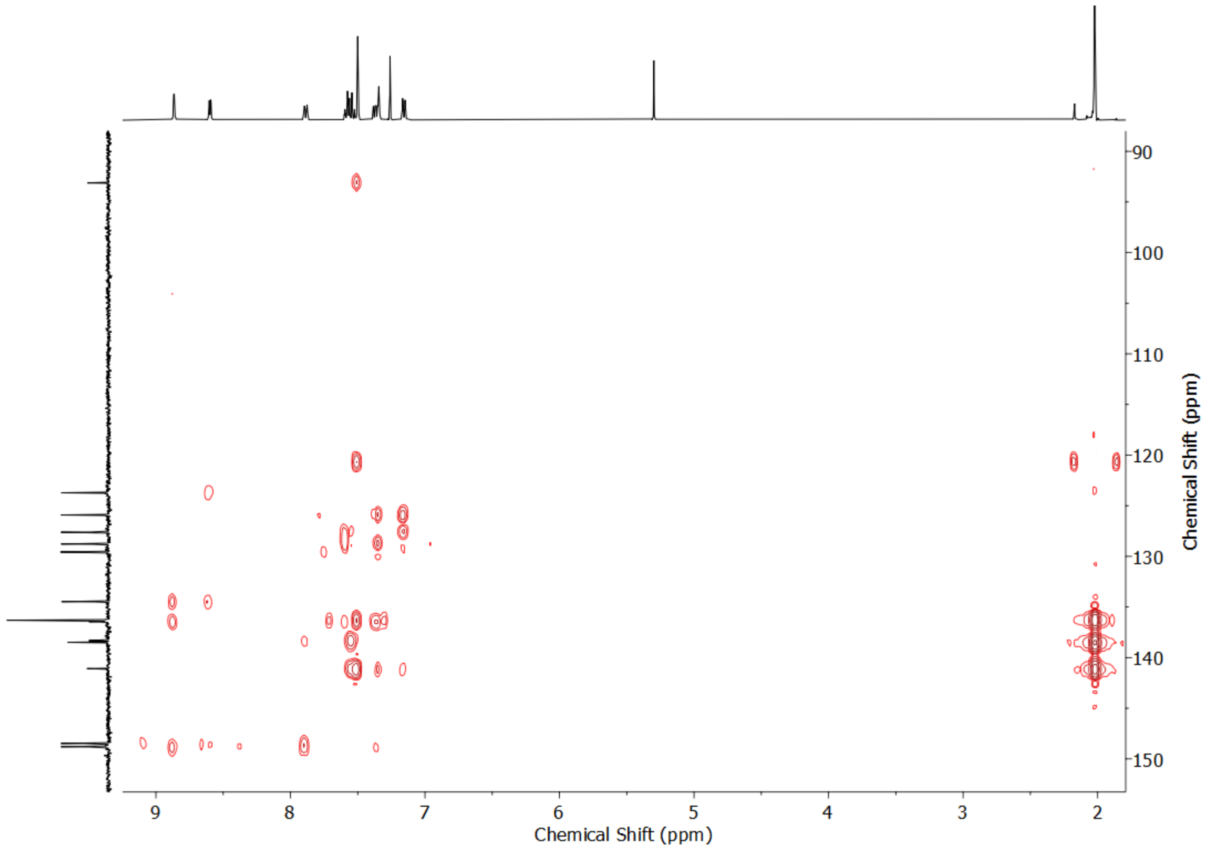

Figure S87 HMBC NMR ($CDCl_3$) of **S9**.

Synthesis of L1^{Xy}

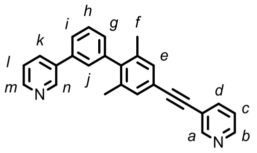

S9 (0.193 g, 0.5 mmol, 1 eq.), 3-ethynylpyridine (0.057 g, 0.55 mmol, 1.1 eq.), $Pd(PPh_3)_2Cl_2$ (0.0088 g, 0.013 mmol, 2.5 mol%) and CuI (0.0048 g, 0.025 mmol, 5 mol%) were stirred at rt in $^{i}Pr_2NH$ (5 mL) for 2 d. EDTA solution (20 mL) was added and the aqueous phase extracted with CH_2Cl_2 (3 × 10 mL). The combined organic phases were dried ($MgSO_4$) and the solvent removed *in vacuo*. Following purification by column chromatography (pentane with step gradient 0 to 45% EtOAc in 15% increments) the product was obtained as a thick, orange oil (0.178 g, 99%).

^{1}H NMR (400 MHz, $CDCl_3$) δ: 8.88 (dd, *J* = 2.4, 0.9 Hz, 1H, H_n), 8.78 (dd, *J* = 2.1, 0.9 Hz, 1H, H_a), 8.60 (dd, *J* = 4.8 1.6 Hz, 1H, H_m), 8.55 (dd, *J* = 4.9, 1.7 Hz, 1H, H_b), 7.90 (m, 1H, H_k), 7.82 (dt, *J* = 7.9, 1.9 Hz, 1H, H_d), 7.61-7.54 (m, 2H, 2 of H_g/H_h/H_i/H_j), 7.39-7.34 (m, 4H, H_e, H_l, 1 of H_g/H_h/H_i/H_j), 7.29 (ddd, *J* = 7.9, 4.9, 0.9 Hz, 1H, H_c), 7.19 (app. dt, *J* = 7.1, 1.6 Hz, 1H, H_g/H_i), 2.09 (s, 6H, H_f).

^{13}C NMR (101 MHz, $CDCl_3$) δ: 152.4 (C_a), 148.8 (C_m), 148.6 (C_b), 148.5 (C_n), 142.3, 141.4, 138.6 (C_d), 138.3, 136.6, 136.5, 134.5 (C_k), 130.7 (C_e), 129.5 (C_g/C_h/C_i/C_j), 128.8 (C_g/C_i), 127.6 (C_l/C_g/C_h/C_i/C_j), 125.9 (C_g/C_h/C_i/C_j), 123.7 (C_l/C_g/C_h/C_i/C_j), 123.2 (C_c), 121.4, 120.8, 92.9, 85.7, 20.9 (C_f).

HR-ESI-MS *m/z* = 361.1705 [M+H]$^+$ calc. 361.1705.

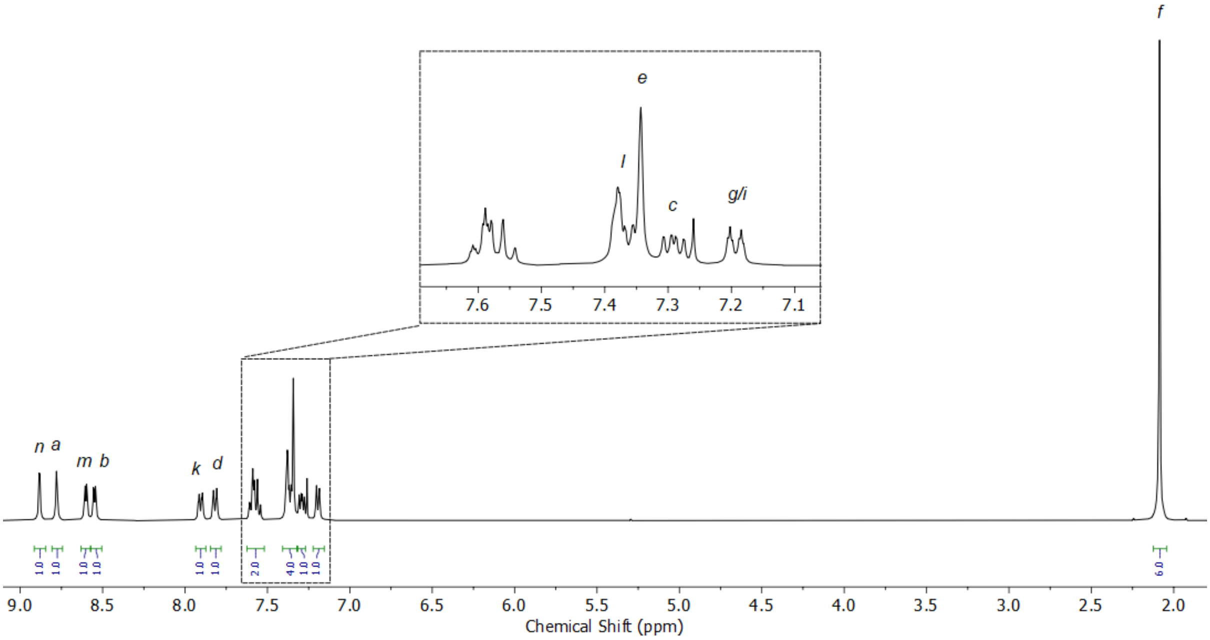

Figure S88 ^{1}H NMR (400 MHz, $CDCl_3$) of **L1^{Xy}**.

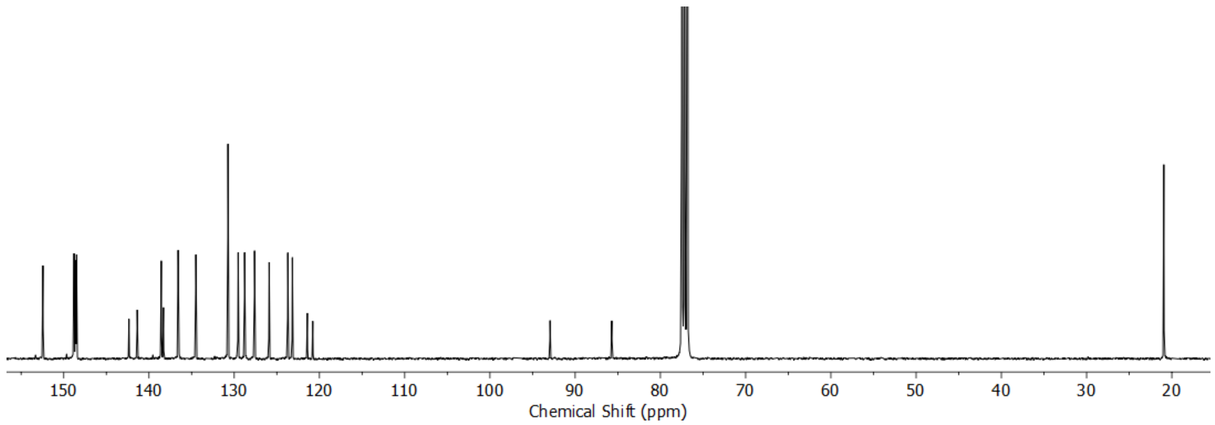

Figure S89 ^{13}C NMR (101 MHz, $CDCl_3$) of **L1**^{Xy}.

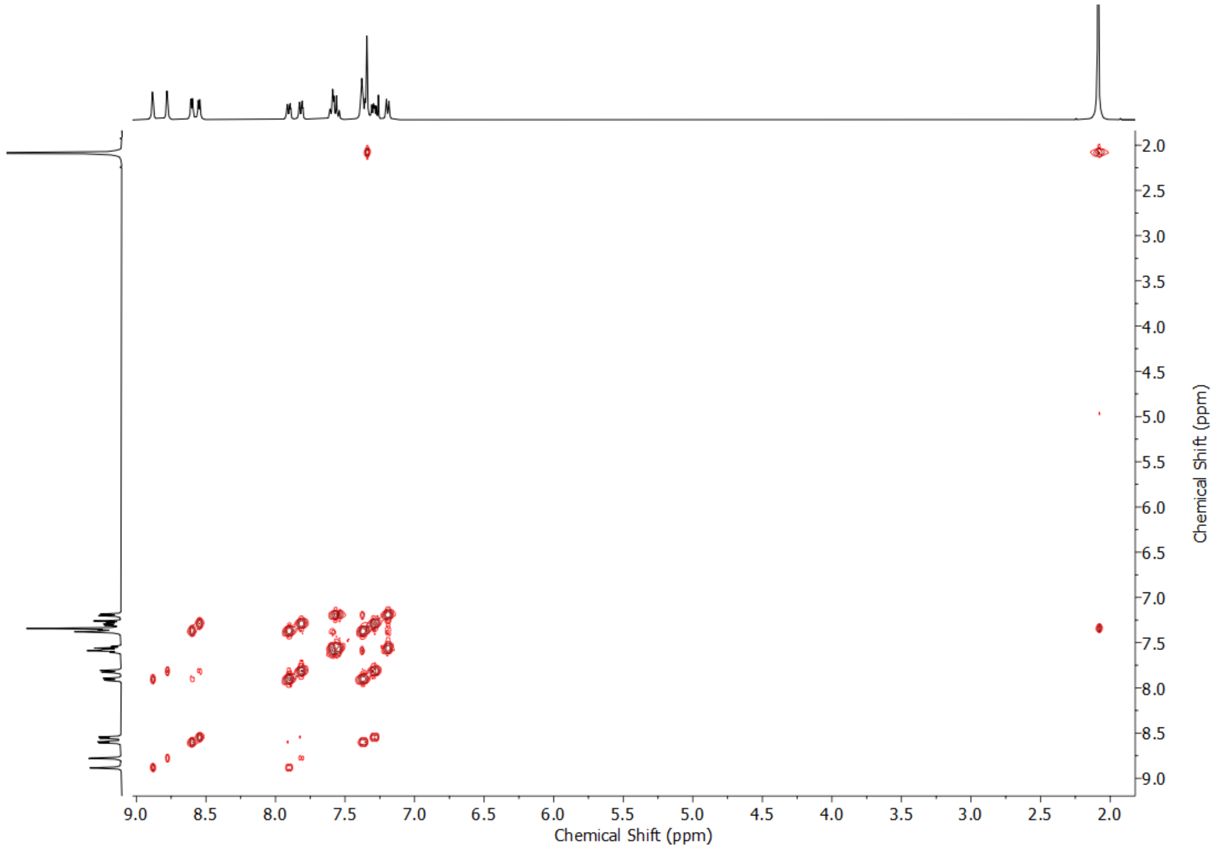

Figure S90 COSY NMR ($CDCl_3$) of **L1**^{Xy}.

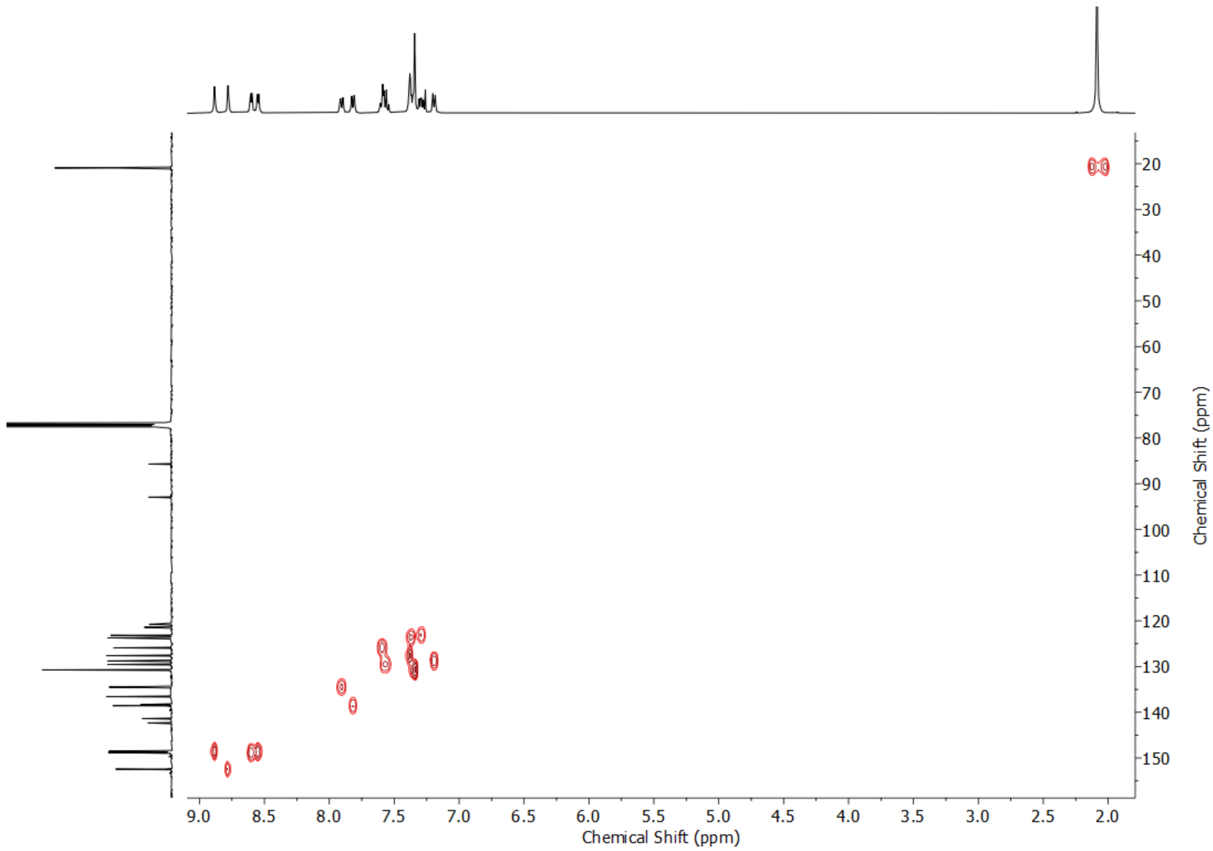

Figure S91 HSQC NMR ($CDCl_3$) of **L1**^{Xy}.

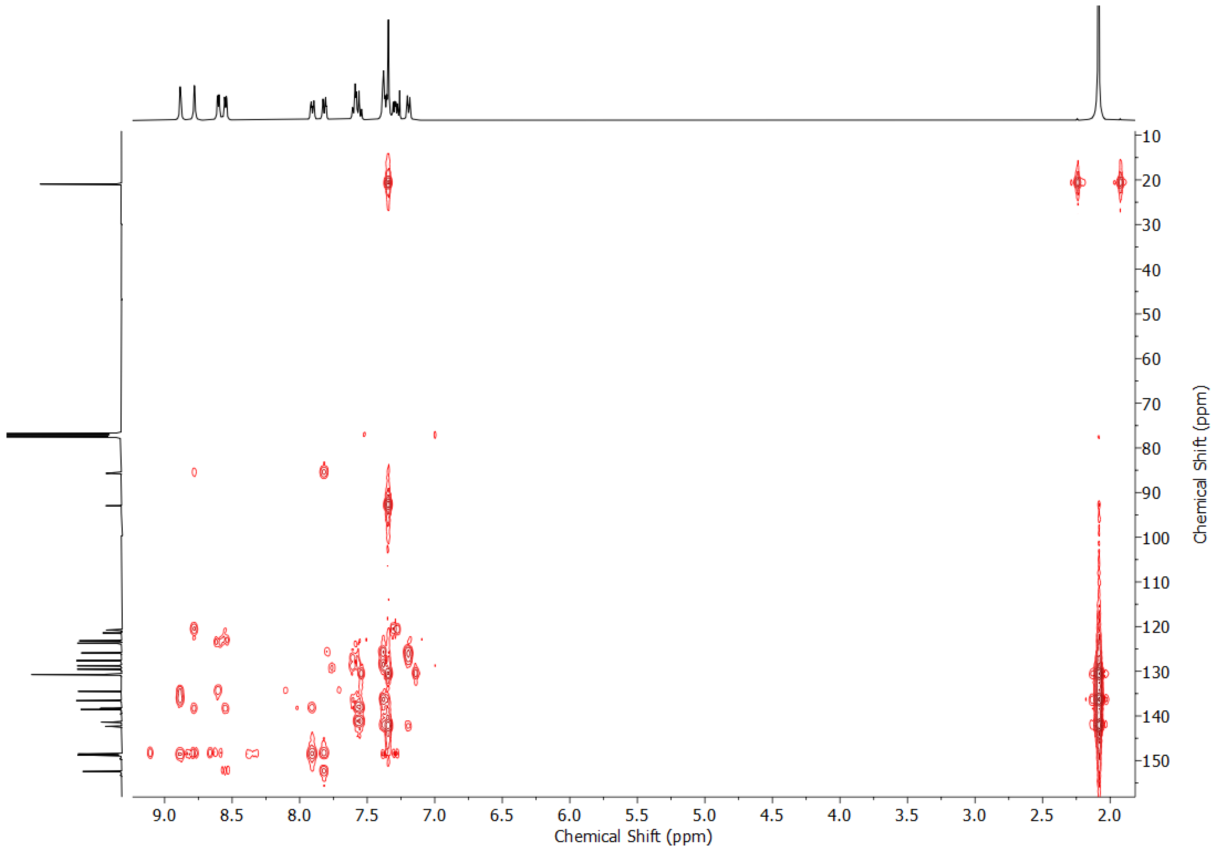

Figure S92 HMBC NMR ($CDCl_3$) of **L1**^{Xy}.

Synthesis of *cis*-[$Pd_2(L1^{Xy})_4$]$(BF_4)_4$ ($C1^{Xy}$)

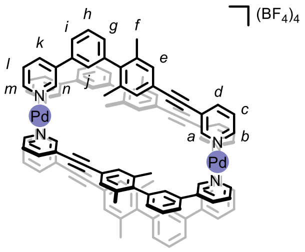

L1^{Xy} (10.8 mg, 30 µmol, 2 eq.) and [$Pd(CH_3CN)_4$]$(BF_4)_2$ (6.7 mg, 15 µmol, 1 eq.) were sonicated in d_6-DMSO (0.75 mL) until a homogenous solution was obtained. After standing at rt for 6 h, quantitative conversion to *cis*-[Pd_2(**L1^{Xy}**)$_4$]$(BF_4)_4$ was observed by ^{1}H NMR.

^{1}H NMR (400 MHz, d_6-DMSO) δ: 9.61 (s, 4H, H_a), 9.27 (d, J = 5.5 Hz, 4H, H_m), 9.16-9.13 (m, 8H, H_b, H_n), 8.60 (d, J = 8.7 Hz, 4H, H_k), 8.20 (app. dt, J = 8.0, 1.5 Hz, 4H, H_d), 7.93 (dd, J = 8.1, 5.7 Hz, 4H, H_l), 7.81 (d, J = 7.7 Hz, 4H, H_i), 7.73-7.70 (m, 8H, H_c, H_j), 7.64 (app. t, J = 7.6 Hz, 4H, H_h), 7.45 (s, 4H, H_e), 7.30-7.28 (m, 8H, $H_{e'}$, H_g), 2.13 (s, 12H, H_f), 1.99 (s, 12H, $H_{f'}$).

Diffusion coefficient (400 MHz, d_6-DMSO) D: 9.10 × 10^{-11} m^2s^{-1}; R_H: 11.0 Å.

^{13}C NMR (101 MHz, d_6-DMSO) δ: 152.6 (C_a), 150.6 (C_b/C_n), 149.6 (C_m), 149.0 (C_b/C_n), 142.6 (C_d), 142.5, 140.2, 138.4 (C_k), 137.7, 137.0, 136.7, 134.3, 130.3 (C_e), 130.1 (C_h), 129.8 ($C_{e'}/C_g$), 129.8 ($C_{e'}/C_g$), 127.5 (C_l), 127.2 (C_c/C_j), 126.3 (C_i), 126.2 (C_c/C_j), 122.4, 119.7, 94.0, 82.7, 20.6 ($C_f/C_{f'}$), 20.5 ($C_f/C_{f'}$).

ESI-MS m/z = 914.73 {[Pd_2(**L1^{Xy}**)$_4$]$(BF_4)_2$}$^{2+}$ calc. 914.73; 1874.41 {[Pd_2(**L1^{Xy}**)$_4$]$(BF_4)_2(HCO_2)$}$^+$ calc. 1874.47.

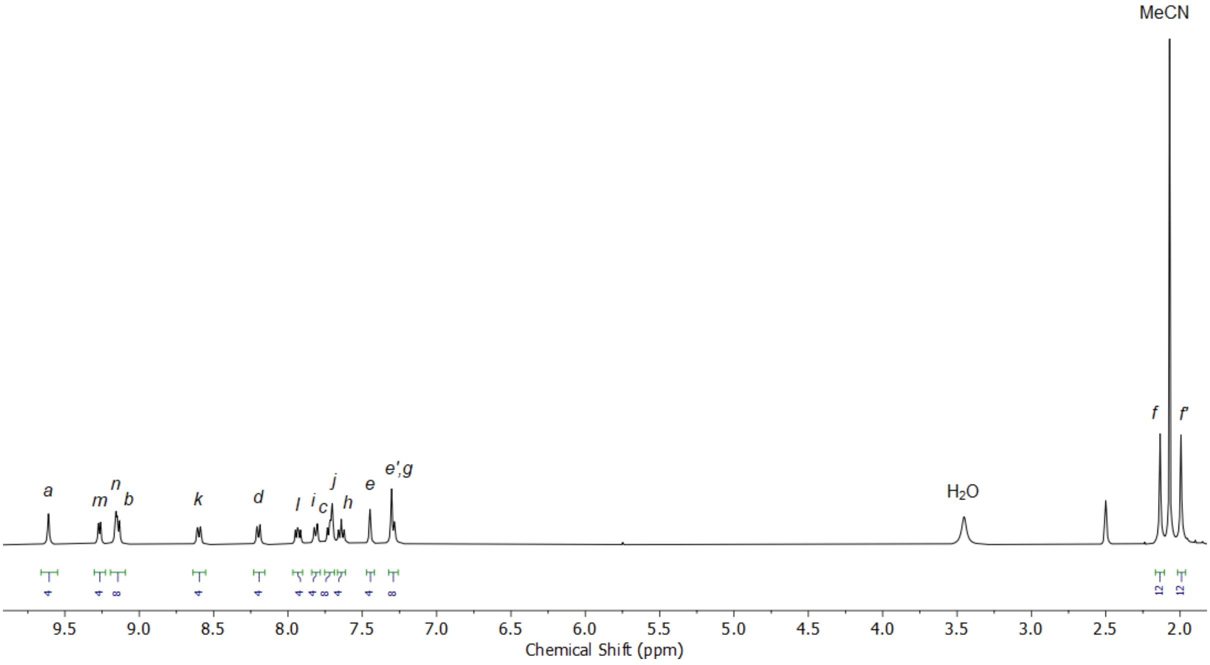

Figure S93 ^{1}H NMR (400 MHz, d_6-DMSO) of [Pd_2(**L1^{Xy}**)$_4$]$(BF_4)_4$.

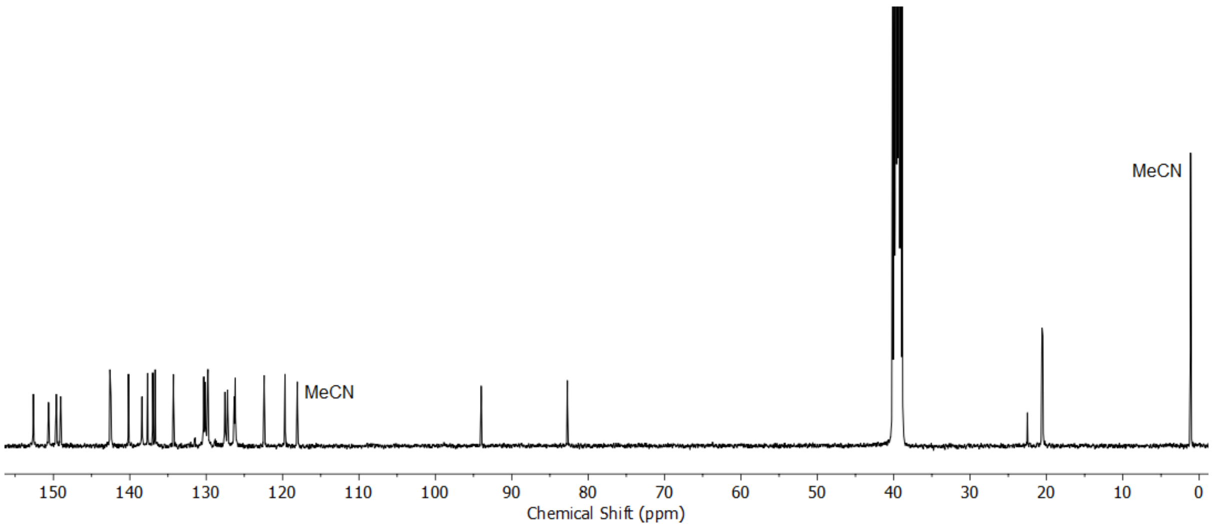

Figure S94 ^{13}C NMR (101 MHz, d_6-DMSO) of $[Pd_2(\mathbf{L1^{Xy}})_4](BF_4)_4$.

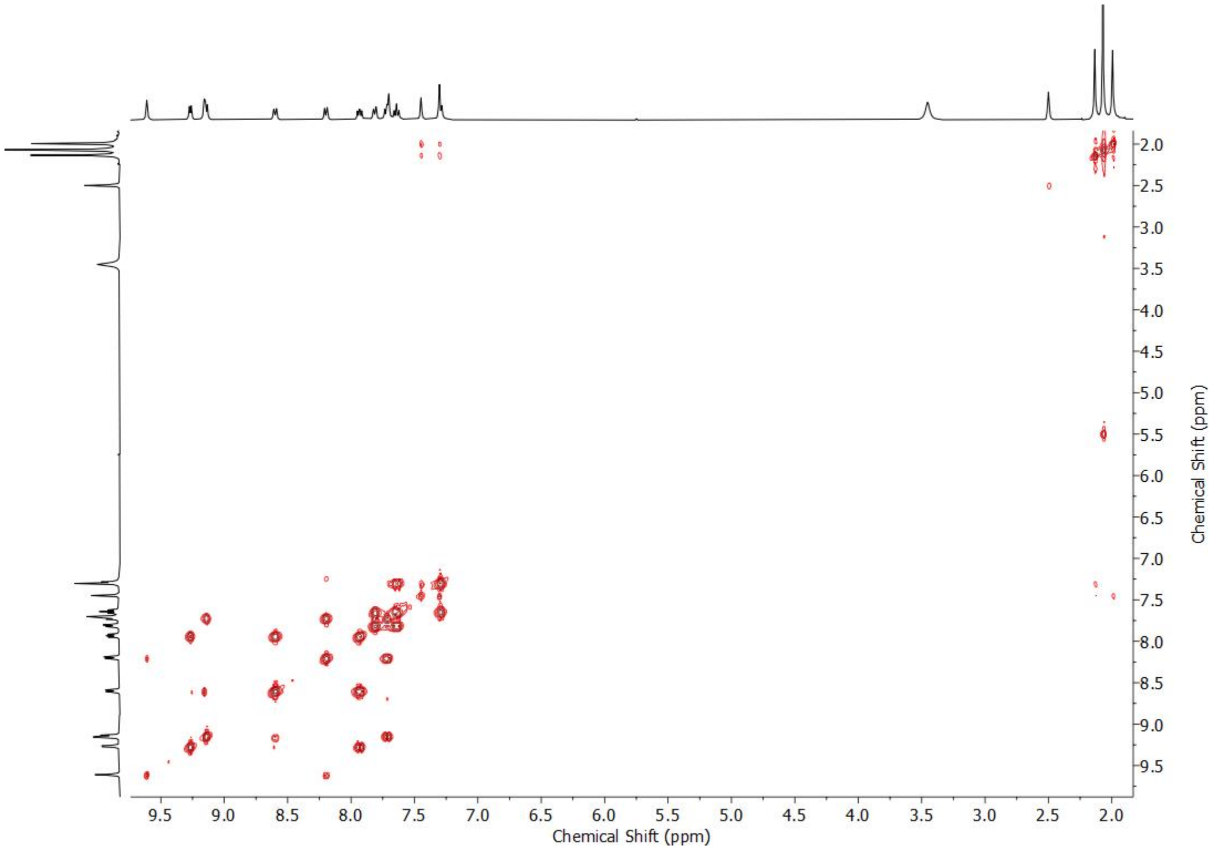

Figure S95 COSY NMR (d_6-DMSO) of $[Pd_2(\mathbf{L1^{Xy}})_4](BF_4)_4$.

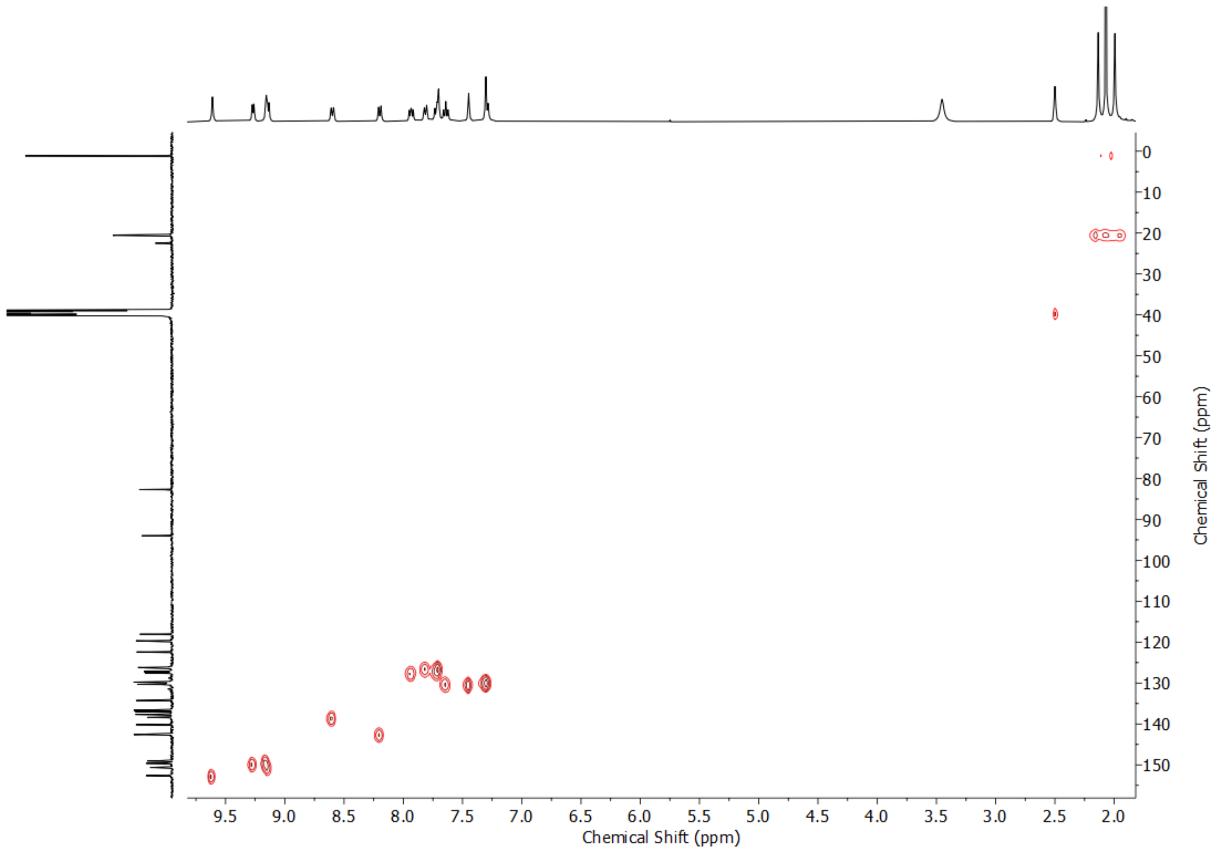

Figure S96 HSQC NMR (d_6-DMSO) of $[Pd_2(\mathbf{L1^{Xy}})_4](BF_4)_4$.

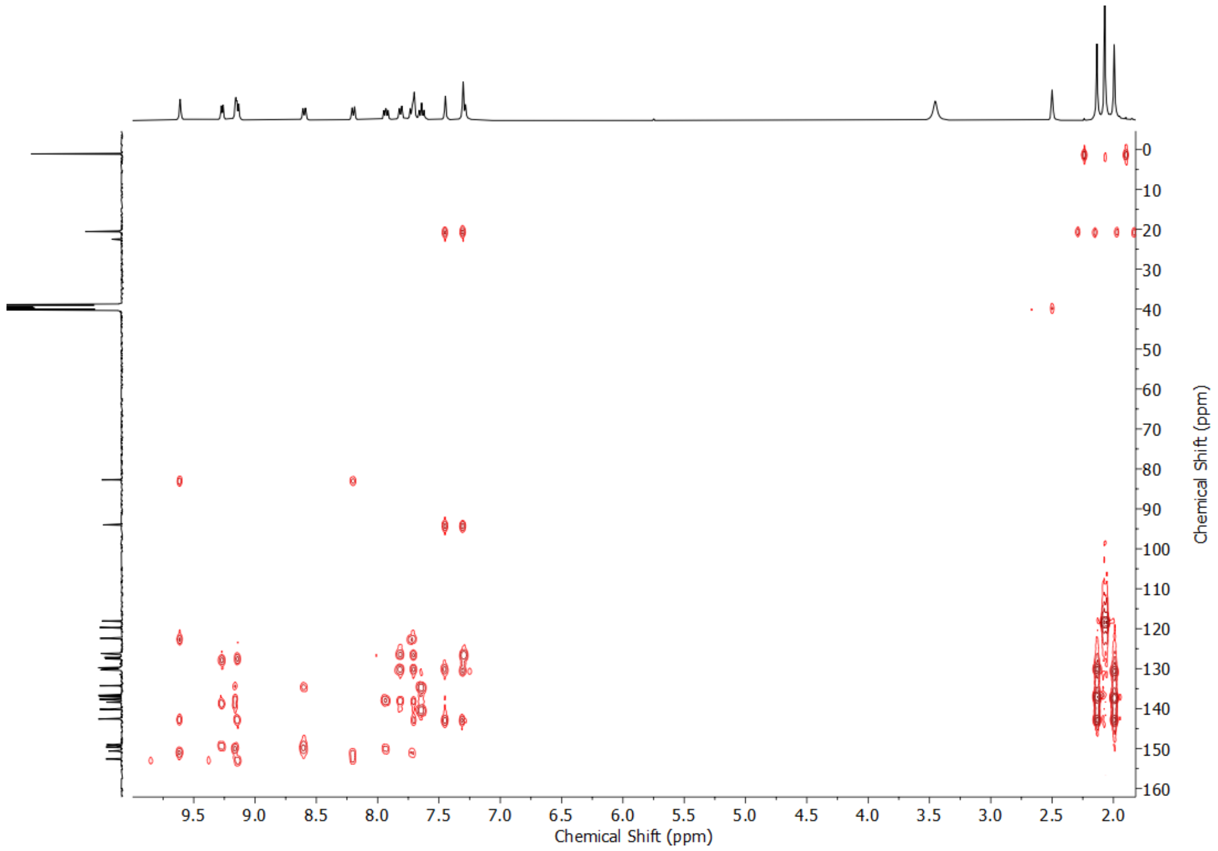

Figure S97 HMBC NMR (d_6-DMSO) of $[Pd_2(\mathbf{L1^{Xy}})_4](BF_4)_4$.

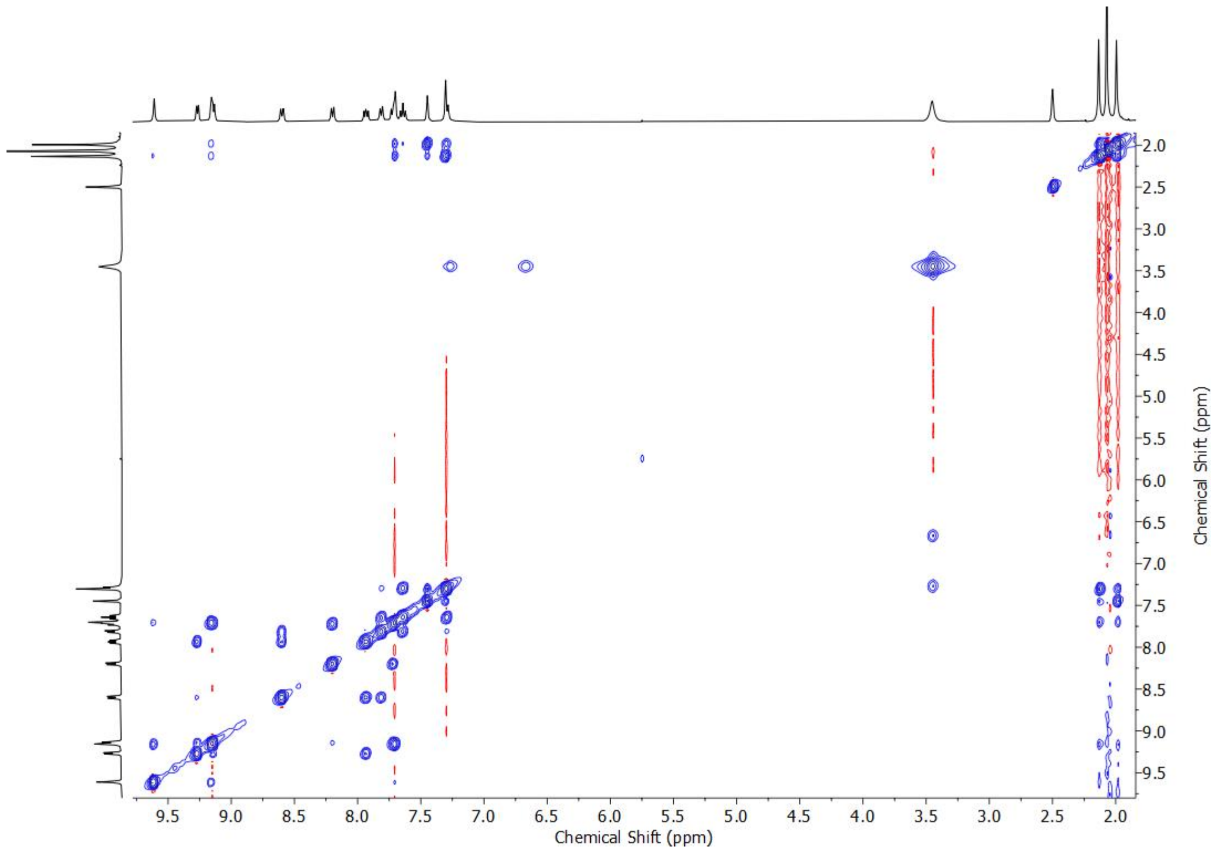

Figure S98 NOESY (d_6-DMSO) of $[Pd_2(\mathbf{L1}^{\mathbf{Xy}})_4](BF_4)_4$.

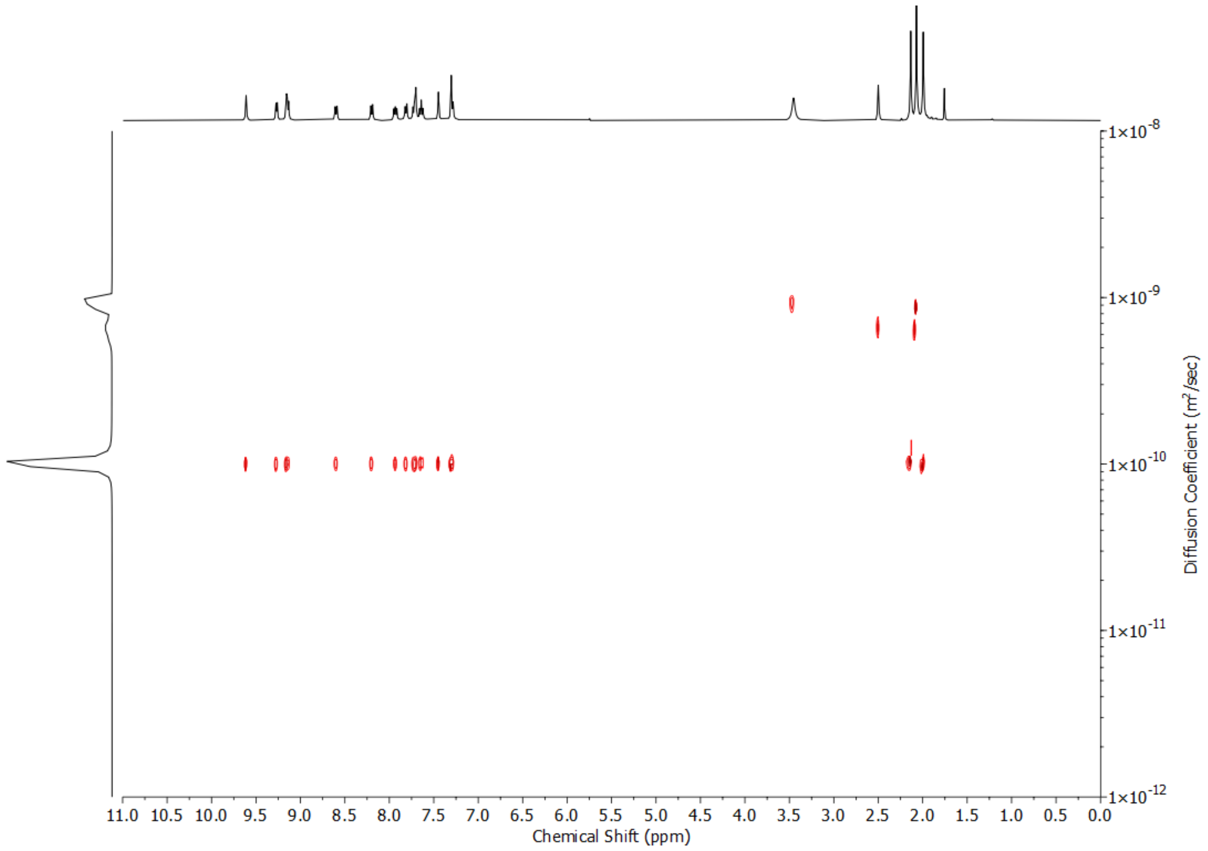

Figure S99 DOSY (d_6-DMSO) of $[Pd_2(\mathbf{L1}^{\mathbf{Xy}})_4](BF_4)_4$.

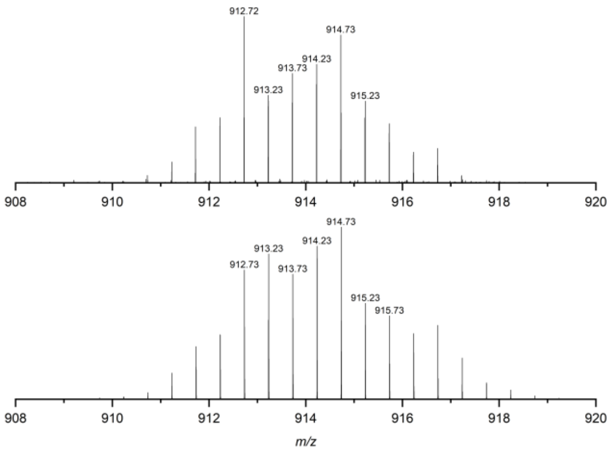

Figure S100 Observed (top) and calculated (bottom) isotopic patterns for $\{[Pd_2(\mathbf{L1^{Xy}})_4](BF_4)_2\}^{2+}$.

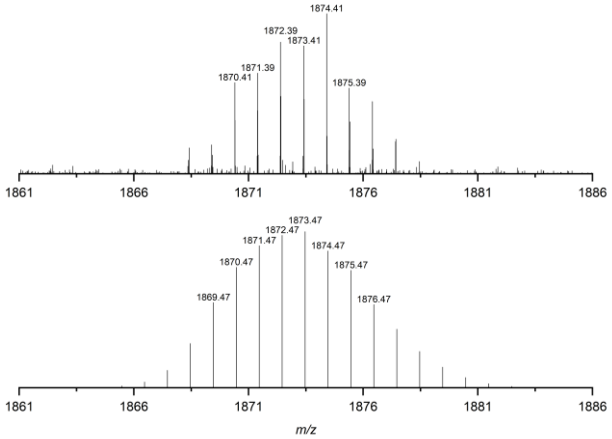

Figure S101 Observed (top) and calculated (bottom) isotopic patterns for $\{[Pd_2(\mathbf{L1^{Xy}})_4](BF_4)_2(HCO_2)\}^{+}$.

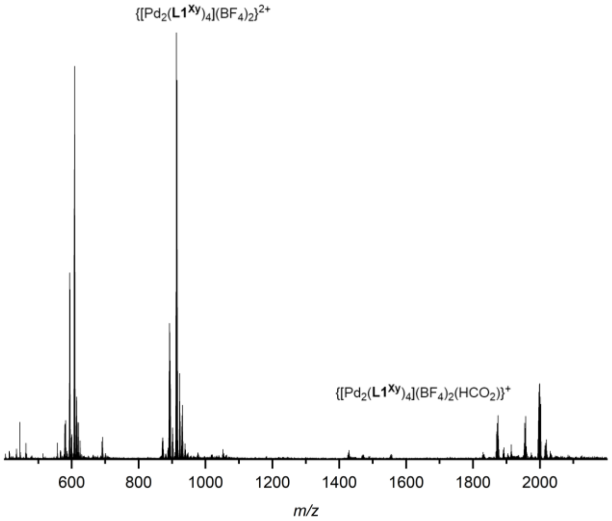

Figure S102 ESI-MS of $[Pd_2(\mathbf{L1^{Xy}})_4](BF_4)_4$.

Synthesis of S10

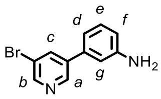

3-Aminophenylboronic acid (1.37 g, 10.0 mmol, 1 eq.), 3,5-dibromopyridine (3.55 g, 15.0 mmol, 1.5 eq.), $Pd(PPh_3)_2Cl_2$ (0.070 g, 0.10 mmol, 1 mol%) and K_2CO_3 (3.46 g, 25.0 mmol, 2.5 eq.) were stirred at 80 °C in 2:1 1,4-dioxane/H_2O (30 mL) for 22 h. H_2O (100 mL) was added and the reaction mixture extracted with CH_2Cl_2 (3 × 50 mL). The combined organic phases were dried ($MgSO_4$) and the solvent removed *in vacuo*. Following purification by column chromatography on silica gel (1:19 acetone/CH_2Cl_2) the product was obtained as a waxy off-white solid (1.66 g, 67%).

^{1}H NMR (400 MHz, $CDCl_3$) δ: 8.72 (d, *J* = 1.9 Hz, 1H, $H_{a/b}$), 8.63 (d, *J* = 2.2 Hz, 1H, $H_{a/b}$), 7.98 (app. t, *J* = 2.1 Hz, 1H, H_c), 7.26 (m, H_e), 6.93 (d, *J* = 7.6 Hz, 1H, $H_{d/f}$), 6.84 (s, H_g) 6.74 (dd, *J* = 8.1, 2.3 Hz, 1H, $H_{d/f}$).

^{13}C NMR (101 MHz, $CDCl_3$) δ: 149.4 (C_a/C_b), 147.3, 146.5 (C_a/C_b), 138.6, 137.6, 137.0 (C_c), 130.3 (C_e), 121.0, 117.6 (C_d/C_f), 115.5 (C_g), 113.6 (C_d/C_f).

HR-ESI-MS *m/z* = 249.0031 $[M+H]^+$ calc. 249.0022.

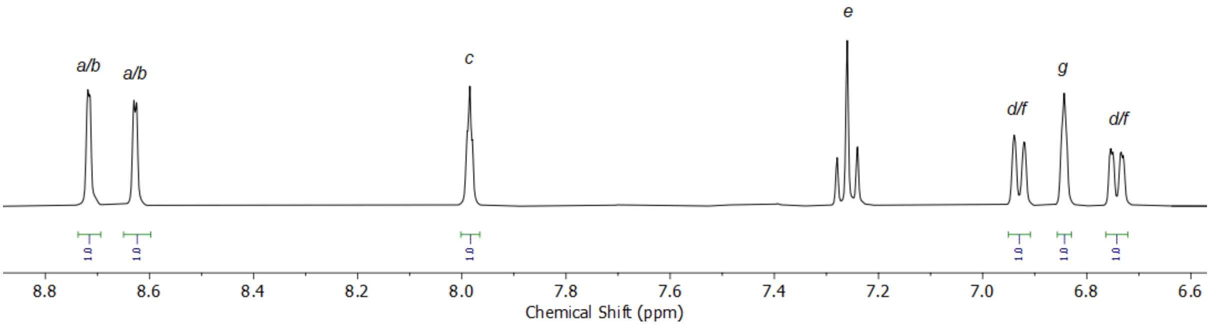

Figure S103 ^{1}H NMR (400 MHz, $CDCl_3$) of **S10**.

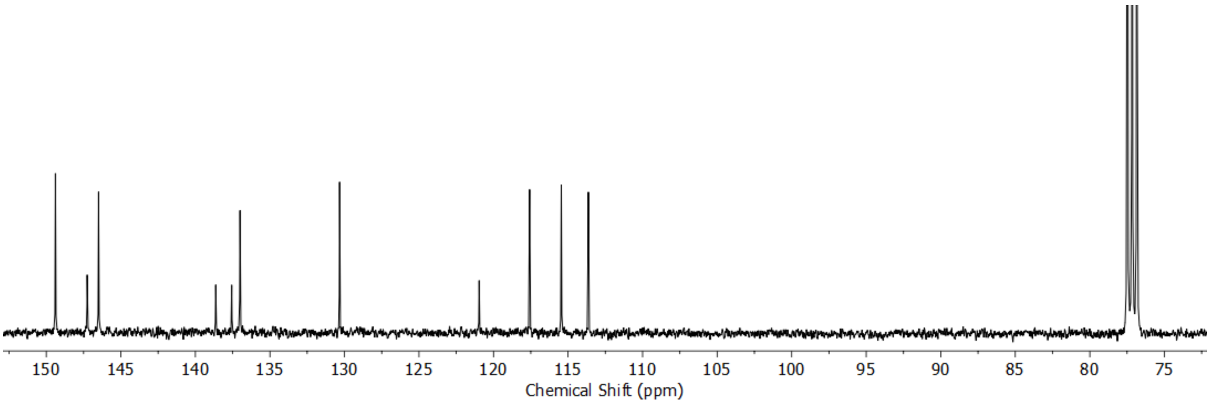

Figure S104 ^{13}C NMR (101 MHz, $CDCl_3$) of **S10**.

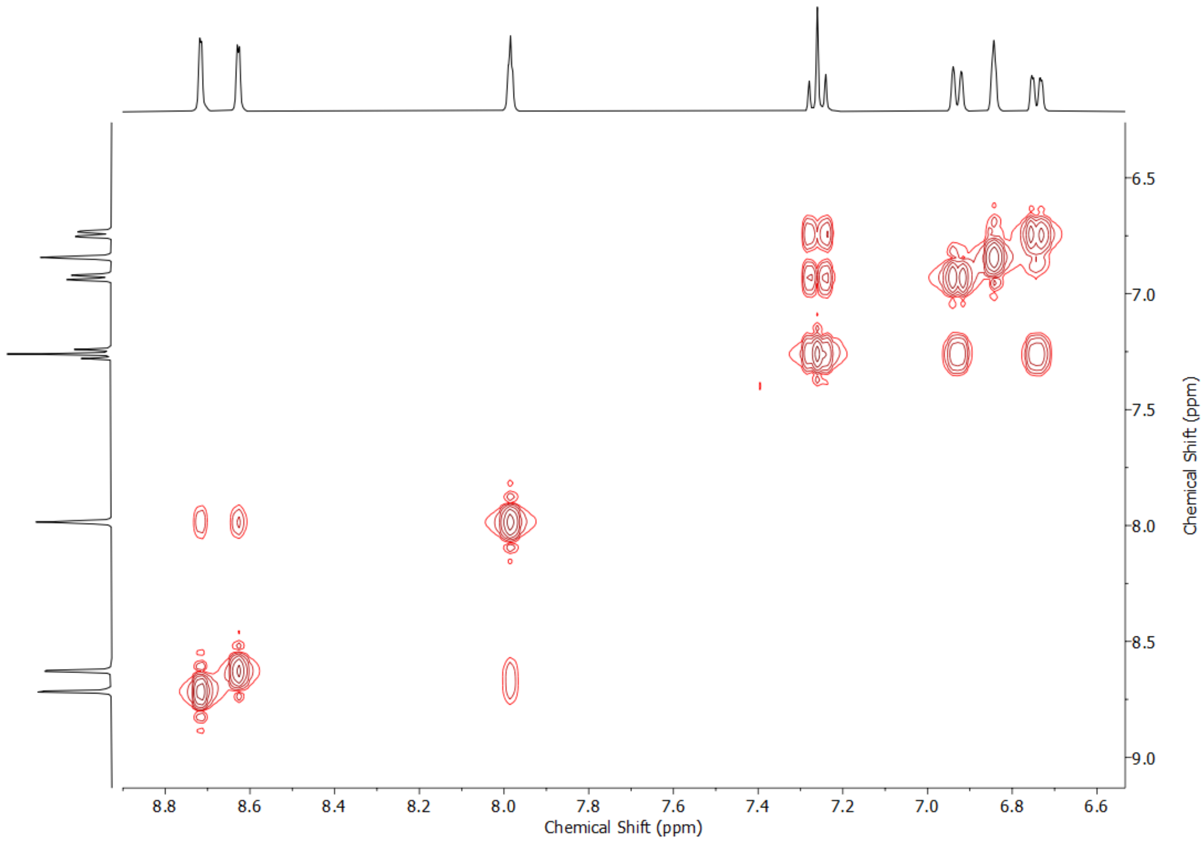

Figure S105 COSY NMR ($CDCl_3$) of **S10**.

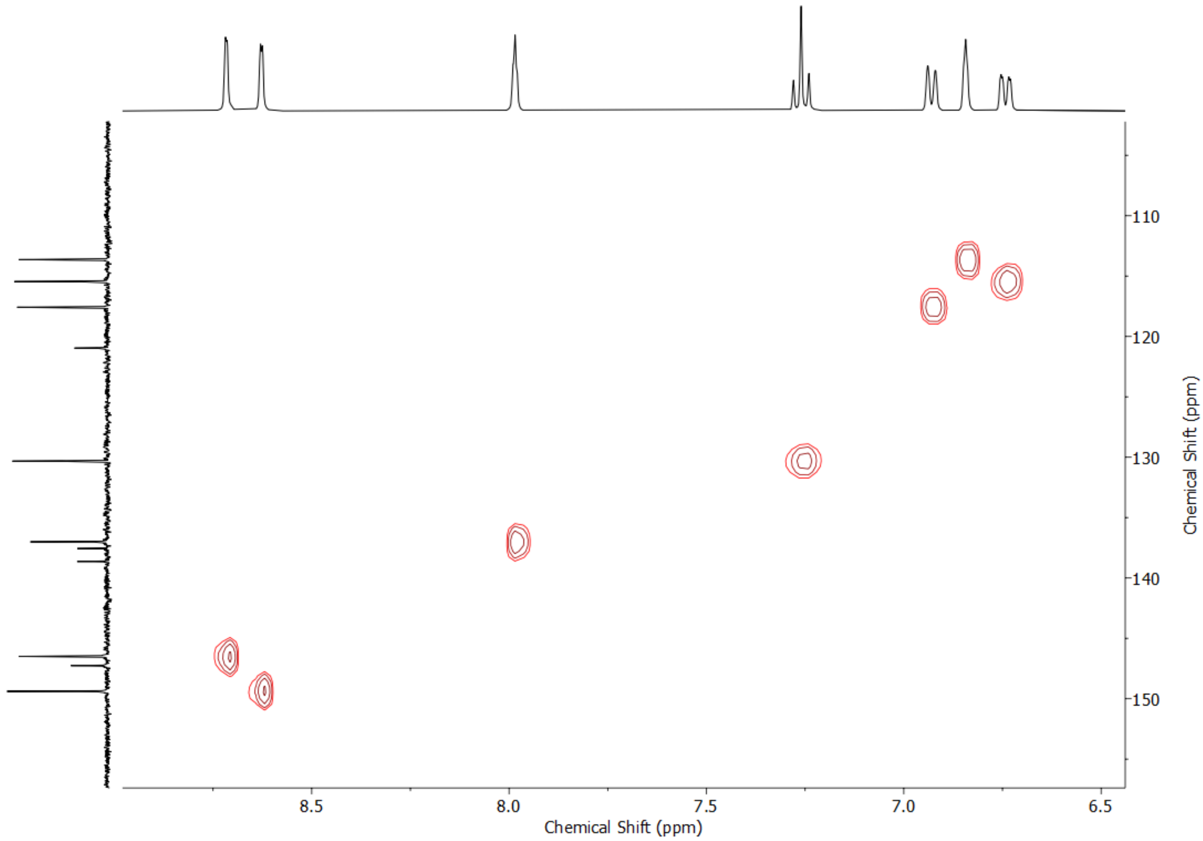

Figure S106 HSQC NMR ($CDCl_3$) of **S10**.

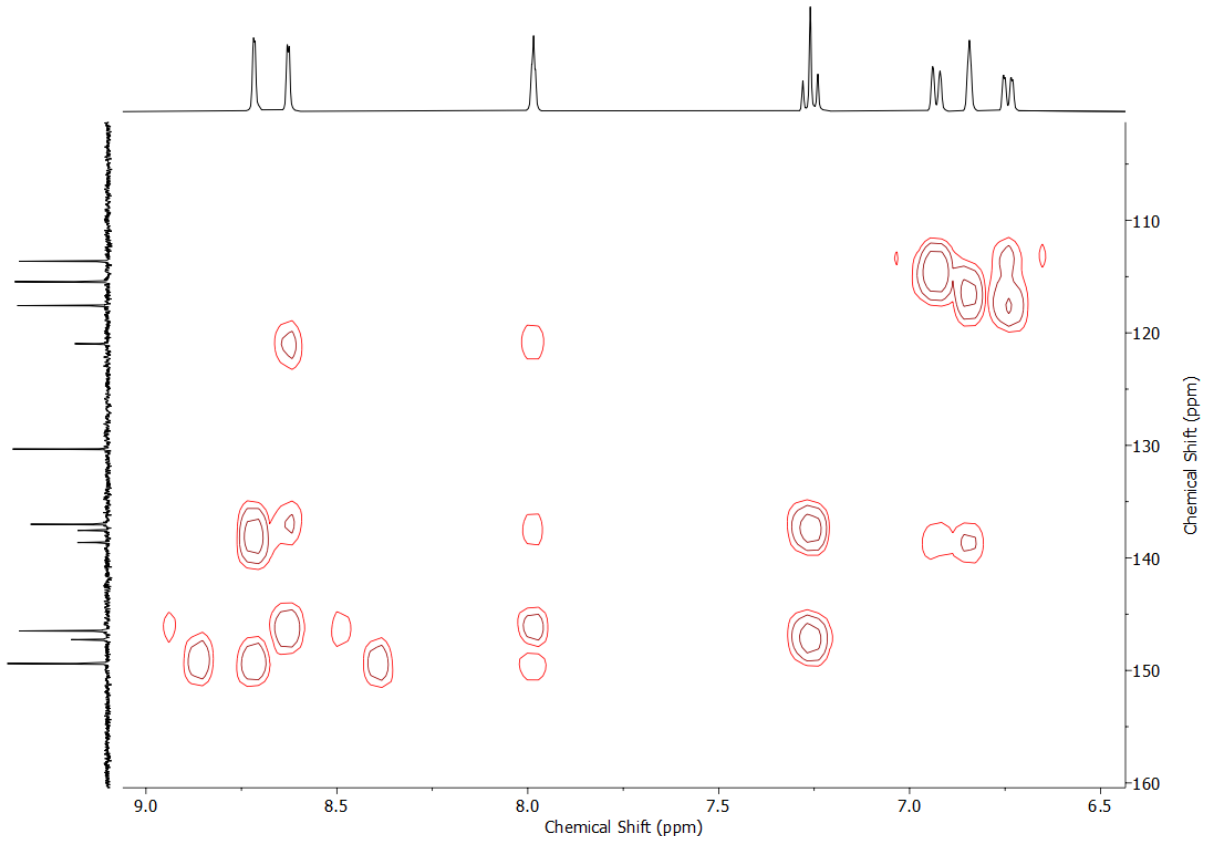

Figure S107 HMBC NMR ($CDCl_3$) of **S10**.

Synthesis of S11

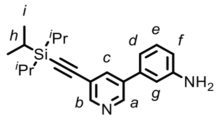

S10 (1.25 g, 5.0 mmol, 1.0 eq.), triisopropylsilylacetylene (1.35 mL, 6.0 mmol, 1.2 eq.), $Pd(PPh_3)_2Cl_2$ (0.070 g, 0.10 mmol, 2 mol%) and CuI (0.048 g, 0.25 mmol, 5 mol%) in 1:1 1,4-dioxane/$^iPr_2NH$ (20 mL) were stirred at 80 °C in a sealed vial for 20 h. EDTA solution (50 mL) was added to the cooled reaction mixture and extracted with CH_2Cl_2 (3 × 25 mL), dried ($MgSO_4$) and the solvent removed *in vacuo*. After purification by column chromatography on silica gel (15:85 EtOAc/pentane) the product was obtained as a brown oil (1.74 g, 99%).

^{1}H NMR (500 MHz, CD_3OD) δ: 8.71 (br. s, 1H, H_a/H_b), 8.54 (br. s, 1H, H_a/H_b), 7.98 (s, 1H, H_c), 7.21 (app. t, *J* = 7.9 Hz, 1H, H_e), 6.97 (t, *J* = 1.9 Hz, 1H, H_g), 6.92 (ddd, *J* = 7.6, 1.7, 0.9 Hz, 1H, H_d/H_f), 6.77 (ddd, *J* = 8.0, 2.3, 0.9 Hz, 1H, H_d/H_f), 1.16 (app. s, 21H, H_h, H_i).

^{13}C NMR (126 MHz, CD_3OD) δ: 151.0 (C_a/C_b), 149.9, 147.7 (C_a/C_b), 138.4, 138.3, 131.0 (C_e), 117.5 (C_d/C_f), 116.7 (C_d/C_f), 114.6 (C_g), 104.5, 96.0, 19.0 (C_i), 12.4 (C_h) (2 × 4° signals missing).

HR-EI-MS *m/z* = 350.2181 $[M]^+$ calc. 350.2173.

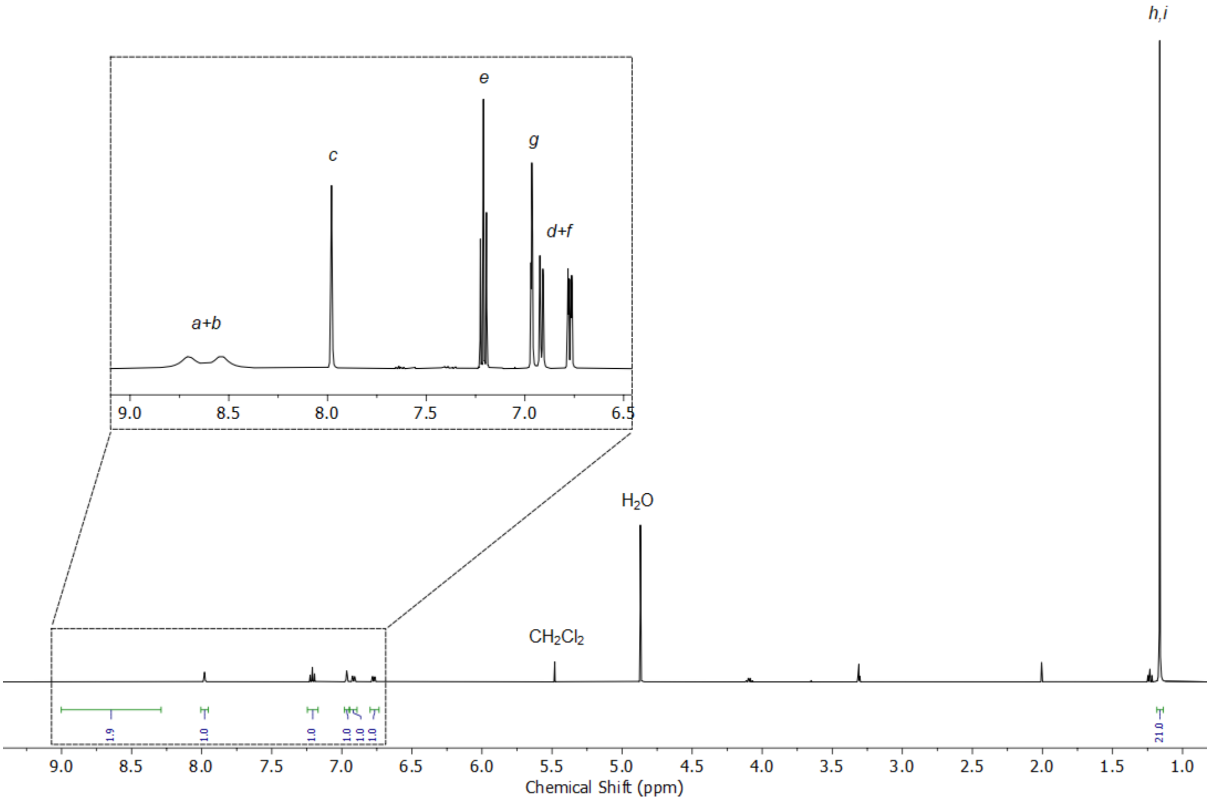

Figure S108 ^{1}H NMR (500 MHz, CD_3OD) of **S11**.

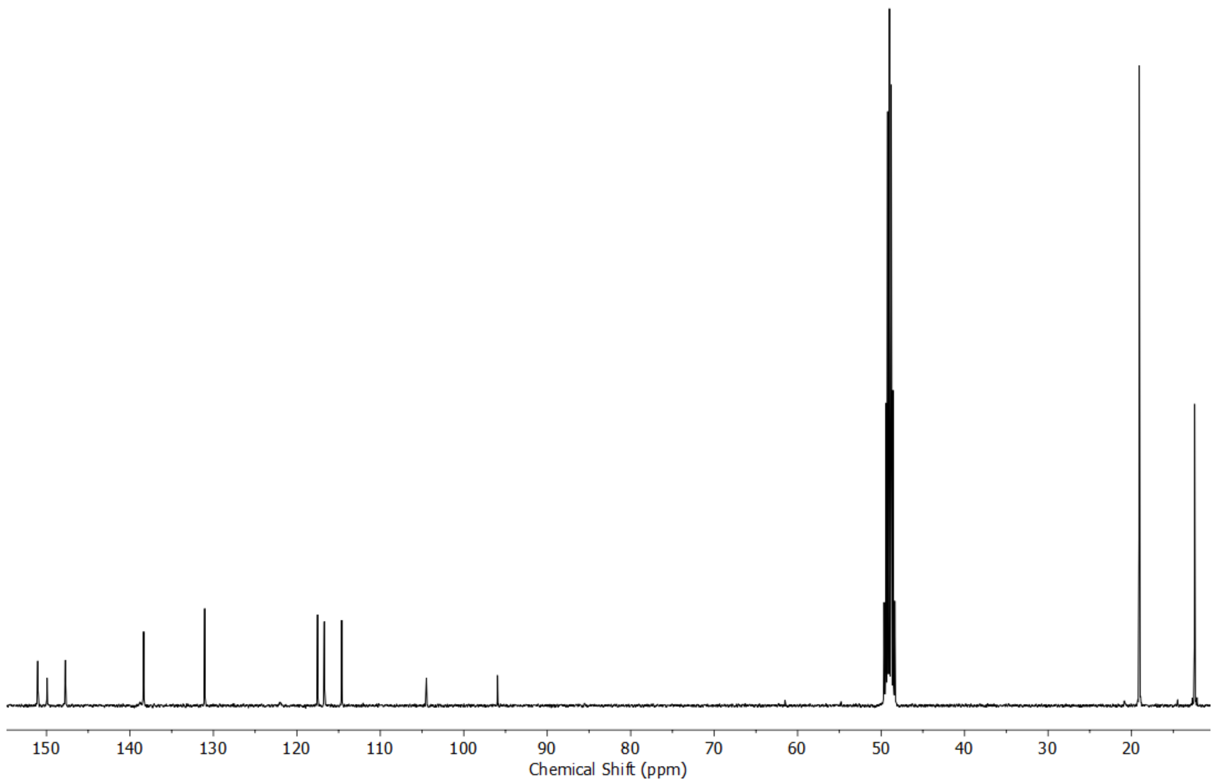

Figure S109 ^{13}C NMR (126 MHz, CD_3OD) of **S11**.

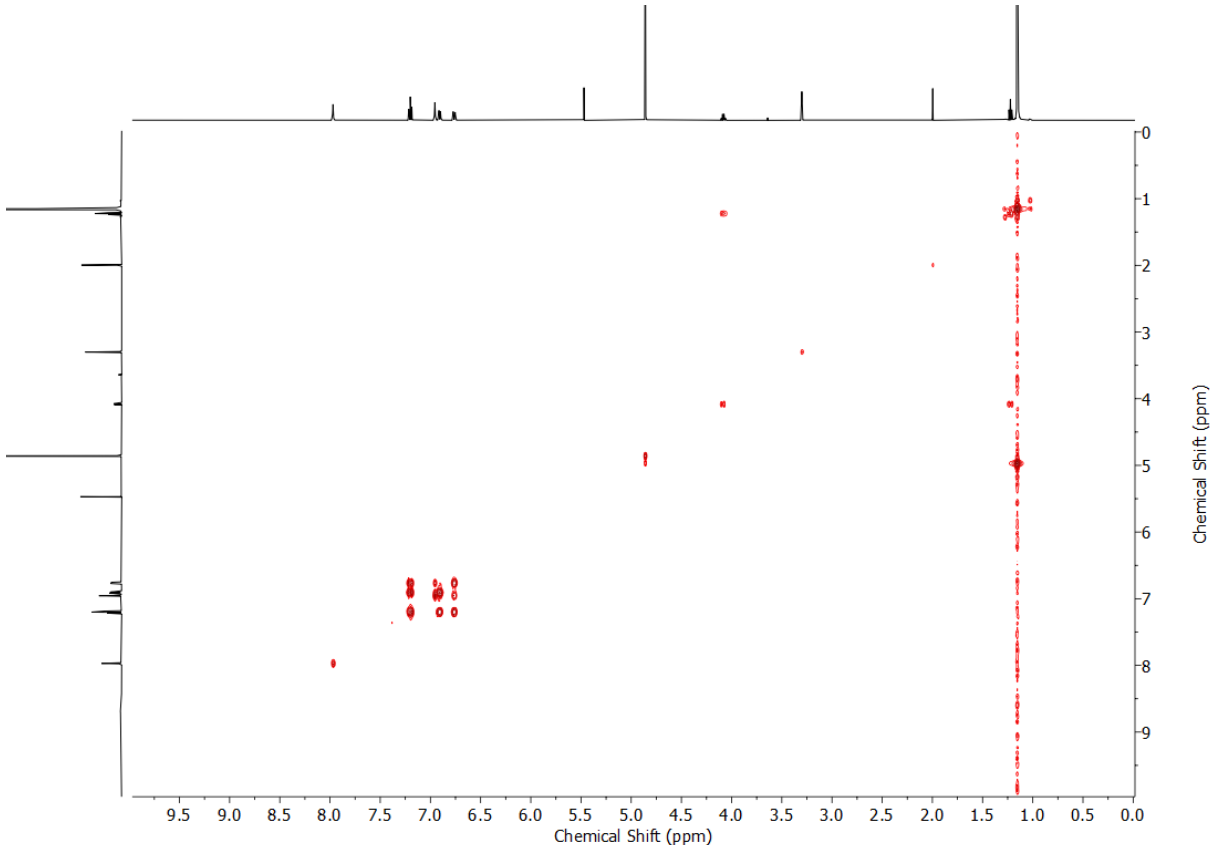

Figure S110 COSY NMR (CD_3OD) of **S11**.

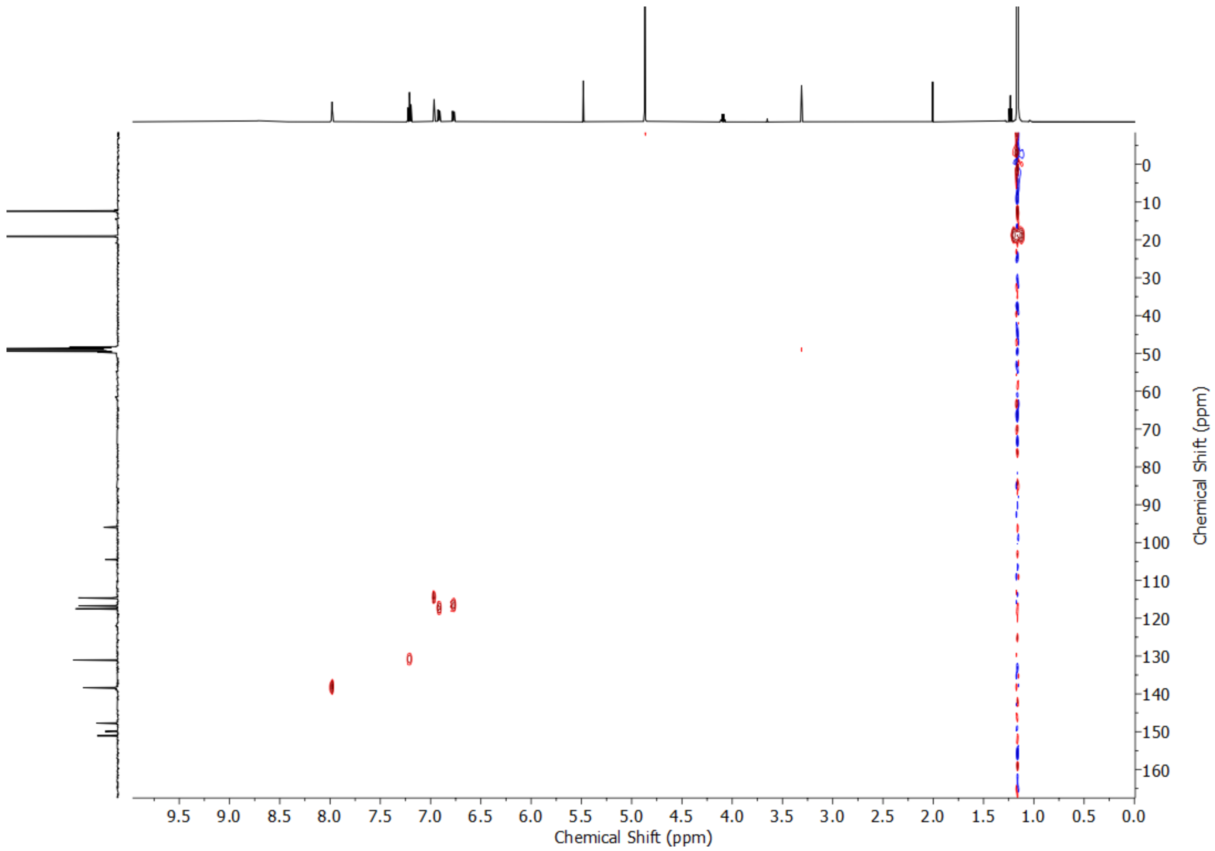

Figure S111 HSQC NMR (CD_3OD) of **S11**.

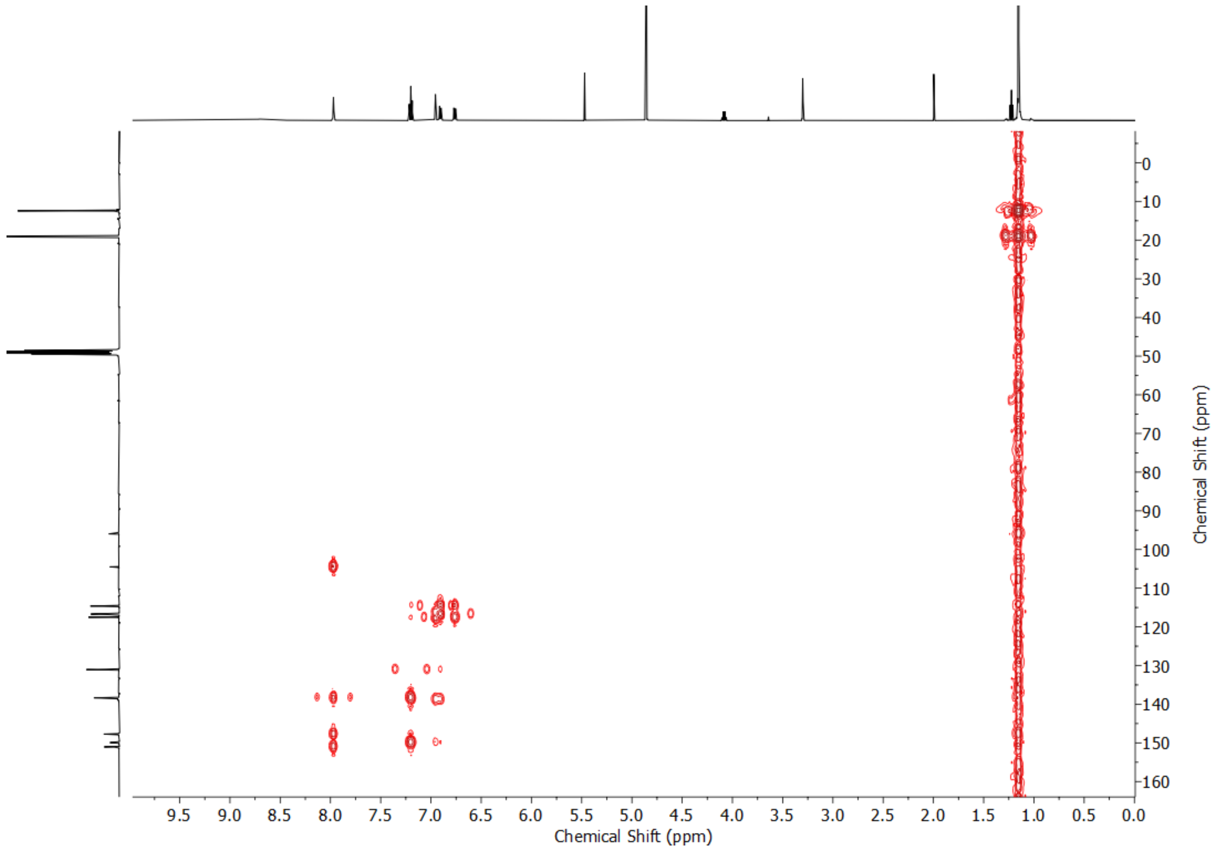

Figure S112 HMBC NMR (CD_3OD) of **S11**.

Synthesis of S12

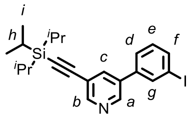

To a solution of **S11** (1.72 g, 4.91 mmol, 1.0 eq) in CH_3CN (30 mL) was added $TsOH{\cdot}H_2O$ (2.80 g, 14.7 mmol, 3.0 eq.). The resultant suspension was cooled to 0 °C and $NaNO_2$ (0.677 g, 9.81 mmol, 2.0 eq) and KI (2.04 g, 12.3 mmol, 2.5 eq.) in H_2O (3 mL) was added dropwise. The reaction mixture was allowed to warm to rt and stirred for 17 h. H_2O (50 mL), sat. aq. $NaHCO_3$ (50 mL) and 0.5 M $Na_2S_2O_3$ (50 mL) were added and extracted with EtOAc (3 × 50 mL). The combined organic phases were dried ($MgSO_4$) and the solvent removed *in vacuo*. After purification by column chromatography on silica gel (5:95 EtOAc/pentane) the product was obtained as a yellow oil (1.56 g, 69%).

^{1}H NMR (400 MHz, $CDCl_3$) δ: 8.69 (br. s, 2H, H_a, H_b), 7.92-7.90 (m, 2H, H_c, H_g), 7.76 (ddd, J = 7.9, 1.7, 1.0 Hz, 1H, H_d/H_f), 7.53 (ddd, J = 7.8, 1.8, 1.0 Hz, 1H, H_d/H_f), 7.22 (app. t, J = 7.8 Hz, 1H, H_e), 1.15-1.14 (m, 21H, H_h, H_i).

^{13}C NMR (101 MHz, $CDCl_3$) δ: 151.5 (C_a/C_b), 146.6 (C_a/C_b), 139.2, 137.6 (C_c/C_d/C_f/C_g), 137.5 (C_c/C_d/C_f/C_g), 136.2 (C_c/C_g), 135.0, 130.9 (C_e), 126.6 (C_d/C_f), 121.0, 103.0, 96.0, 95.1, 18.8 (C_i), 11.4 (C_h).

HR-EI-MS m/z = 461.1032 $[M]^+$ calc. 461.1030.

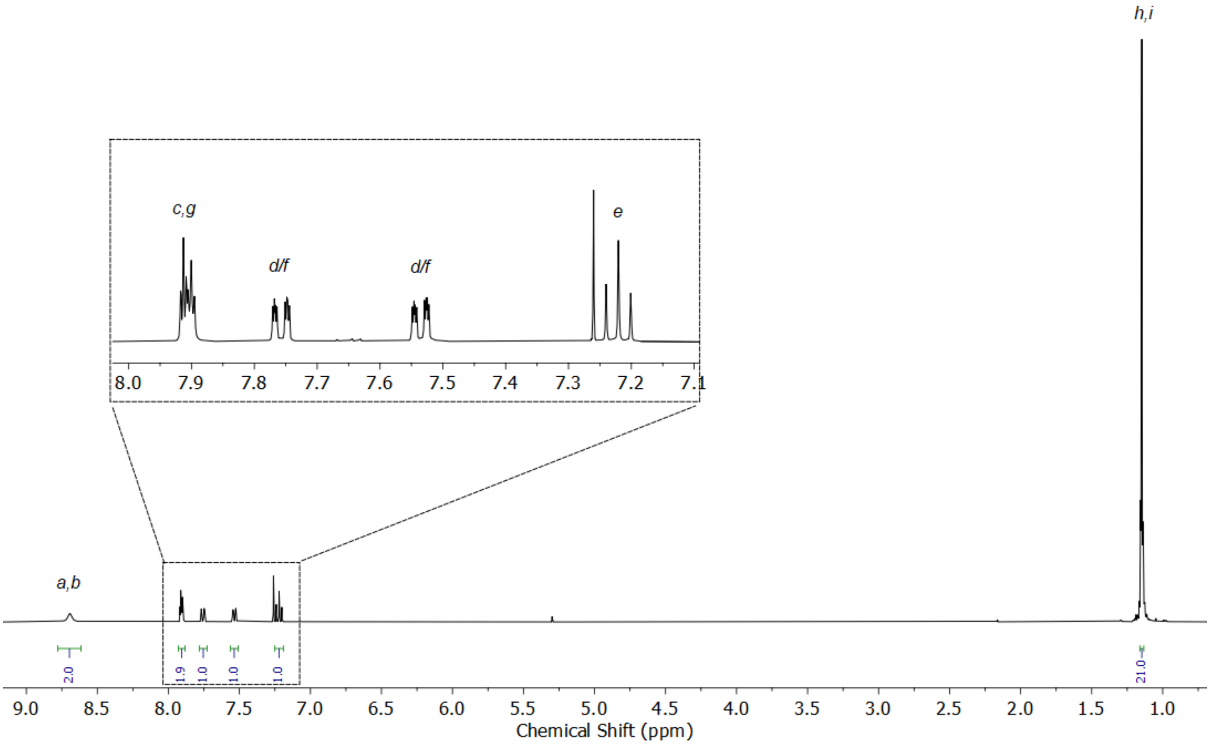

Figure S113 ^{1}H NMR (400 MHz, $CDCl_3$) of **S12**.

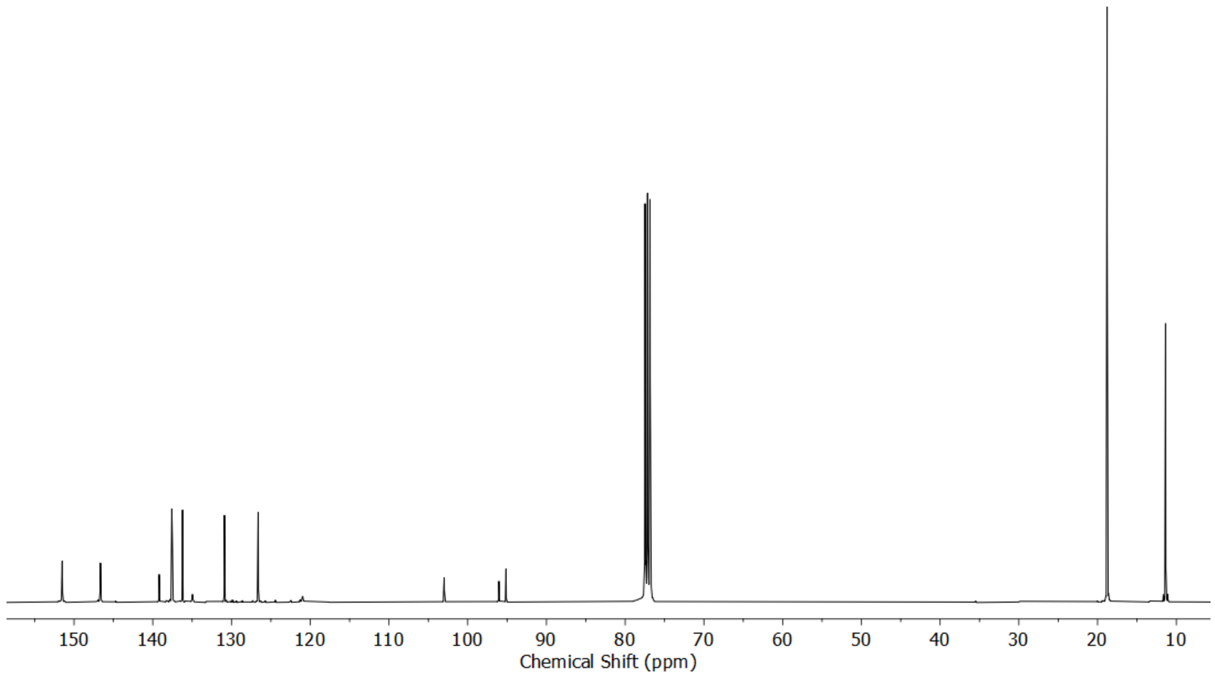

Figure S114 ^{13}C NMR (101 MHz, $CDCl_3$) of **S12**.

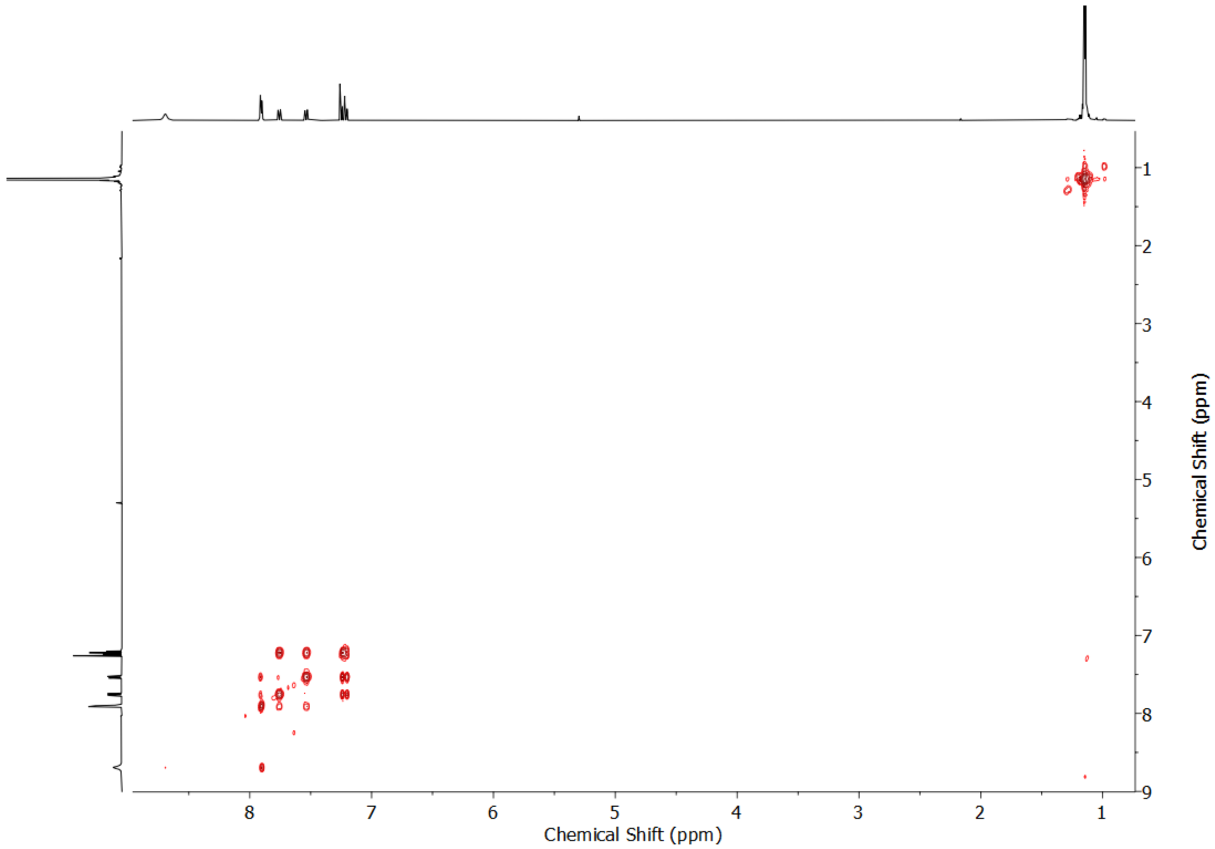

Figure S115 COSY NMR ($CDCl_3$) of **S12**.

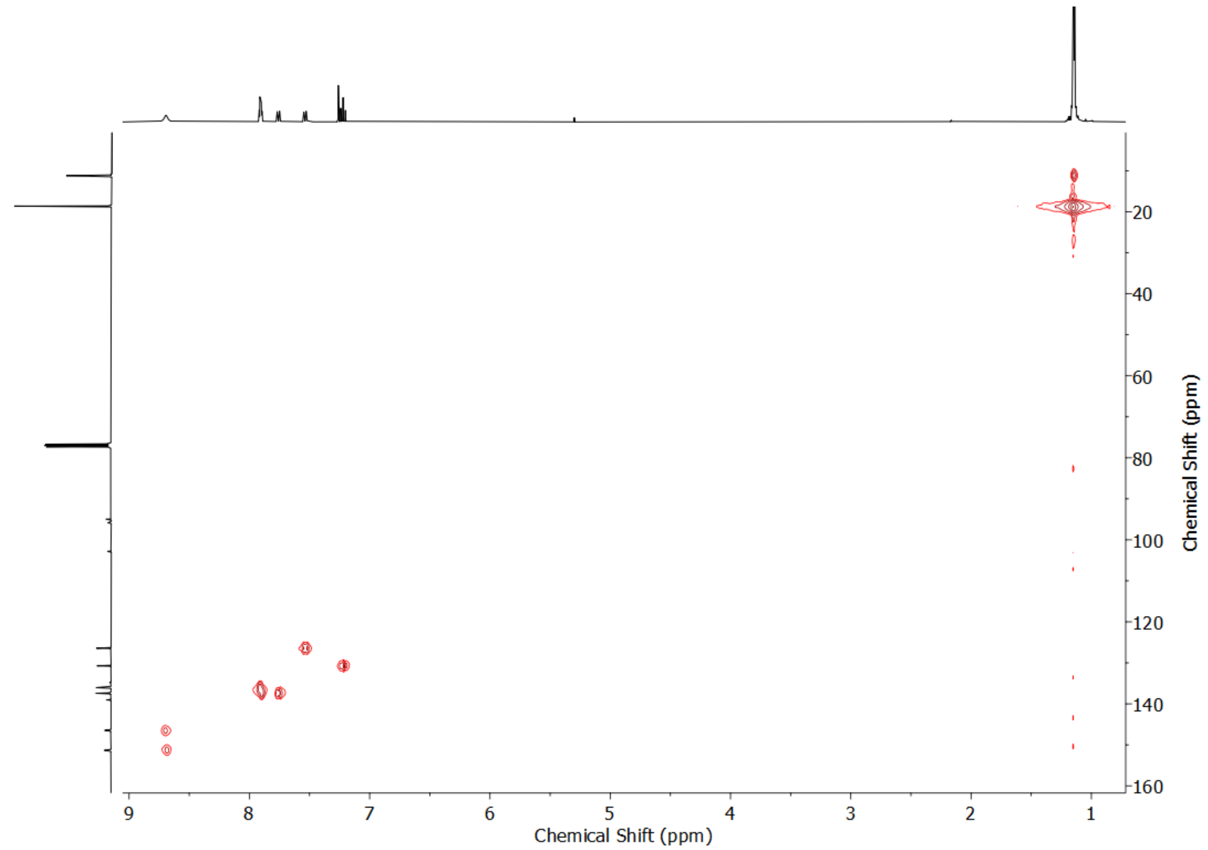

Figure S116 HSQC NMR ($CDCl_3$) of **S12**.

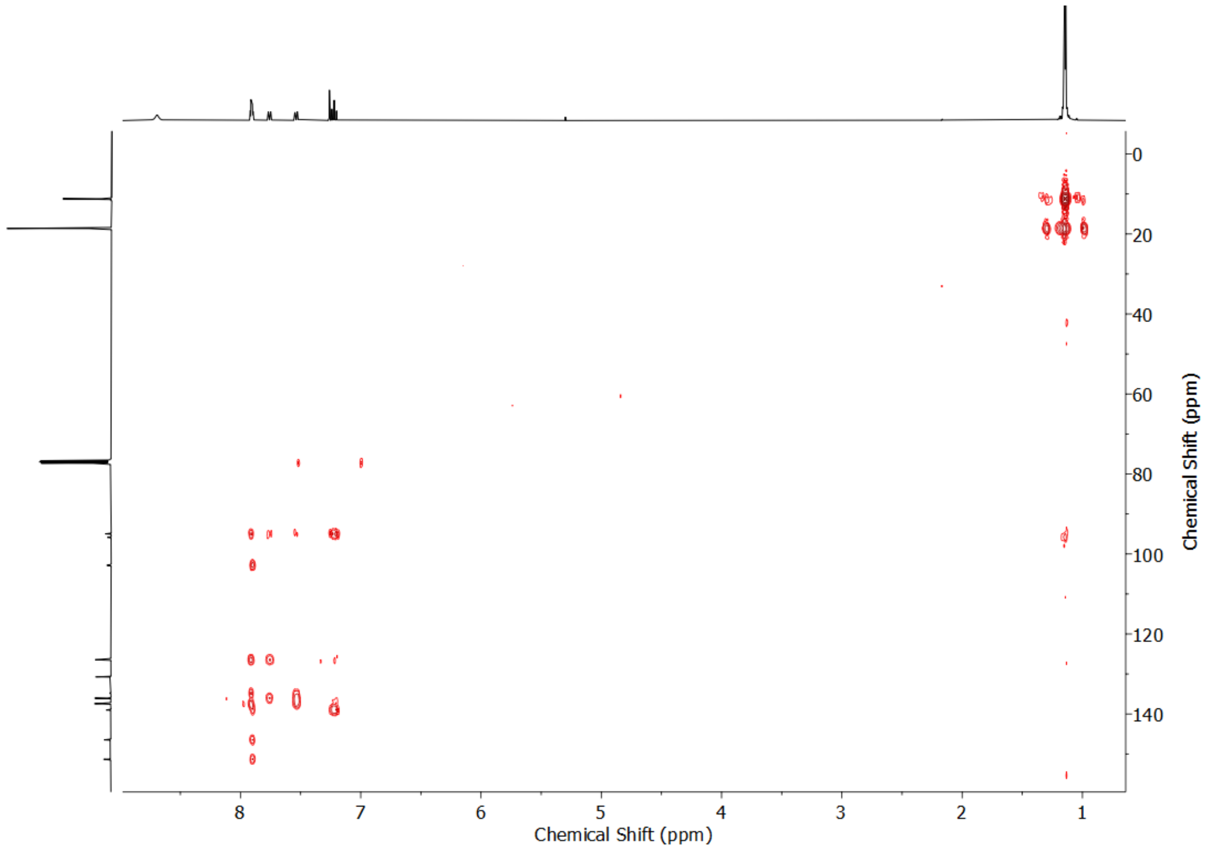

Figure S117 HMBC NMR ($CDCl_3$) of **S12**.

Synthesis of S13

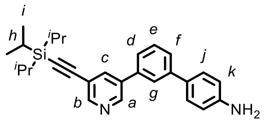

S12 (1.35 g, 2.93 mmol,1.0 eq.), 4-aminophenylboronic acid pinacol ester (0.802 g, 3.66 mmol, 1.25 eq.), $Pd(PPh_3)_2Cl_2$ (0.051 g, 0.073 mmol, 2.5 mol%) and K_2CO_3 (1.01 g, 7.33 mmol, 2.5 eq.) in 2:1 1,4-dioxane/H_2) (9.0 mL) were stirred at 90 °C in a sealed vial for 22 h. H_2O (50 mL) was added and the aqueous phase extracted with EtOAc (3 × 50 mL). The combined organic phases were dried ($MgSO_4$) and the solvent removed *in vacuo*. Following purification by column chromatography (1st column 0→3% acetone in 1:1 CH_2Cl_2/pentane; 2nd column 0→10% EtOAc in 1:1 CH_2Cl_2/pentane) the product was obtained as an orange/brown oil that solidified on standing (1.06 g, 85%).

^{1}H NMR (400 MHz, CD_3OD) δ: 8.79 (d, *J* = 1.8 Hz, 1H, H_a), 8.57 (d, *J* = 1.4 Hz, 1H, H_b), 8.09 (t, *J* = 2.1 Hz, 1H, H_c), 7.77 (m, 1H, H_g), 7.60 (m, 1H, H_f), 7.51-7.50 (m, 2H, H_d, H_e), 7.46 (d, *J* = 8.7 Hz, 2H, H_j), 6.81 (d, *J* = 8.7 Hz, 2H, H_k), 1.18 (app. s, 21H, H_h, H_i).

^{13}C NMR (101 MHz, CD_3OD) δ: 151.4 (C_b), 148.9, 148.0 (C_a), 143.9, 138.6 (C_c), 138.1, 131.3, 130.7 (C_d/C_e), 128.8 (C_j), 127.5 (C_f), 125.9 (C_d/C_e/C_g), 125.9 (C_d/C_e/C_g), 122.1, 116.8 (C_k), 104.4, 96.2, 19.1 (C_i), 12.5 (C_h) (one 4° signal missing).

HR-APCI-MS *m/z* = 427.2563 [M+H]$^+$ calc. 427.2564.

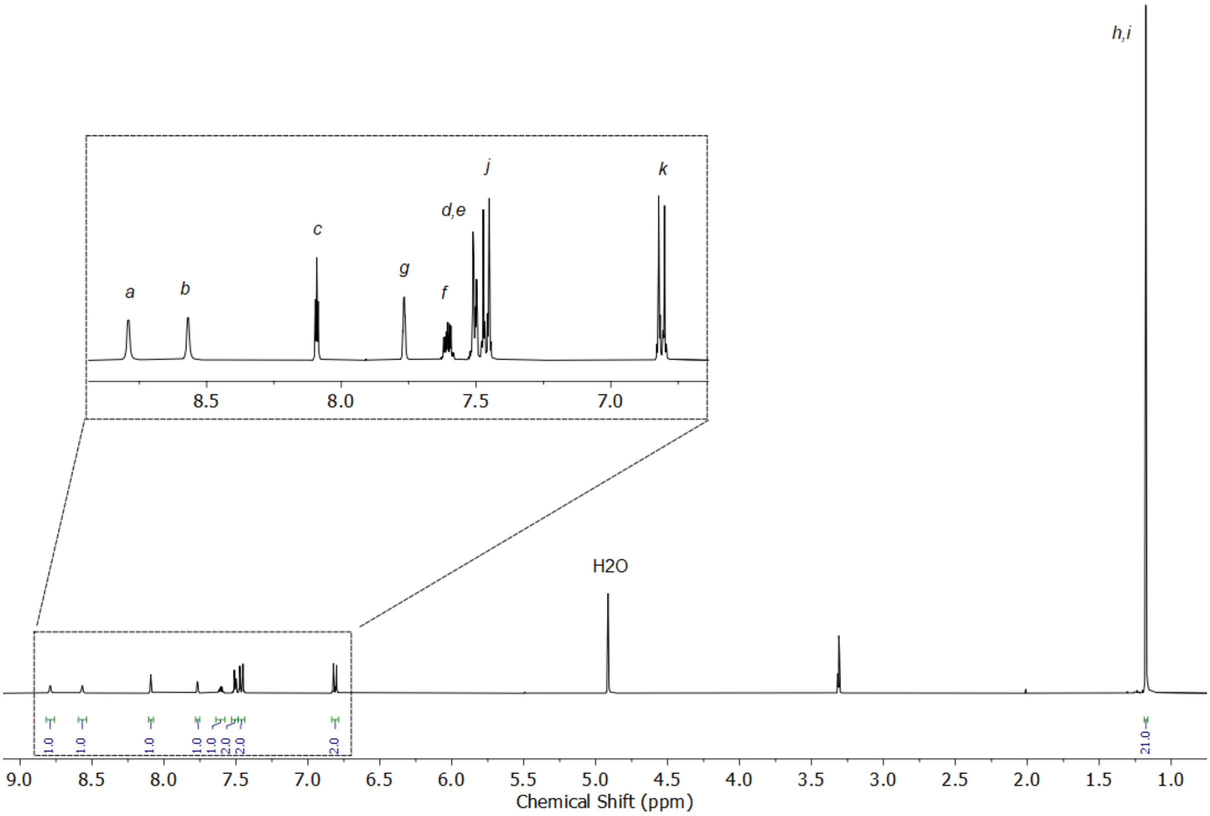

Figure S118 ^{1}H NMR (400 MHz, CD_3OD) of **S13**.

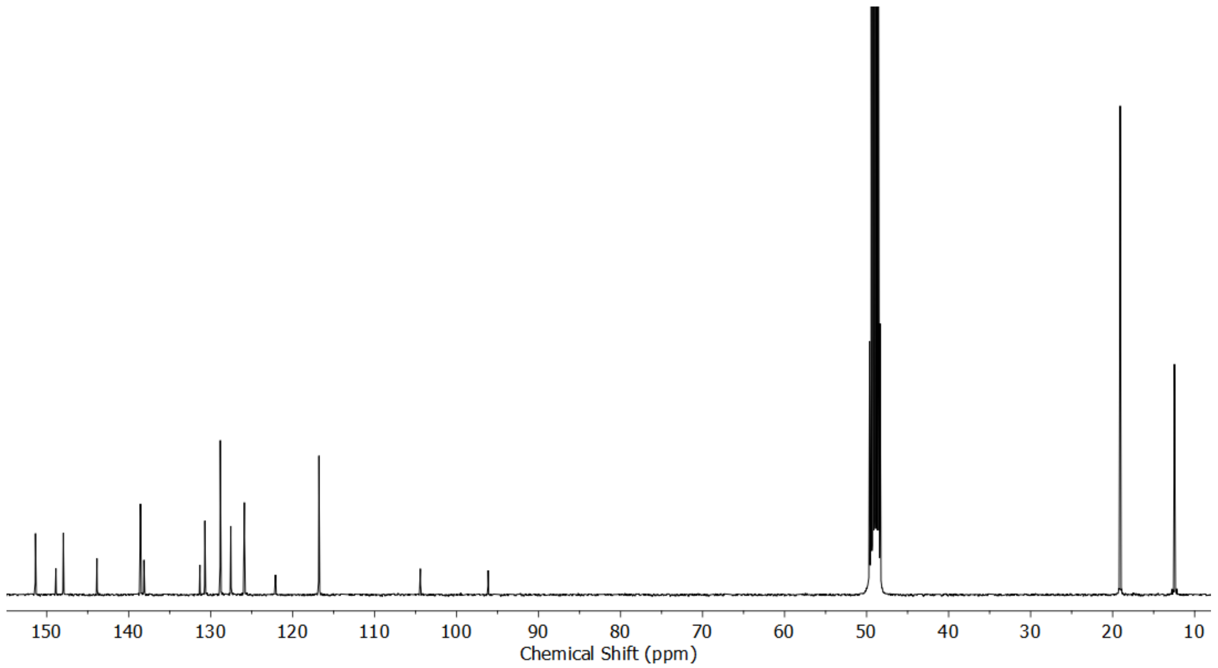

Figure S119 ^{13}C NMR (101 MHz, CD_3OD) of **S13**.

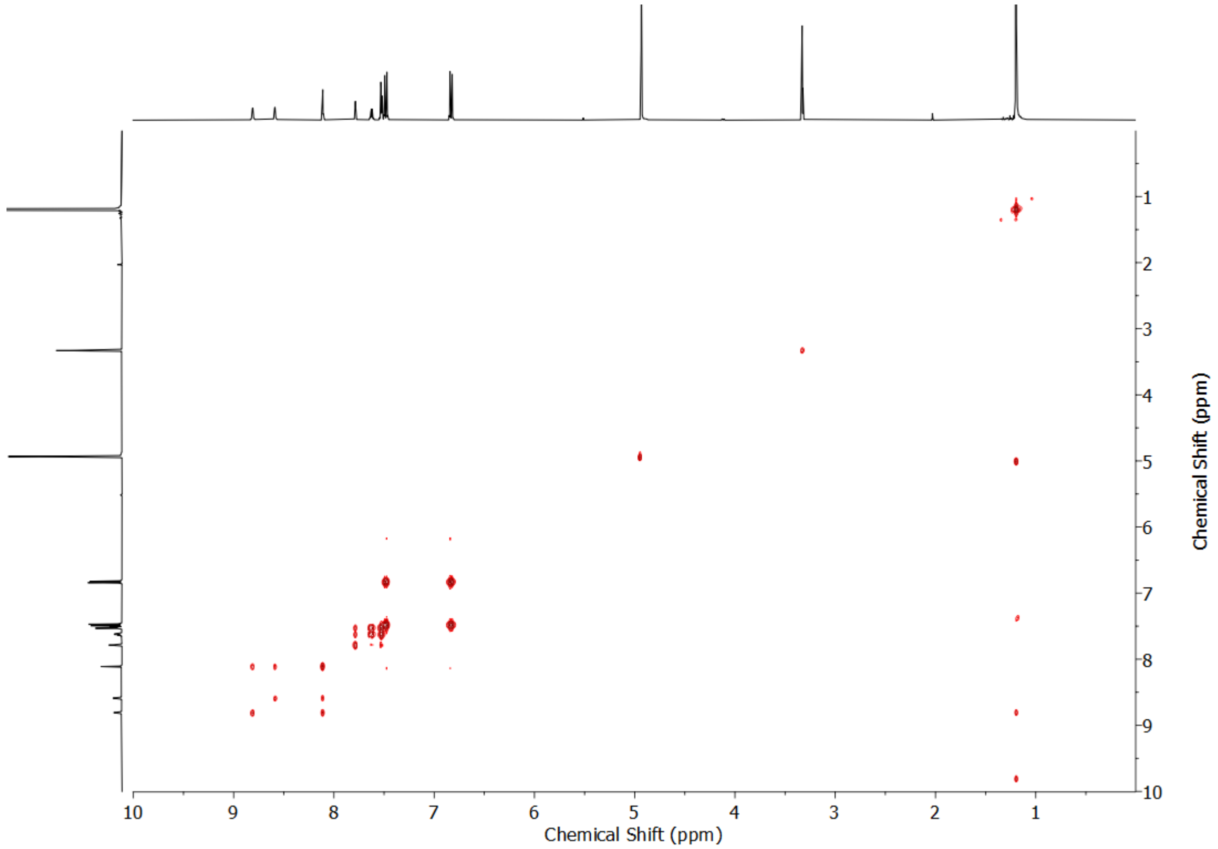

Figure S120 COSY NMR (CD_3OD) of **S13**.

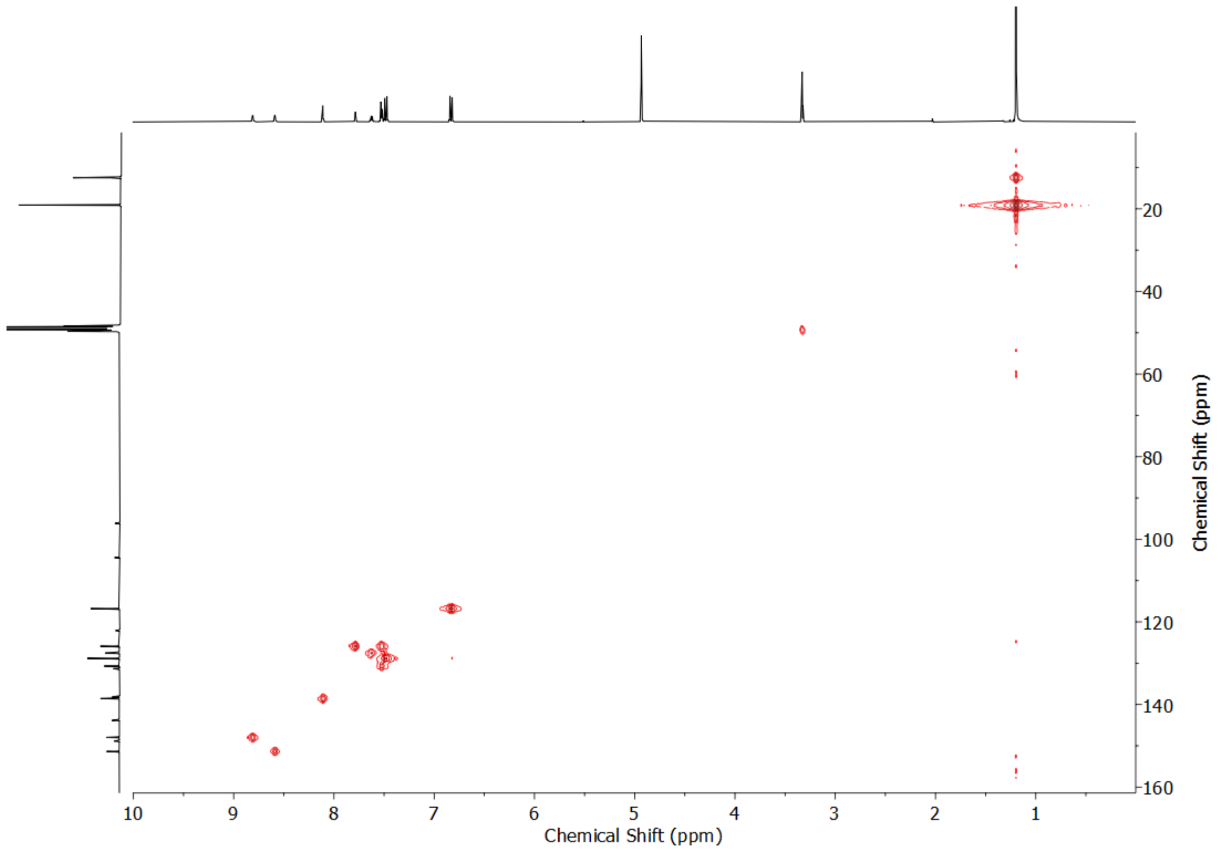

Figure S121 HSQC NMR (CD_3OD) of **S13**.

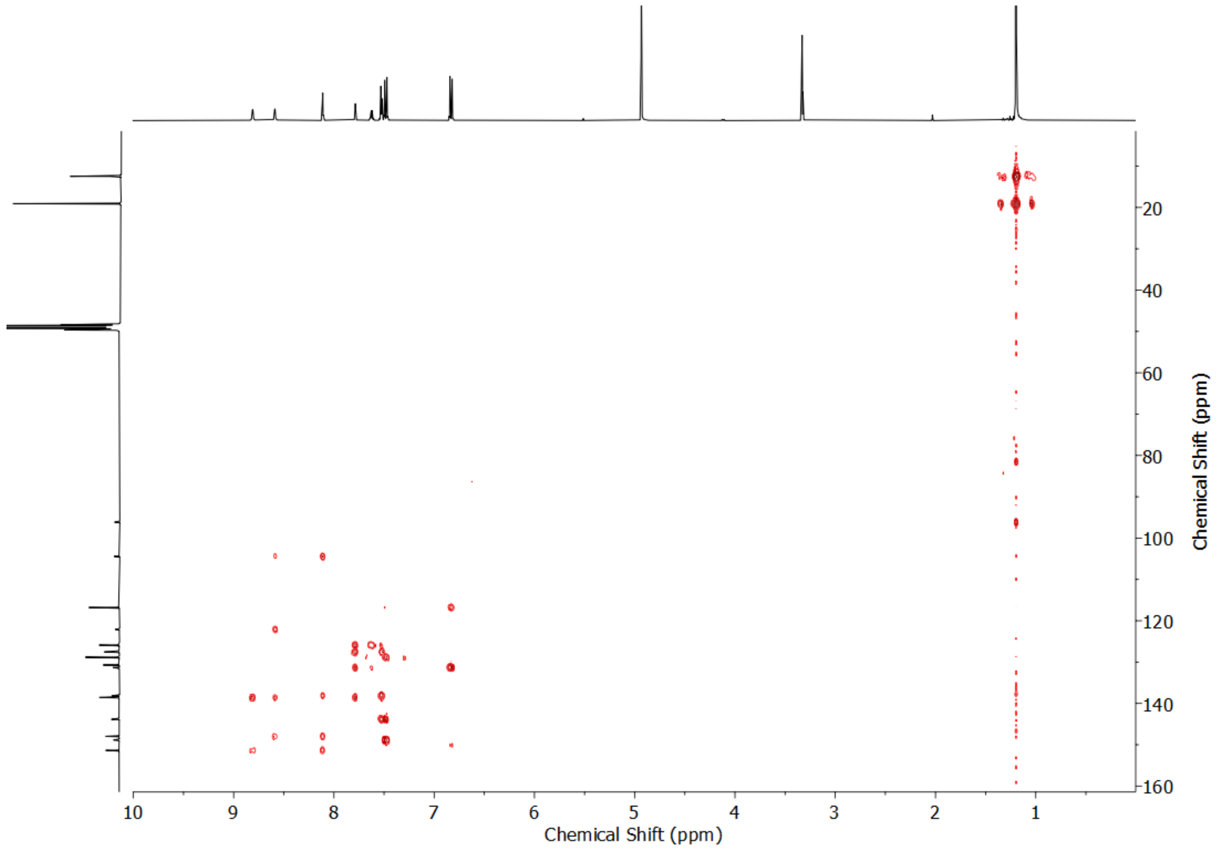

Figure S122 HMBC NMR (CD_3OD) of **S13**.

Synthesis of S14

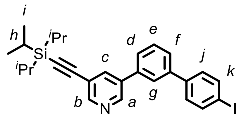

To a solution of **S13** (0.812 g, 1.90 mmol, 1.0 eq) in CH_3CN (19 mL) was added $TsOH{\cdot}H_2O$ (1.09 g, 5.71 mmol, 3.0 eq.). The resultant suspension was cooled to 0 °C and $NaNO_2$ (0.263 g, 3.81 mmol, 2.0 eq) and KI (0.790 g, 4.76 mmol, 2.5 eq.) in H_2O (2 mL) was added dropwise. The reaction mixture was allowed to warm to rt and stirred for 19 h. H_2O (50 mL), sat. aq. $NaHCO_3$ (50 mL) and 0.5 M $Na_2S_2O_3$ (50 mL) were added and the aqueous phase extracted with EtOAc (3 × 50 mL). The combined organic phases were dried ($MgSO_4$) and the solvent removed *in vacuo*. After purification by column chromatography on silica gel (1:1 CH_2Cl_2/pentane) the product was obtained as a light yellow oil (0.755 g, 74%).

^{1}H NMR (400 MHz, $CDCl_3$) δ: 8.80-8.72 (br. m, 2H, H_a, H_b), 7.99 (s, H_c), 7.81 (d, J = 8.6 Hz, 2H, H_j), 7.71 (m, 1H, H_g), 7.62-7.55 (m, 3H, H_d, H_e, H_f), 7.38 (d, J = 8.6 Hz, H_k), 1.16-1.15 (m, 21H, H_h, H_i).

^{13}C NMR (101 MHz, $CDCl_3$) δ: 151.0 (C_a/C_b), 146.6 (C_a/C_b), 141.3, 140.2, 138.2 (C_j), 137.8, 137.8, 129.9 (C_d/C_e/C_f), 129.2 (C_k), 127.2 (C_d/C_e/C_f), 126.7 (C_d/C_e/C_f), 126.0 (C_g), 103.1, 96.0, 93.7, 18.8 (C_i), 11.4 (C_h) (2 × 4° signals missing).

HR-APCI-MS *m/z* = 538.1412 [M+H]$^+$ calc. 538.1421.

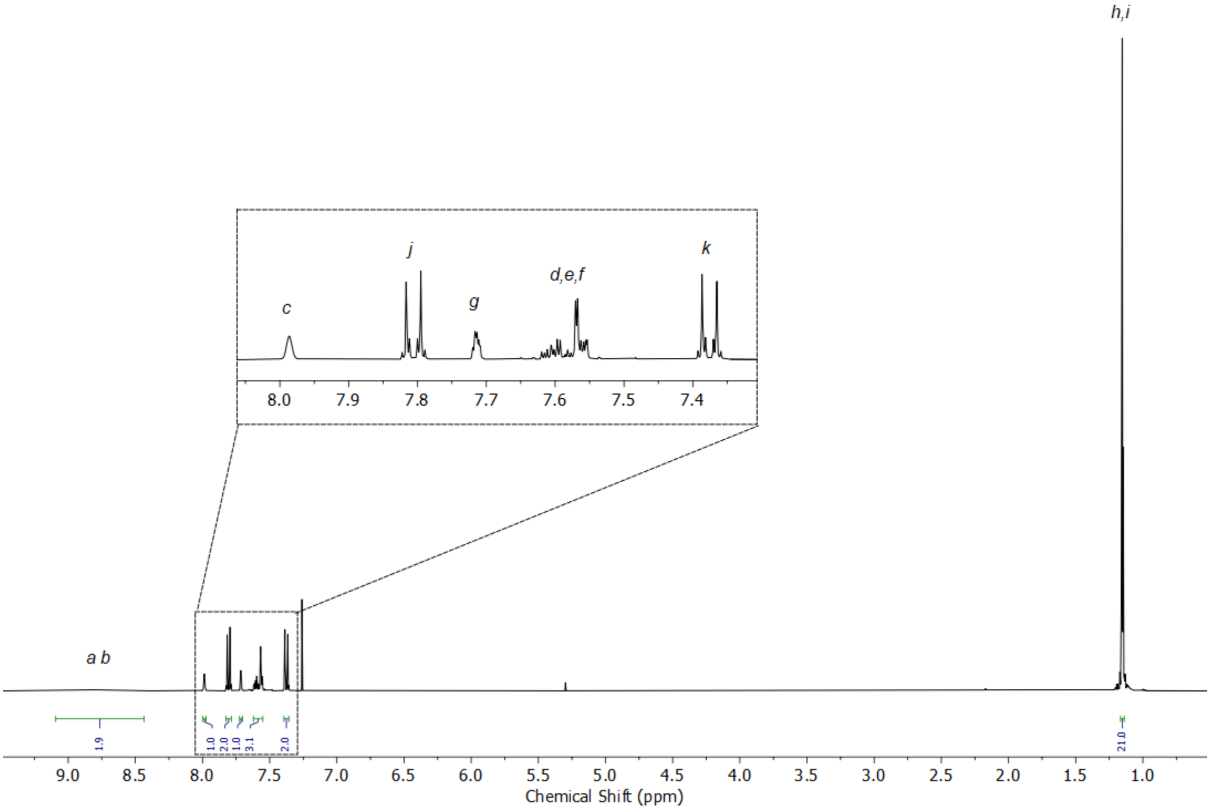

Figure S123 ^{1}H NMR (400 MHz, $CDCl_3$) of **S14**.

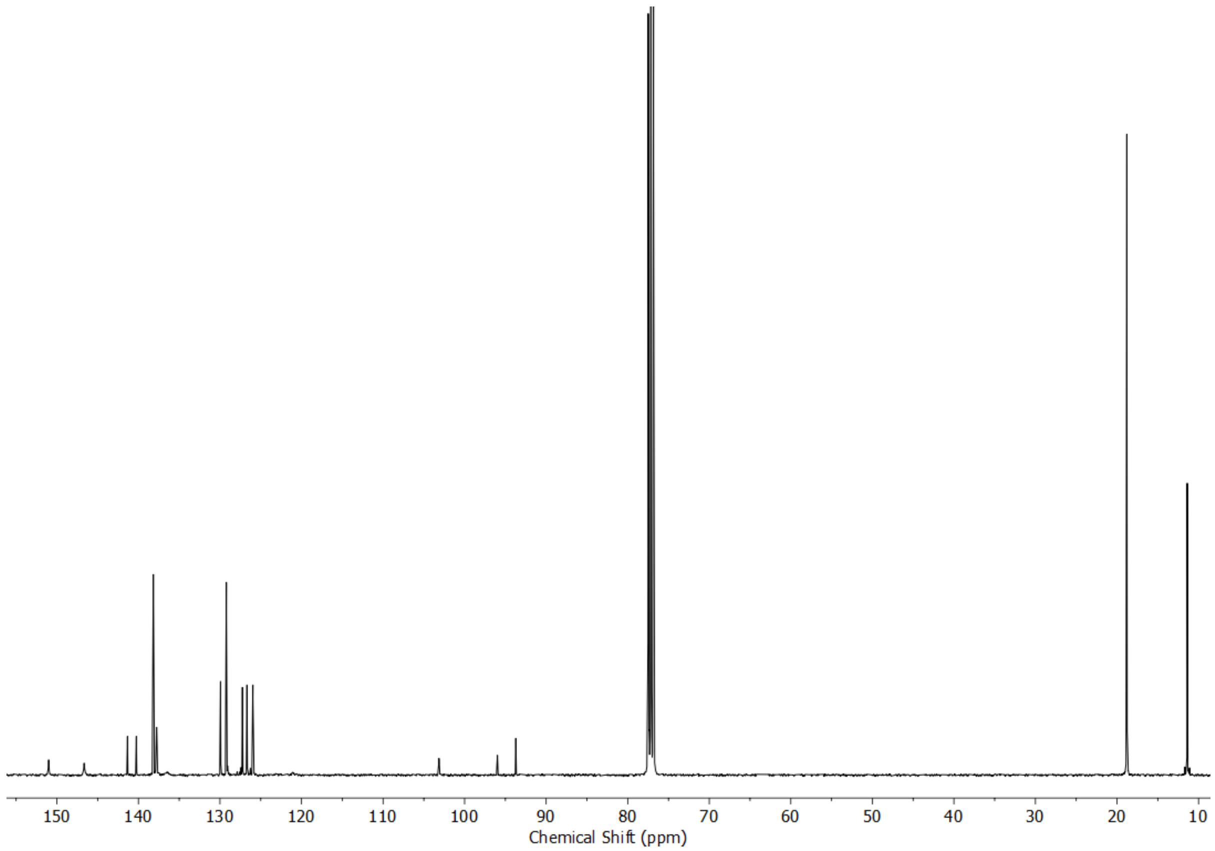

Figure S124 ^{13}C NMR (101 MHz, $CDCl_3$) of **S14**.

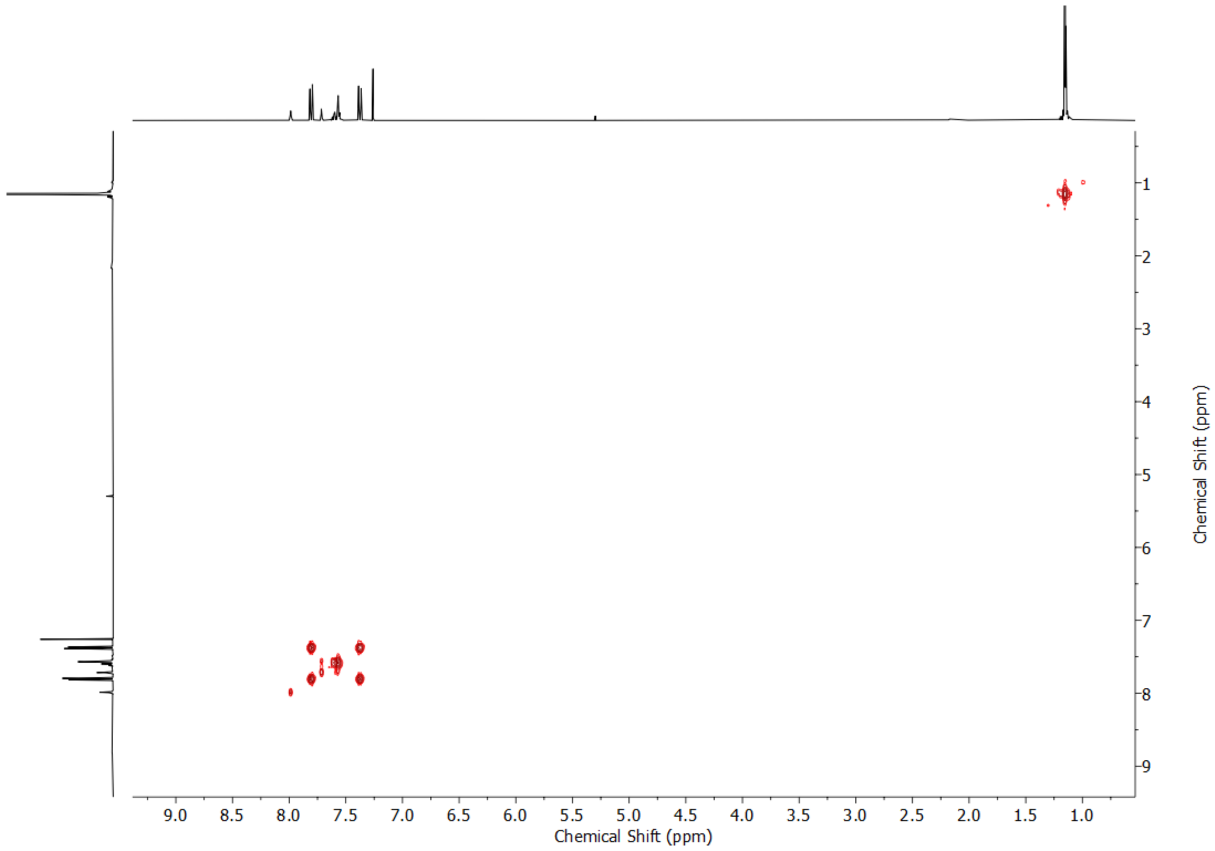

Figure S125 COSY NMR ($CDCl_3$) of **S14**.

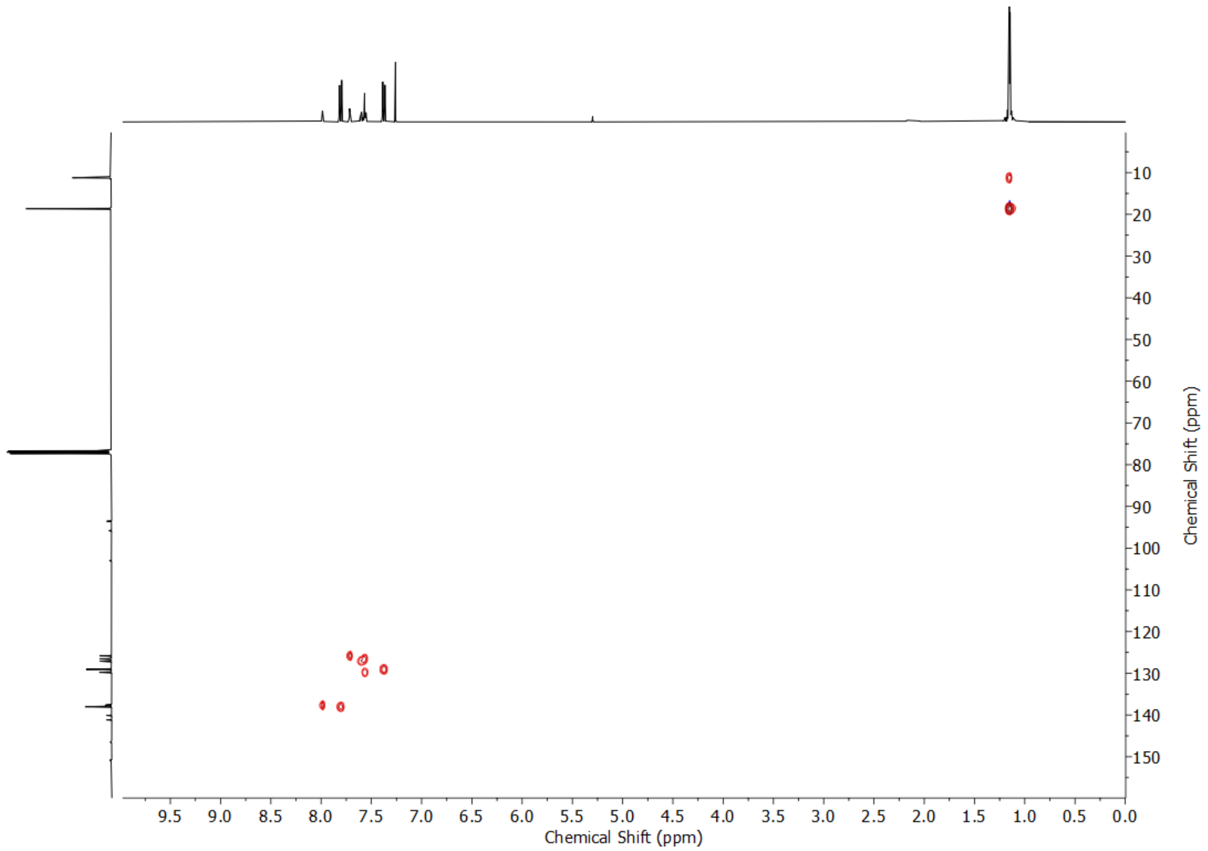

Figure S126 HSQC NMR ($CDCl_3$) of **S14**.

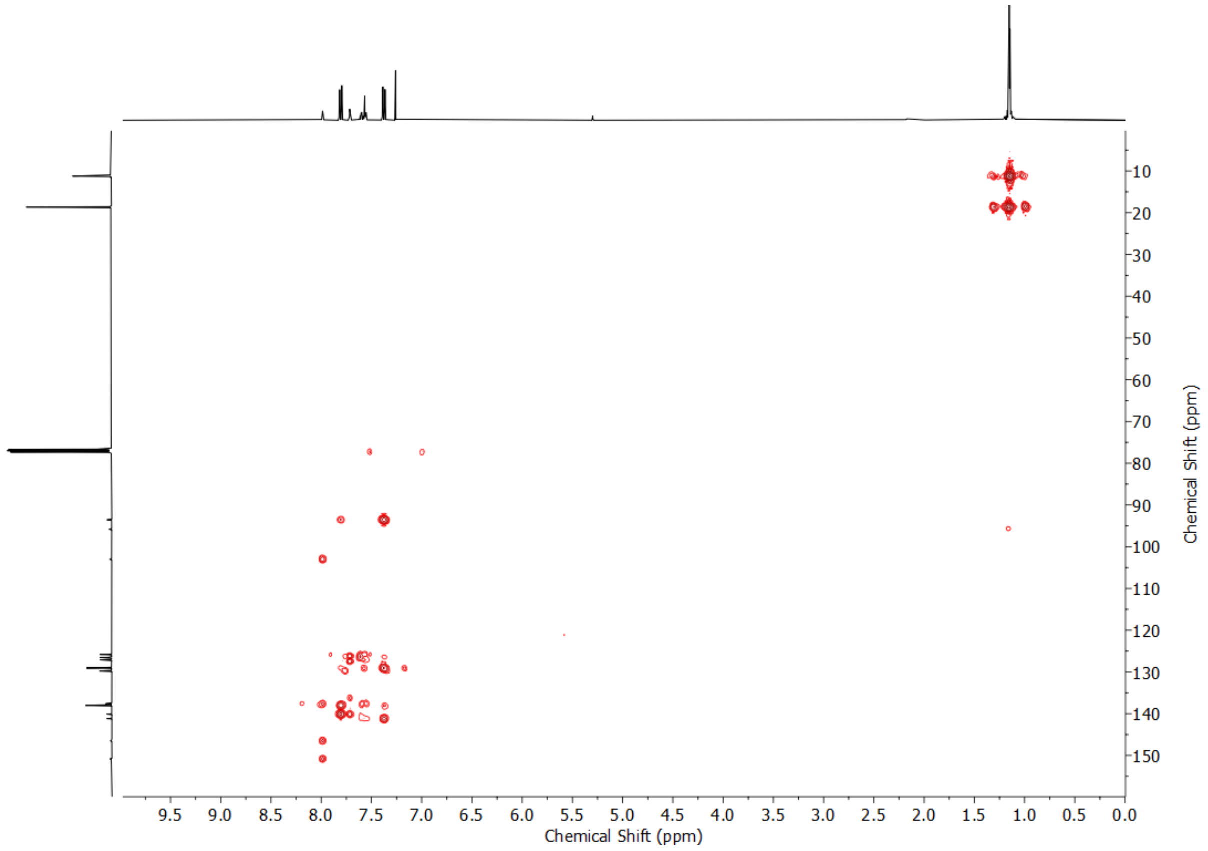

Figure S127 HMBC NMR ($CDCl_3$) of **S14**.

Synthesis of L1^{TIPS}

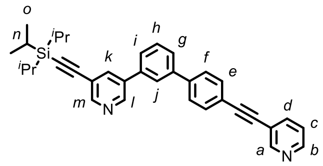

S14 (0.403 g, 0.75 mmol, 1.0 eq.), 3-ethynylpyridine (0.085 g, 0.83 mmol, 1.1 eq.), $Pd(PPh_3)_4$ (0.022 g, 0.019 mmol, 2.5 mol%) and CuI (0.0036 g, 0.019 mmol, 2.5 mol%) in 1:1 1,4-dioxane/$^{i}Pr_2NH$ (7.5 mL) were stirred at rt for 22 h. EDTA solution (50 mL) was added and the aqueous phase extracted with CH_2Cl_2 (3 × 25 mL). The combined organic phases were dried ($MgSO_4$) and the solvent removed *in vacuo*. After purification by column chromatography on silica gel (pentane with step gradient 0 to 20% EtOAc in 5% increments) the product was obtained as an off-white solid (0.343 g, 89%).

^{1}H NMR (400 MHz, $CDCl_3$) δ: 8.80 (app. s, 2H, H_a, H_l), 8.70 (s, 1H, H_m), 8.57 (d, J = 4.8 Hz, 1H, H_b), 8.00 (br. s, 1H, H_k), 7.88 (m, 1H, H_d), 7.78 (s, 1H, H_j), 7.66 (app. s, 5H, H_e, H_f, H_h), 7.58 (app. d, J = 4.4 Hz, 2H, H_g, H_i), 7.34 (dd, J = 7.9, 5.0 Hz, 1H, H_c), 1.16 (app. s, 21 H, H_n, H_o).

^{13}C NMR (101 MHz, $CDCl_3$) δ: 151.9 (C_a), 151.2 (C_m), 148.2 (C_b), 146.9 (C_l), 141.4, 141.1, 139.1 (C_d), 137.8, 137.7 (C_k), 136.3, 132.4 (C_e/C_f), 129.9 (C_g/C_i), 127.4 (C_e/C_f), 127.4, 126.8 (C_g/C_i), 126.1 (C_j), 123.4 (C_c), 121.9, 120.9, 120.8, 103.2, 95.8, 92.9, 86.8, 18.8 (C_o), 11.4 (C_n).

HR-APCI-MS *m/z* = 513.2722 [M+H]$^{+}$ calc. 513.2721.

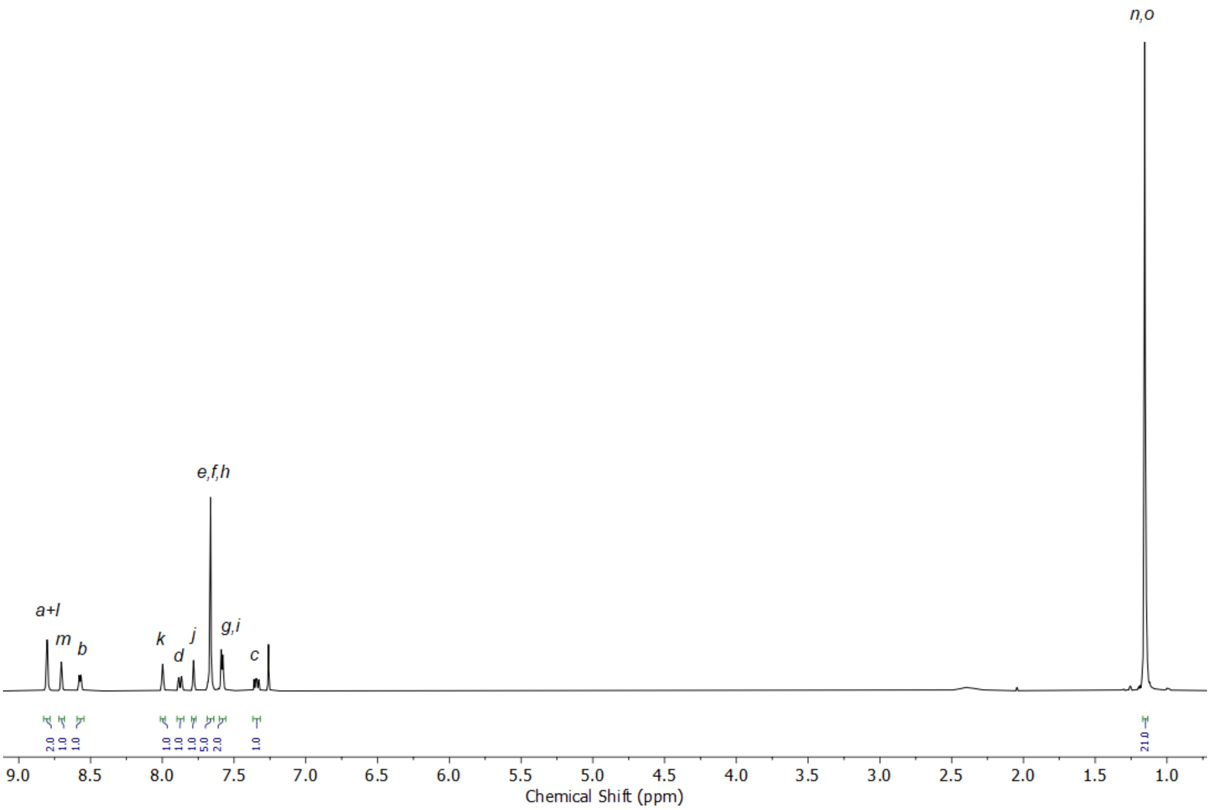

Figure S128 ^{1}H NMR (400 MHz, $CDCl_3$) of **L1^{TIPS}**.

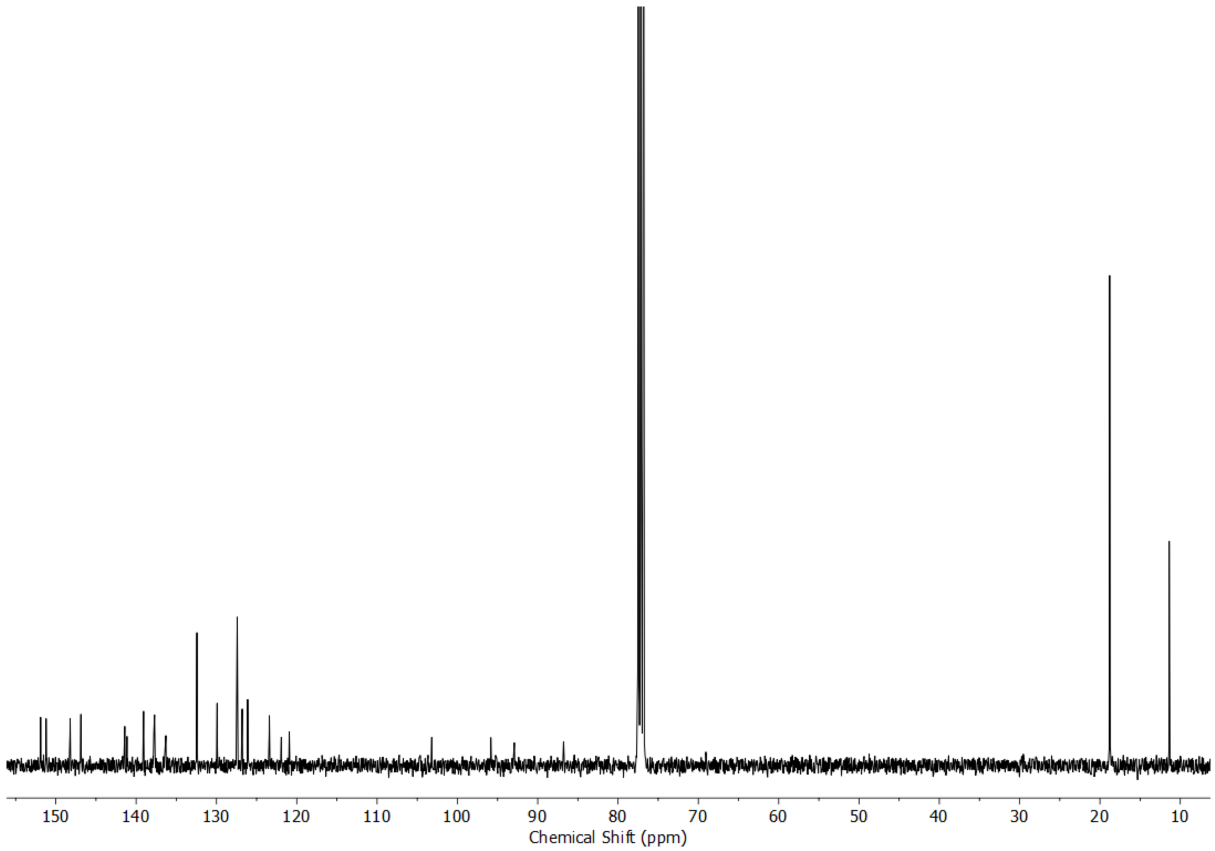

Figure S129 ^{13}C NMR (101 MHz, $CDCl_3$) of **L1**$^{\mathbf{TIPS}}$.

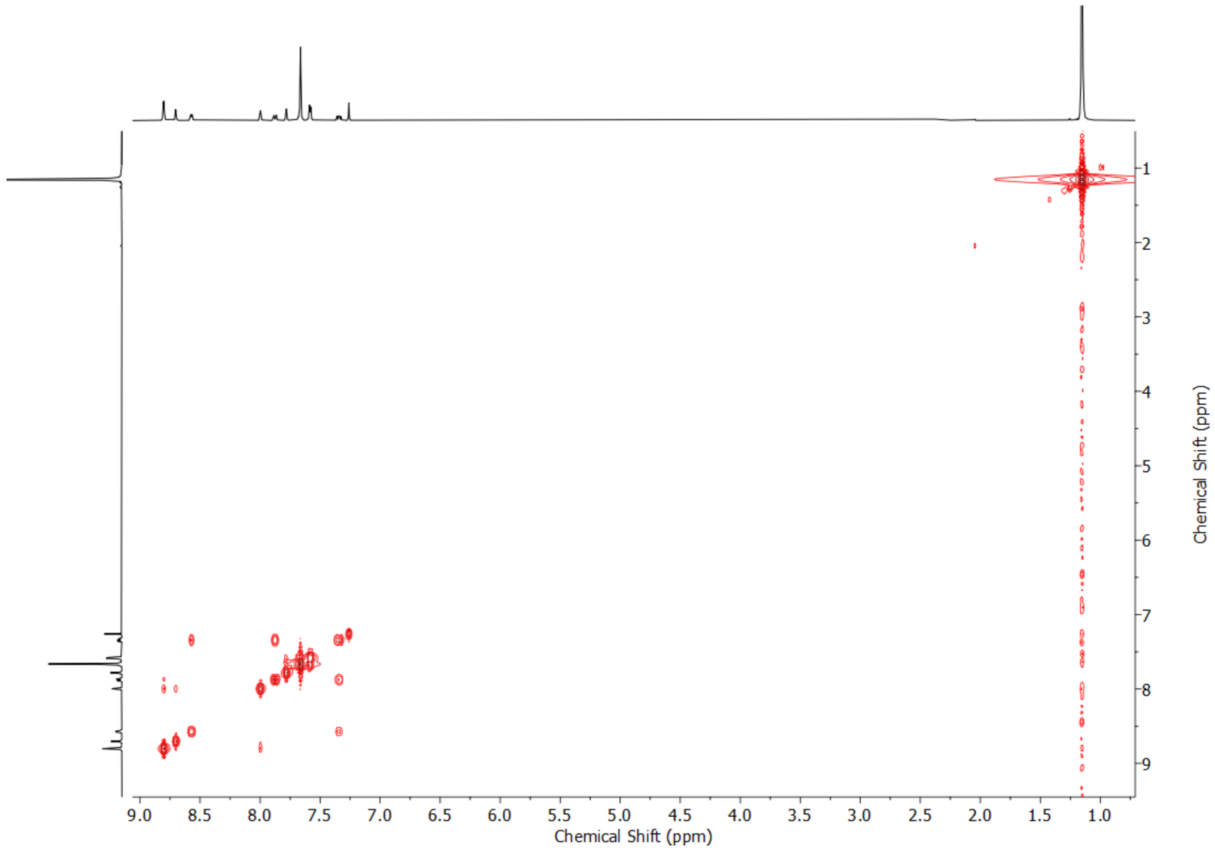

Figure S130 COSY NMR ($CDCl_3$) of **L1**$^{\mathbf{TIPS}}$.

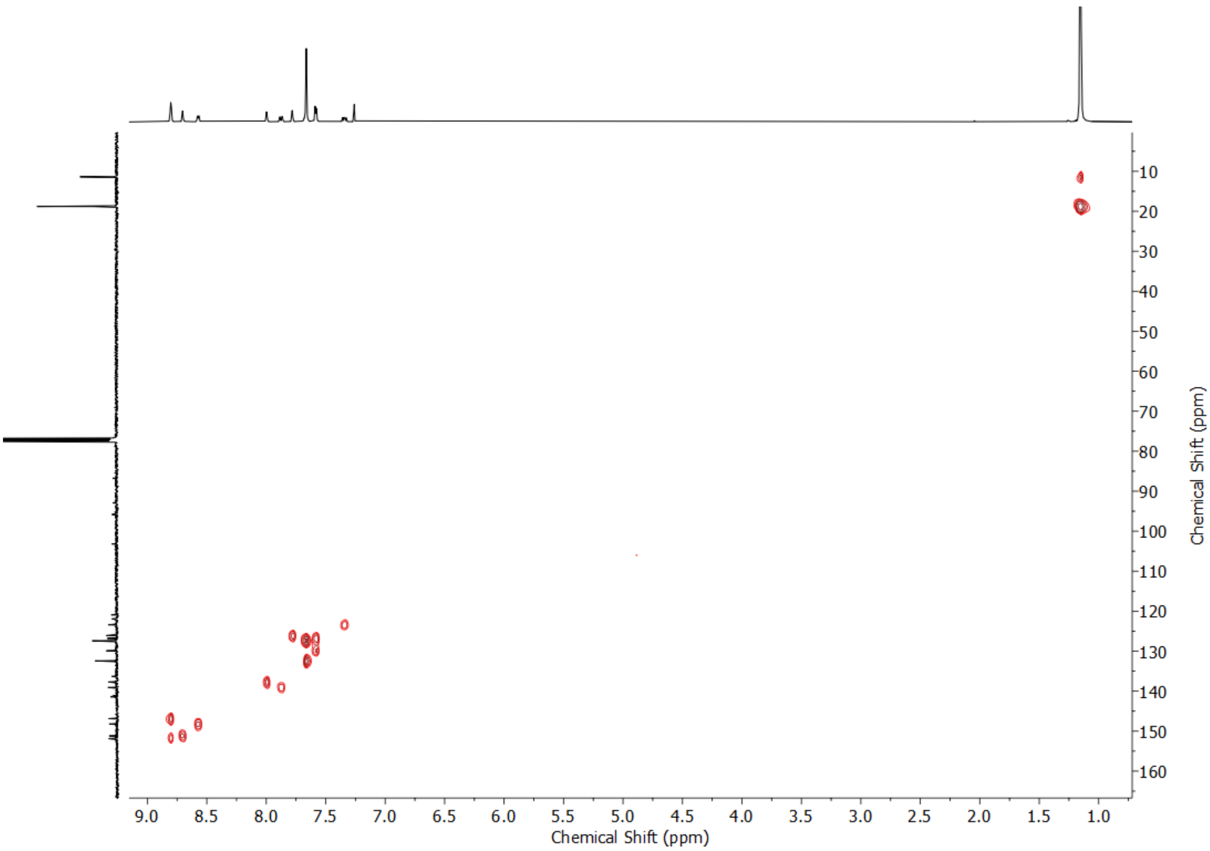

Figure S131 HSQC NMR ($CDCl_3$) of **L1**$^{\mathbf{TIPS}}$.

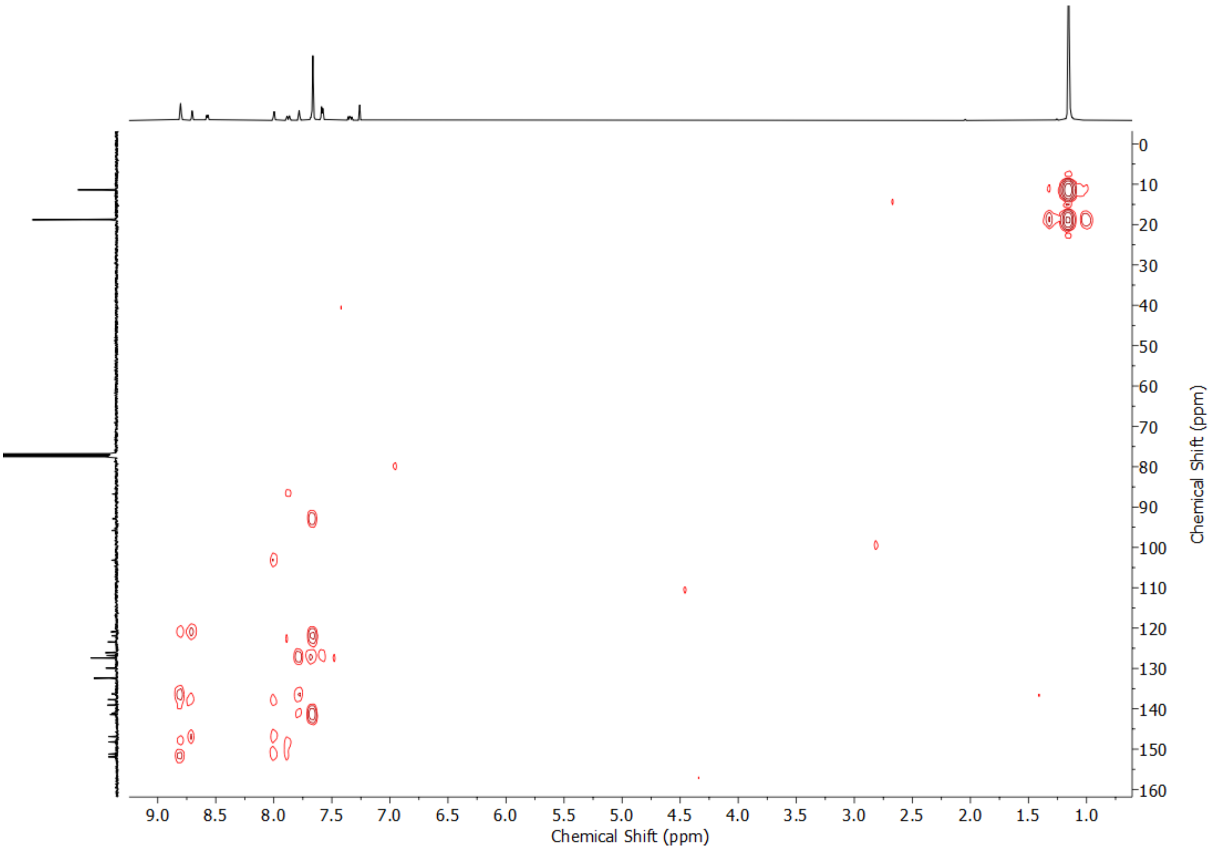

Figure S132 HMBC NMR ($CDCl_3$) of **L1**$^{\mathbf{TIPS}}$.

Synthesis of L1^{CCH}

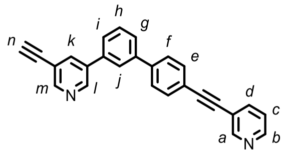

To a solution of **L1^{TIPS}** (0.256 g, 0.5 mmol, 1 eq.) in THF (5 mL) was added 1M TBAF in THF (1.0 mL, 1.0 mmol, 2 eq.). After stirring at rt for 2 h, sat. aq. NH_4Cl (20 mL) was added and the aqueous phase extracted with CH_2Cl_2 (3 × 20 mL). The combined organic phases were dried ($MgSO_4$) and the solvent removed *in vacuo*. After purification by column chromatography (1:9 acetone/CH_2Cl_2) the product was obtained as a white solid (0.175 g, 98%).

^{1}H NMR (400 MHz, $CDCl_3$) δ: 8.85 (s, 1H, H_l), 8.80 (s, 1H, H_a), 8.72 (s, 1H, H_m), 8.56 (d, J = 4.0 Hz, 1H, H_b), 8.03 (s, 1H, H_k), 7.84 (d, J = 7.8 Hz, 1H, H_d), 7.78 (s, 1H, H_j), 7.68-7.64 (m, 5H, H_e, H_f, 1 of H_g/H_h/H_i), 7.58-7.57 (m, 2H, 2 of H_g/H_h/H_i), 7.30 (dd, J = 7.8, 5.0 Hz, 1H, H_c), 3.27 (s, 1H, H_n).

^{13}C NMR (101 MHz, $CDCl_3$) δ: 152.4 (C_a), 151.6 (C_m), 148.8 (C_b), 147.9 (C_l), 141.4, 141.0, 138.6 (C_d), 137.7, 137.6 (C_k), 136.1, 132.4 (C_e/C_f), 129.9 (C_g/C_h/C_i), 127.4 (× 2, 1 of C_e/C_f, 1 of C_g/C_h/C_i), 126.7 (C_g/C_h/C_i), 126.1 (C_j), 123.2 (C_c), 122.0, 120.6, 119.4, 92.5, 87.0, 81.1 (C_n), 80.4.

HR-ESI-MS m/z = 357.1381 [M+H]$^+$ calc. 357.1392.

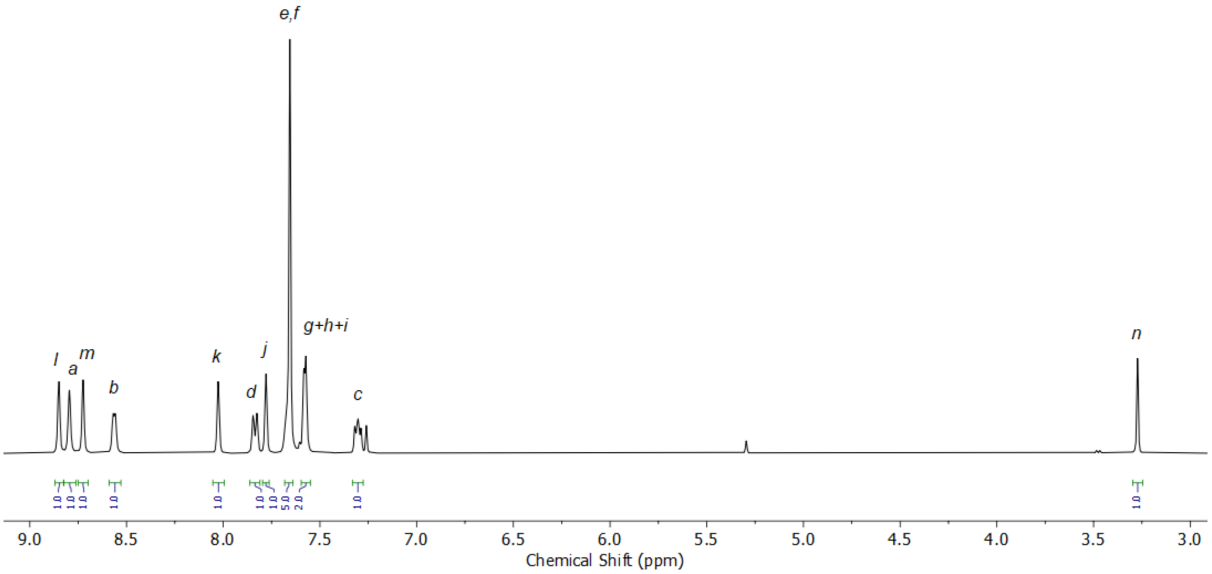

Figure S133 ^{1}H NMR (400 MHz, $CDCl_3$) of **L1^{CCH}**.

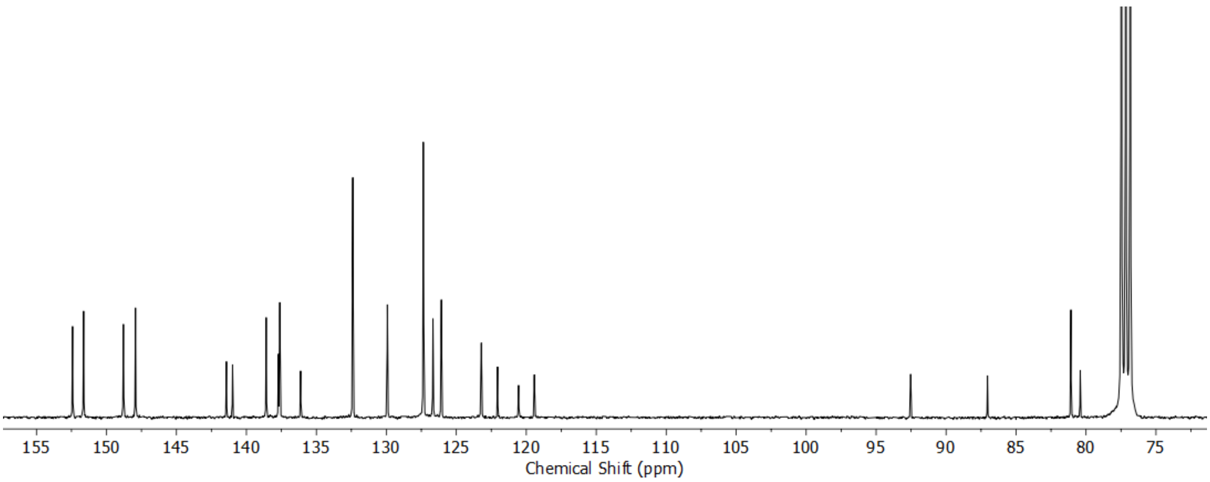

Figure S134 ^{13}C NMR (101 MHz, $CDCl_3$) of **L1**$^{\mathbf{CCH}}$.

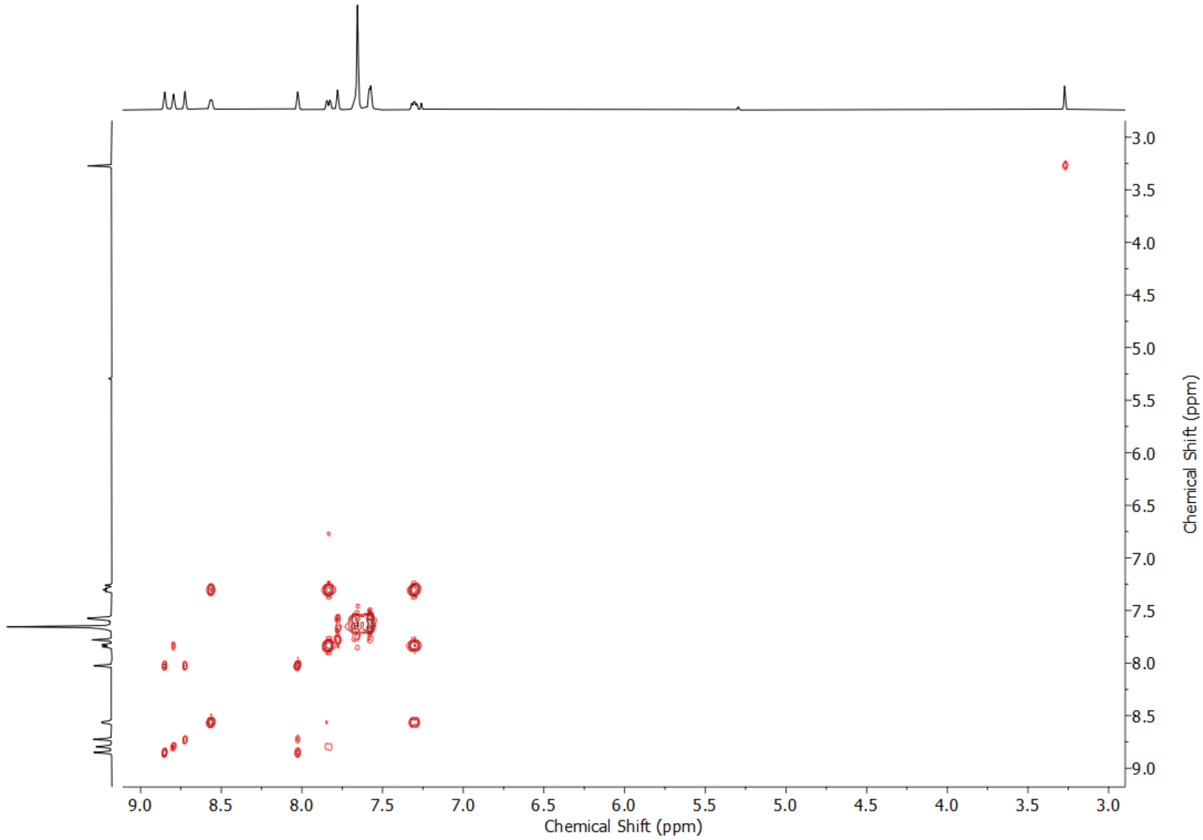

Figure S135 COSY NMR ($CDCl_3$) of **L1**$^{\mathbf{CCH}}$.

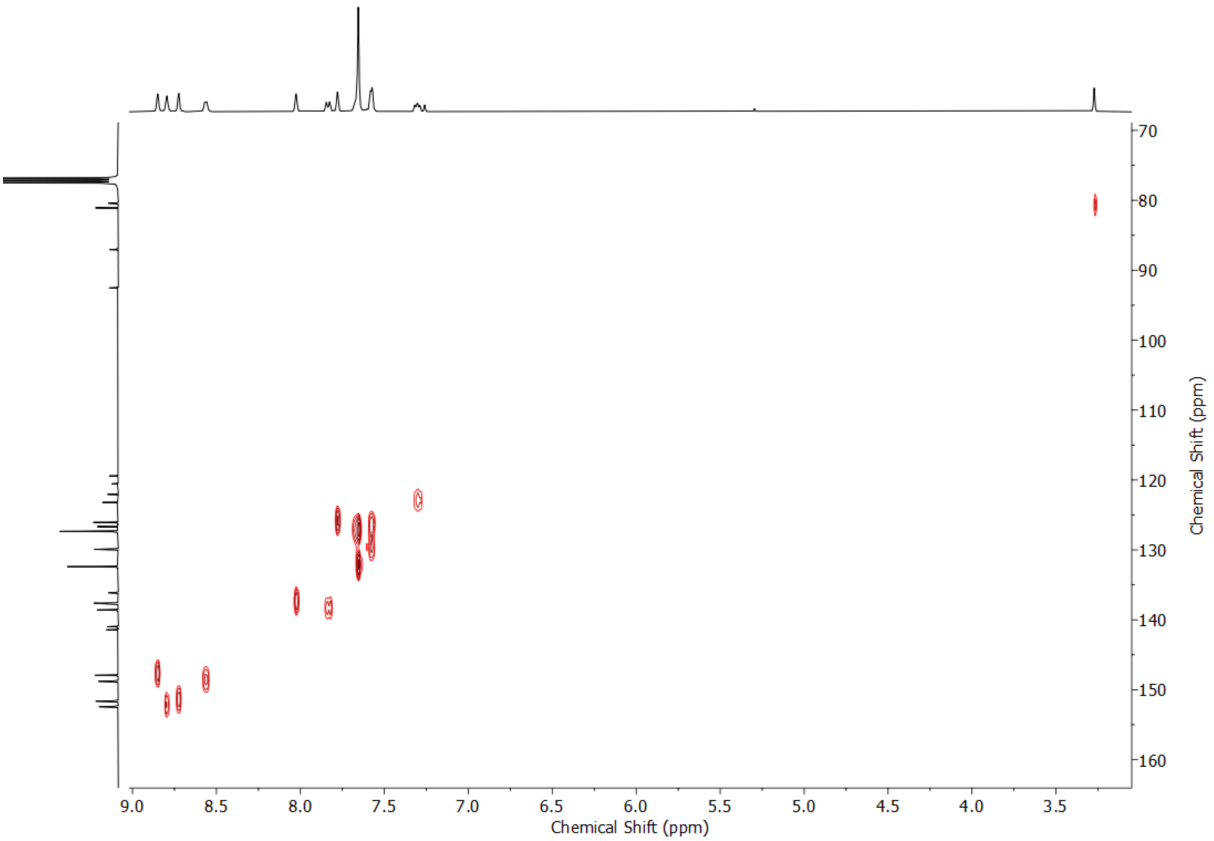

Figure S136 HSQC NMR ($CDCl_3$) of **L1**$^{\mathbf{CCH}}$.

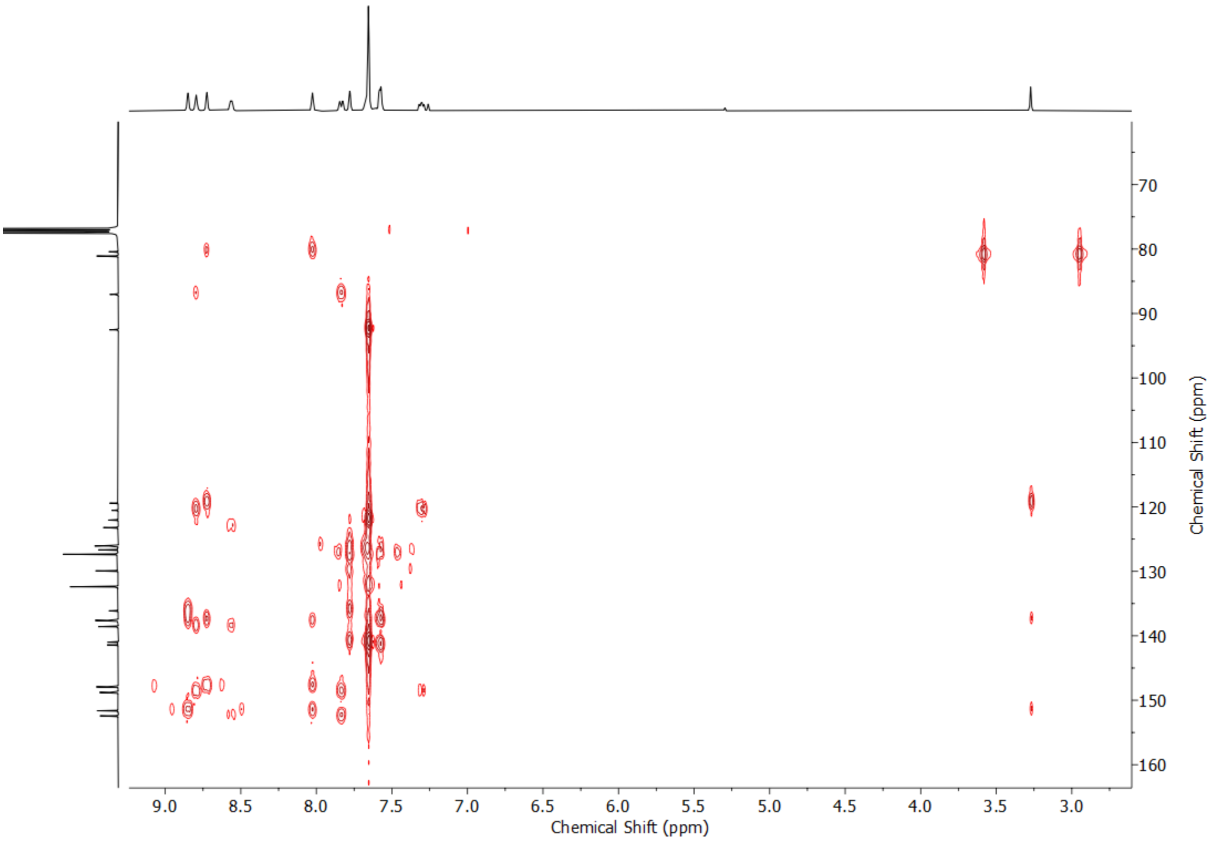

Figure S137 HMBC NMR ($CDCl_3$) of **L1**$^{\mathbf{CCH}}$.

Synthesis of L2^{Ph}

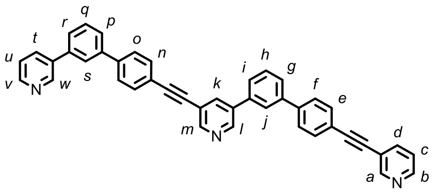

L1^{CCH} (0.0535 g, 0.15 mmol, 1 eq.), **S9** (0.0534 g, 0.15 mmol, 1 eq.), $Pd(PPh_3)_4$ (0.0087 g, 0.0075 mmol, 5 mol%) and CuI (0.0014 g, 0.0075 mmol, 5 mol%) were stirred at rt in 1:1 dioxane/$^iPr_2NH$ (2 mL) for 19 h, with additional dioxane (1 mL) added after 2 h. EDTA solution (20 mL) was added and the aqueous phase extracted with CH_2Cl_2 (3 × 10 mL). The combined organic phases were dried ($MgSO_4$) and the solvent removed *in vacuo*. The crude residue was dissolved in 2:1 CH_2Cl_2/^iPrOH (6 mL). The CH_2Cl_2 was removed *in vacuo* to give a precipitate. Following addition of pentane (4 mL) the suspension was filtered over celite, washing copiously with pentane until the filtrate ran clear. After discarding the filtrate, the solid was dissolved in CH_2Cl_2 and the solvent removed *in vacuo* to give the product as a yellow solid (0.0804 g, 91%).

^{1}H NMR (400 MHz, $CDCl_3$) δ: 8.92 (s, 1H, H_w), 8.84 (d, J = 1.9 Hz, 1H, H_l), 8.80 (br. app. s, 2H, H_a, H_m), 8.63 (d, J = 4.8 Hz, 1H, H_v), 8.56 (dd, J = 5.0, 1.7 Hz, 1H, H_b), 8.09 (app. t, J = 2.1 Hz, 1H, H_k), 7.94 (app. dt, J = 7.9, 1.9 Hz, 1H, H_t), 7.85-7.80 (m, 3H, H_d, H_j, H_s), 7.69-7.57 (m, 14H, H_e, H_f, H_g, H_h, H_i, H_n, H_o, H_p, H_q, H_r), 7.41 (dd, J = 7.9, 4.8 Hz, 1H, H_u), 7.30 (ddd, J = 7.9, 4.9, 0.9 Hz, 1H, H_c).

^{13}C NMR (101 MHz, $CDCl_3$) δ: 152.4 (C_a/C_m), 151.1 (C_a/C_m), 148.8 (× 2, C_b, C_v), 148.4 (C_w), 147.4 (C_l), 141.4, 141.3, 141.0, 138.7, 138.6 (C_d/C_j/C_s), 137.9, 137.0 (C_k), 136.7, 136.2, 134.7 (C_t), 132.4 (1 of C_e/C_f/C_o/C_n), 132.4 (1 of C_e/C_f/C_o/C_n), 129.9, 129.8, 127.4 (1 of C_e/C_f/C_o/C_n), 127.4 (1 of C_e/C_f/C_o/C_n), 127.3, 127.0, 126.7, 126.7, 126.1 (2 of C_d/C_j/C_s), 123.8 (C_u), 123.2 (C_c), 122.0, 121.9, 120.6, 92.9, 92.6, 87.0, 86.9 (3 signals missing due to peak overlap).

HR-ESI-MS m/z = 586.2265 $[M+H]^+$ calc. 586.2283.

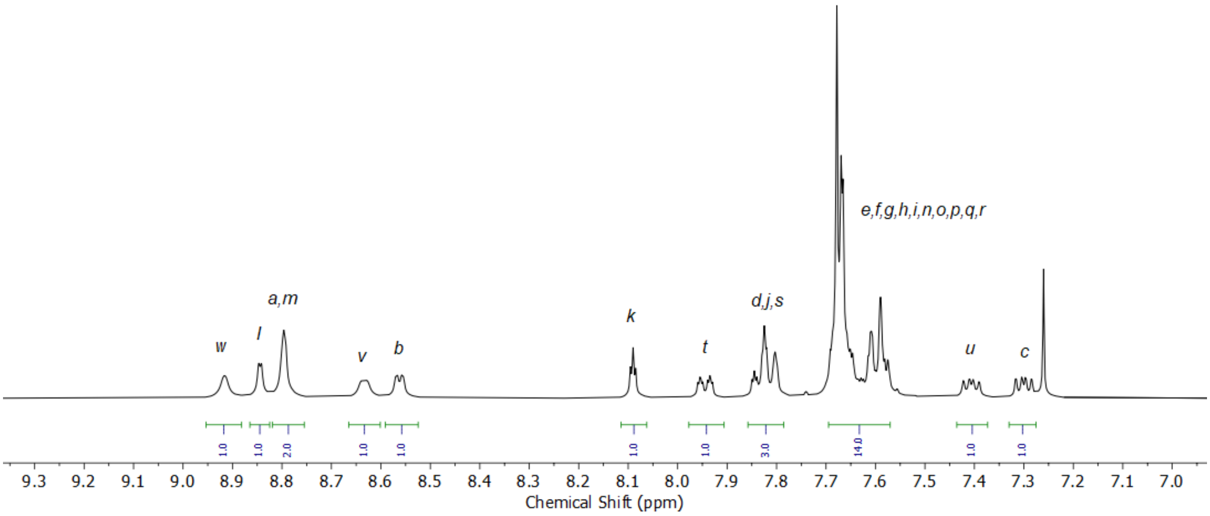

Figure S138 ^{1}H NMR (400 MHz, $CDCl_3$) of **L2^{Ph}**.

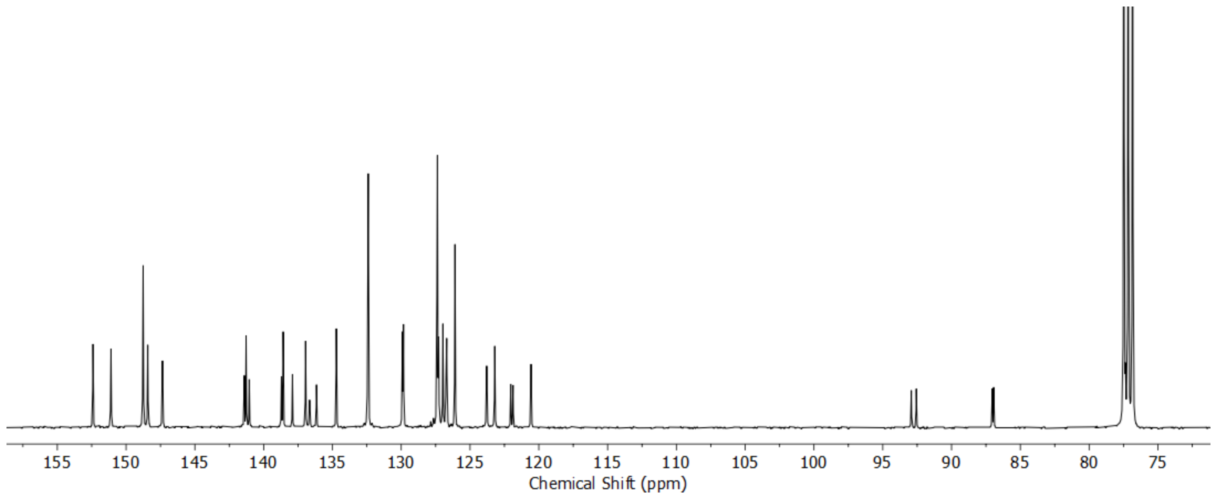

Figure S139 ^{13}C NMR (101 MHz, $CDCl_3$) of **L2^{Ph}**.

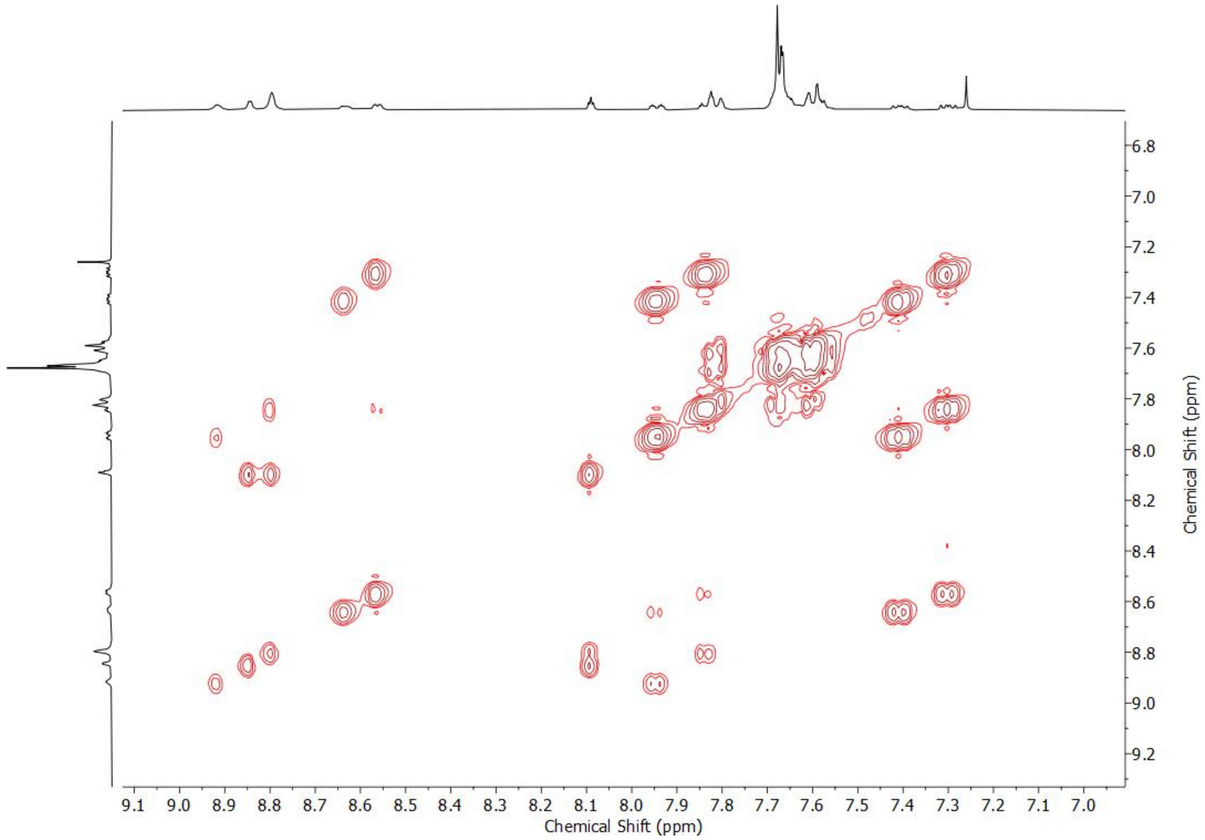

Figure S140 COSY NMR ($CDCl_3$) of **L2^{Ph}**.

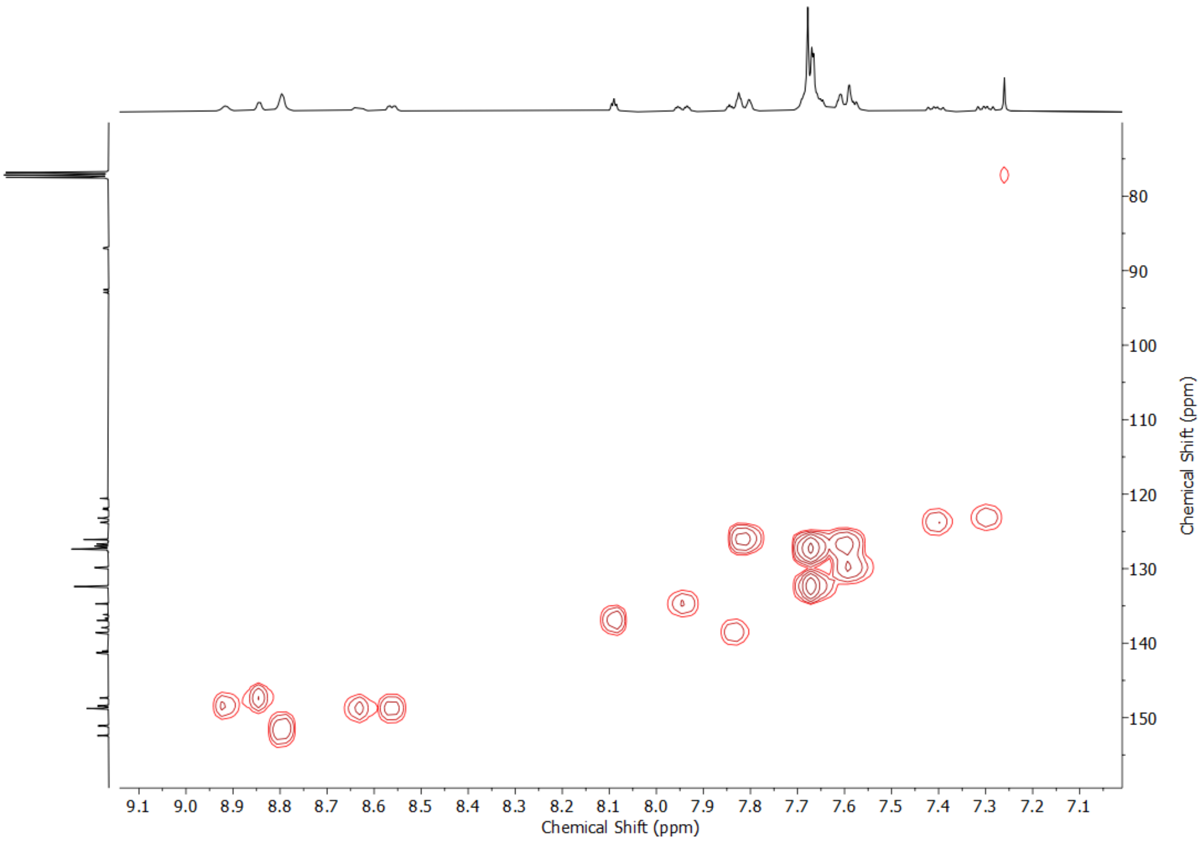

Figure S141 HSQC NMR ($CDCl_3$) of **L2^{Ph}**.

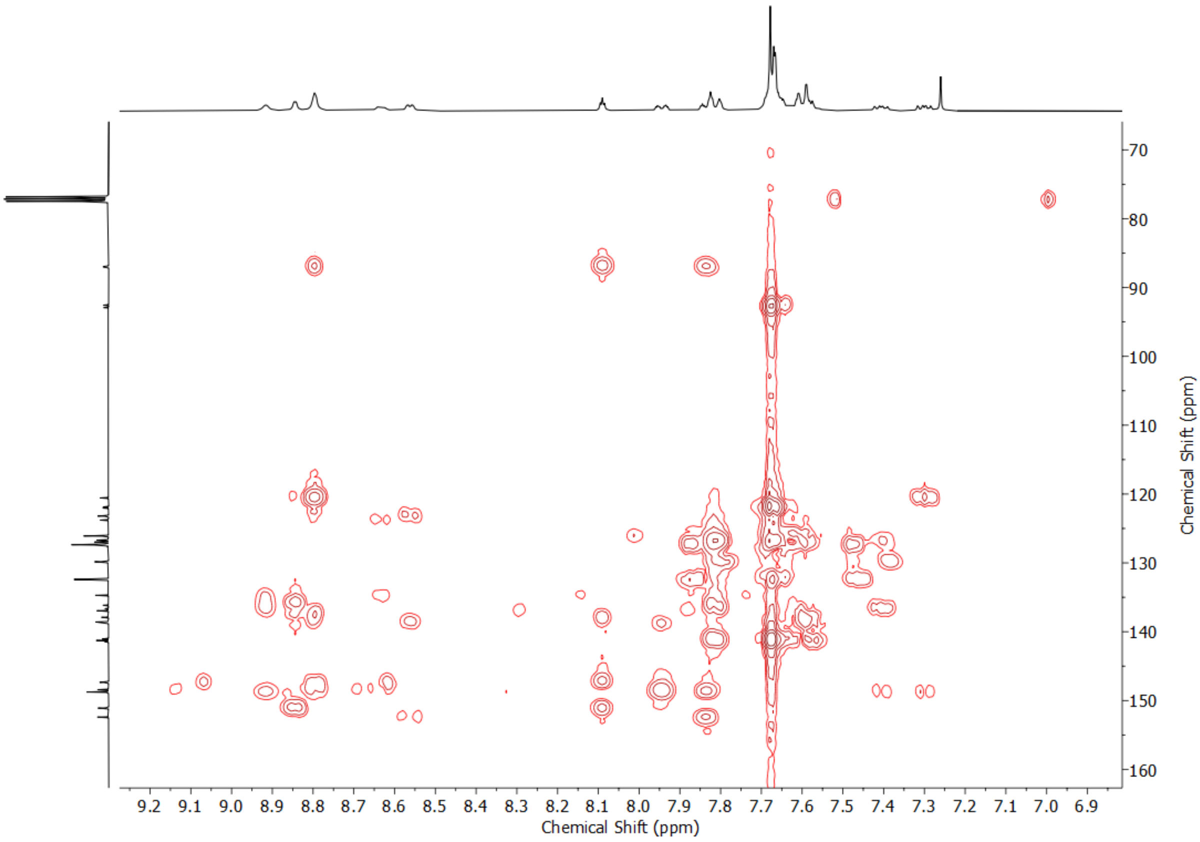

Figure S142 HMBC NMR ($CDCl_3$) of **L2^{Ph}**.

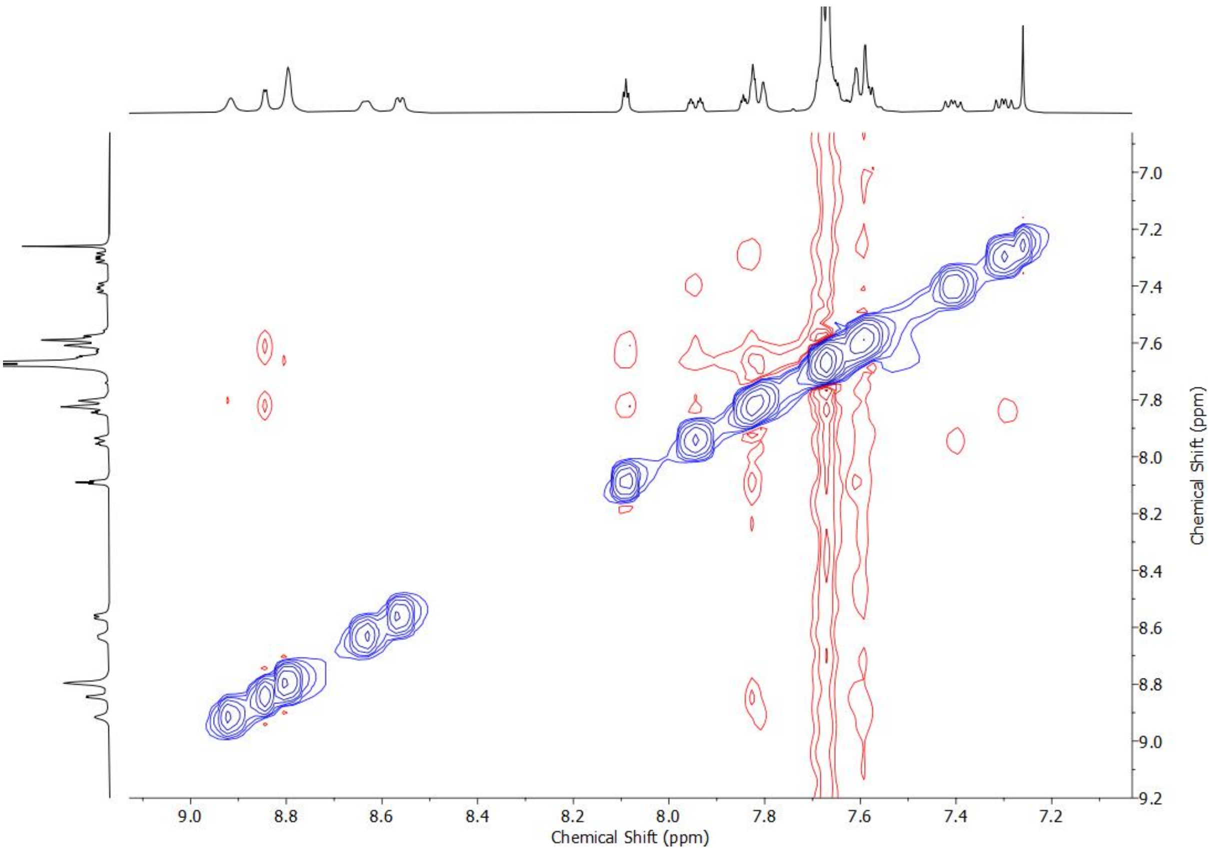

Figure S143 NOESY ($CDCl_3$) of **L2^{Ph}**.

Synthesis of *cis*-$[Pd_3(L2^{Ph})_4](BF_4)_6$ ($C2^{Ph}$)

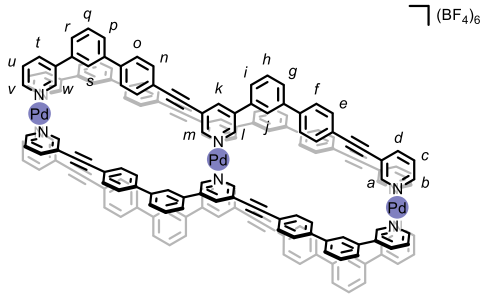

To a solution of $[Pd(CH_3CN)_4](BF_4)_2$ (6.7 mg, 15 µL) in d_6-DMSO (0.75 mL) was added $\mathbf{L2^{Ph}}$ (11.7 mg, 20 µmol, 4 eq.) in $CDCl_3$ (0.25 mL). After standing at 50 °C for 3 d, quantitative conversion to *cis*-$[Pd_3(\mathbf{L2^{Ph}})_4](BF_4)_6$ ($\mathbf{C2^{Ph}}$) was observed by ^1H NMR.

^{1}**H NMR** (400 MHz, 3:1 d_6-DMSO/$CDCl_3$) δ: 9.94 (s, 4H, H_m), 9.89 (s, 4H, H_a), 9.60 (s, 4H, H_l), 9.51 (s, 4H, H_w), 9.44 (d, J = 5.9 Hz, 4H, H_v), 9.33 (d, J = 5.5 Hz, 4H, H_b), 8.78 (s, 4H, H_k), 8.55 (d, J = 8.3 Hz, 8H, H_t), 8.42 (s, 4H, H_j), 8.34 (s, 4H, H_s), 8.20 (d, J = 8.1 Hz, 4H, H_d), 8.09 (d, J = 7.7 Hz, 8H, H_o), 8.04 (d, J = 7.8 Hz, 8H, H_f), 7.94-7.90 (m, 8H, H_i, H_u), 7.86-7.75 (m, 64H, H_c, H_e, H_g, H_n, H_p, h_r), 7.63-7.59 (m, 8H, H_h, H_q).

Diffusion coefficient (400 MHz, 3:1 d_6-DMSO/$CDCl_3$) D: 8.73 × 10^{-11} m^2s^{-1}.

^{13}**C NMR** (101 MHz, 3:1 d_6-DMSO/$CDCl_3$) δ: 153.6 (C_a), 152.0 (Cm), 150.6 (C_b), 150.2 (C_v), 148.6 (C_w), 148.1 (C_l), 142.5 (C_d), 141.2, 140.6, 140.1 (C_k), 139.1 (C_t), 138.6, 138.0, 134.9, 133.9, 132.7, 130.8, 129.1, 128.4, 128.2, 128.0, 127.5, 127.3, 124.9 (×2, C_j, C_s), 123.6, 123.5, 121.0, 120.8, 118.0, 95.8, 95.3, 85.3, 85.0 (5 signals missing due to peak overlap).

ESI-MS m/z = 973.74 {$[Pd_3(\mathbf{L2^{Ph}})_4(BF_4)_3]^{3+}$ calc. 973.87; 1504.10 {$[Pd_3(\mathbf{L2^{Ph}})_4(BF_4)_4]^{2+}$ calc. 1504.31.

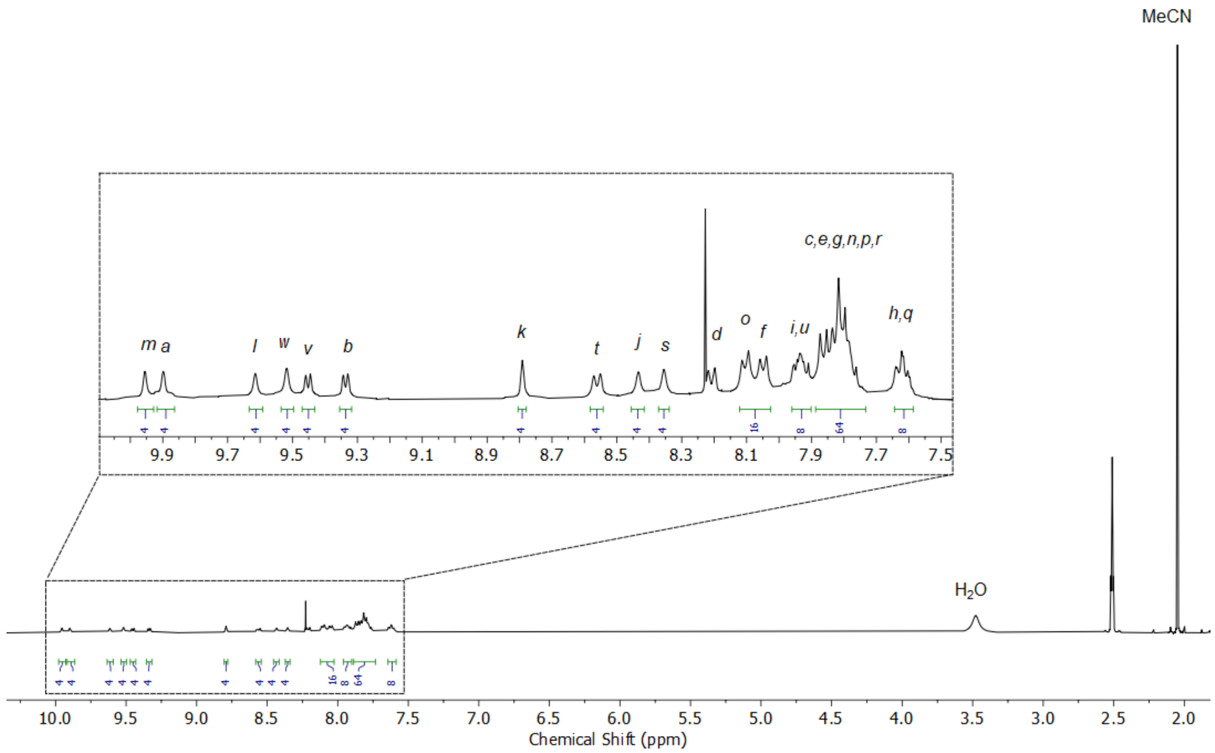

Figure S144 ^{1}H NMR (400 MHz, 3:1 d_6-DMSO/$CDCl_3$) of $[Pd_3(\mathbf{L2^{Ph}})_4](BF_4)_6$.

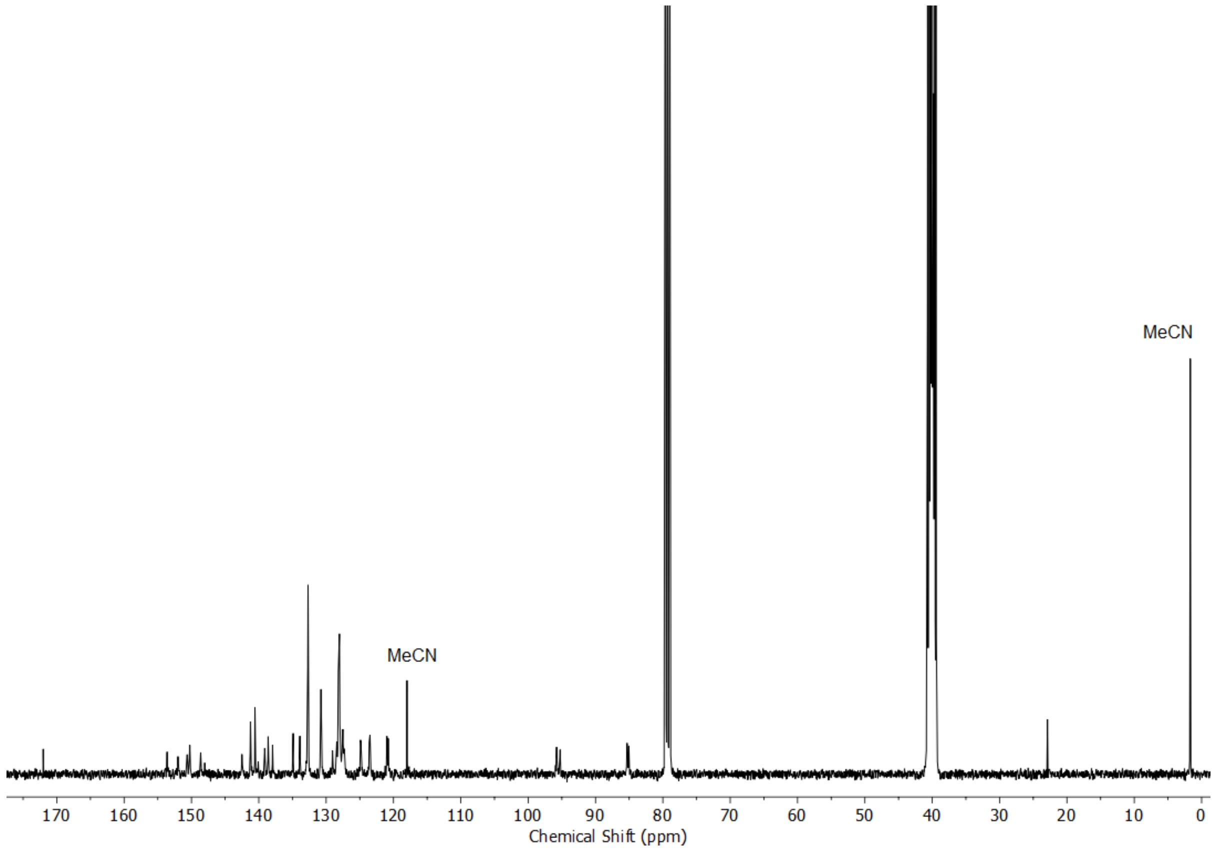

Figure S145 ^{13}C NMR (101 MHz, 3:1 d_6-DMSO/$CDCl_3$) of $[Pd_3(\mathbf{L2^{Ph}})_4](BF_4)_6$.

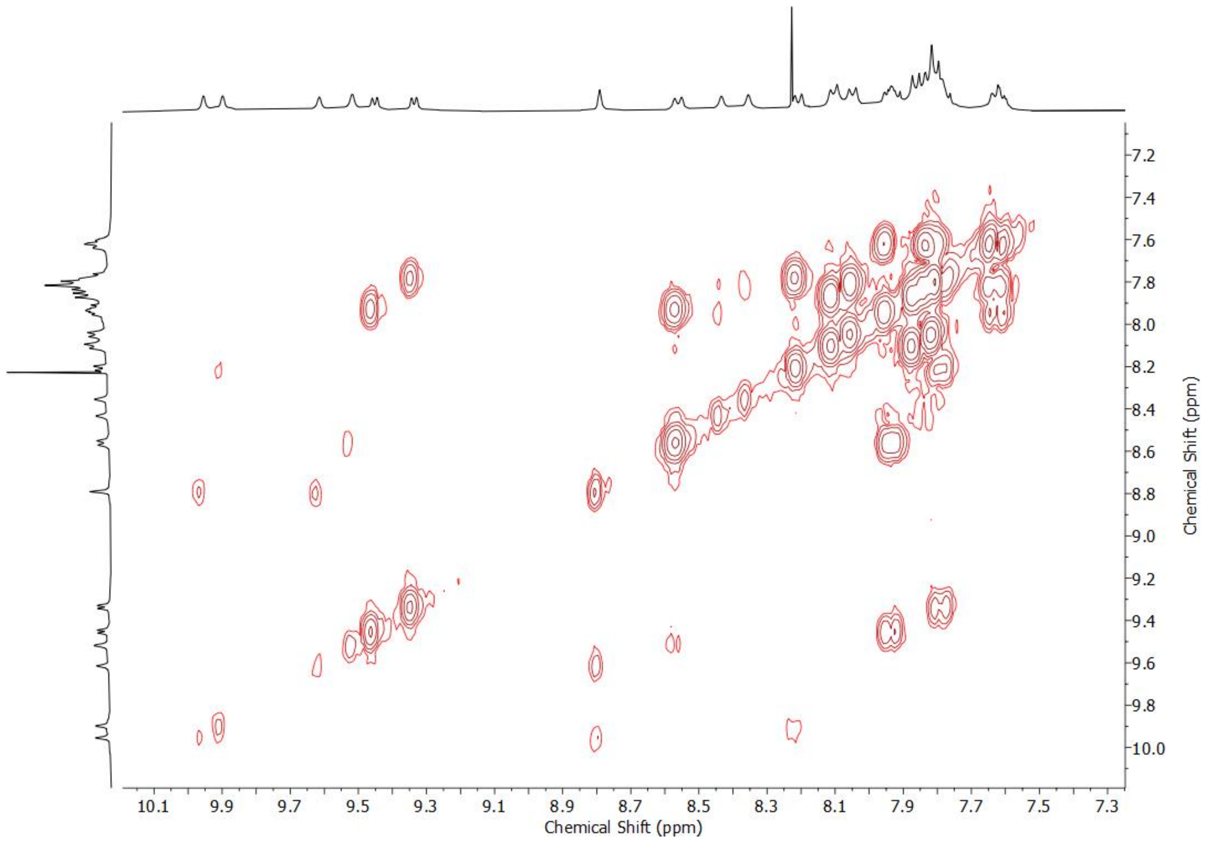

Figure S146 COSY NMR (3:1 d_6-DMSO/$CDCl_3$) of $[Pd_3(\mathbf{L2^{Ph}})_4](BF_4)_6$.

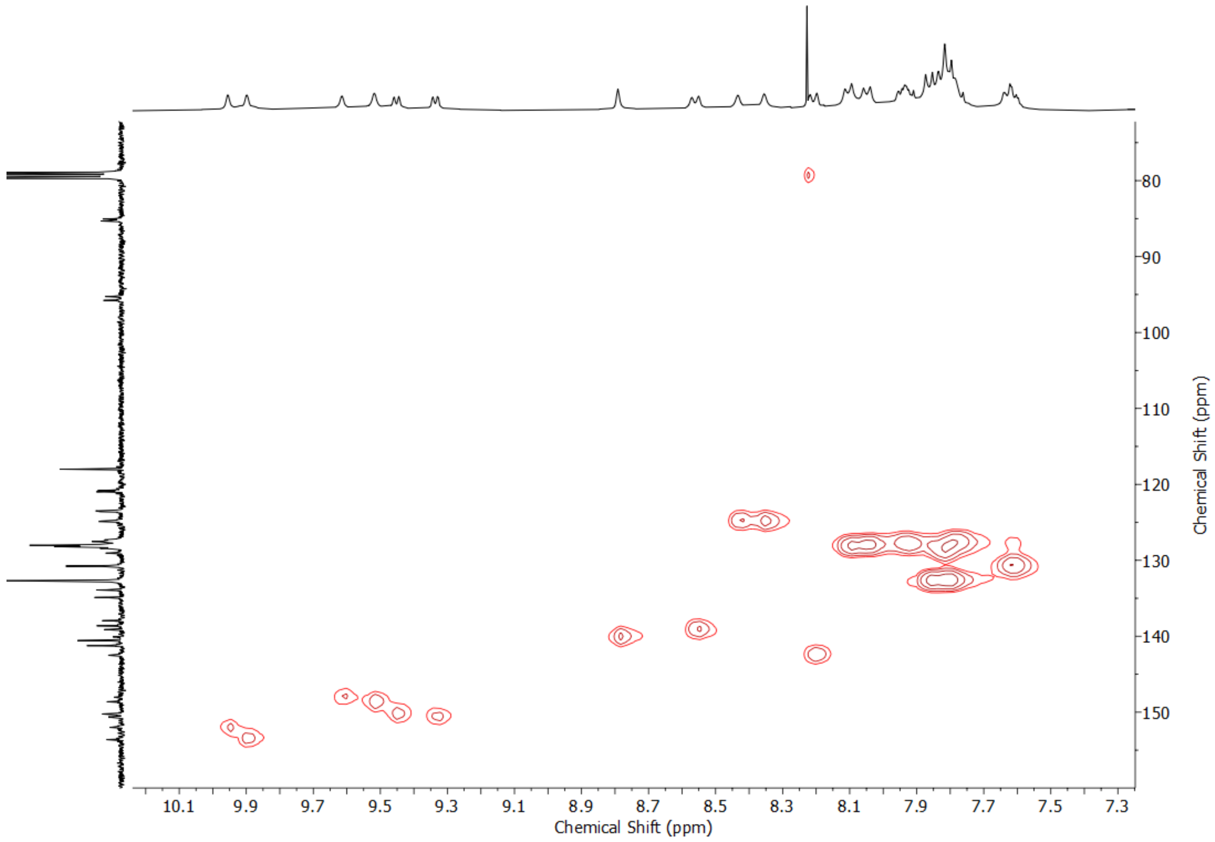

Figure S147 HSQC NMR (3:1 d_6-DMSO/$CDCl_3$) of $[Pd_3(\mathbf{L2^{Ph}})_4](BF_4)_6$.

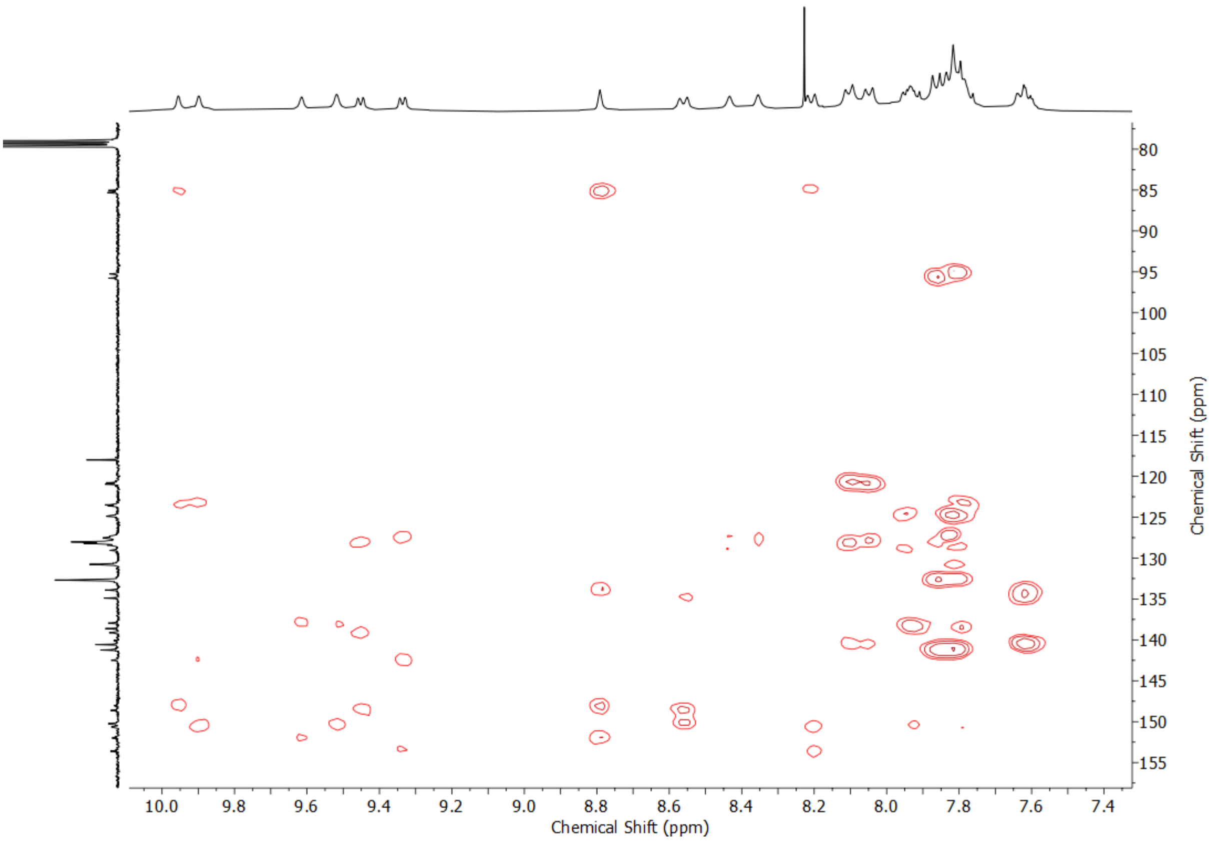

Figure S148 HMBC NMR (3:1 d_6-DMSO/$CDCl_3$) of $[Pd_3(\mathbf{L2^{Ph}})_4](BF_4)_6$.

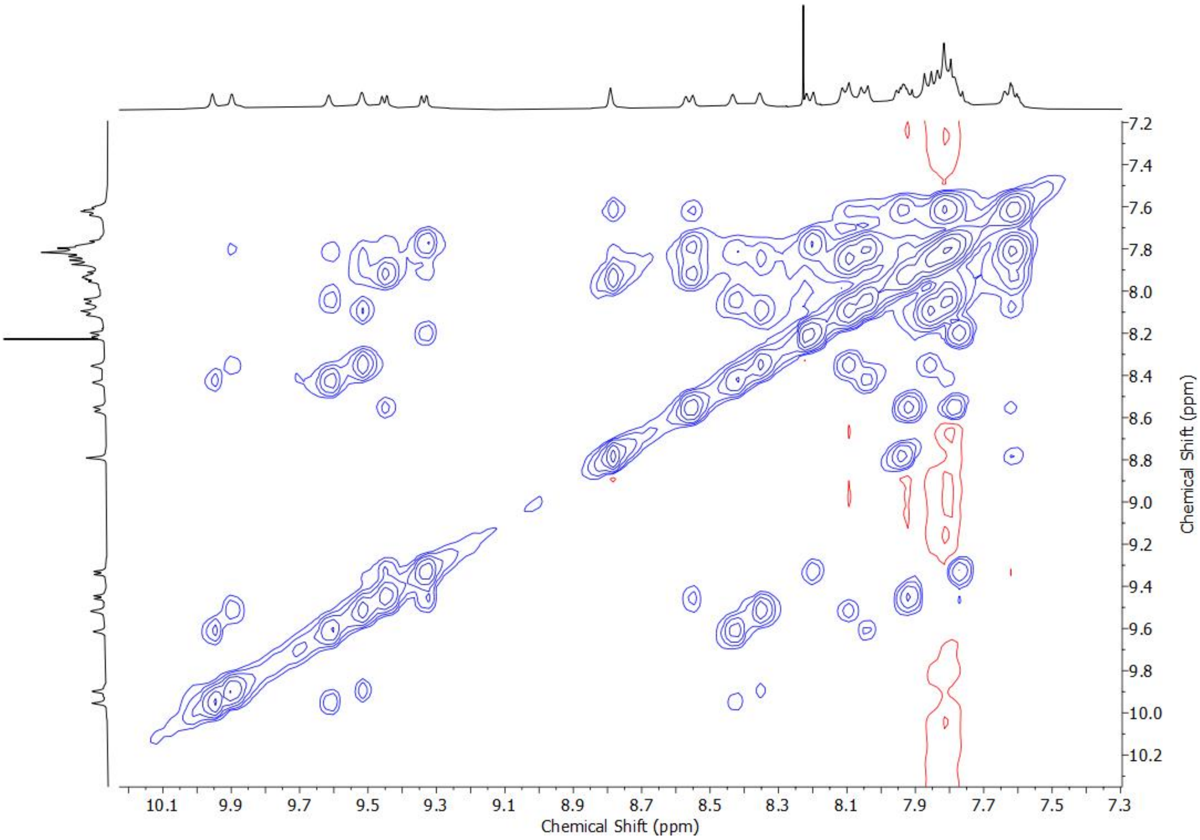

Figure S149 NOESY (3:1 d_6-DMSO/$CDCl_3$) of $[Pd_3(\mathbf{L2^{Ph}})_4](BF_4)_6$.

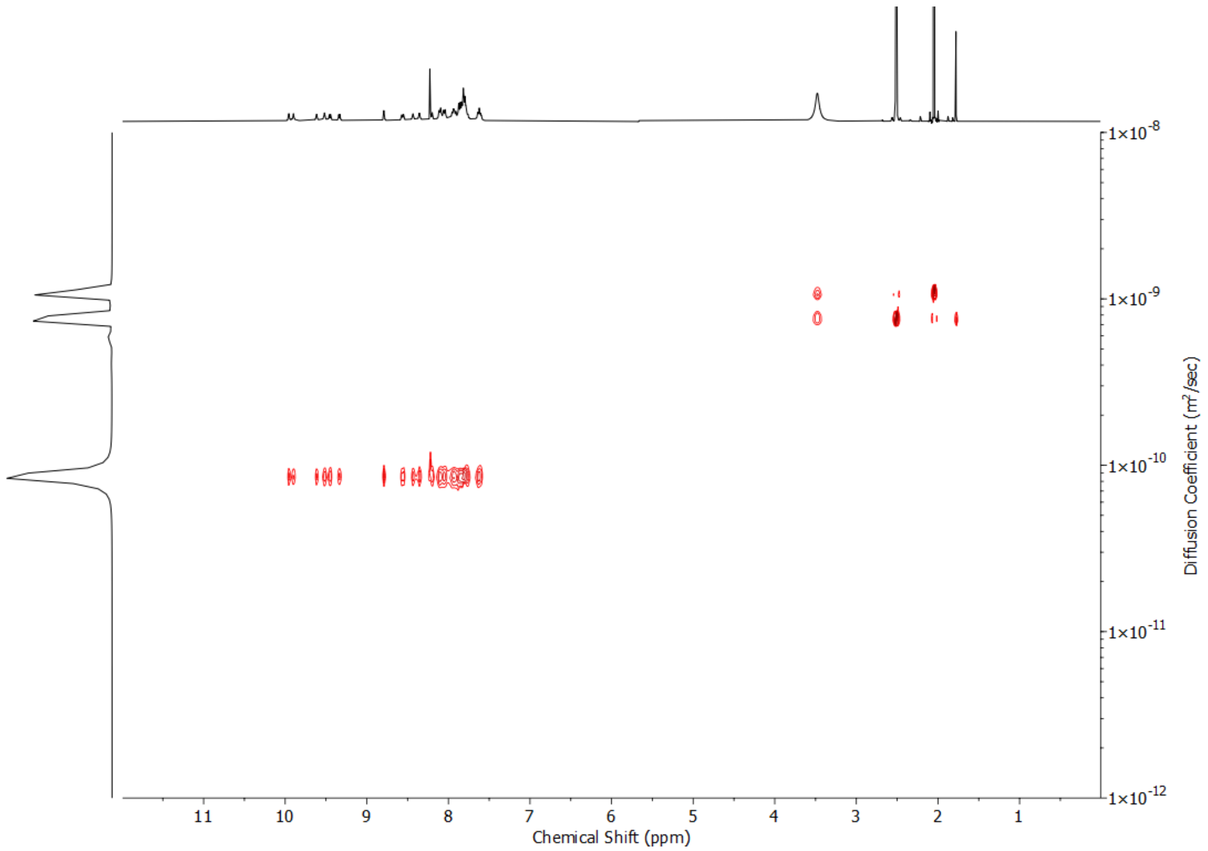

Figure S150 DOSY (3:1 d_6-DMSO/$CDCl_3$) of $[Pd_3(\mathbf{L2^{Ph}})_4](BF_4)_6$.

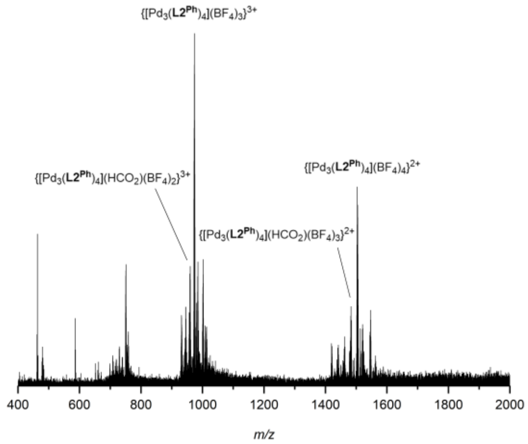

Figure S151 ESI-MS of $[Pd_3(\mathbf{L2^{Ph}})_4](BF_4)_6$.

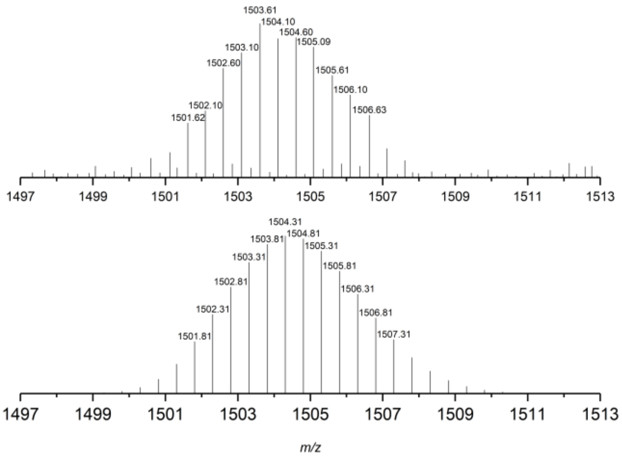

Figure S152 Observed (top) and calculated (bottom) isotopic patterns for $\{[Pd_3(\mathbf{L2^{Ph}})_4(BF_4)_4]^{2+}$.

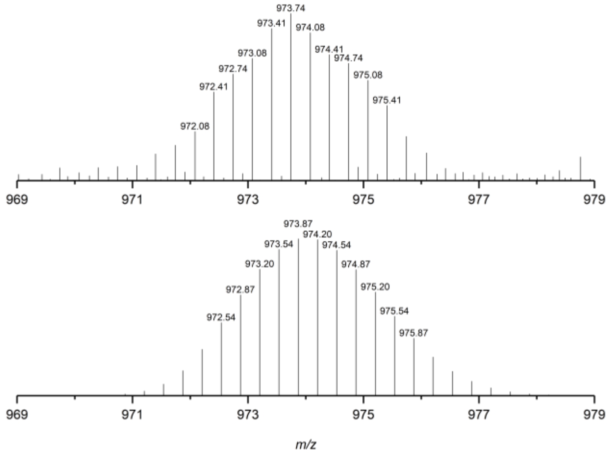

Figure S153 Observed (top) and calculated (bottom) isotopic patterns for $\{[Pd_3(\mathbf{L2^{Ph}})_4(BF_4)_3]^{3+}$.

Synthesis of L2^{Xy}

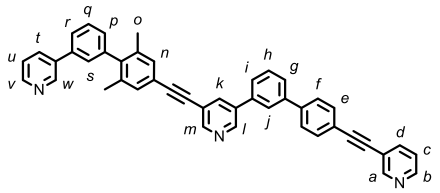

L1^{CCH} (0.0535 g, 0.15 mmol, 1 eq.), **S9** (0.0578 g, 0.15 mmol, 1 eq.), $Pd(PPh_3)_4$ (0.0087 g, 0.0075 mmol, 5 mol%) and CuI (0.0014 g, 0.0075 mmol, 5 mol%) were stirred at rt in 1:1 $^{i}Pr_2NH$/dioxane (2 mL) for 24 h. EDTA solution (20 mL) was added and the aqueous phase extracted with CH_2Cl_2 (3 × 10 ML). The combined organic phases were dried ($MgSO_4$) and the solvent removed *in vacuo*. Following purification by column chromatography on silica gel (CH_2Cl_2 with step gradient 0 to 30% acetone in 10% increments) a light orange oil was obtained. This was dissolved in a minimal amount of CH_2Cl_2 before the addition of $^{i}PrOH$ (2 mL). The CH_2Cl_2 was removed under a flow of air before an excess of pentane was added. The mother liquor was carefully decanted from the resultant precipitate which was dried *in vacuo* to give the product as an off-white foam (0.0647 g, 70%).

^{1}H NMR (400 MHz, $CDCl_3$) δ: 8.89 (br. s, 1H, H_w), 8.84 (d, J = 2.2 Hz, 1H, H_l), 8.80 (d, J = 1.5 Hz, 1H, H_a), 8.78 (d, J = 1.9 Hz, 1H, H_m), 8.61 (br. d, J = 4.8 Hz, 1H, H_v), 8.57 (dd, J = 4.9, 1.7 Hz, 1H, H_b), 8.08 (t, J = 2.1 Hz, 1H, H_k), 7.94 (ddd, J = 7.9, 2.4, 1.6 Hz, 1H, H_t), 7.86-7.82 (m, 2H, H_d, H_j), 7.70-7.55 (m, 9H, H_e, H_f, H_g, H_h, H_i, H_q, H_r), 7.42-7.37 (m, 4H, H_n, H_s, H_u), 7.31 (ddd, J = 7.9, 4.9, 0.9 Hz, 1H, H_c), 7.21 (app. dt, J = 6.9, 1.7 Hz, 1H, H_p), 2.10 (s, 6H, H_o).

^{13}C NMR (101 MHz, $CDCl_3$) δ: 152.4 (C_a), 151.1 (C_m), 148.8 (C_b), 148.4 (C_v), 148.1 (C_w), 147.2 (C_l), 142.4, 141.4 (×2), 141.1, 138.6 (C_d), 138.1, 138.0, 137.0, 136.7, 136.6 (C_k), 136.1, 134.8 (C_t), 132.4 (C_e/C_f), 130.8 (C_n/C_s/C_u), 129.9 (C_g/C_h/C_i/C_q/C_r), 129.6 (C_g/C_h/C_i/C_q/C_r), 128.9 (C_p), 127.6 (C_n/C_s/C_u), 127.4 (C_e/C_f), 127.3 (C_g/C_h/C_i/C_q/C_r), 126.7 (C_g/C_h/C_i/C_q/C_r), 126.1 (C_g/C_h/C_i/C_q/C_r), 125.9 (C_j), 123.9 (C_n/C_s/C_u), 123.2 (C_c), 122.0, 121.3, 120.8, 120.6, 93.3, 92.6, 87.0, 85.6, 21.0 (C_o).

HR-ESI-MS m/z = 614.2582 $[M+H]^+$ calc. 614.2596.

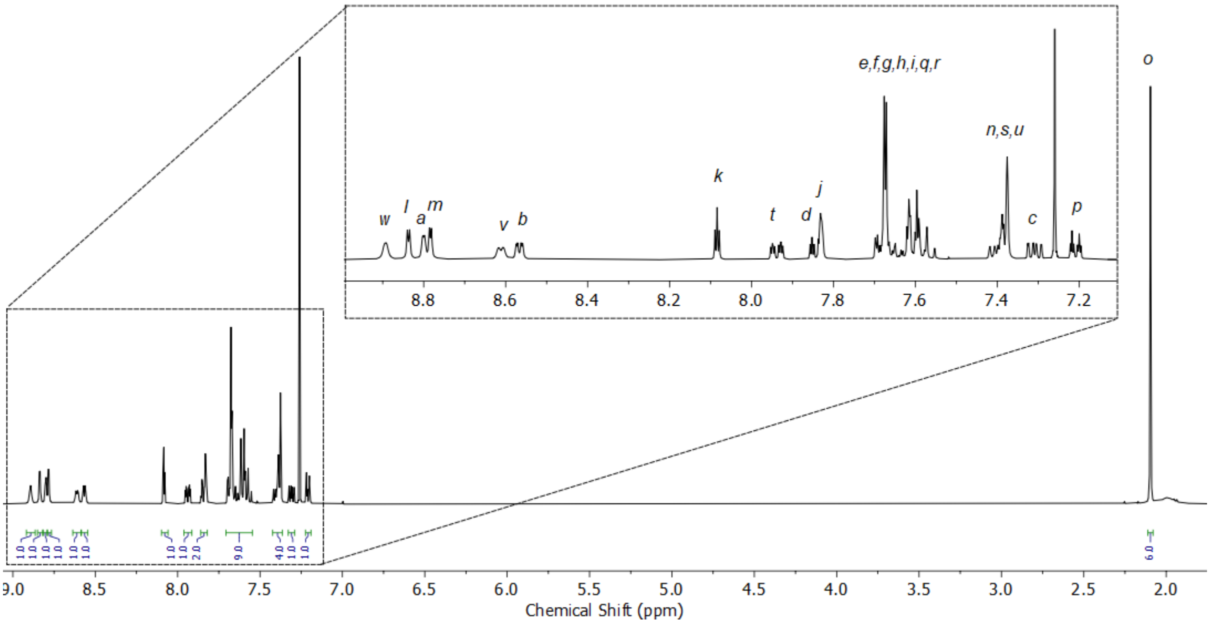

Figure S154 ^{1}H NMR (400 MHz, $CDCl_3$) of **L2^{Xy}**.

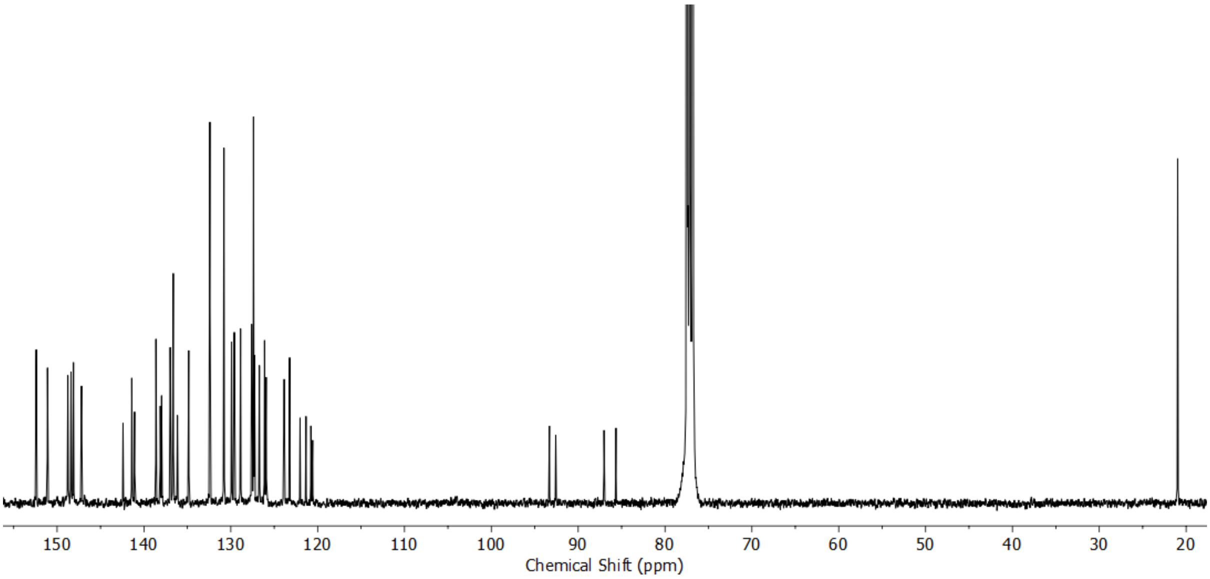

Figure S155 ^{13}C NMR (101 MHz, $CDCl_3$) of **L2^{Xy}**.

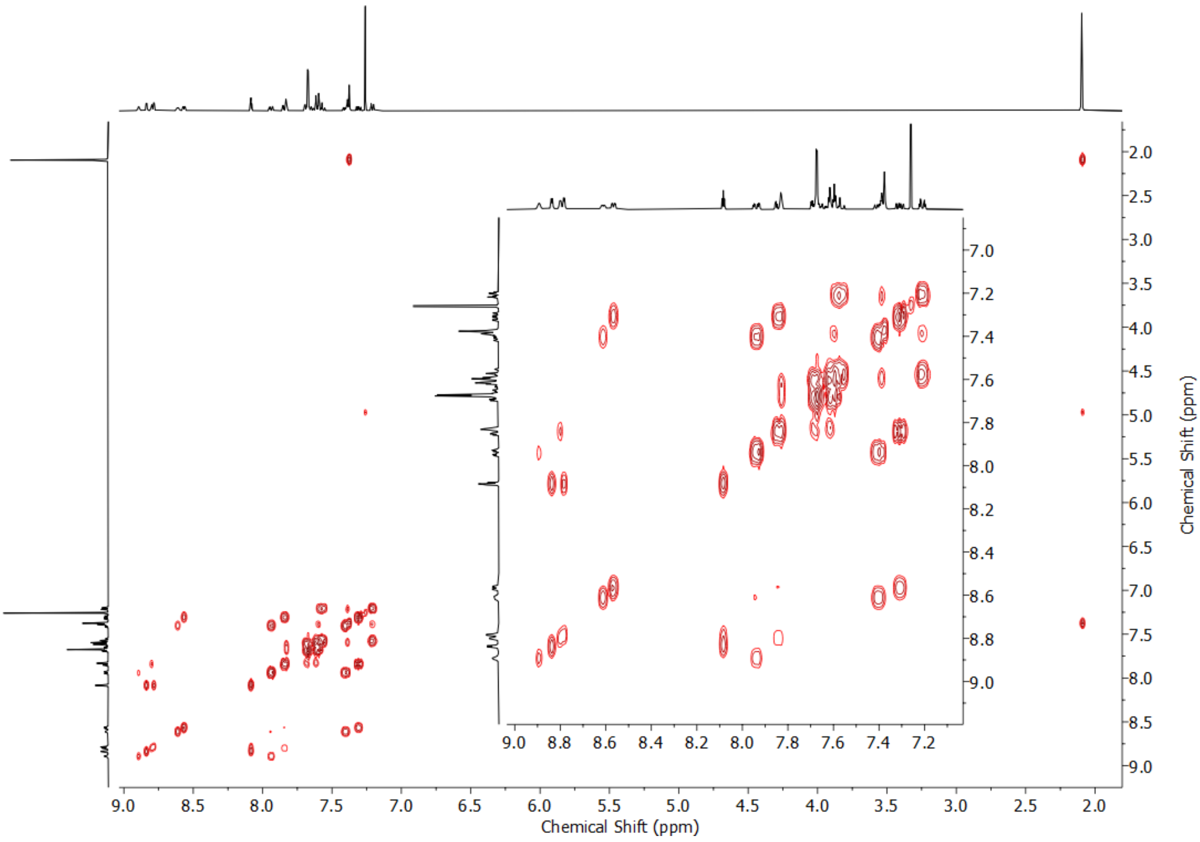

Figure S156 COSY NMR ($CDCl_3$) of **L2^{Xy}**.

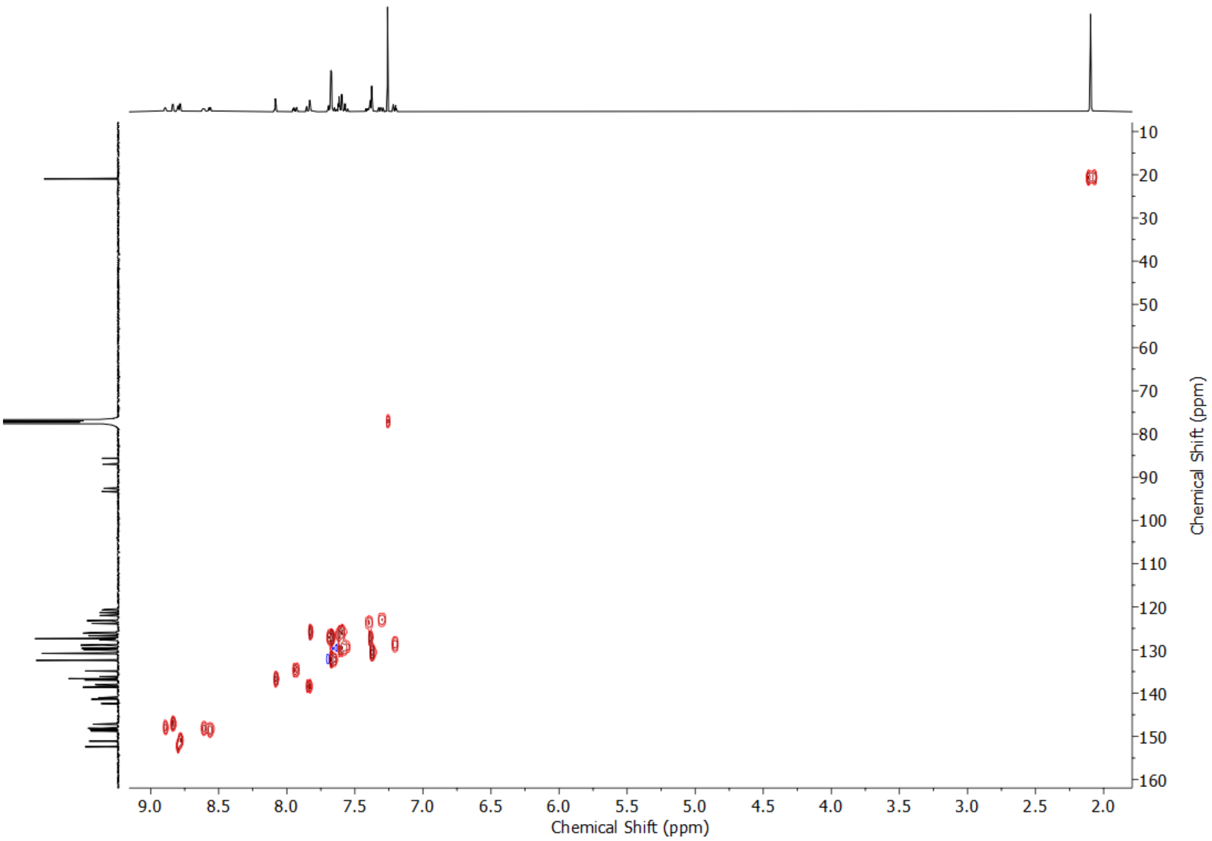

Figure S157 HSQC NMR ($CDCl_3$) of **L2^{Xy}**.

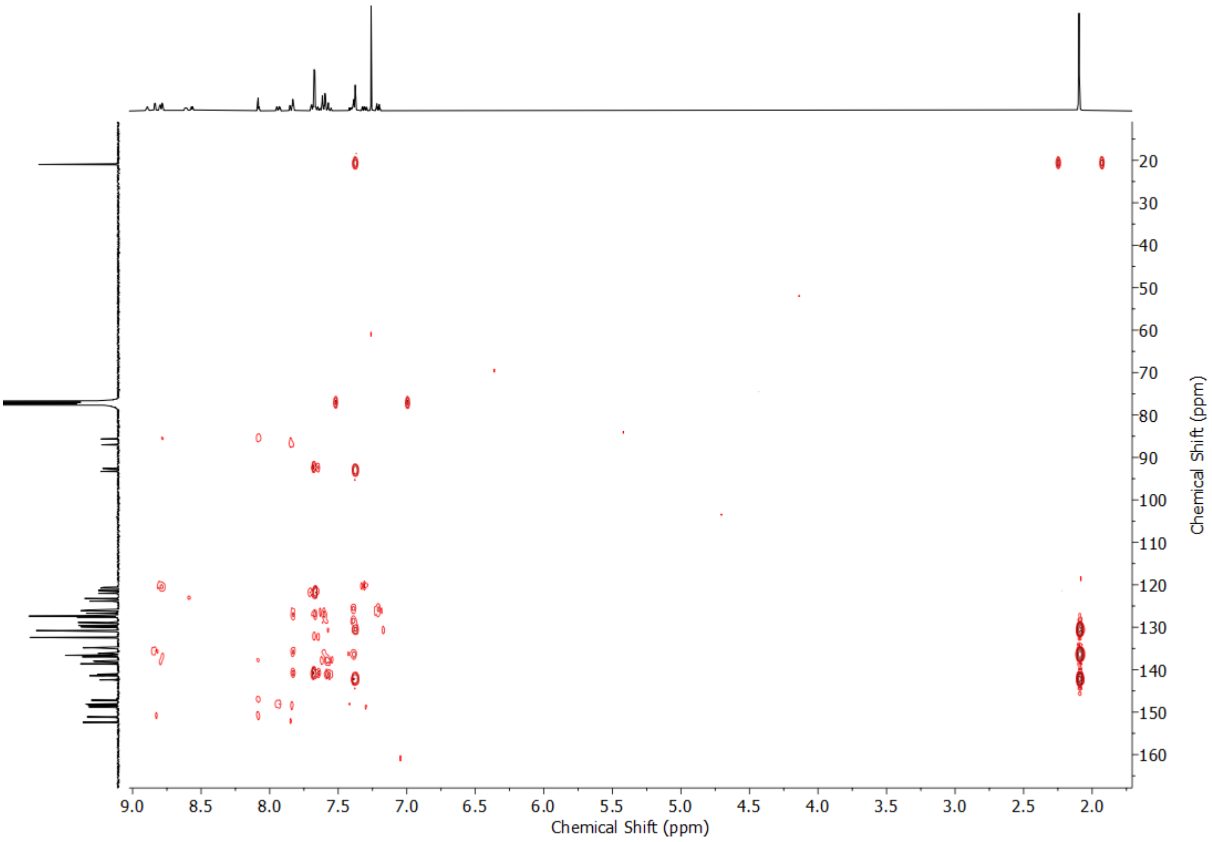

Figure S158 HMBC NMR ($CDCl_3$) of **L2^{Xy}**.

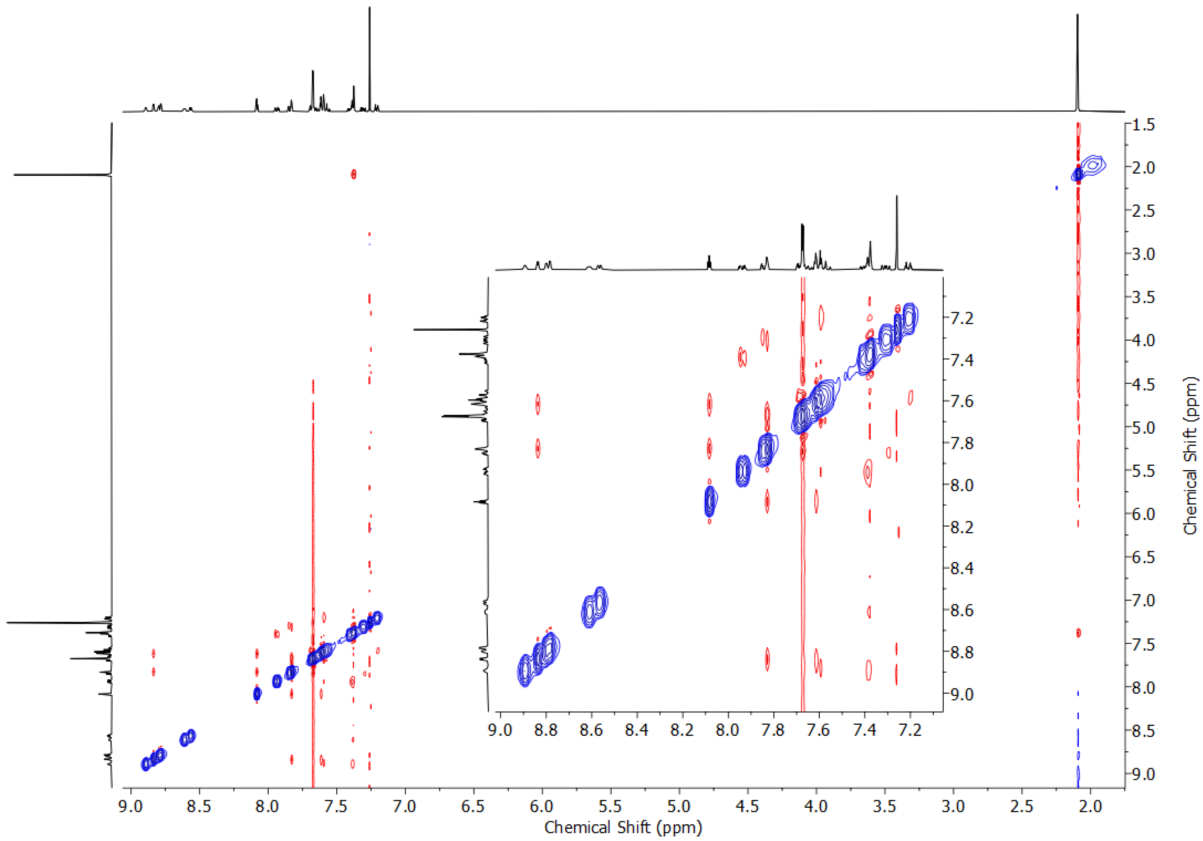

Figure S159 NOESY ($CDCl_3$) of **L2**$^{\mathbf{Xy}}$.

Synthesis of *cis*-[Pd$_3$(L2^{Xy})$_4$](BF$_4$)$_6$ (C2^{Xy})

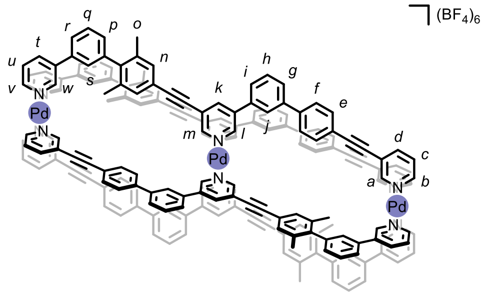

To a solution of [Pd(CH$_3$CN)$_4$](BF$_4$)$_2$ (6.7 mg, 15 µL) in d_6-DMSO (0.75 mL) was added **L2^{Xy}** (12.3 mg, 20 µmol, 4 eq.) in CDCl$_3$ (0.25 mL). After standing at 50 °C for 3 d, quantitative conversion to *cis*-[Pd$_3$(**L2^{Xy}**)$_4$](BF$_4$)$_6$ (**C2^{Xy}**) was observed by ^{1}H NMR.

^{1}H NMR (400 MHz, 3:1 d_6-DMSO/CDCl$_3$) δ: 10.00 (s, 4H, H$_m$), 9.71 (s, 4H, H$_a$), 9.61 (s, 4H, H$_l$), 9.30 (d, J = 5.3 Hz, 4H, H$_v$), 9.26 (d, J = 5.9 Hz, 4H, H$_b$), 9.16 (s, 4H, H$_w$), 8.78 (s, 4H, H$_k$), 8.55-8.53 (m, 8H, H$_j$, H$_t$), 8.24 (m, 8H, H$_f$), 8.18 (d, J = 8.1 Hz, 4H, H$_d$), 7.96 (d, J = 7.9 Hz, 4H, H$_i$), 7.92-7.89 (m, 8H, H$_g$, H$_u$), 7.80 (d, J = 7.9 Hz, 8H, H$_e$), 7.76-7.73 (m, 8H, H$_c$, H$_r$), 7.65-7.60 (m, 12H, H$_h$, H$_p$, H$_s$), 7.47 (s, 4H, H$_n$), 7.29-7.27 (m, 8H, H$_{n'}$, H$_q$), 2.03 (s, 12H, H$_o$), 1.93 (s, 12H, H$_{o'}$).

Diffusion coefficient (400 MHz, 3:1 d_6-DMSO/CDCl$_3$) D: 8.68 × 10^{-11} m^{2}s^{-1}.

^{13}C NMR (101 MHz, 3:1 d_6-DMSO/CDCl$_3$) δ: 152.3 (C$_a$), 151.8 (C$_m$), 150.2 (C$_b$), 149.4 (C$_v$), 148.2 (C$_w$), 147.1 (C$_l$), 142.4, 142.1 (C$_d$), 140.5, 140.1, 139.6, 139.3 (C$_k$), 138.4 (C$_j$/C$_t$), 137.9, 137.8, 136.3, 136.1, 134.3, 133.5, 132.3 (C$_e$), 130.3, 130.1, 129.5, 128.1, 127.6, 127.4, 127.1, 127.0, 126.3, 125.5, 123.7 (C$_j$/C$_t$), 123.5, 122.9, 120.2, 120.1, 117.5, 95.0, 94.9, 84.3, 82.9, 20.5 (C$_o$), 20.3 (C$_{o'}$).

ESI-MS m/z = 969.48 {[Pd$_3$(**L2^{Xy}**)$_4$(HCO$_2$)$_3$]$^{3+}$ calc. 969.57; 1477.36 {[Pd$_3$(**L2^{Xy}**)$_4$(HCO$_2$)$_4$]$^{2+}$ calc. 1477.23.

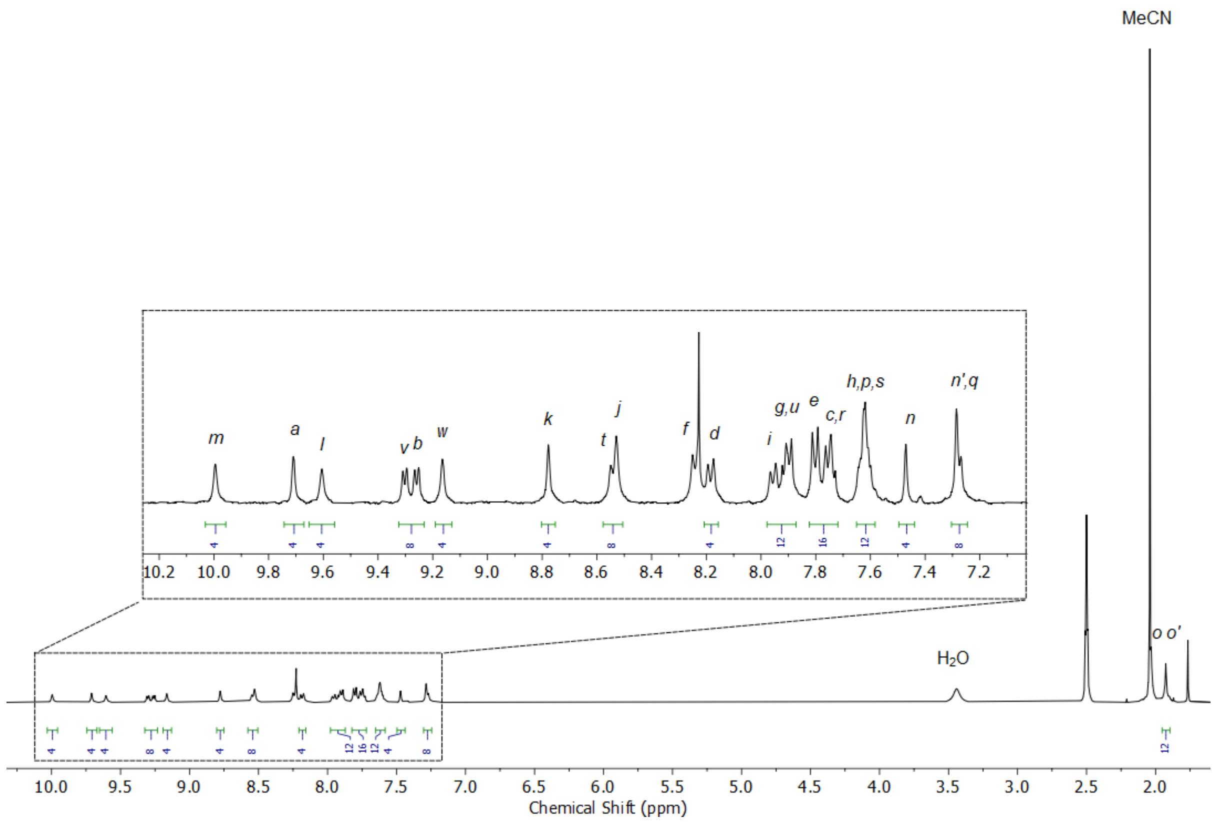

Figure S160 ^{1}H NMR (400 MHz, 3:1 d_6-DMSO/$CDCl_3$) of $[Pd_3(\mathbf{L2^{Xy}})_4](BF_4)_6$.

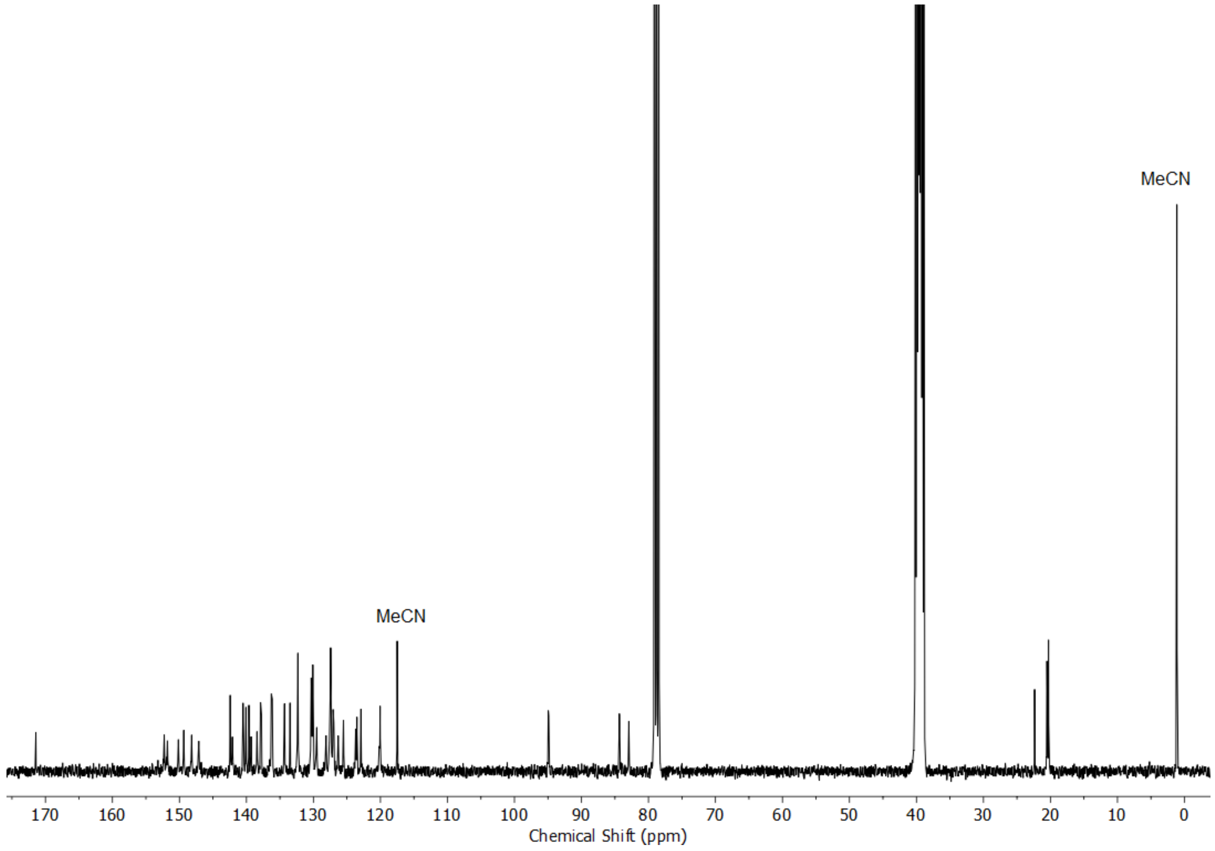

Figure S161 ^{13}C NMR (101 MHz, 3:1 d_6-DMSO/$CDCl_3$) of $[Pd_3(\mathbf{L2^{Xy}})_4](BF_4)_6$.

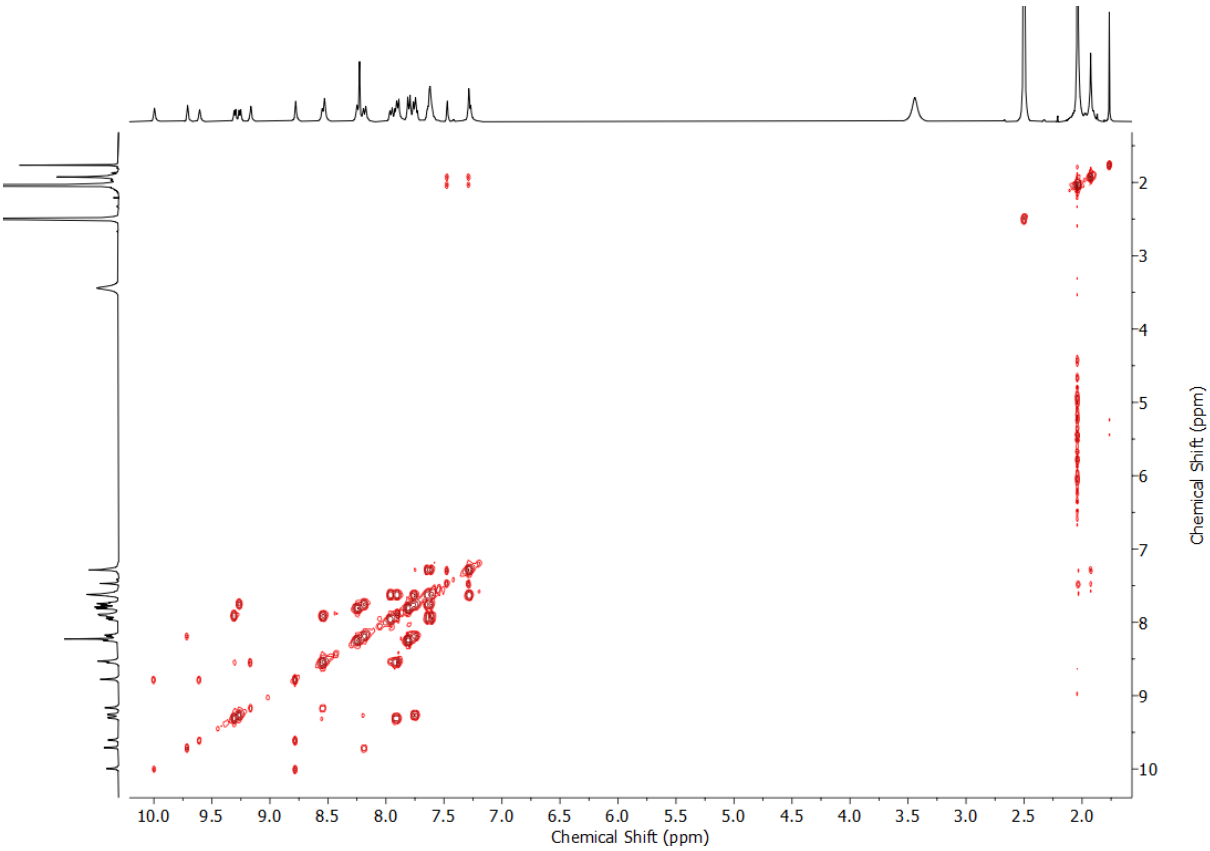

Figure S162 COSY NMR (3:1 d_6-DMSO/$CDCl_3$) of $[Pd_3(\mathbf{L2^{Xy}})_4](BF_4)_6$.

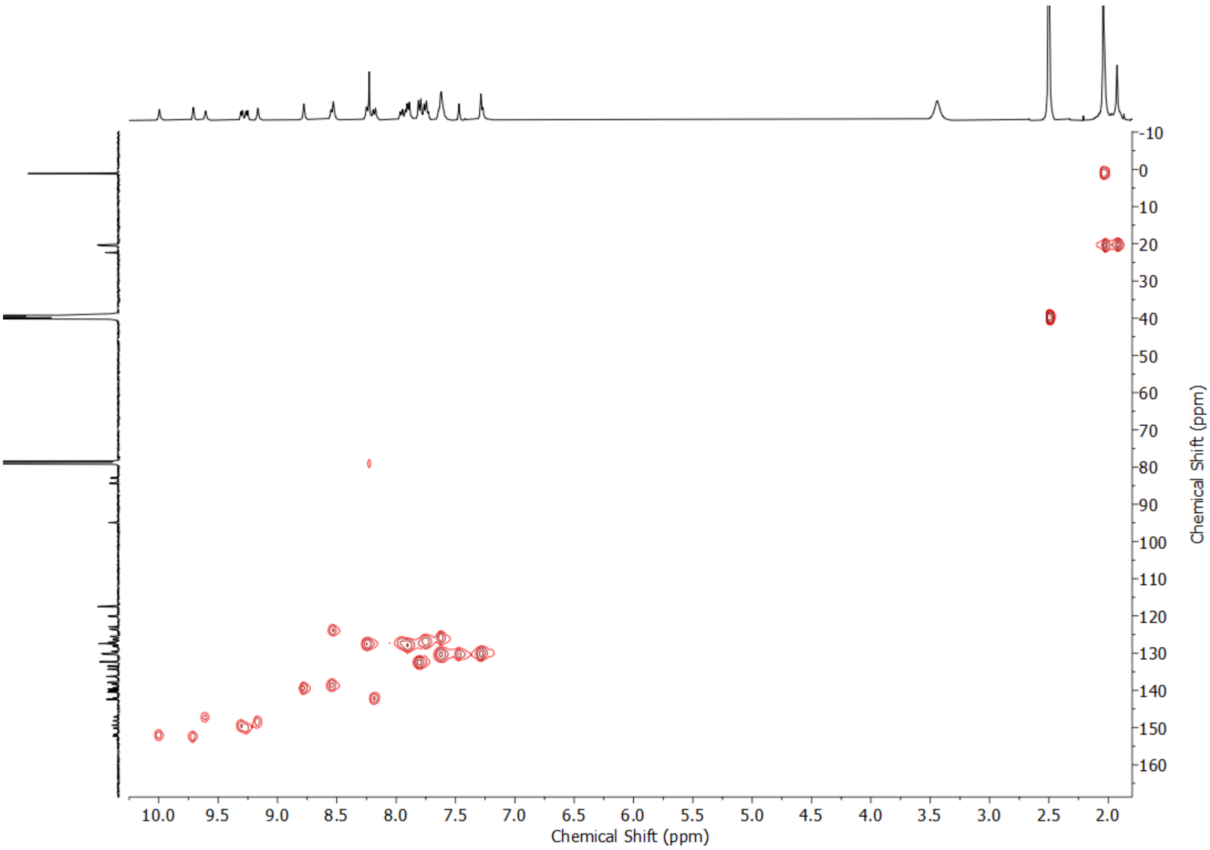

Figure S163 HSQC NMR (3:1 d_6-DMSO/$CDCl_3$) of $[Pd_3(\mathbf{L2^{Xy}})_4](BF_4)_6$.

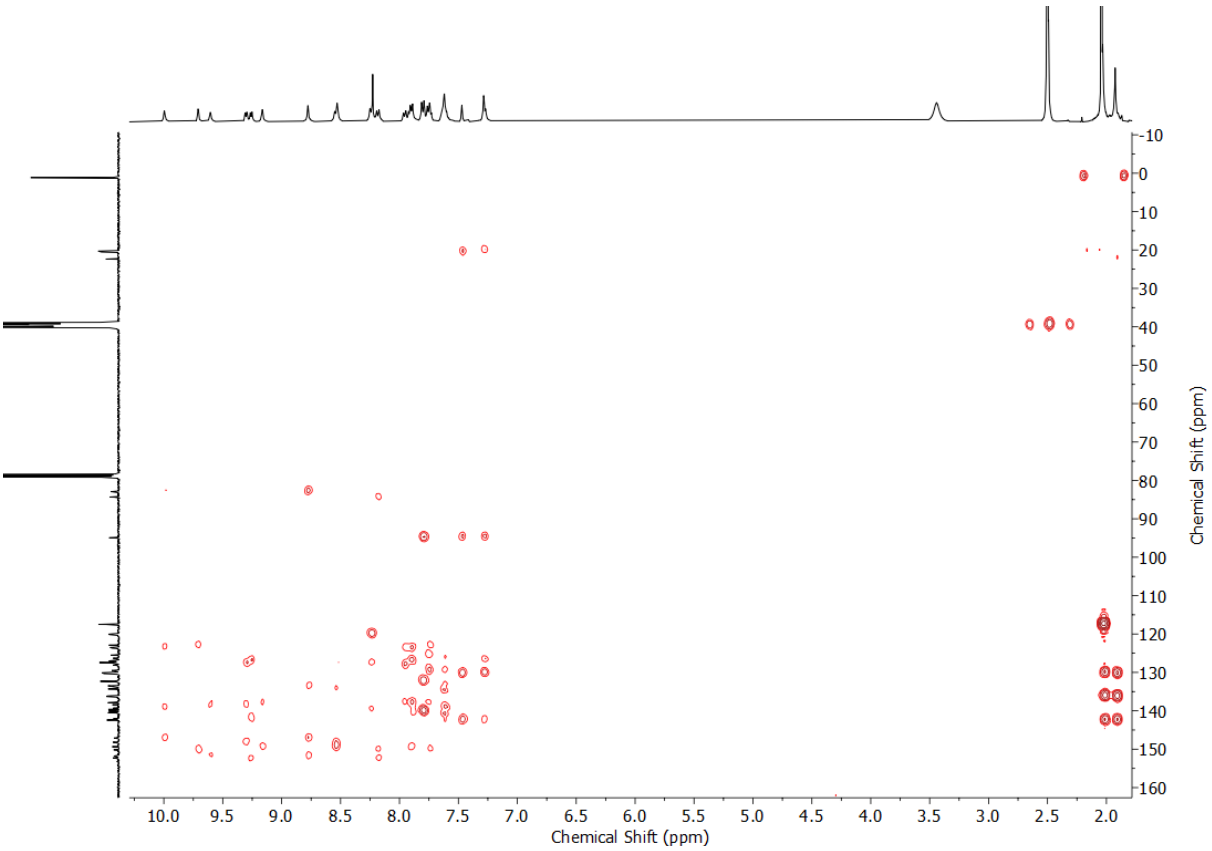

Figure S164 HMBC NMR (3:1 d_6-DMSO/CDCl$_3$) of [Pd$_3$(**L2**^{Xy})$_4$](BF$_4$)$_6$.

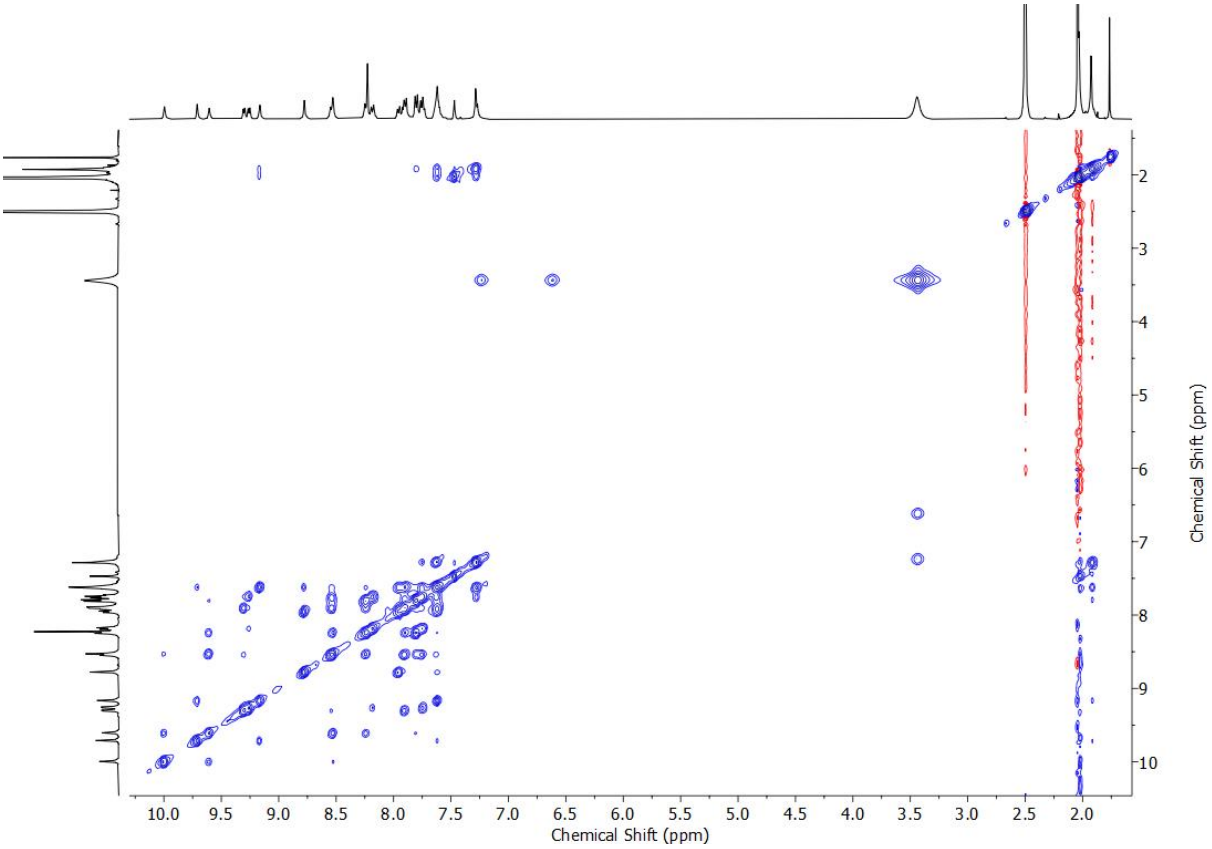

Figure S165 NOESY (3:1 d_6-DMSO/CDCl$_3$) of [Pd$_3$(**L2**^{Xy})$_4$](BF$_4$)$_6$.

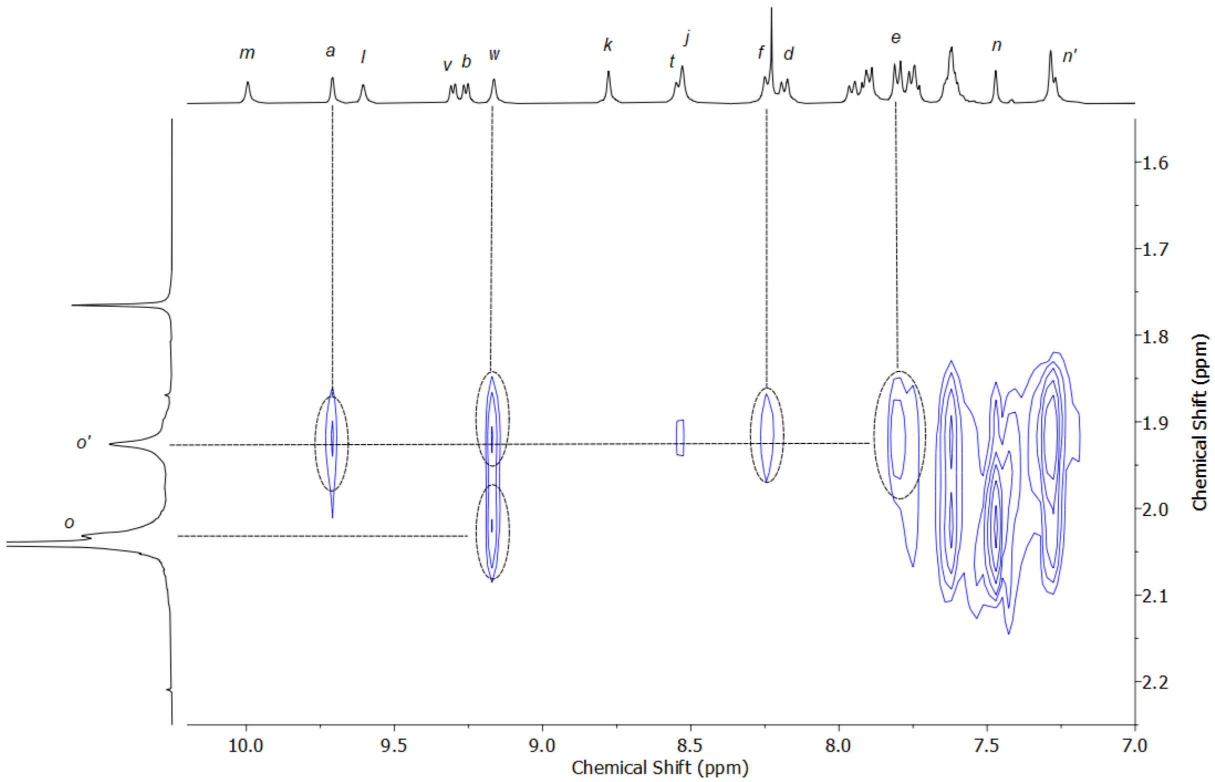

Figure S166 Partial NOESY (3:1 d_6-DMSO/$CDCl_3$) of $[Pd_3(\mathbf{L2^{Xy}})_4](BF_4)_6$.

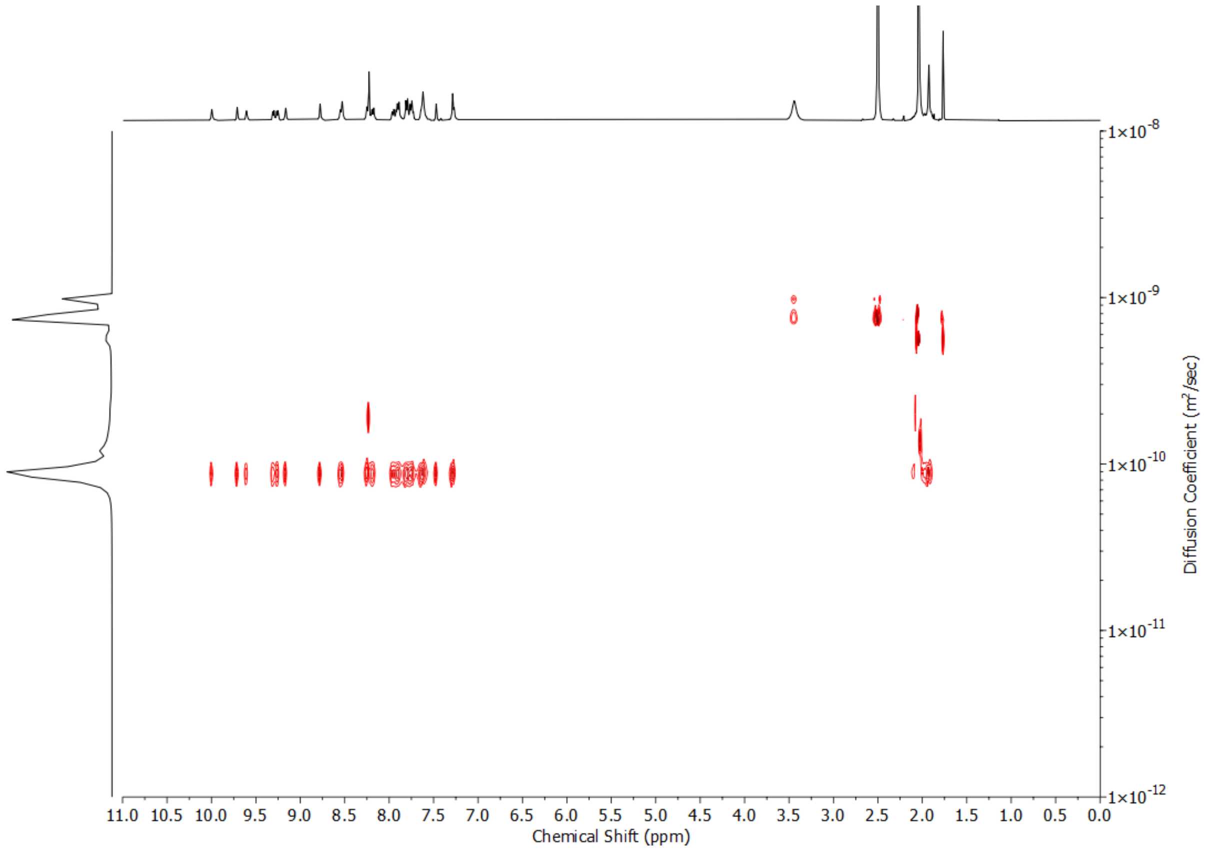

Figure S167 DOSY (3:1 d_6-DMSO/$CDCl_3$) of $[Pd_3(\mathbf{L2^{Xy}})_4](BF_4)_6$.

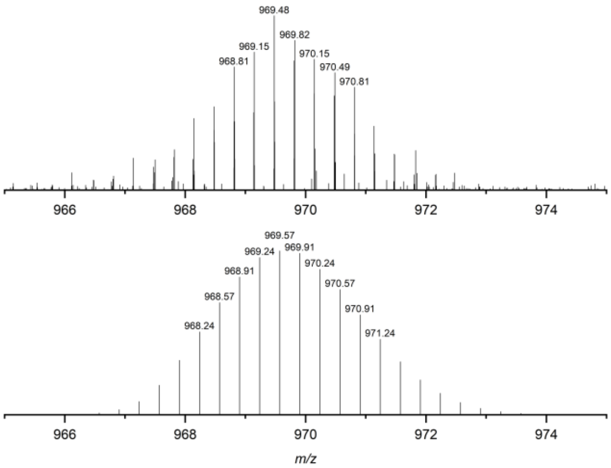

Figure S168 Observed (top) and calculated (bottom) isotopic patterns for $\{[Pd_3(\mathbf{L2^{Xy}})_4(HCO_2)_3]^{3+}$.

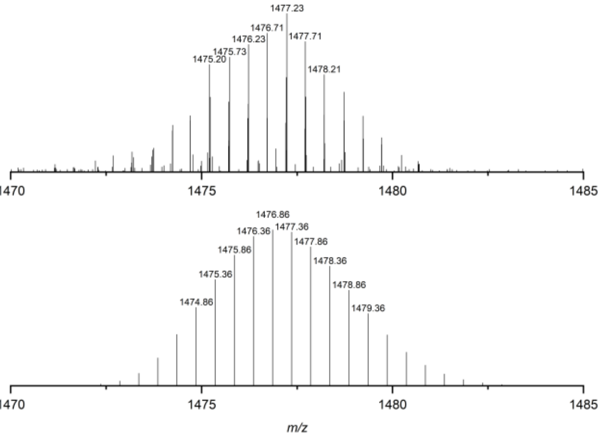

Figure S169 Observed (top) and calculated (bottom) isotopic patterns for $\{[Pd_3(\mathbf{L2^{Xy}})_4(HCO_2)_4]^{2+}$.

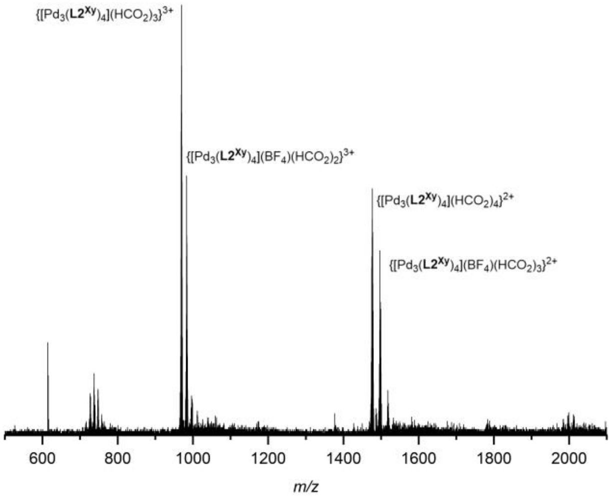

Figure S170 ESI-MS of $[Pd_3(\mathbf{L2^{Xy}})_4](BF_4)_6$.

3. Geometry Optimised Structures

Geometry optimisations were performed using the semi-empirical method PM6[2] in Gaussian 16.[3]

C2^Ph

Calculation Type = FOPT
Calculation Method = RPM6
Formula = $C_{172}H_{108}N_{12}Pd_3$
Basis Set = ZDO
Charge = 6
Spin = Singlet
Solvation = None
E(RPM6) = 3.7386819 Hartree
RMS Gradient Norm = 3.2e-08 Hartree/Bohr
Dipole Moment = 0.84660001 Debye
Point Group = C1
Molecular Mass = 2658.5924 amu

Maximum force = 0
RMS force = 0
Maximum displacement = 0.000714
RMS displacement = 8.8e-05
Predicted energy change = -3.740876e-11 Hartree

Atom coordinates:
1 Pd1 -0.0000 0.0000 -0.0161 Pd
2 N2 -0.5059 -1.3334 1.4511 N
3 C3 -1.8163 -1.6402 1.6397 C
4 C4 0.4288 -1.8862 2.2687 C
5 C5 -2.2352 -2.5471 2.6438 C
6 H6 -2.5493 -1.1593 0.9786 H
7 H7 1.4732 -1.5954 2.0872 H
8 C8 -1.2581 -3.1287 3.4697 C
9 H9 -1.5643 -3.8504 4.2390 H
10 N10 0.5059 1.3332 1.4512 N
11 C11 -0.4288 1.8860 2.2689 C
12 C12 1.8163 1.6400 1.6399 C
13 C13 -0.0973 2.7888 3.3105 C
14 H14 -1.4732 1.5952 2.0874 H
15 H15 2.5493 1.1592 0.9787 H
16 C16 1.2581 3.1283 3.4701 C
17 H17 1.5643 3.8499 4.2394 H
18 N18 -0.5029 -1.3355 -1.4782 N
19 C19 0.4295 -1.8785 -2.3046 C
20 C20 -1.8112 -1.6604 -1.6493 C
21 H21 1.4720 -1.5714 -2.1393 H

22	C22	-2.2305	-2.5780	-2.6442	C
23	H23	-2.5432	-1.1844	-0.9838	H
24	C24	-1.2548	-3.1503	-3.4788	C
25	H25	-1.5597	-3.8798	-4.2406	H
26	N26	0.5029	1.3357	-1.4780	N
27	C27	1.8112	1.6606	-1.6492	C
28	C28	-0.4295	1.8788	-2.3044	C
29	H29	2.5432	1.1846	-0.9837	H
30	C30	-0.0973	2.7913	-3.3377	C
31	H31	-1.4720	1.5717	-2.1391	H
32	C32	1.2548	3.1507	-3.4785	C
33	H33	1.5597	3.8803	-4.2402	H
34	C34	-1.1392	3.3213	4.2092	C
35	C35	-0.7959	3.7555	5.5028	C
36	C36	-2.4852	3.3804	3.8018	C
37	C37	-1.7782	4.2452	6.3677	C
38	H38	0.2381	3.7103	5.8503	H
39	C39	-3.4716	3.8489	4.6798	C
40	H40	-2.7726	3.0786	2.7966	H
41	C41	-3.1136	4.2934	5.9643	C
42	H42	-1.4993	4.5905	7.3689	H
43	H43	-3.8794	4.6690	6.6496	H
44	C44	-4.8981	3.8485	4.2796	C
45	C45	-5.5217	2.6346	3.9410	C
46	C46	-5.6409	5.0391	4.2721	C
47	C47	-6.8743	2.6089	3.6077	C
48	H48	-4.9507	1.7078	3.9665	H
49	C49	-6.9967	5.0208	3.9384	C
50	H50	-5.1608	5.9859	4.5352	H
51	C51	-7.6213	3.8040	3.6058	C
52	H52	-7.3597	1.6650	3.3641	H
53	H53	-7.5729	5.9499	3.9448	H
54	C54	-9.0002	3.7658	3.2804	C
55	C55	-10.1770	3.7094	2.9968	C
56	C56	-11.5480	3.6546	2.6709	C
57	C57	-12.4769	4.5650	3.2214	C
58	C58	-12.0220	2.6757	1.7682	C
59	C59	-13.8182	4.4730	2.8536	C
60	H60	-12.1412	5.3329	3.9307	H
61	H61	-11.3381	1.9399	1.3256	H
62	C62	-14.2204	3.4830	1.9400	C
63	H63	-14.5594	5.1683	3.2704	H
64	H64	-15.2700	3.3937	1.6233	H
65	C65	-3.6028	-2.8740	-2.7714	C
66	C66	-4.7882	-3.1089	-2.8699	C
67	C67	-6.1813	-3.3513	-2.9523	C
68	C68	-6.7627	-3.8181	-4.1465	C
69	C69	-6.9927	-3.1159	-1.8232	C

70	C70	-8.1401	-4.0369	-4.2110	C
71	H71	-6.1381	-4.0130	-5.0218	H
72	C72	-8.3663	-3.3357	-1.8953	C
73	H73	-6.5436	-2.7701	-0.8933	H
74	C74	-8.9493	-3.7954	-3.0898	C
75	H75	-8.5892	-4.4004	-5.1403	H
76	H76	-8.9908	-3.1701	-1.0183	H
77	C77	-10.4093	-4.0418	-3.1466	C
78	C78	-11.3131	-2.9923	-2.9273	C
79	C79	-10.8866	-5.3373	-3.4049	C
80	C80	-12.6962	-3.2396	-2.9485	C
81	H81	-10.9354	-1.9852	-2.7677	H
82	C82	-12.2617	-5.5803	-3.4268	C
83	H83	-10.1854	-6.1562	-3.5858	H
84	C84	-13.1668	-4.5408	-3.1955	C
85	H85	-12.6339	-6.5907	-3.6283	H
86	H86	-14.2381	-4.7537	-3.2040	H
87	C87	-13.6607	-2.1487	-2.7046	C
88	C88	-14.8555	-2.0458	-3.4465	C
89	C89	-13.4236	-1.1853	-1.7027	C
90	C90	-15.7645	-1.0298	-3.1542	C
91	H91	-15.0685	-2.7603	-4.2533	H
92	H92	-12.5048	-1.2126	-1.1028	H
93	C93	-15.4705	-0.1146	-2.1294	C
94	H94	-16.7041	-0.9430	-3.7157	H
95	H95	-16.1728	0.6889	-1.8633	H
96	N96	-14.3104	-0.1867	-1.4201	N
97	N97	-13.3344	2.5972	1.4063	N
98	C98	-1.1345	3.3115	-4.2490	C
99	C99	-2.4875	3.3499	-3.8620	C
100	C100	-0.7778	3.7535	-5.5363	C
101	C101	-3.4677	3.7951	-4.7585	C
102	H102	-2.7862	3.0394	-2.8630	H
103	C103	-1.7540	4.2267	-6.4172	C
104	H104	0.2624	3.7275	-5.8664	H
105	C105	-3.0969	4.2448	-6.0377	C
106	H106	-1.4644	4.5825	-7.4114	H
107	H107	-3.8581	4.6138	-6.7320	H
108	C108	-4.9011	3.7705	-4.3833	C
109	C109	-5.7894	2.9663	-5.1180	C
110	C110	-5.3819	4.5480	-3.3188	C
111	C111	-7.1429	2.9292	-4.7866	C
112	H112	-5.4214	2.3759	-5.9599	H
113	C113	-6.7369	4.5187	-2.9839	C
114	H114	-4.7007	5.1988	-2.7683	H
115	H115	-7.8315	2.3136	-5.3687	H
116	H116	-7.1112	5.1411	-2.1696	H
117	C117	-3.6102	-2.8277	2.7901	C

118	C118	-4.7941	-3.0582	2.9089	C
119	C119	-6.1870	-3.3009	3.0150	C
120	C120	-6.8273	-4.1290	2.0720	C
121	C121	-6.9340	-2.7135	4.0540	C
122	C122	-8.2018	-4.3445	2.1573	C
123	H123	-6.2466	-4.6144	1.2860	H
124	C124	-8.3107	-2.9289	4.1295	C
125	H125	-6.4362	-2.1007	4.8074	H
126	C126	-8.9527	-3.7387	3.1796	C
127	H127	-8.6955	-4.9994	1.4361	H
128	H128	-8.8872	-2.4868	4.9455	H
129	C129	-10.4146	-3.9721	3.2500	C
130	C130	-11.3108	-2.9140	3.0448	C
131	C131	-10.8996	-5.2647	3.5085	C
132	C132	-12.6955	-3.1523	3.0716	C
133	H133	-10.9277	-1.9099	2.8816	H
134	C134	-12.2763	-5.4951	3.5517	C
135	H135	-10.2023	-6.0880	3.6862	H
136	C136	-13.1750	-4.4482	3.3276	C
137	H137	-12.6548	-6.5010	3.7635	H
138	H138	-14.2480	-4.6512	3.3487	H
139	C139	-13.6530	-2.0576	2.8182	C
140	C140	-14.8333	-1.9215	3.5772	C
141	C141	-13.4237	-1.1266	1.7844	C
142	C142	-15.7370	-0.9047	3.2707	C
143	H143	-15.0392	-2.6110	4.4073	H
144	H144	-12.5156	-1.1817	1.1702	H
145	C145	-15.4523	-0.0234	2.2139	C
146	H146	-16.6656	-0.7914	3.8456	H
147	H147	-16.1515	0.7788	1.9363	H
148	N148	-14.3059	-0.1284	1.4867	N
149	Pd149	-13.8591	1.2001	0.0054	Pd
150	N150	-13.3325	2.5363	-1.4537	N
151	C151	-12.0182	2.6086	-1.8106	C
152	C152	-14.2228	3.3855	-2.0379	C
153	C153	-11.5473	3.5432	-2.7603	C
154	H154	-11.3304	1.9033	-1.3265	H
155	C155	-13.8234	4.3308	-2.9985	C
156	H156	-15.2736	3.3030	-1.7235	H
157	C157	-12.4804	4.4153	-3.3628	C
158	H158	-14.5674	4.9977	-3.4546	H
159	H159	-12.1461	5.1492	-4.1084	H
160	C160	-10.1749	3.5947	-3.0843	C
161	C161	-8.9997	3.6547	-3.3726	C
162	C162	-7.6228	3.7012	-3.7105	C
163	Pd163	13.8591	-1.2002	0.0053	Pd
164	N164	13.3343	-2.5974	1.4060	N
165	C165	12.0220	-2.6759	1.7679	C

166	C166	14.2204	-3.4833	1.9396	C
167	C167	11.5480	-3.6550	2.6705	C
168	H168	11.3381	-1.9401	1.3254	H
169	C169	13.8182	-4.4734	2.8530	C
170	H170	15.2700	-3.3940	1.6229	H
171	C171	12.4769	-4.5654	3.2209	C
172	H172	14.5594	-5.1687	3.2697	H
173	H173	12.1412	-5.3334	3.9301	H
174	N174	14.3059	0.1282	1.4867	N
175	C175	13.4237	1.1264	1.7845	C
176	C176	15.4523	0.0231	2.2139	C
177	C177	13.6531	2.0572	2.8184	C
178	H178	12.5156	1.1815	1.1704	H
179	C179	15.7370	0.9043	3.2708	C
180	H180	16.1515	-0.7791	1.9362	H
181	C181	14.8334	1.9211	3.5774	C
182	H182	16.6656	0.7909	3.8457	H
183	H183	15.0392	2.6104	4.4076	H
184	N184	13.3325	-2.5362	-1.4540	N
185	C185	14.2227	-3.3854	-2.0383	C
186	C186	12.0182	-2.6084	-1.8110	C
187	C187	13.8233	-4.3305	-2.9990	C
188	H188	15.2736	-3.3029	-1.7239	H
189	C189	11.5473	-3.5429	-2.7607	C
190	H190	11.3304	-1.9031	-1.3267	H
191	C191	12.4803	-4.4149	-3.3633	C
192	H192	14.5674	-4.9974	-3.4551	H
193	H193	12.1460	-5.1488	-4.1090	H
194	N194	14.3104	0.1868	-1.4201	N
195	C195	15.4705	0.1148	-2.1294	C
196	C196	13.4236	1.1855	-1.7026	C
197	C197	15.7645	1.0301	-3.1541	C
198	H198	16.1728	-0.6887	-1.8634	H
199	C199	13.6607	2.1490	-2.7044	C
200	H200	12.5048	1.2127	-1.1027	H
201	C201	14.8555	2.0462	-3.4463	C
202	H202	16.7041	0.9434	-3.7156	H
203	H203	15.0685	2.7607	-4.2530	H
204	C204	12.6955	3.1519	3.0719	C
205	C205	13.1750	4.4477	3.3281	C
206	C206	11.3108	2.9137	3.0452	C
207	C207	12.2763	5.4946	3.5523	C
208	H208	14.2480	4.6508	3.3492	H
209	C209	10.4146	3.9717	3.2504	C
210	H210	10.9278	1.9095	2.8818	H
211	C211	10.8996	5.2643	3.5091	C
212	H212	12.6548	6.5005	3.7643	H
213	H213	10.2023	6.0876	3.6869	H

214	C214	8.9527	3.7383	3.1800	C
215	C215	8.3107	2.9284	4.1298	C
216	C216	8.2018	4.3442	2.1578	C
217	C217	6.9340	2.7130	4.0543	C
218	H218	8.8872	2.4862	4.9458	H
219	C219	6.8274	4.1288	2.0725	C
220	H220	8.6955	4.9993	1.4366	H
221	C221	6.1870	3.3005	3.0153	C
222	H222	6.4362	2.1002	4.8077	H
223	H223	6.2466	4.6142	1.2866	H
224	C224	4.7941	3.0579	2.9092	C
225	C225	10.1749	-3.5943	-3.0848	C
226	C226	8.9997	-3.6543	-3.3730	C
227	C227	7.6228	-3.7008	-3.7110	C
228	C228	7.1429	-2.9286	-4.7870	C
229	C229	6.7369	-4.5184	-2.9845	C
230	C230	5.7894	-2.9656	-5.1183	C
231	H231	7.8315	-2.3130	-5.3689	H
232	C232	5.3819	-4.5476	-3.3194	C
233	H233	7.1112	-5.1409	-2.1702	H
234	C234	4.9011	-3.7699	-4.3838	C
235	H235	5.4214	-2.3751	-5.9602	H
236	H236	4.7007	-5.1985	-2.7690	H
237	C237	3.4677	-3.7945	-4.7590	C
238	C238	2.4875	-3.3494	-3.8624	C
239	C239	3.0969	-4.2440	-6.0382	C
240	H240	2.7862	-3.0390	-2.8633	H
241	C241	1.7540	-4.2258	-6.4177	C
242	H242	3.8580	-4.6129	-6.7325	H
243	C243	0.7778	-3.7527	-5.5368	C
244	H244	1.4643	-4.5815	-7.4120	H
245	H245	-0.2624	-3.7267	-5.8669	H
246	C246	12.6962	3.2398	-2.9481	C
247	C247	11.3131	2.9926	-2.9270	C
248	C248	13.1668	4.5411	-3.1950	C
249	C249	10.4094	4.0421	-3.1462	C
250	H250	10.9354	1.9855	-2.7675	H
251	C251	12.2618	5.5807	-3.4261	C
252	H252	14.2381	4.7540	-3.2035	H
253	C253	10.8866	5.3376	-3.4043	C
254	H254	12.6340	6.5911	-3.6276	H
255	H255	10.1854	6.1566	-3.5851	H
256	C256	8.9493	3.7957	-3.0894	C
257	C257	8.1401	4.0374	-4.2105	C
258	C258	8.3663	3.3359	-1.8949	C
259	C259	6.7628	3.8186	-4.1460	C
260	H260	8.5893	4.4010	-5.1398	H
261	C261	6.9927	3.1161	-1.8229	C

262	H262	8.9908	3.1702	-1.0180	H
263	H263	6.1382	4.0135	-5.0213	H
264	H264	6.5436	2.7702	-0.8930	H
265	C265	10.1770	-3.7097	2.9964	C
266	C266	9.0002	-3.7662	3.2800	C
267	C267	7.6213	-3.8044	3.6054	C
268	C268	6.9967	-5.0212	3.9379	C
269	C269	6.8743	-2.6093	3.6074	C
270	C270	5.6409	-5.0395	4.2716	C
271	H271	7.5729	-5.9504	3.9443	H
272	C272	5.5217	-2.6350	3.9407	C
273	H273	7.3597	-1.6654	3.3638	H
274	C274	4.8981	-3.8489	4.2792	C
275	H275	5.1608	-5.9864	4.5346	H
276	H276	4.9507	-1.7082	3.9663	H
277	C277	3.4716	-3.8494	4.6794	C
278	C278	2.4852	-3.3808	3.8014	C
279	C279	3.1136	-4.2939	5.9638	C
280	H280	2.7726	-3.0789	2.7963	H
281	C281	1.7782	-4.2458	6.3672	C
282	H282	3.8794	-4.6696	6.6491	H
283	C283	0.7959	-3.7560	5.5024	C
284	H284	1.4993	-4.5912	7.3684	H
285	H285	-0.2380	-3.7108	5.8499	H
286	C286	4.7882	3.1092	-2.8696	C
287	C287	6.1813	3.3516	-2.9519	C
288	C288	1.1344	-3.3110	-4.2494	C
289	C289	0.0973	-2.7909	-3.3381	C
290	C290	2.2352	2.5468	2.6441	C
291	C291	2.2305	2.5783	-2.6439	C
292	C292	0.0973	-2.7892	3.3102	C
293	C293	1.1392	-3.3217	4.2088	C
294	C294	3.6028	2.8743	-2.7710	C
295	C295	3.6102	2.8274	2.7904	C

C2^{Xy}

Calculation Type = FOPT
Calculation Method = RPM6
Formula = $C_{180}H_{124}N_{12}Pd_3$
Basis Set = ZDO
Charge = 6
Spin = Singlet
Solvation = None
E(RPM6) = 3.6079528 Hartree
RMS Gradient Norm = 3e-08 Hartree/Bohr
Dipole Moment = 2.486 Debye
Point Group = C1
Molecular Mass = 2770.7176 amu

Maximum force = 0
RMS force = 0
Maximum displacement = 5.6e-05
RMS displacement = 9e-06
Predicted energy change = -1.065522e-11 Hartree

Atom Coordinates:
1 Pd1 -0.0000 -0.4068 -0.0000 Pd
2 N2 0.4711 1.0435 -1.3602 N
3 C3 1.7091 1.6026 -1.3039 C
4 C4 -0.4015 1.4504 -2.3200 C
5 C5 2.1186 2.6015 -2.2210 C
6 H6 2.3872 1.2501 -0.5160 H
7 H7 -1.3778 0.9471 -2.3442 H
8 C8 1.1962 3.0413 -3.1876 C
9 H9 1.4836 3.8417 -3.8822 H
10 N10 -0.4711 1.0435 1.3602 N
11 C11 0.4015 1.4504 2.3199 C
12 C12 -1.7091 1.6026 1.3039 C
13 C13 0.0815 2.4615 3.2590 C
14 H14 1.3778 0.9471 2.3442 H
15 H15 -2.3872 1.2501 0.5160 H
16 C16 -1.1962 3.0413 3.1876 C
17 H17 -1.4836 3.8417 3.8822 H
18 N18 0.4628 -1.8473 -1.3636 N
19 C19 -0.4733 -2.7118 -1.8373 C
20 C20 1.7451 -1.9521 -1.7986 C
21 H21 -1.4916 -2.6029 -1.4379 H
22 C22 2.1348 -2.9312 -2.7472 C
23 H23 2.4800 -1.2476 -1.3878 H
24 C24 1.1540 -3.8033 -3.2512 C
25 H25 1.4326 -4.5486 -4.0076 H
26 N26 -0.4628 -1.8473 1.3636 N

27	C27	-1.7451	-1.9521	1.7986	C
28	C28	0.4733	-2.7118	1.8373	C
29	H29	-2.4800	-1.2476	1.3878	H
30	C30	0.1711	-3.7159	2.7901	C
31	H31	1.4916	-2.6029	1.4378	H
32	C32	-1.1540	-3.8033	3.2512	C
33	H33	-1.4326	-4.5486	4.0076	H
34	C34	1.0538	2.8712	4.2908	C
35	C35	0.5978	3.2759	5.5580	C
36	C36	2.4350	2.8552	4.0259	C
37	C37	1.5103	3.6712	6.5397	C
38	H38	-0.4681	3.2802	5.7915	H
39	C39	3.3495	3.2255	5.0222	C
40	H40	2.8050	2.5642	3.0455	H
41	C41	2.8814	3.6474	6.2790	C
42	H42	1.1483	4.0005	7.5193	H
43	H43	3.5891	3.9646	7.0510	H
44	C44	4.8072	3.1505	4.7709	C
45	C45	5.3839	3.7968	3.6654	C
46	C46	5.6237	2.4240	5.6553	C
47	C47	6.7567	3.7035	3.4349	C
48	H48	4.7607	4.3951	2.9999	H
49	C49	6.9965	2.3306	5.4325	C
50	H50	5.1838	1.9348	6.5278	H
51	C51	7.5698	2.9615	4.3110	C
52	H52	7.2061	4.2191	2.5752	H
53	H53	7.6275	1.7775	6.1317	H
54	C54	8.9626	2.8360	4.0743	C
55	C55	10.1491	2.6954	3.8742	C
56	C56	11.5311	2.5140	3.6525	C
57	C57	12.5052	3.2107	4.4012	C
58	C58	11.9683	1.6032	2.6659	C
59	C59	13.8548	2.9754	4.1444	C
60	H60	12.1972	3.9258	5.1757	H
61	H61	11.2449	1.0391	2.0620	H
62	C62	14.2221	2.0516	3.1493	C
63	H63	14.6313	3.5041	4.7138	H
64	H64	15.2800	1.8441	2.9257	H
65	C65	3.4897	-3.0004	-3.1306	C
66	C66	4.6656	-3.0391	-3.4220	C
67	C67	6.0553	-3.0417	-3.7192	C
68	C68	6.7852	-4.2378	-3.6954	C
69	C69	6.6927	-1.8241	-4.0138	C
70	C70	8.1686	-4.2228	-3.9432	C
71	H71	6.2808	-5.1840	-3.4965	H
72	C72	8.0702	-1.7996	-4.2677	C
73	H73	6.1135	-0.9044	-4.0632	H
74	C74	8.8147	-2.9976	-4.2090	C

75	C75	10.2772	-2.9615	-4.4510	C
76	C76	11.1633	-2.7871	-3.3780	C
77	C77	10.7728	-3.0889	-5.7590	C
78	C78	12.5468	-2.7329	-3.6154	C
79	H79	10.7725	-2.7167	-2.3663	H
80	C80	12.1487	-3.0339	-5.9899	C
81	H81	10.0825	-3.2350	-6.5935	H
82	C82	13.0377	-2.8539	-4.9255	C
83	H83	12.5355	-3.1358	-7.0092	H
84	H84	14.1095	-2.8055	-5.1259	H
85	C85	13.4947	-2.5587	-2.4966	C
86	C86	14.6489	-3.3608	-2.3940	C
87	C87	13.2802	-1.5776	-1.5061	C
88	C88	15.5404	-3.1563	-1.3409	C
89	H89	14.8432	-4.1453	-3.1382	H
90	H90	12.3980	-0.9238	-1.5408	H
91	C91	15.2680	-2.1565	-0.3936	C
92	H92	16.4474	-3.7680	-1.2507	H
93	H93	15.9525	-1.9645	0.4453	H
94	N94	14.1493	-1.3820	-0.4725	N
95	N95	13.2915	1.3756	2.4216	N
96	C96	1.2201	-4.6474	3.2479	C
97	C97	2.5694	-4.2485	3.2797	C
98	C98	0.8806	-5.9563	3.6332	C
99	C99	3.5665	-5.1559	3.6615	C
100	H100	2.8518	-3.2319	3.0149	H
101	C101	1.8748	-6.8494	4.0431	C
102	H102	-0.1574	-6.2927	3.6102	H
103	C103	3.2150	-6.4589	4.0536	C
104	H104	1.6010	-7.8625	4.3556	H
105	H105	3.9886	-7.1636	4.3730	H
106	C106	4.9932	-4.7566	3.6261	C
107	C107	5.8814	-5.4407	2.7787	C
108	C108	5.4658	-3.7071	4.4294	C
109	C109	7.2241	-5.0692	2.7185	C
110	H110	5.5220	-6.2734	2.1702	H
111	C111	6.8093	-3.3327	4.3773	C
112	H112	4.7865	-3.1989	5.1155	H
113	H113	7.9123	-5.6108	2.0666	H
114	H114	7.1772	-2.5296	5.0172	H
115	C115	3.4347	3.1048	-2.1676	C
116	C116	4.5747	3.5157	-2.1463	C
117	C117	5.9238	3.9613	-2.1132	C
118	C118	6.6693	4.0179	-3.2996	C
119	C119	6.5055	4.3293	-0.8888	C
120	C120	8.0142	4.4218	-3.2671	C
121	H121	6.2031	3.7637	-4.2519	H
122	C122	7.8453	4.7397	-0.8468	C

123	H123	5.9104	4.3158	0.0217	H
124	C124	8.6090	4.7609	-2.0338	C
125	C125	10.0371	5.1565	-1.9793	C
126	C126	11.0251	4.1782	-1.7991	C
127	C127	10.3983	6.5090	-2.0912	C
128	C128	12.3769	4.5530	-1.7096	C
129	H129	10.7365	3.1318	-1.7474	H
130	C130	11.7426	6.8770	-2.0103	C
131	H131	9.6294	7.2704	-2.2434	H
132	C132	12.7318	5.9079	-1.8143	C
133	H133	12.0262	7.9311	-2.1005	H
134	H134	13.7754	6.2194	-1.7374	H
135	C135	13.4238	3.5350	-1.4919	C
136	C136	14.6711	3.6082	-2.1451	C
137	C137	13.2106	2.4635	-0.6000	C
138	C138	15.6485	2.6510	-1.8755	C
139	H139	14.8720	4.4139	-2.8641	H
140	H140	12.2535	2.3566	-0.0716	H
141	C141	15.3723	1.6202	-0.9615	C
142	H142	16.6284	2.7000	-2.3680	H
143	H143	16.1272	0.8588	-0.7169	H
144	N144	14.1652	1.5235	-0.3382	N
145	Pd145	13.7565	0.0082	0.9685	Pd
146	N146	13.2840	-1.4873	2.2863	N
147	C147	11.9916	-1.9171	2.3553	C
148	C148	14.2023	-2.0158	3.1424	C
149	C149	11.5701	-2.8827	3.2973	C
150	H150	11.2818	-1.4815	1.6398	H
151	C151	13.8544	-2.9936	4.0902	C
152	H152	15.2336	-1.6406	3.0638	H
153	C153	12.5330	-3.4297	4.1737	C
154	H154	14.6208	-3.4058	4.7602	H
155	H155	12.2381	-4.1887	4.9107	H
156	C156	10.2153	-3.2727	3.3599	C
157	C157	9.0555	-3.6144	3.4379	C
158	C158	7.6934	-4.0039	3.5111	C
159	Pd159	-13.7565	0.0082	-0.9685	Pd
160	N160	-13.2915	1.3756	-2.4216	N
161	C161	-11.9683	1.6032	-2.6659	C
162	C162	-14.2221	2.0516	-3.1493	C
163	C163	-11.5311	2.5140	-3.6525	C
164	H164	-11.2449	1.0391	-2.0620	H
165	C165	-13.8548	2.9754	-4.1443	C
166	H166	-15.2800	1.8441	-2.9257	H
167	C167	-12.5052	3.2107	-4.4012	C
168	H168	-14.6313	3.5041	-4.7138	H
169	H169	-12.1972	3.9258	-5.1756	H
170	N170	-14.1652	1.5235	0.3382	N

171 C171	-13.2106	2.4635	0.6000	C
172 C172	-15.3723	1.6202	0.9615	C
173 C173	-13.4238	3.5350	1.4919	C
174 H174	-12.2535	2.3566	0.0716	H
175 C175	-15.6485	2.6510	1.8756	C
176 H176	-16.1272	0.8588	0.7169	H
177 C177	-14.6711	3.6082	2.1451	C
178 H178	-16.6284	2.7000	2.3680	H
179 H179	-14.8720	4.4139	2.8641	H
180 N180	-13.2840	-1.4873	-2.2863	N
181 C181	-14.2023	-2.0158	-3.1424	C
182 C182	-11.9916	-1.9171	-2.3553	C
183 C183	-13.8544	-2.9936	-4.0902	C
184 H184	-15.2336	-1.6406	-3.0638	H
185 C185	-11.5701	-2.8827	-3.2973	C
186 H186	-11.2817	-1.4815	-1.6398	H
187 C187	-12.5330	-3.4297	-4.1737	C
188 H188	-14.6208	-3.4058	-4.7602	H
189 H189	-12.2381	-4.1887	-4.9107	H
190 N190	-14.1493	-1.3820	0.4725	N
191 C191	-15.2680	-2.1565	0.3936	C
192 C192	-13.2802	-1.5776	1.5061	C
193 C193	-15.5404	-3.1563	1.3409	C
194 H194	-15.9525	-1.9645	-0.4453	H
195 C195	-13.4947	-2.5587	2.4966	C
196 H196	-12.3980	-0.9238	1.5408	H
197 C197	-14.6489	-3.3608	2.3940	C
198 H198	-16.4474	-3.7680	1.2507	H
199 H199	-14.8432	-4.1453	3.1382	H
200 C200	-12.3769	4.5530	1.7096	C
201 C201	-12.7318	5.9078	1.8143	C
202 C202	-11.0252	4.1782	1.7991	C
203 C203	-11.7426	6.8770	2.0103	C
204 H204	-13.7754	6.2194	1.7375	H
205 C205	-10.0371	5.1564	1.9793	C
206 H206	-10.7365	3.1318	1.7474	H
207 C207	-10.3983	6.5090	2.0913	C
208 H208	-12.0262	7.9311	2.1006	H
209 H209	-9.6294	7.2704	2.2435	H
210 C210	-8.6090	4.7609	2.0338	C
211 C211	-7.8453	4.7397	0.8468	C
212 C212	-8.0142	4.4217	3.2671	C
213 C213	-6.5055	4.3293	0.8888	C
214 C214	-6.6693	4.0179	3.2996	C
215 C215	-5.9238	3.9613	2.1132	C
216 H216	-5.9104	4.3158	-0.0217	H
217 H217	-6.2031	3.7636	4.2519	H
218 C218	-4.5747	3.5157	2.1463	C

219	C219	-10.2153	-3.2727	-3.3599	C
220	C220	-9.0555	-3.6144	-3.4379	C
221	C221	-7.6934	-4.0039	-3.5111	C
222	C222	-7.2241	-5.0692	-2.7185	C
223	C223	-6.8093	-3.3327	-4.3774	C
224	C224	-5.8814	-5.4407	-2.7787	C
225	H225	-7.9123	-5.6107	-2.0666	H
226	C226	-5.4658	-3.7071	-4.4294	C
227	H227	-7.1772	-2.5296	-5.0172	H
228	C228	-4.9932	-4.7566	-3.6261	C
229	H229	-5.5220	-6.2734	-2.1702	H
230	H230	-4.7865	-3.1989	-5.1155	H
231	C231	-3.5665	-5.1559	-3.6615	C
232	C232	-2.5694	-4.2485	-3.2797	C
233	C233	-3.2150	-6.4589	-4.0536	C
234	H234	-2.8518	-3.2319	-3.0149	H
235	C235	-1.8748	-6.8493	-4.0432	C
236	H236	-3.9886	-7.1636	-4.3730	H
237	C237	-0.8806	-5.9563	-3.6332	C
238	H238	-1.6010	-7.8625	-4.3557	H
239	H239	0.1574	-6.2927	-3.6103	H
240	C240	-12.5468	-2.7329	3.6154	C
241	C241	-11.1633	-2.7871	3.3780	C
242	C242	-13.0377	-2.8539	4.9255	C
243	C243	-10.2772	-2.9615	4.4510	C
244	H244	-10.7725	-2.7167	2.3663	H
245	C245	-12.1487	-3.0339	5.9899	C
246	H246	-14.1095	-2.8055	5.1259	H
247	C247	-10.7728	-3.0889	5.7590	C
248	H248	-12.5355	-3.1358	7.0092	H
249	H249	-10.0825	-3.2350	6.5935	H
250	C250	-8.8147	-2.9976	4.2090	C
251	C251	-8.1686	-4.2228	3.9432	C
252	C252	-8.0702	-1.7996	4.2676	C
253	C253	-6.7852	-4.2378	3.6954	C
254	C254	-6.6927	-1.8241	4.0138	C
255	H255	-6.2808	-5.1840	3.4965	H
256	H256	-6.1135	-0.9044	4.0632	H
257	C257	-10.1491	2.6954	-3.8742	C
258	C258	-8.9626	2.8360	-4.0743	C
259	C259	-7.5698	2.9615	-4.3110	C
260	C260	-6.9965	2.3306	-5.4325	C
261	C261	-6.7567	3.7035	-3.4349	C
262	C262	-5.6237	2.4240	-5.6553	C
263	H263	-7.6275	1.7776	-6.1317	H
264	C264	-5.3839	3.7968	-3.6654	C
265	H265	-7.2061	4.2191	-2.5751	H
266	C266	-4.8072	3.1505	-4.7709	C

267	H267	-5.1838	1.9348	-6.5278	H
268	H268	-4.7607	4.3952	-2.9999	H
269	C269	-3.3495	3.2256	-5.0222	C
270	C270	-2.4350	2.8552	-4.0259	C
271	C271	-2.8814	3.6474	-6.2790	C
272	H272	-2.8050	2.5642	-3.0455	H
273	C273	-1.5103	3.6712	-6.5397	C
274	H274	-3.5891	3.9646	-7.0510	H
275	C275	-0.5978	3.2759	-5.5580	C
276	H276	-1.1483	4.0005	-7.5193	H
277	H277	0.4681	3.2802	-5.7915	H
278	C278	-4.6656	-3.0391	3.4220	C
279	C279	-6.0553	-3.0417	3.7192	C
280	C280	-1.2201	-4.6474	-3.2479	C
281	C281	-0.1711	-3.7159	-2.7901	C
282	C282	-2.1186	2.6014	2.2210	C
283	C283	-2.1348	-2.9312	2.7472	C
284	C284	-0.0815	2.4616	-3.2590	C
285	C285	-1.0538	2.8712	-4.2908	C
286	C286	-3.4897	-3.0004	3.1306	C
287	C287	-3.4347	3.1048	2.1676	C
288	C288	8.9292	-5.5035	-3.9432	C
289	H289	9.1511	-5.8272	-4.9768	H
290	H290	9.8995	-5.4229	-3.4319	H
291	H291	8.3855	-6.3348	-3.4727	H
292	C292	8.7287	-0.5077	-4.6232	C
293	H293	8.9003	-0.4544	-5.7137	H
294	H294	8.1282	0.3707	-4.3561	H
295	H295	9.7132	-0.3926	-4.1501	H
296	C296	8.7878	4.5085	-4.5372	C
297	H297	9.8057	4.1025	-4.4450	H
298	H298	8.3074	3.9913	-5.3791	H
299	H299	8.9056	5.5623	-4.8514	H
300	C300	8.4386	5.1771	0.4507	C
301	H301	9.4852	4.8637	0.5670	H
302	H302	8.4390	6.2809	0.5179	H
303	H303	7.8826	4.8084	1.3302	H
304	C304	-8.9292	-5.5035	3.9432	C
305	H305	-9.1510	-5.8272	4.9768	H
306	H306	-9.8995	-5.4229	3.4319	H
307	H307	-8.3855	-6.3348	3.4726	H
308	C308	-8.7287	-0.5077	4.6232	C
309	H309	-9.7131	-0.3926	4.1501	H
310	H310	-8.9004	-0.4544	5.7137	H
311	H311	-8.1282	0.3707	4.3561	H
312	C312	-8.7878	4.5085	4.5372	C
313	H313	-9.8057	4.1025	4.4450	H
314	H314	-8.3073	3.9913	5.3791	H

315	H315	-8.9057	5.5623	4.8514	H
316	C316	-8.4386	5.1772	-0.4507	C
317	H317	-9.4852	4.8637	-0.5669	H
318	H318	-8.4390	6.2809	-0.5179	H
319	H319	-7.8826	4.8085	-1.3302	H

4. Hydrodynamic Radii Calculations

Hydrodynamic radii were calculated using a variation of the Stokes-Einstein equation:

$$R_H = \frac{k_B T}{6\pi\eta D}$$

Where R_H is the hydrodynamic radius (m)

k_B is the Boltzmann constant (1.38×10^{-23} J K^{-1})

T is the temperature (K)

η is the solvent viscosity (2.180×10^{-3} kg $s^{-1}m^{-1}$ for d_6-DMSO)[4]

D is the diffusion coefficient (m^2s^{-1})

5. X-ray Crystallography

cis-$[Pd_2(L1^{Xy})_4](BF_4)_4$

$L1^{Xy}$ (10.8 mg, 30 µmol) and $[Pd(CH_3CN)_4](BF_4)_2$ (6.7 mg, 15 µmol) were sonicated in DMF (1 mL) until a homogenous solution was obtained. After standing at rt for 24 h, the solution was diluted with additional DMF (1 mL), filtered through celite and left for vapour diffusion of Et_2O, from which X-ray quality crystals were obtained.

Crystal data for **$C1^{Xy}$**: $[C_{104}H_{80}N_8Pd_2](BF_4)_4{\cdot}6.75(C_3H_7NO)$, M = 2495.19, triclinic, P–1 (no. 2), a = 12.7020(6), b = 22.5260(12), c = 23.6140(12) Å, α = 112.784(5), β = 101.482(4), γ = 94.081(4)°, V = 6021.9(6) Å^3, Z = 2 [two independent C_i-symmetric complexes], D_c = 1.376 g cm^{-3}, μ(Cu-Kα) = 3.135 mm^{-1}, T = 173 K, colourless plates, Agilent Xcalibur PX Ultra A diffractometer; 22923 independent measured reflections (R_{int} = 0.0772), F^2 refinement,[5,6,7] R_1(obs) = 0.0890, wR_2(all) = 0.2920, 12546 independent observed absorption-corrected reflections [$|F_o| > 4\sigma(|F_o|)$, completeness to θ_{full}(67.7°) = 98.3%], 1299 parameters. CCDC 2202606.

The structure of **$C1^{Xy}$** was found to contain two independent complexes (**$C1^{Xy}$-A** and **$C1^{Xy}$-B**) both of which sit across a centre of symmetry at the middle of the cage. The four unique tetrafluoroborate anions were each found to be disordered. In each case two orientations were identified (of *ca*. 69:31, 76:24, 71:29 and 60:40% occupancy for the B60-, B70-, B80-, and B90-based BF_4 anions respectively), the geometries of each pair of orientations were optimised, the thermal parameters of adjacent atoms were restrained to be similar, and only the atoms of the major occupancy orientations were refined anisotropically (those of the minor occupancy orientations were refined isotropically). The included solvent was found to be highly disordered, and the best approach to handling this diffuse electron density was found to be the SQUEEZE routine of PLATON.[8] This suggested a total of 537 electrons per unit cell, equivalent to 268.5 electrons per complex. Before the use of SQUEEZE the solvent clearly resembled dimethylformamide (C_3H_7NO, 40 electrons), and 6.75 dimethylformamide molecules corresponds to 270 electrons, so this was used as the solvent present. As a result, the atom list for the asymmetric unit is low by $6.75(C_3H_7NO)$ = $C_{20.25}H_{47.25}N_{6.75}O_{6.75}$ (and that for the unit cell low by $C_{40.5}H_{94.5}N_{13.5}O_{13.5}$) compared to what is actually presumed to be present. The two largest residual electron density peaks (Q1 and Q2 of 2.22 and 2.17 eÅ^{-3} respectively), are both less than 1 Å away from palladium atoms. Three of the next four peaks (Q3, Q4 and Q6 of 1.37, 1.01 and 0.85 eÅ^{-3} respectively) are similarly within *ca*. 1.2 Å of the palladium centres. These peaks are not in chemically sensible positions, and so are highly likely to be artefacts related to residual absorption effects.

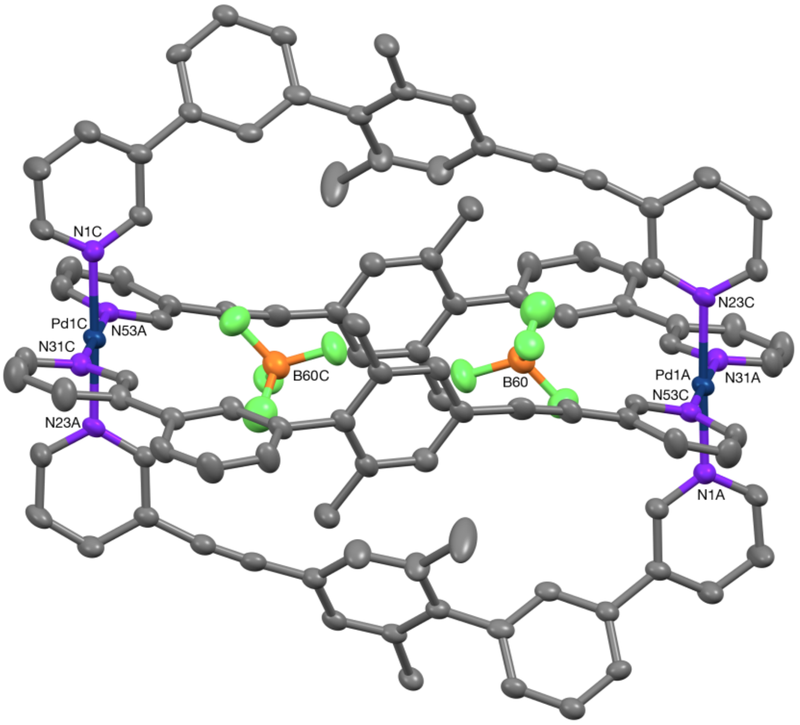

Figure S171 The structure of the C_i-symmetric complex **C1^{Xy}-A**, one of the two independent complexes present in the crystal of **C1^{Xy}**, showing the encapsulated tetrafluoroborate anions (20% probability ellipsoids).

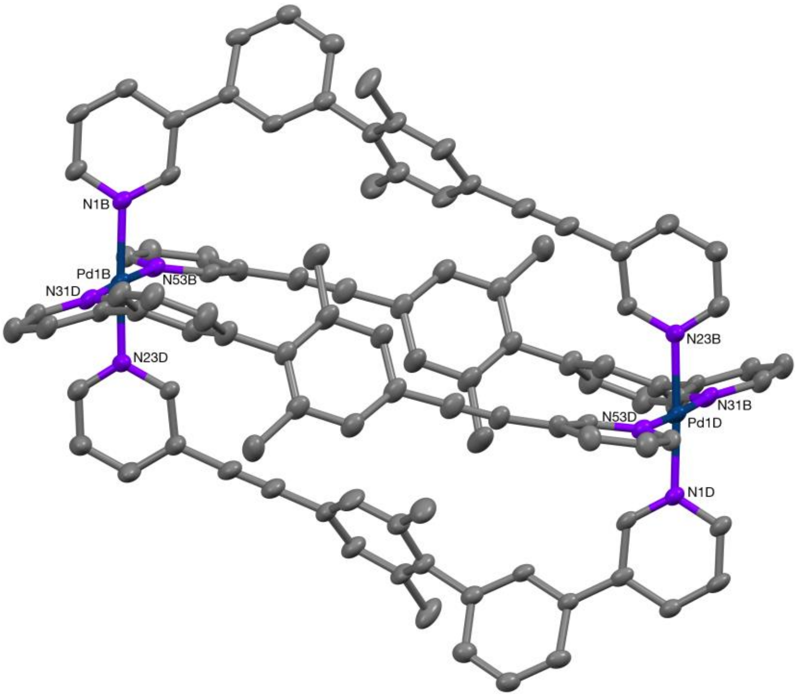

Figure S172 The structure of the C_i-symmetric complex **C1^{Xy}-B**, one of the two independent complexes present in the crystal of **C1^{Xy}** (20% probability ellipsoids).

[$Pd_2(L^{P4})_2 \supset Cl$]($BF_4$)$_3$

L^P4^ (11.0 mg, 20 µmol), [$Pd(CH_3CN)_4$](BF_4)$_2$ (8.9 mg, 20 µmol) and Bu_4NCl (2.8 mg, 10 µmol) were sonicated in d_6-DMSO (0.75 mL) until a homogenous solution was obtained. After standing at rt for 2 d the solution was diluted with DMF (0.75 mL), filtered through celite and left vapour diffusion of Et_2O. After 5 d the mother liquor was decanted off and the yellow precipitate washed with Et_2O before drying in air. This was dissolved in DMF, filtered through celite and left for vapour diffusion of Et_2O, from which X-ray quality crystals were obtained.

Crystal data for $\mathbf{C^{P4}}$: [$C_{68}H_{44}N_8O_8Pd_2$](BF_4)$_3$(Cl)·5(C_2H_6OS), *M* = 2000.43, monoclinic, *P*2/*n* (no. 13), *a* = 11.8232(6), *b* = 15.259(4), *c* = 28.9453(18) Å, β = 98.438(5)°, *V* = 5165.6(13) Å^3, *Z* = 2 [C_2 symmetry], D_c = 1.286 g cm^{-3}, μ(Cu-Kα) = 4.646 mm^{-1}, *T* = 173 K, pale yellow platy needles, Agilent Xcalibur PX Ultra A diffractometer; 9828 independent measured reflections (R_{int} = 0.0707), F^2 refinement,[5,6,7] R_1(obs) = 0.1117, wR_2(all) = 0.3999, 3162 independent observed absorption-corrected reflections [$|F_o| > 4\sigma(|F_o|)$, completeness to θ_{full}(67.7°) = 97.9%], 393 parameters. CCDC 2202605.

The structure of $\mathbf{C^{P4}}$ was found to sit across a C_2 axis that passes through the encapsulated chloride anion Cl1, and bisects the O33···O33A vector. The crystal that was studied was a weak scatterer, the full data set having a mean *I*/σ of only 2.27 for a 15 hour data collection designed for only the symmetry unique data. The drop-off of intensity with resolution is quite rapid, with the mean $F^2/\sigma(F^2)$ already dropping below 2 past *ca*. 1.25 Å. This suggests significant disorder, so it was not surprising that both the presumed tetrafluoroborate anions and the included solvent were found to be highly disordered. The best approach to handling this diffuse electron density was found to be the SQUEEZE routine of PLATON.[8] (Before the use of SQUEEZE, only two partial occupancy tetrafluoroborate anion sites could be identified amongst the extensive presumed disordered solvent peaks, and these did not refine in a sensible manner.) This suggested a total of 688 electrons per unit cell, equivalent to 344 electrons per complex. Accounting for the electron density of the presumed three tetrafluoroborate anions per complex (BF_4, 41 electrons, the Cl1 chlorine atom located at the middle of the cage providing the fourth negative charge required for charge balance) leaves 344 – (4 x 41) = 221 electrons for the solvent. With the refinements from before the use of SQUEEZE giving no clear indication as to the identity of the solvent, the most recently used solvent (dmso, C_2H_6SO, 42 electrons) was assumed; 5 dimethylsulphoxide molecules corresponds to 210 electrons, so this was used as the solvent present. As a result, the atom list for the asymmetric unit is low by 0.5 x [3(BF_4) + 5(C_2H_6SO)] = $C_5H_{15}B_{1.5}F_6O_{2.5}S_{2.5}$ (and that for the unit cell low by $C_{20}H_{60}B_6F_{24}O_{10}S_{10}$) compared to what is actually presumed to be present.

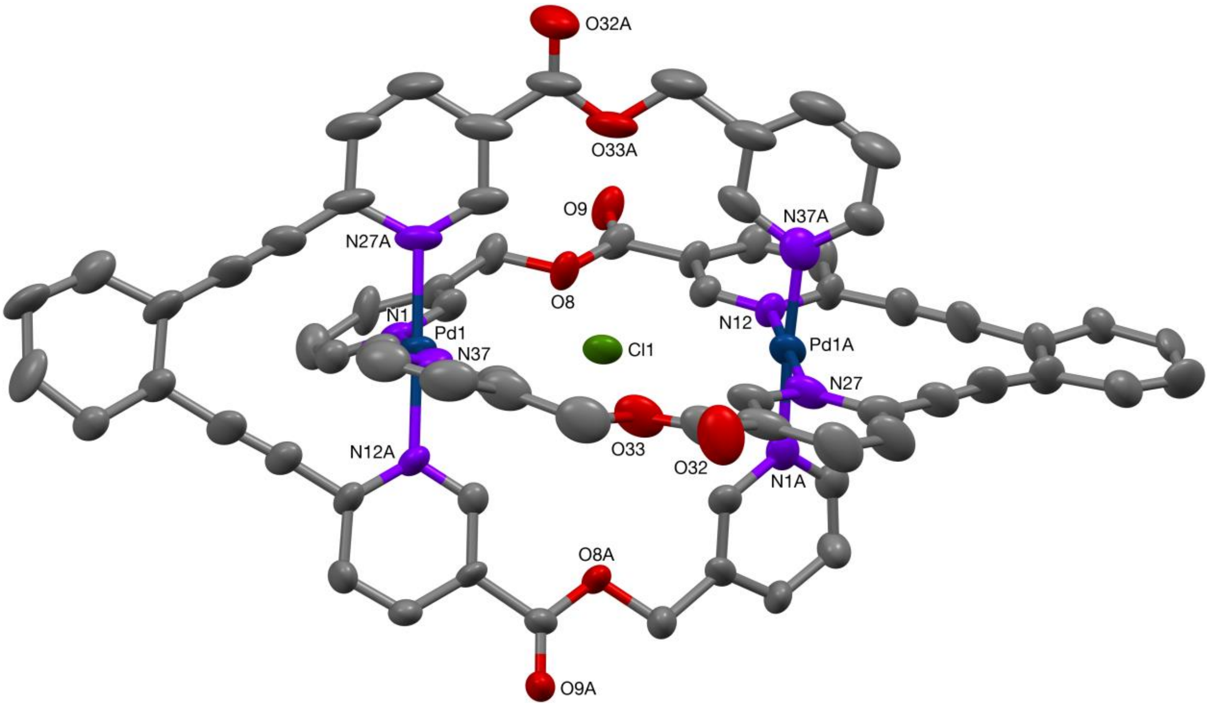

Figure S173 The crystal structure of the C_2-symmetric complex $\mathbf{C}^{\mathbf{P4}}$ showing the encapsulated chloride anion (20% probability ellipsoids).

$[Pd_2(L^{P3Q})_2 \supset NO_3](NO_3)_3$

L^P3Q^ (12.0 mg, 20 µmol) and $Pd(NO_3)_2{\cdot}2H_2O$ (5.3 mg, 20 µmol) were sonicated in d_6-DMSO (0.75 mL) until a homogenous solution was obtained. After standing at 50 °C for 2 h, ^{1}H NMR showed quantitative conversion to $[Pd_2(\mathbf{L^{P3Q}})_2](NO_3)_4$. The solution was diluted with CH_3CN (0.75 mL), filtered through celite and left vapour diffusion of Et_2O, from which X-ray quality crystals were obtained.

Crystal data for $\mathbf{C^{P3Q}}$:$[C_{76}H_{48}N_8O_8Pd_2](NO_3)_4{\cdot}3(C_2H_6OS)$, M = 1896.44, monoclinic, $I2/a$ (no. 15), a = 24.9892(14), b = 10.3668(5), c = 33.204(2) Å, β = 110.848(7)°, V = 8038.6(8) Å^3, Z = 4 [C_2 symmetry], D_c = 1.567 g cm^{-3}, µ(Cu-Kα) = 5.065 mm^{-1}, T = 173 K, pale yellow platy needles, Agilent Xcalibur PX Ultra A diffractometer; 7730 independent measured reflections (R_{int} = 0.0490), F^2 refinement,[5,6,7] R_1(obs) = 0.0557, wR_2(all) = 0.1683, 4750 independent observed absorption-corrected reflections [$|F_o| > 4\sigma(|F_o|)$, completeness to θ_{full}(67.7°) = 98.6%], 479 parameters. CCDC 2202607.

The structure of $\mathbf{C^{P3Q}}$ was found to sit across a C_2 axis that passes through the N50 and O52 atoms of the encapsulated nitrate anion, and bisects the O33···O33A vector. Three of the four expected nitrate anions per complex were reliably located, but the fourth could not be found and so has been presumed to be somewhere amongst the highly disordered solvent. The best approach to handling this diffuse electron density was found to be the SQUEEZE routine of PLATON.[8] This suggested a total of 607 electrons per unit cell, equivalent to 151.75 electrons per complex. Accounting for the electron density of the presumed "missing" nitrate anion (NO_3, 31 electrons) leaves 151.75 – 31 = 120.75 electrons for the solvent. Before the use of SQUEEZE the solvent most resembled dimethylsulphoxide (C_2H_6OS, 42 electrons), and 3 dimethylsulphoxide molecules corresponds to 126 electrons, so this was used as the solvent present. As a result, the atom list for the asymmetric unit is low by 0.5 x [NO_3 + $3(C_2H_6SO)$] = $C_3H_9N_{0.5}O_3S_{1.5}$ (and that for the unit cell low by $C_{24}H_{72}N_4O_{24}S_{12}$) compared to what is actually presumed to be present.

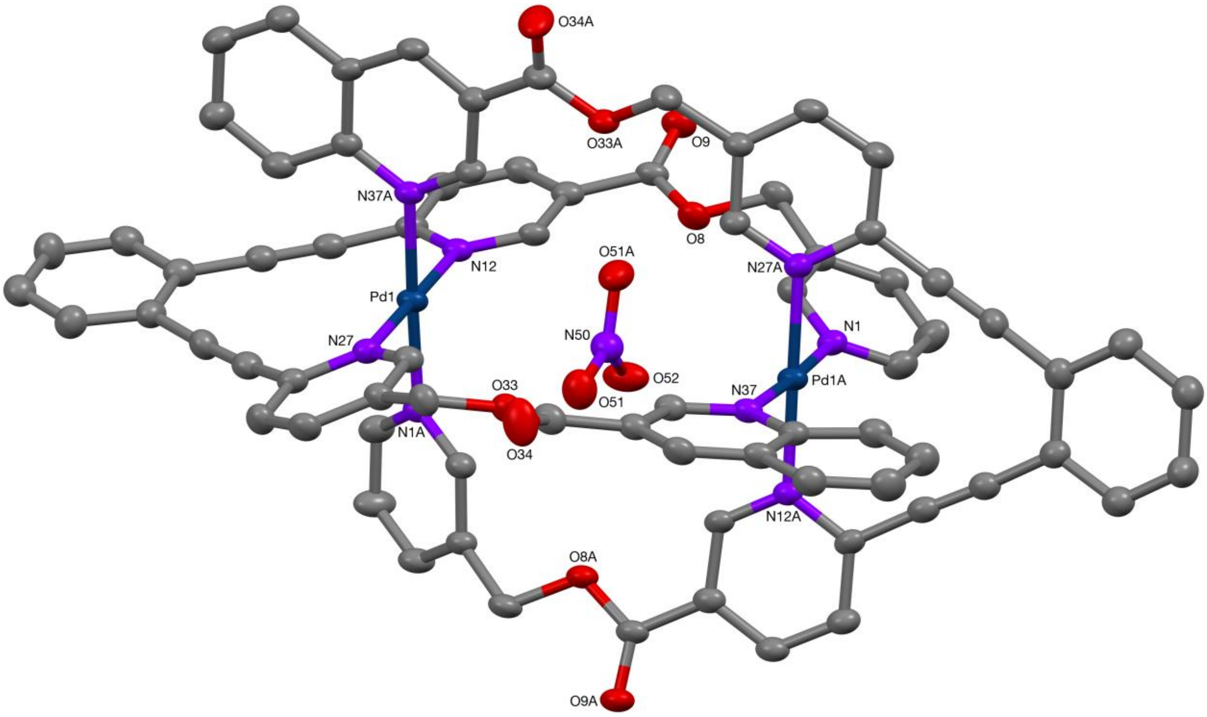

Figure S174 The crystal structure of the C_2-symmetric complex $\mathbf{C^{P3Q}}$ showing the encapsulated nitrate anion (20% probability ellipsoids).

6. Chirality Assignment in $\mathbf{C}^{P3Q}$

Priorities for the pairs of *trans*-coordinating units were assigned using CIP priority rules. These are indicated below with the higher priority shown in blue, the lower in orange. Using these, the axial chirality of each Pd(II) centre was assigned. The cage was found to crystallise as a racemic mixture of (*S*,*S*)- and (*R*,*R*)-$\mathbf{C}^{\mathbf{P3Q}}$ enantiomers (shown in pink and green, respectively).

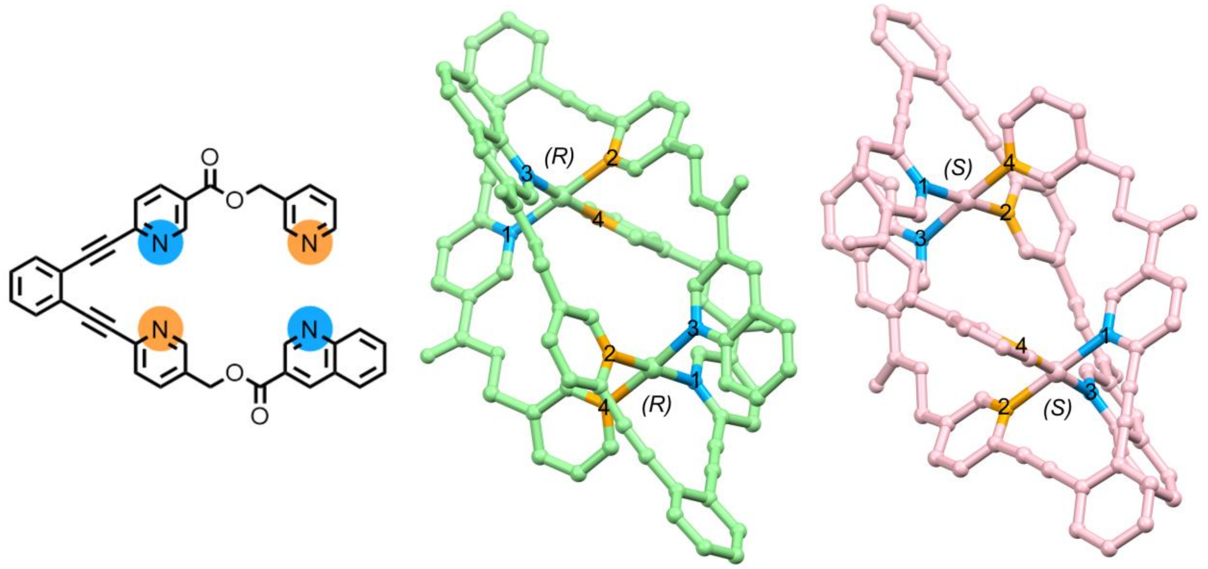

Figure S175 Chirality assignment for the (*S*,*S*)- (shown in pink) and (*R*,*R*)-$\mathbf{C}^{\mathbf{P3Q}}$ (shown in green) cage enantiomers. For each of the two *trans*-heterobidentate coordinating pairs, the higher priority coordinating nitrogen atom is shown in blue, the lower in orange.

7. References

[1] P. Reine, A. G. Campaña, L. Alvarez de Cienfuegos, V. Blanco, S. Abbate, A. J. Mota, G. Longhi, D. Miguel and J. M. Cuerva, *Chem. Commun.*, 2019, **55**, 10685-10688.

[2] J. J. P. Stewart, *J. Mol. Model.*, 2007, **13**, 1173-1213.

[3] Gaussian 16, Revision C.01, M. J. Frisch, G. W. Trucks, H. B. Schlegel, G. E. Scuseria, M. A. Robb, J. R. Cheeseman, G. Scalmani, V. Barone, G. A. Petersson, H. Nakatsuji, X. Li, M. Caricato, A. V. Marenich, J. Bloino, B. G. Janesko, R. Gomperts, B. Mennucci, H. P. Hratchian, J. V. Ortiz, A. F. Izmaylov, J. L. Sonnenberg, D. Williams-Young, F. Ding, F. Lipparini, F. Egidi, J. Goings, B. Peng, A. Petrone, T. Henderson, D. Ranasinghe, V. G. Zakrzewski, J. Gao, N. Rega, G. Zheng, W. Liang, M. Hada, M. Ehara, K. Toyota, R. Fukuda, J. Hasegawa, M. Ishida, T. Nakajima, Y. Honda, O. Kitao, H. Nakai, T. Vreven, K. Throssell, J. A. Montgomery, Jr., J. E. Peralta, F. Ogliaro, M. J. Bearpark, J. J. Heyd, E. N. Brothers, K. N. Kudin, V. N. Staroverov, T. A. Keith, R. Kobayashi, J. Normand, K. Raghavachari, A. P. Rendell, J. C. Burant, S. S. Iyengar, J. Tomasi, M. Cossi, J. M. Millam, M. Klene, C. Adamo, R. Cammi, J. W. Ochterski, R. L. Martin, K. Morokuma, O. Farkas, J. B. Foresman, and D. J. Fox, Gaussian, Inc., Wallingford CT, 2016.

[4] M. Holz, X. Mao, D. Seiferling and A. Sacco, *J. Chem. Phys.*, 1996, **104**, 669.

[5] O. V. Dolomanov, L.J. Bourhis, R.J. Gildea, J.A.K. Howard, H. Puschmann, *J. Appl. Cryst.*, 2009, **42**, 339-341.

[6] SHELXTL v5.1, Bruker AXS, Madison, WI, 1998.

[7] SHELX-2013, G.M. Sheldrick, *Acta Cryst.*, 2015, **C71**, 3-8.

[8] A.L. Spek (2003, 2009) PLATON, A Multipurpose Crystallographic Tool, Utrecht University, Utrecht, The Netherlands. See also A.L. Spek, *Acta. Cryst.*, 2015, **C71**, 9-18.
